# C–P bond formation of cyclophanyl-, and aryl halides *via* a UV-induced photo Arbuzov reaction: a versatile portal to phosphonate-grafted scaffolds†

**DOI:** 10.1039/d2ra00094f

**Published:** 2022-01-25

**Authors:** Simon Oßwald, Christoph Zippel, Zahid Hassan, Martin Nieger, Stefan Bräse

**Affiliations:** Institute of Organic Chemistry, Karlsruhe Institute of Technology (KIT) Fritz-Haber-Weg 6 76131 Karlsruhe Germany zahid.hassan@kit.edu braese@kit.edu; Department of Chemistry, University of Helsinki P.O. Box 55 00014 Helsinki Finland; Institute of Biological and Chemical Systems–Functional Molecular Systems, Karlsruhe Institute of Technology (KIT) Herman-von-Helmholtz-Platz 1, 76344 Eggenstein-Leopoldshafen Germany

## Abstract

A new versatile method for the C–P bond formation of (hetero)aryl halides with trimethyl phosphite *via* a UV-induced photo-Arbuzov reaction, accessing diverse phosphonate-grafted arenes, heteroarenes and co-facially stacked cyclophanes under mild reaction conditions without the need for catalyst, additives, or base is developed. The UV-induced photo-Arbuzov protocol has a wide synthetic scope with large functional group compatibility exemplified by over 30 derivatives. Besides mono-phosphonates, di- and tri-phosphonates are accessible in good to excellent yields. Mild and transition metal-free reaction conditions consolidate this method's potential for synthesizing pharmaceutically relevant compounds and precursors of supramolecular nanostructured materials.

## Abstract 2

UV-induced C–P bond formation of aryl halides *via* photo Arbuzov reaction: a versatile portal to phosphonate-grafted scaffolds.

## Graphical abstract

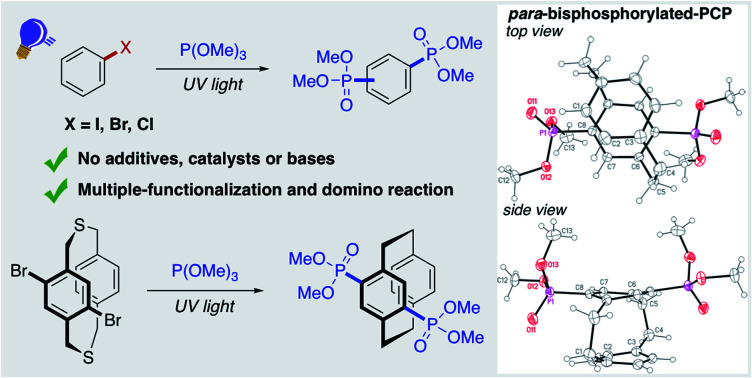

Aryl phosphonates and phosphonic acids, as an important class of organophosphorus products, have elicited enormous interest in pharmaceutical science for their biological application,^1–5^ in catalysis as precursors of privileged phosphine ligands,^6–8^ and in materials science as supramolecular tectons.^9–11^ For the synthesis of aryl phosphonates, besides conventional Grignard or organolithium protocols, the development of the palladium-catalyzed Hirao cross-coupling of H-phosphonates with aryl halides is a convenient method,^12^ and several modified protocols have been reported in recent years.^13,14^

Development of new and generally useful C–P bond formation methods with controlled selectivity that are widely applicable, cheap, and operationally simple have been an outstanding objective since phosphonylated products have a broad spectrum of applications. For instance, phosphono-fluoresceins due to the influence of the phosphoryl moiety insertion, show an almost 70-fold increase in intracellular brightness, compared to the analogous sulfono-, and carboxy-fluorescein, as recently reported by Miller and co-workers.^2^ Recent trends to realize C–P bond formation are focused on visible-light-driven reactions, where Toste and co-workers first developed a dual catalytic strategy by combining gold and ruthenium photoredox catalysis for the oxidative P-arylation of H-phosphonates.^15^ Similarly, König and co-workers reported the ruthenium-catalyzed phosphonylation of electron-rich arenes mediated by C^Ar^–H activation employing trialkyl phosphites.^16^ Metal-free variants using either organic dyes,^17–20^ hypervalent iodine reagents,^21^ or strong base^22^ are additionally emerged as a useful tool to promote C–P bond formation (Fig. 1).

**Fig. 1:**
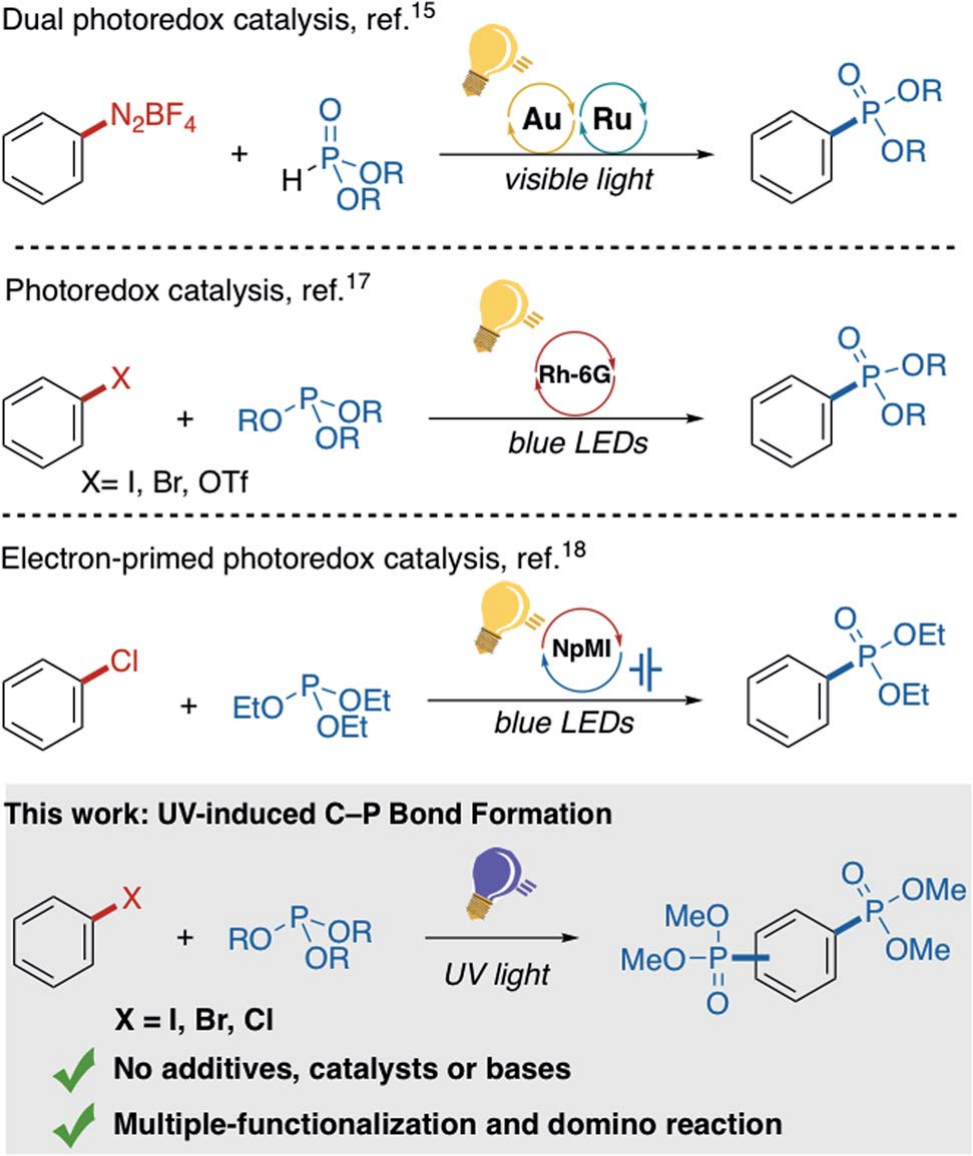
Selected methods previously reported for light-induced C–P bond formation.

Strategies that employ trialkyl phosphites have the drawback of expensive reagents, transition metal catalysts, or large catalyst loadings. The UV-induced photo-Arbuzov reaction has mostly been neglected, even though it is more than 50 years old.^23–25^ The original publication by Griffin and coworkers describes the reaction of aryl iodides with trialkyl phosphites at low temperatures (−8 to 0 °C).^24^ The phosphonylation of aryl triflates with trialkyl phosphites *via* photo-induced Arbuzov-type reaction, using UV light in combination with a base as additive has recently been realized.^26^ The need for aryl iodides and poor substrate scope foreclosed this reaction to become broadly applicable. Advancing the UV-induced photo-Arbuzov reaction for the preparation of aryl phosphonates, in this work, we report a new and more effective method for C–P bond formation under mild conditions without the need for catalysts, additives, or a base employing a wide array of functionalized aryl-, heteroaryl and thiacyclophanyl halides. The P-arylation proceeds with excellent functional group tolerance regardless of the steric hindrance and represents an important step forward in enabling C–P bond formation. Multiple and domino approach was investigated under the optimized conditions form the application perspective of the phosphonates as molecular tectons in nanostructured materials.

## Results and discussion

**Scheme 1:**
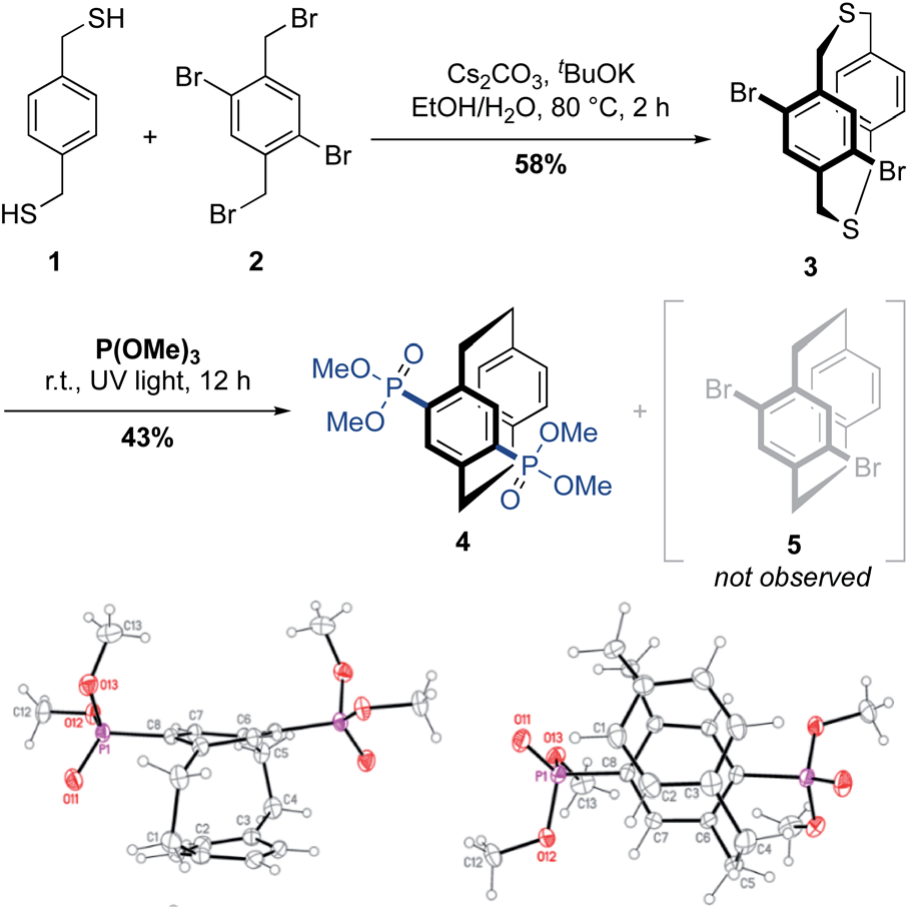
Unprecedented domino-type de-sulfurization/phosphonylation of dibromo-dithia[3.3]paracyclophane 3 towards *para*-bisphosphonylated-PCP 4. Crystal structure of product 4. Displacement parameters are drawn at a 50% probability level: top view (bottom left) and side view (bottom right).

We commenced our studies by reacting dibromo-dithia[3.3]paracyclophane 3 (Scheme 1, top), with trimethyl phosphite under UV irradiation at room temperature. The phosphonylated [2.2]paracyclophane 4 was exclusively obtained by domino-type de-sulfurization/phosphonylation, whereas the bromo-substituted [2.2]paracyclophane derivative 5 was not detected during our investigations. The C–P coupling of the bromo-substituted dithia[3.3]paracyclophane 3 is unprecedented compared to fluorinated-analogs, which under similar reaction conditions solely undergo sulphur-extrusion and no C–P bond formation occurs.^27^ Photolytic sulphur extrusion of [3.3]dithiacyclophanes is a known high yielding synthesis method of the corresponding PCP derivatives, an intriguing co-facially stacked scaffold, which is widely utilized in asymmetric catalysis, π-stacked conjugated polymers and other emerging optoelectronic materials.^28–31^ The *para*-bisphosphonylated [2.2]paracyclophane 4, obtained by this domino-type reaction, was analyzed by detailed spectroscopic techniques, and unambiguously confirmed by single-crystal X-ray diffraction analysis (Scheme 1, bottom). The precursor dithia[3.3]cyclophane 3 was prepared by cyclization of 1,4-bis(mercaptomethyl)benzene (1) and dibromo-2,5-dibromo-*p*-xylene (2) under dilution. Alternatively, enantiomerically pure planar-chiral dithia[3.3]paracyclophane derivatives can also be obtained by rhodium-catalyzed cyclization of the same two precursors.^32^

**Scheme 2:**
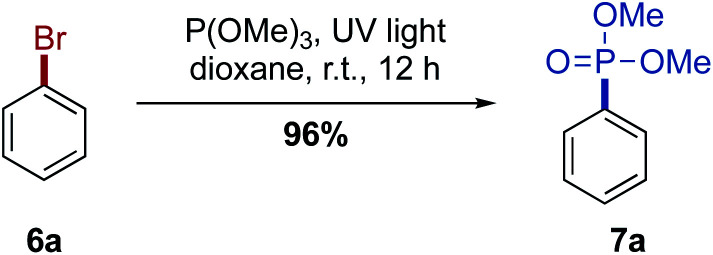
Optimized phosphonylation reaction conditions. 6a (1.0 m, 1.00 mmol), P(OMe)_3_ (5.00 mmol), UV light (245 nm, 224 W).

The phosphonylation reaction conditions were optimized using bromobenzene as test substrate (Scheme 2 and ESI, Table S1†). With 1,4-dioxane as the solvent and 5.00 equiv. trimethyl phosphite, phosphonate 7a was isolated in 96% yield. Other light sources were examined, but neither blacklight (368 nm, 160 W) nor LED UV lamps (385 nm, 64 W) or blue LEDs (471 nm, 30 W) led to any conversion, and staring material was recovered.

**Scheme 3:**
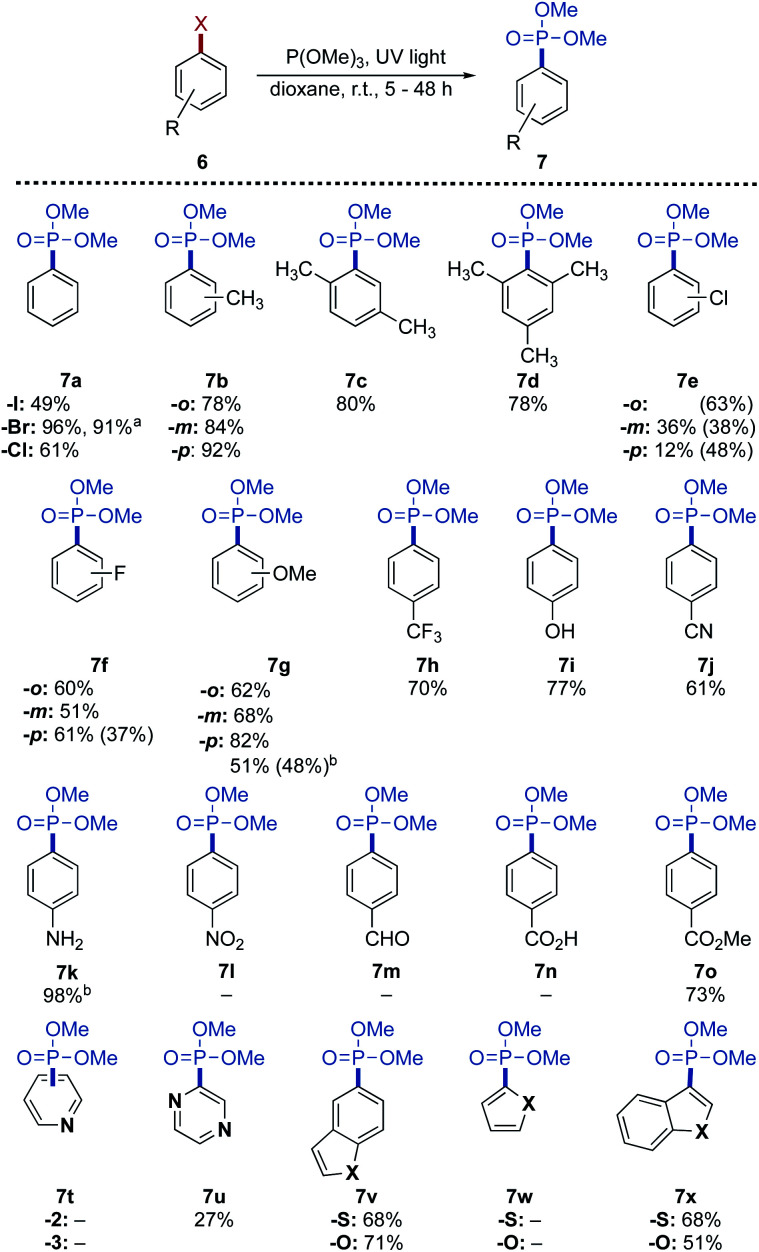
Synthetic scope of the photo-Arbuzov reaction. Aryl bromide 6 (1.0 m, 1.00 mmol), P(OMe)_3_ (5.00 mmol), UV light (245 nm, 224 W). All yields refer to the isolated yield. Yield in parentheses refers to the corresponding di-phosphonylated products. ^*a*^ conducted on a 0.1 mol scale. ^*b*^ conducted in neat P(OMe)_3_ (20.0 mmol).

To evaluate the synthetic scope and its limitations were explored based on the optimized reaction conditions (Scheme 3). Both chloro- and iodobenzene led to the phosphonylated product 7a, whereas phenyl triflate led to a complex mixture. In general, the reaction proceeds in good to excellent yield regardless of the steric hindrance (7b–7d) and electronic effects.

Chloro-substituted bromobenzenes 6e led to a mixture of mono- and di-phosphonylated products except for the *ortho*-chlorinated substrate 6e-*o*, which exclusively led to the di-phosphonylated product. The fluorinated bromobenzenes 6f gave the corresponding mono-phosphonates with a C–F bond intact, reflecting its more negative reduction potential than a C–Cl bond.^33^ The exception here is *para*-fluoro substituted bromobenzene 6f-*p*, where the di-phosphonate 6f′-*p* was isolated in 37% yield. A similar result was observed in the case of bromoanisole derivatives 6g. The *ortho*- and *meta*-substituted compounds were isolated as mono-phosphonylated products, as was the *para*-substituted derivative under the optimized conditions. Instead, when the reaction was conducted without solvent and 20 equiv. of trimethyl phosphite, the di-phosphonate 7g′-*p* was obtained in a 48% yield. In terms of functional group tolerance, products bearing trifluoromethyl, nitrile, hydroxy, amino, and ester substituents were all isolated in very good yield. The highly reactive nitro- and aldehyde functionality did not lead to any product formation, as did the carboxylic acid 6n due to its low solubility in the solvents tested in this work.

**Scheme 4:**
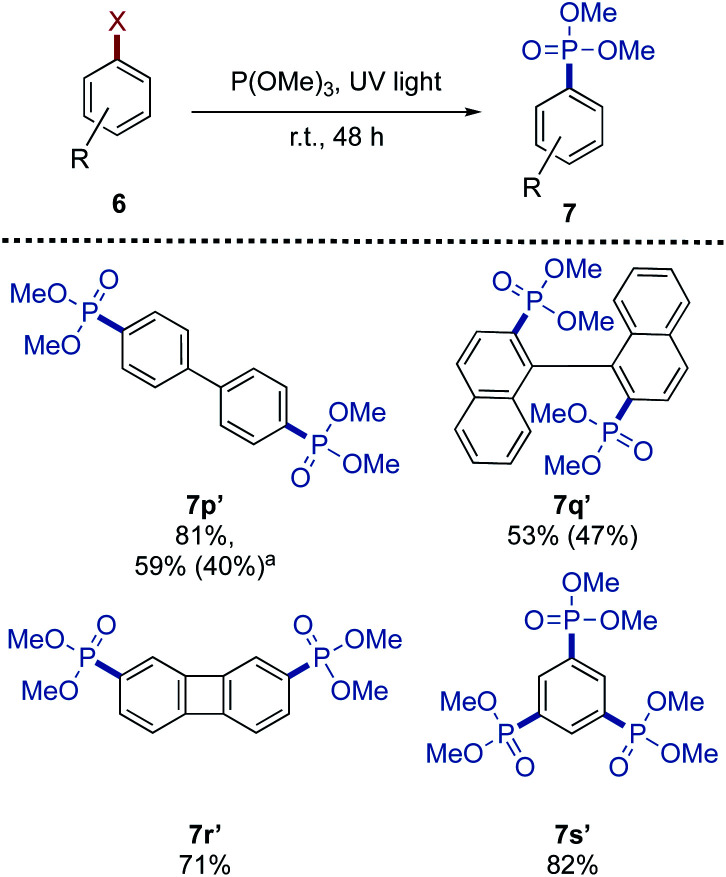
Multiple C–P bond formation. 6 (1.00 mmol), P(OMe)_3_ (20.0 mmol), UV light (245 nm, 224 W). All yields refer to the isolated yield. Yield in parentheses refers to the corresponding mono-phosphonylated products. ^*a*^Conducted with 5.00 equiv. P(OMe)_3_ in dioxane.

For further diversification studies, benzofused system of halogenated heterocycles as reaction partners to achieve the desired C–P transformation were demonstrated. Conversion towards the pyridine derivatives 7t was observed, but the final product could not be purified. The pyrazine 7u and the fused heterocycles 7v and 7x were all obtained in moderate yield. We also pursued multiple-substituted C–P coupling variants, the arylphosphonates with multiple phosphonate groups has applications as molecular tectons to constitute a series of unconventional nanostructured frameworks (MOFs and COFs) with unique properties.^34,35^ Under modified reaction conditions, *i.e.*, in neat trimethyl phosphite (20.0 equiv.), di- and tri-phosphonylation of the corresponding bromides were successfully achieved in good yield (Scheme 4).

**Scheme 5:**
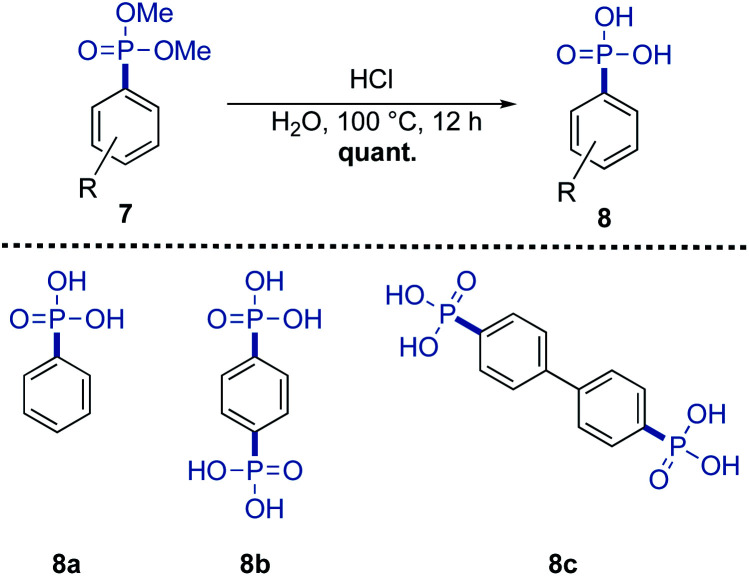
Acidic hydrolysis of the di-phosphonylated substrates.

The phosphonates 7p′–7s′ can be hydrolyzed to the corresponding phosphonic acids and can serve as potentially useful tectons. Under acidic hydrolysis, compounds 8a–c were obtained in quantitative yield without further purification (Scheme 5). The phosphonic moiety can coordinate with metals, which offers a useful library of tectons for constructing supramolecular nanostructured materials, *e.g.*, proton-conducting frameworks.^10^

In summary, we have established a UV-induced photo-Arbuzov reaction to synthesize skeletally diverse (hetero)aryl phosphonates, which avoids the use of catalysts, additives, or bases. This synthetic methodology tolerates a wide spectrum of functional groups and has been applied to a variety of aryl and heteroaryl halides. Dithia[3.3]cyclophanyl bromide, undergoes a domino-type de-sulfurization/phosphonylation. Besides, selective mono-phosphonates, di- and tri-phosphonates were prepared, and readily converted to their corresponding phosphonic acids. This transition metal-free methodology will further advance the implication of phosphonates and phosphonic acids scaffolds to construct supramolecular nanostructured materials with unique properties as well as transforming pharmaceutically relevant compounds.

## Conflicts of interest

No conflicts of interest to declare.

## Supplementary Material

RA-012-D2RA00094F-s001

RA-012-D2RA00094F-s002
