# Supplementary material for: C–P bond formation of cyclophanyl-, and aryl halides via a UV-induced photo Arbuzov reaction: a versatile portal to phosphonate-grafted scaffolds
Source: RSC Adv. 2022 Jan 25;12(6):3309–12. doi: 10.1039/d2ra00094f (PMC8979280; doi:10.1039/d2ra00094f)
Supplement: RA-012-D2RA00094F-s001 [file RA-012-D2RA00094F-s001.pdf]

Electronic Supplementary Material (ESI) for RSC Advances.
This journal is © The Royal Society of Chemistry 2022

Supporting Information

C–P Bond Formation of Cyclophanyl-, and Aryl Halides via UV-induced Photo Arbuzov Reaction: A Versatile Portal to Phosphonate-grafted Scaffolds

Simon Oßwald,[a] Christoph Zippel,[a] Zahid Hassan,[a] Martin Nieger,[b] and Stephan Bräse[a,b]*

[a] Christoph Zippel, Simon Oßwald, Dr. Zahid Hassan, and Prof. Dr. Stefan Bräse
Institute of Organic Chemistry (IOC),
Karlsruhe Institute of Technology (KIT),
Fritz-Haber-Weg 6, 76131 Karlsruhe, Germany.
E-mail: zahid.hassan@kit.edu; braese@kit.edu

http://www.ioc.kit.edu/braese

[b] Dr. Martin Nieger
Department of Chemistry,
University of Helsinki,
P. O. Box 55, 00014 University of Helsinki, Finland.

[c] Prof. Dr. Stefan Bräse
Institute of Biological and Chemical Systems–
Functional Molecular Systems (IBCS-FMS),
Karlsruhe Institute of Technology (KIT),
Hermann-von-Helmholtz-Platz 1
76344 Eggenstein-Leopoldshafen, Germany.

* Corresponding Author

1. **GENERAL INFORMATION** 3

2. **SYNTHETIC PROCEDURES** 5

General Procedure A for the Photo-Arbuzov Reaction 9

General Procedure B for the Photo-Arbuzov Reaction 9

3. **NMR SPECTRA** 36

4. **REACTION OPTIMIZATION** 98

5. **UV-REACTOR SETUP** 99

6. **TENTATIVE MECHANISM** 100

1. General Information

Materials and Methods

The starting materials, solvents, and reagents were purchased from ABCR, ACROS, ALFA AESAR, APOLLO SCIENTIFIC, CARBOLUTION, CHEMPUR, FLUKA, FLUOROCHEM, MERCK, RIEDEL-DE HAËN, SIGMA ALDRICH, STREM, TCI, or THERMO FISHER SCIENTIFIC and used without further purification unless stated otherwise.

Solvents of technical quality were purified with the solvent purification system MB SPS5 from MBRAUN. Solvents of *p.a.* quality were purchased from ACROS, FISHER SCIENTIFIC, SIGMA ALDRICH, Roth, or RIEDEL-DE HAËN and were used without further purification.

Oxygen-free solvents were obtained by freeze-pump-thaw (three cycles) technique.

Air- and moisture-sensitive reactions were carried out under argon atmosphere in oven-dried glassware using standard Schlenk techniques.

Liquids were added with a stainless-steel cannula and solids were added in powdered shape.

Reactions at low temperatures were cooled using flat dewars produced by ISOTHERM (Karlsruhe) with water/ice or isopropanol/dry ice mixtures.

Solvents were evaporated under reduced pressure at 45 °C using a rotary evaporator. For solvent mixtures, each solvent was measured volumetrically.

Flash column chromatography was performed using MERCK silica 60 (0.040 × 0.063 mm, 230–400 mesh ASTM) and quartz sand (glowed and purified with hydrochloric acid).

The RAYONET reactor Model RPR-100 with (16) 14W light bulbs (254 nm) was used for the irradiation with UV light.

Visible-light catalysis was run in a photoreactor made from LED strips (RGB LEDs, 30 W) with an adhesive back, attached to a crystallization dish (190 × 90 mm). Cooling was provided by a fan powered by a battery pack placed on the bottom of the crystallization dish. Reactions were run with blue LEDs at 471 nm (peak wavelength).

For the irradiation with a blacklight, an OMNILUX UV lamp (160W, 368 nm) was used.

For the irradiation with LED UV light (385 nm, 8 × 8W), a LZC-ICH2 photoreactor from LUZCHEM was used.

Reaction Monitoring

All reactions were monitored by thin-layer chromatography (TLC) using silica-coated aluminum plates (MERCK, silica 60, F254). UV active compounds were detected with a UV-lamp at 254 nm and 366 nm excitation.

GC-MS (gas chromatography-mass spectrometry) measurements were performed on an AGILENT TECHNOLOGIES model 6890N (electron impact ionization), equipped with an AGILENT 19091S-433 column (5% phenyl methyl siloxane, 30 m, 0.25 µm) and a 5975B VL MSD detector with a turbopump. Helium was used as a carrier gas.

Nuclear Magnetic Resonance Spectroscopy (NMR)

NMR spectra were recorded on a BRUKER Avance 400 NMR instrument at 400 MHz for ^{1}H NMR, 101 MHz for ^{13}C NMR, 376 MHz for ^{19}F NMR, and 162 MHz for ^{31}P NMR, or a BRUKER Avance 500 NMR instrument at 500 MHz for ^{1}H NMR, 126 MHz for ^{13}C NMR and 470 MHz for ^{19}F NMR and 202 MHz for ^{31}P NMR.

The NMR spectra were recorded at room temperature in deuterated solvents acquired from EURISOTOP, SIGMA ALDRICH, or DEUTERO. The chemical shift δ is displayed in parts per million [ppm] and the references used were the ^{1}H and ^{13}C peaks of the solvents themselves:

d_1-chloroform ($CDCl_3$): 7.26 ppm for ^{1}H and 77.16 ppm for ^{13}C

d_6-dimethyl sulfoxide (DMSO-d_6): 2.50 ppm for ^{1}H and 39.52 ppm for ^{13}C

For the characterization of centrosymmetric signals, the signal's median point was chosen, for multiplets the signal range. The following abbreviations were used to describe the proton splitting pattern: d = doublet, t = triplet, m = multiplet, dd = doublet of doublet, ddd = doublet of doublet of doublet, dddd = doublet of doublet of doublet of doublet, dt = doublet of triplet. Absolute values of the coupling constants "*J*" are given in Hertz [Hz] in absolute value and decreasing order. Signals of the ^{13}C spectrum were assigned by distortionless enhancement by polarization transfer (DEPT) spectra DEPT90 and DEPT135 or phase edited heteronuclear single quantum coherence (HSQC) and was specified in the following way: + = primary or tertiary carbon atoms (positive phase), – = secondary carbon atoms (negative phase), C_q = quaternary carbon atoms (no signal).

Infrared Spectroscopy (IR)

The infrared spectra were recorded with a BRUKER, Alpha P instrument. All samples were measured by attenuated total reflection (ATR). The positions of the absorption bands are given in wavenumbers $\tilde{\nu}$ in cm^{-1} and were measured in the range from 3600 cm^{-1} to 500 cm^{-1}.

Characterization of the absorption bands was done in dependence of the absorption strength with the following abbreviations: vs (very strong, 0−9%), s (strong, 10−39%), m (medium, 40−69%), w (weak, 70−89%), vw (very weak, 90−100%).

Mass Spectrometry (MS)

Electron ionization (EI) and fast atom bombardment (FAB) experiments were conducted using a FINNIGAN, MAT 90 (70 eV) instrument, with 3-nitrobenzyl alcohol (3-NBA) as matrix and reference for high resolution. For the interpretation of the spectra, molecular peaks $[M]^+$, peaks of protonated molecules $[M+H]^+$ and characteristic fragment peaks are indicated with their mass-to-charge ratio (*m/z*) and their intensity in percent, relative to the base peak (100%) is given. In case of high-resolution measurements, the maximum tolerated error is ±5 ppm.

2. Synthetic Procedures

1,4-Phenylenedimethanethiol (1)

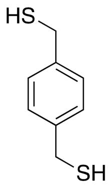

1,4-Bis(bromomethyl)benzene (10.0 g, 38.0 mmol, 1.00 equiv.) and thiourea (5.77 g, 76 mmol, 2.00 equiv.) were dissolved in anhydrous, degassed ethanol (100 mL) and stirred for 4 hours. The solvent was removed under reduced pressure to afford the crude thiouronium salt as a colorless solid. Sodium hydroxide (degassed 5.0 M aq. solution, 30 mL) was added and the reaction was heated to 100 °C for 30 min. The solution was cooled to 0 °C and hydrochloric acid (6.0 M aq. solution) was added until acidic (pH = 2). The solution was diluted with water (100 mL) and chloroform (200 mL). The phases were separated, and the aqueous phase was extracted with chloroform (3 × 100 mL). The combined organic layers were dried over sodium sulfate and the solvent was removed under reduced pressure to afford the title compound (6.40 g, 37.6 mmol, 99%) as a clear oil that solidifies upon prolonged standing.

R_f = 0.74 (CyHex/EtOAc, 2:1)

^{1}H NMR (400 MHz, $CDCl_3$, ppm) δ = 7.28 (s, 4H, H_{Ar}), 3.73 (d, J = 7.5 Hz, 4H, CH_2), 1.75 (t, J = 7.5 Hz, 2H, SH).

^{13}C NMR (101 MHz, $CDCl_3$, ppm) δ = 140.1 (C_q, 2C, C_{Ar}), 128.5 (+, 4C, CH, C_{Ar}), 28.7 (–, 2C, CH_2).

IR (ATR, cm^{-1}) $\tilde{\nu}$ = 3050 (vw), 3024 (w), 3003 (w), 2962 (w), 2928 (w), 2546 (w), 1911 (vw), 1796 (vw), 1687 (w), 1572 (w), 1510 (s), 1428 (m), 1418 (s), 1357 (w), 1254 (m), 1210 (w), 1163 (w), 1106 (m), 1021 (w), 967 (w), 839 (vs), 754 (vs), 669 (vs), 620 (w), 615 (w), 555 (w), 516 (vs), 452 (vw).

MS (FAB, 3-NBA, %) m/z = 171 (7) $[M+H]^+$, 170 (90) $[M]^+$, 137 (100) $[M–SH]^+$, 104 (100) $[M–S_2H_2]^+$.

HRMS (FAB, $[M]^+$, $C_8H_{10}{}^{32}S_2$) calcd.: 170.0224; found: 170.0223.

Additional information on the chemical synthesis is available *via* the Chemotion repository:

https://dx.doi.org/10.14272/reaction/SA-FUHFF-UHFFFADPSC-IYPNRTQAOX-UHFFFADPSC-NUHFF-NUHFF-NUHFF-ZZZ

Additional information on the analysis of the target compound is available *via* the Chemotion repository:

https://dx.doi.org/10.14272/IYPNRTQAOXLCQW-UHFFFAOYSA-N.1

Dimethyl 2,5-dibromobenzene-1,4-dicarboxylate

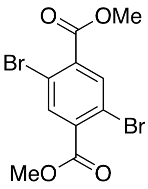

2,5-Dibromoterephthalic acid (5.00 g, 15.4 mmol, 1.00 equiv.) was dissolved in thionyl chloride (18 mL) and *N*,*N*-dimethylformamide (28.2 mg, 29.9 µL, 386 µmol, 2.5 mol%) was added. The mixture was heated to 100 °C for 5 h. Thionyl chloride was removed by distillation and the crude oil was cooled to 0 °C. Methanol (14.0 mL) and triethylamine (6.5 mL) were slowly added. After 2 h, the solvent was removed under reduced pressure and the residue was diluted with ethyl acetate (100 mL), washed with hydrochloric acid (1.0 M aq. solution, 50 mL), sodium hydrogen carbonate (sat.

aq. solution, 100 mL) and dried over sodium sulfate. The solvent was removed under reduced pressure to obtain the title compound (4.35 g, 12.4 mmol, 80%) as a colorless solid

m.p.: 143 °C

R_f = 0.67 (CyHex/EtOAc/CH_2Cl_2, 8:1:1)

^{1}H NMR (500 MHz, $CDCl_3$, ppm) δ = 7.99 (s, 2H, H_{Ar}), 3.89 (s, 6H, CO_2CH_3).

^{13}C NMR (126 MHz, $CDCl_3$, ppm) δ = 164.7 (C_q, 2C, CO_2CH_3), 136.8, (+, 2C, CH, C_{Ar}) 135.5 (C_q, 2C, C_{Ar}), 120.3 (C_q, 2C, *C*Br), 53.1 (+, CH_3, 2C, CO_2CH_3).

IR (ATR, cm^{-1}) $\tilde{\nu}$ = 3095 (w), 3037 (w), 3010 (w), 2953 (w), 2895 (w), 2874 (w), 2827 (w), 1800 (vw), 1732 (vs), 1647 (w), 1585 (w), 1557 (vw), 1531 (vw), 1468 (m), 1446 (w), 1421 (s), 1322 (w), 1288 (vs), 1239 (vs), 1184 (vs), 1119 (vs), 1054 (vs), 976 (m), 952 (vs), 908 (vs), 880 (m), 817 (s), 773 (vs), 697 (w), 680 (m), 635 (w), 601 (m), 560 (s), 509 (s), 456 (s), 436 (m), 425 (w), 404 (w), 378 (vs).

MS (FAB, 3-NBA, %) *m/z* = 355/353/351 (8/16/8) $[M+2H]^+$.

HRMS (EI, $[M+H]^+$, $C_{10}H_9O_4{}^{79}Br_2$) calcd.: 350.8862, found: 350.8864.

1,4-Dibromo-2,5-bis(bromomethyl)benzene (2)

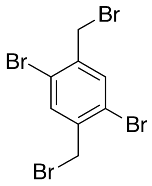

Dimethyl 2,5-dibromobenzene-1,4-dicarboxylate (4.20 g, 11.9 mmol, 1.00 equiv.) was dissolved in THF (28 mL) and cooled to 0°C. Lithium borohydride (635 mg, 35.8 mmol, 3.00 equiv.) and methanol (1.0 mL) was added. The reaction mixture was stirred at room temperature for 18 h and acidified with hydrochloric acid (1 M aq. solution) to pH 6. The mixture was diluted with dichloromethane (100 mL) and the phases were separated. The aqueous phase was extracted with dichloromethane (2 × 50 mL) and the combined organic layers were washed with sodium hydrogen carbonate (sat. aq. solution, 100 mL) and dried over sodium sulfate to obtain the title compound which was used without further purification in the next step.

The crude [2,5-Dibromo-4-(hydroxymethyl)phenyl]methanol (3.40 g, 11.5 mmol, 1.00 equiv.) was dissolved in anhydrous THF (120 mL) and cooled to 0 °C. Phosphorous tribromide (6.84 g, 2.40 mL, 25.3 mmol, 2.20 equiv.) was added and the mixture was warmed to room temperature. After 48 h, water (50 mL) was carefully added, and the mixture was stirred for 10 min. The mixture was diluted with ethyl acetate (100 mL) and the phases were separated. The aqueous phase was extracted with ethyl acetate (2 × 50 mL), the combined organic layers were washed with sodium hydrogen carbonate (sat. aq. solution, 3 × 100 mL) and dried over sodium sulfate. The solvent was removed under reduced pressure and the crude solid was recrystallized from ethanol to obtain the title compound (3.75 g, 8.89 mmol, 77%) as a colorless solid.

R_f = 0.26 (*n*-pentane)

^{1}H NMR (500 MHz, $CDCl_3$, ppm) δ = 7.66 (s, 2H, H_{Ar}), 4.51 (s, 4H, CH_2Br).

^{13}C NMR (126 MHz, $CDCl_3$, ppm) δ = 139.1 (C_q, 2C, C_{Ar}), 135.5 (+, 2C, CH, C_{Ar}), 123.4 (C_q, 2C, *C*Br), 31.6 (–, 2C, CH_2).

IR (ATR, cm^{-1}) $\tilde{\nu}$ = 3033 (w), 1781 (w), 1473 (m), 1434 (m), 1358 (s), 1282 (w), 1217 (vs), 1190 (m), 1169 (w), 1061 (vs), 898 (vs), 867 (vs), 792 (w), 779 (s), 739 (m), 704 (m), 674 (vs), 588 (w), 555 (w), 537 (vs), 475 (m), 455 (vs), 438 (s), 418 (m), 401 (m), 384 (m).

MS (EI, 70 eV, 80 °C, %) *m/z* = 425/423/421/419/417 (3/14/23/16/4) $[M]^+$, 344/342/240/338 (33/95/100/34) $[M–Br]^+$, 264/262/260 (32/64/34) $[M+H–Br]^+$, 102 (62) $[M–Br_4]$.

HRMS (EI, $[M]^+$, $C_8H_6{}^{79}Br_4$) calcd.: 417.7197, found: 417.7195.

5,8-Dibromo-2,11-dithia[3.3]paracyclophane (3)

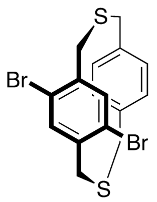

1,4-Dibromo-2,5-bis(bromomethyl)benzene (2.97 g, 7.04 mmol, 1.00 equiv.) and 1,4-bis(mercaptomethyl) benzene (1.20 g, 7.05 mmol, 1.00 equiv.) were dissolved in THF (500 mL). The solution was added to a suspension of potassium carbonate (2.23 g, 16.1 mmol, 2.30 equiv.) in ethanol (3.0 L) at 80 °C, under an argon atmosphere. After the addition was complete (8 h), the mixture was heated for 1 h and the solvent was removed under reduced pressure. The crude solid was dissolved in dichloromethane (500 mL) and washed with water (2 × 150 mL). The organic layer was dried over sodium sulfate and the solvent was removed under reduced pressure. The crude solid was purified over a short silica plug (*n*-pentane/CH_2Cl_2, 1:1) and then recrystallized from toluene. The title compound (1.40 g, 3.25 mmol, 46%) was obtained as colorless crystals.

R_f = 0.55 (*n*-pentane/CH_2Cl_2, 1:1)

^{1}H NMR (400 MHz, DMSO-d_6, ppm) δ = 7.23 (s, 2H, H_{Ar}), 7.13 (dd, J = 7.9, 2.0 Hz, 2H, H_{Ar}), 6.91 (dd, J = 7.8, 2.0 Hz, 2H, H_{Ar}), 3.91 – 3.79 (m, 8H, H_{Pc}).

^{13}C NMR (101 MHz, DMSO-d_6, ppm) δ = 136.8 (C_q, 2C, C_{Ar}), 135.0 (C_q, 2C, C_{Ar}), 135.0 (+, 2C, CH, C_{Ar}), 129.1 (+, 2C, CH, C_{Ar}), 127.7 (+, 2C, CH, C_{Ar}), 122.8 (C_q, 2C, C_{Ar}Br), 36.4 (–, 2C, CH_2), 35.6(–, 2C, CH_2).

IR (ATR, cm^{-1}) $\tilde{\nu}$ = 2972 (w), 2928 (w), 2911 (m), 2894 (w), 2868 (w), 2853 (w), 1735 (w), 1591 (w), 1507 (w), 1466 (w), 1446 (w), 1421 (s), 1401 (m), 1381 (w), 1354 (s), 1279 (w), 1222 (w), 1191 (w), 1118 (w), 1099 (m), 1045 (vs), 1017 (m), 943 (w), 898 (m), 873 (vs), 844 (m), 819 (m), 807 (m), 785 (m), 765 (m), 738 (s), 717 (m), 693 (s), 649 (m), 550 (m), 504 (w), 469 (w), 438 (m), 425 (w), 404 (m).

MS (ESI^+, %) *m/z* = 432/430/428 (41/80/38) $[M+H]^+$, 324/322/320 (13/29/14) $[M–C_8H_7]^+$.

HRMS (ESI, $[M+H]^+$, $C_{16}H_{14}{}^{79}Br_2\ S_2$) calcd.: 428.8977, found: 428.8974.

Tetramethyl [2.2]paracyclophane-2,7-diylbis(phosphonate) (4)

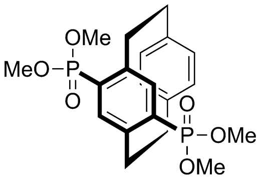

5,8-Dibromo-2,11-dithia[3.3]paracyclophane (1.40 g, 3.25 mmol, 1.00 equiv.) was suspended in degassed trimethyl phosphite (87 mL) in a quartz flask and irradiated with UV light (254 nm, 224 W) at room temperature for 24 h. Trimethyl phosphite was removed under reduced pressure and the residue was purified by column chromatography (silica, CH_2Cl_2/acetone, 9:1 to 1:1) to yield the title compound (600 mg, 1.41 mmol, 43%) as a colorless solid.

R_f = 0.13 (*n*-pentane/EtOAc, 2:1 + 5% CH_3OH)

^{1}H NMR (500 MHz, $CDCl_3$, ppm) δ = 7.04 (dd, *J* = 15.0, 5.9 Hz, 2H, H^{Ar}), 6.83 (dd, *J* = 7.9, 2.0 Hz, 2H, H^{Ar}), 6.47 (dd, *J* = 7.9, 2.0 Hz, 2H, H^{Ar}), 3.77 (dd, *J* = 11.0, 10.1 Hz, 12H, POCH_3), 3.74 – 3.69 (m, 2H, H^{Pc}), 3.26 (ddd, *J* = 12.6, 10.5, 5.6 Hz, 2H, H^{Pc}), 3.13 (ddd, *J* = 12.7, 10.6, 2.0 Hz, 2H, H^{Pc}), 3.01 (ddd, *J* = 13.1, 10.6, 5.5 Hz, 2H, H^{Pc}).

^{13}C NMR (126 MHz, $CDCl_3$, ppm) δ = 144.97 – 144.37 (m, 2C, C_q, C^{Ar}), 139.39 (2C, C_q, C^{Ar}), 138.87 – 138.44 (+, m, 2C, CH, C^{Ar}), 132.44 (+, 2C, CH, C^{Ar}), 132.37 (+, 2C, CH, C^{Ar}), 130.42 (dd, *J* = 189.3, 4.0 Hz, 2C, C_q, C^{Ar}), 53.33 – 51.19 (+, m, 4C, CH_3, POCH_3), 35.27 – 35.11 (–, m, 2C, CH_2), 35.04 (–, 2C, CH_2).

^{31}P NMR (202 MHz, $CDCl_3$, ppm) δ = 19.38 – 18.83 (m).

IR (ATR, cm^{-1}) $\tilde{\nu}$ = 3016 (vw), 3003 (vw), 2995 (vw), 2956 (w), 2945 (w), 2931 (w), 2894 (vw), 2853 (w), 2840 (w), 1504 (vw), 1456 (w), 1438 (w), 1415 (vw), 1366 (w), 1251 (vs), 1215 (w), 1204 (w), 1179 (m), 1153 (w), 1116 (m), 1016 (vs), 956 (m), 921 (w), 892 (w), 874 (w), 823 (vs), 807 (vs), 761 (vs), 734 (s), 722 (vs), 696 (w), 594 (vs), 565 (s), 545 (m), 521 (s), 479 (s), 433 (s), 405 (vs), 392 (s).

MS (EI, 70 eV, 140 °C, %) *m/z* = 424 (100) $[M+H]^+$, 320 (6) $[M–C_8H_8]^+$, 104 (49) $[C_8H_8]^+$.

HRMS (EI, $[M]^+$, $C_{20}H_{26}O_6P_2$) calcd.: 424.1199; found: 424.1201.

Additional information on the chemical synthesis is available *via* the Chemotion repository:

https://dx.doi.org/10.14272/reaction/SA-FUHFF-UHFFFADPSC-GMDSCBQOTF-UHFFFADPSC-NUHFF-NUHFF-NUHFF-ZZZ

Additional information on the analysis of the target compound is available *via* the Chemotion repository:

https://dx.doi.org/10.14272/GMDSCBQOTFRVGY-UHFFFAOYSA-N.1

General Procedure A for the Photo-Arbuzov Reaction

In a photochemical reactor system (RAYONET reactor Model RPR-100) the corresponding aryl bromide (1.00 mmol) was dissolved in trimethyl phosphite (591 µL, 5.00 mmol, 5.00 equiv.) and dioxane (1.0 mL) in a quartz flask and irradiated with UV light (254 nm, 224 W) at room temperature until the starting material was consumed. The solvent and excess trimethyl phosphite were removed under reduced pressure and the residue was purified by flash column chromatography (silica, *n*-pentane/EtOAc, 2:1 + 5% MeOH to 2:1 + 10% MeOH).

General Procedure B for the Photo-Arbuzov Reaction

In a photochemical reactor system (RAYONET reactor Model RPR-100) the corresponding aryl bromide (1.00 mmol) was dissolved in trimethyl phosphite (2.36 mL) in a quartz flask and irradiated with UV light (254 nm, 224 W) at room temperature until the starting material was consumed. Excess trimethyl phosphite was removed by short-path distillation (0.3 mbar, 50 °C) and the residue was purified by flash column chromatography (silica, *n*-pentane/EtOAc, 2:1 + 5% MeOH to 2:1 + 10% MeOH).

Dimethyl phenylphosphonate (7a)

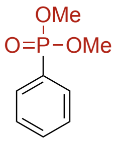

According to the general procedure A, bromobenzene (157 mg, 105 µL) was irradiated for 18 h. The title compound (176 mg, 946 µmol, 95%) was obtained as a colorless oil.

R_f = 0.27 (*n*-pentane/EtOAc, 2:1 + 5% CH_3OH)

^{1}H NMR (500 MHz, $CDCl_3$, ppm) δ = 7.82 – 7.75 (m, 2H, H^{Ar}), 7.58 – 7.52 (m, 1H, H^{Ar}), 7.48 – 7.42 (m, 2H, H^{Ar}), 3.74 (d, *J* = 11.1 Hz, 6H, POCH_3).

^{13}C NMR (126 MHz, $CDCl_3$, ppm) δ = 132.73 (d, *J* = 2.8 Hz, C_q, C^{Ar}), 131.98 (+, d, *J* = 9.9 Hz, 2C, CH, C^{Ar}), 128.65 (+, d, *J* = 15.4 Hz, 2C, CH, C^{Ar}), 127.03 (d, *J* = 188.8 Hz, C_q, C^{Ar}), 52.77 (+, d, *J* = 5.9 Hz, 2C, CH_3, POCH_3).

^{31}P NMR (202 MHz, $CDCl_3$, ppm) δ = 22.03 – 21.36 (m).

IR (ATR, cm^{-1}) $\tilde{\nu}$ = 3529 (vw), 3486 (vw), 2953 (w), 2850 (vw), 1594 (w), 1485 (w), 1459 (w), 1439 (m), 1248 (vs), 1183 (m), 1130 (vs), 1020 (vs), 881 (w), 826 (vs), 788 (vs), 751 (vs), 697 (vs), 640 (w), 629 (w), 618 (w), 558 (vs), 514 (s), 456 (m), 439 (w), 425 (m), 384 (w).

MS (EI, 70 eV, 20 °C, %) *m/z* = 186 (97) $[M]^+$, 185 (100) $[M–H]^+$, 156 (32) $[C_6H_6O_3P]^+$, 110 (25) $[C_2H_7O_3P]^+$, 79 (28) $[C_6H_7]^+$.

HRMS (EI, $[M]^+$, $C_8H_{11}O_3P$) calcd.: 186.0440; found: 186.0440.

Additional information on the chemical synthesis is available *via* the Chemotion repository:

https://dx.doi.org/10.14272/reaction/SA-FUHFF-UHFFFADPSC-OXDOANYFRL-UHFFFADPSC-NUHFF-NUHFF-NUHFF-ZZZ

Additional information on the analysis of the target compound is available *via* the Chemotion repository:

https://dx.doi.org/10.14272/OXDOANYFRLHSML-UHFFFAOYSA-N.1

Dimethyl *o*-tolylphosphonate (7b-*o*)

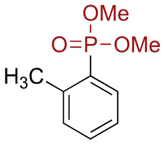

According to the general procedure A, 2-bromotoluene (171 mg, 120 µL) was irradiated for 18 h. The title compound (157 mg, 784 µmol, 78%) was obtained as a colorless oil.

R_f = 0.41 (*n*-pentane/EtOAc, 2:1 + 10% CH_3OH)

^{1}H NMR (500 MHz, $CDCl_3$, ppm) δ = 7.88 (ddd, *J* = 14.4, 7.9, 1.5 Hz, 1H, H^{Ar}), 7.43 (tt, *J* = 7.5, 1.5 Hz, 1H, H^{Ar}), 7.31–7.22 (m, 2H, H^{Ar}), 3.75 (d, *J* = 11.2 Hz, 6H, POCH_3), 2.55 (d, *J* = 1.8 Hz, 3H, CH_3).

^{13}C NMR (126 MHz, $CDCl_3$, ppm) δ = 142.1 (d, *J* = 10.4 Hz, C_q, C^{Ar}), 134.2 (+, d, *J* = 10.3 Hz, C_H, C^{Ar}), 132.8 (d, *J* = 3.1 Hz, C_H, C^{Ar}), 131.4 (d, *J* = 15.0 Hz, C_H, C^{Ar}), 125.6 (d, *J* = 184.7 Hz, C_q, C^{Ar}), 125.5 (+, d, *J* = 14.9 Hz, C_H, C^{Ar}), 52.6 (d, *J* = 5.8 Hz, 2C, CH_3, POCH_3), 21.3 (+, d, *J* = 3.6 Hz, CH_3, CH_3).

^{31}P NMR (202 MHz, $CDCl_3$, ppm) δ = 22.80 – 22.13 (m).

IR (ATR, cm^{-1}) $\tilde{\nu}$ = 3526 (vw), 3482 (vw), 3465 (vw), 3456 (vw), 3442 (vw), 3434 (vw), 3414 (vw), 3357 (vw), 3012 (vw), 2953 (w), 2850 (vw), 1875 (vw), 1854 (vw), 1846 (vw), 1829 (vw), 1803 (vw), 1795 (vw), 1786 (vw), 1775 (vw), 1752 (vw), 1735 (vw), 1720 (vw), 1707 (vw), 1703 (vw), 1655 (w), 1638 (w), 1632 (w), 1596 (w), 1578 (vw), 1570 (w), 1553 (vw), 1544 (vw), 1534 (vw), 1527 (vw), 1510 (vw), 1475 (w), 1452 (w), 1384 (w), 1279 (w), 1235 (s), 1201 (w), 1181 (m), 1143 (m), 1123 (w), 1091 (m), 1018 (vs), 880 (w), 826 (vs), 813 (vs), 756 (vs), 717 (m), 683 (m), 647 (m), 633 (m), 623 (m), 575 (vs), 533 (vs), 477 (s), 453 (s), 432 (m), 421 (m), 401 (m), 387 (m).

MS (EI, 70 eV, 20 °C, %) *m/z* = 200 (100) $[M]^+$, 199 (67) $[M–H]^+$, 185 (48) $[C_8H_{10}O_3P]^+$, 91 (43) $[C_7H_7]^+$.

HRMS (EI, $[M]^+$, $C_9H_{13}O_3P$) calcd.: 200.0597; found: 200.0597.

Additional information on the chemical synthesis is available *via* the Chemotion repository:

https://dx.doi.org/10.14272/reaction/SA-FUHFF-UHFFFADPSC-GCSOCNSAUQ-UHFFFADPSC-NUHFF-NUHFF-NUHFF-ZZZ

Additional information on the analysis of the target compound is available *via* the Chemotion repository:

https://dx.doi.org/10.14272/GCSOCNSAUQLHSB-UHFFFAOYSA-N.1

Dimethyl *m*-tolylphosphonate (7b-*m*)

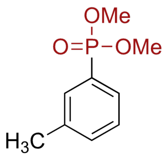

According to the general procedure A, 3-bromotoluene (171 mg, 121 µL) was irradiated for 18 h. The title compound (169 mg, 844 µmol, 84%) was obtained as a colorless oil.

R_f = 0.38 (*n*-pentane/EtOAc, 2:1 + 10% CH_3OH)

^1H NMR (500 MHz, $CDCl_3$, ppm) δ = 7.65–7.53 (m, 2H, H^{Ar}), 7.41–7.30 (m, 2H, H^{Ar}), 3.75 (d, *J* = 11.1 Hz, 6H, $POCH_3$), 2.39 (d, *J* = 0.9 Hz, 3H, CH_3).

^{13}C NMR (126 MHz, $CDCl_3$, ppm) δ = 138.6 (d, *J* = 14.7 Hz, C_q, C_{Ar}), 133.6 (+, d, *J* = 2.8 Hz, CH, C^{Ar}), 132.5 (+, d, *J* = 10.0 Hz, CH, C^{Ar}), 129.1 (+, d, *J* = 9.9 Hz, CH, C^{Ar}), 128.6 (+, d, *J* = 15.6 Hz, CH, C^{Ar}), 126.8 (d, *J* = 187.2 Hz, Cq, C^{Ar}), 52.8 (+, d, *J* = 5.5 Hz, 2C, CH_3, $POCH_3$), 21.4 (+, CH_3, CH_3).

^{31}P NMR (202 MHz, $CDCl_3$, ppm) δ = 22.15 (dh, J = 23.4, 12.2 Hz).

IR (ATR, cm^{-1}) $\tilde{\nu}$ = 3471 (w), 3459 (w), 3451 (w), 3434 (w), 3425 (w), 3019 (vw), 2996 (vw), 2953 (w), 2922 (vw), 2873 (vw), 2850 (w), 1863 (vw), 1833 (vw), 1812 (vw), 1751 (vw), 1734 (vw), 1701 (vw), 1638 (w), 1601 (w), 1581 (w), 1479 (w), 1458 (w), 1451 (w), 1412 (w), 1381 (w), 1310 (vw), 1244 (vs), 1224 (s), 1183 (m), 1122 (s), 1092 (w), 1048 (vs), 1021 (vs), 999 (s), 875 (s), 827 (vs), 771 (vs), 696 (s), 565 (vs), 544 (s), 523 (s), 470 (m), 439 (m), 414 (m), 394 (w), 377 (m).

MS (EI, 70 eV, 20 °C, %) *m/z* = 200 (95) $[M]^+$, 199 (100) $[M–H]^+$, 185 (9) $[C_8H_{10}O_3P]^+$, 91 (63) $[C_7H_7]^+$.

HRMS (EI, $[M]^+$, $C_9H_{13}O_3P$) calcd.: 200.0597; found: 200.0596.

Additional information on the chemical synthesis is available *via* the Chemotion repository:

https://dx.doi.org/10.14272/reaction/SA-FUHFF-UHFFFADPSC-BOVXCNOYJD-UHFFFADPSC-NUHFF-NUHFF-NUHFF-ZZZ

Additional information on the analysis of the target compound is available *via* the Chemotion repository:

https://dx.doi.org/10.14272/BOVXCNOYJDLWHV-UHFFFAOYSA-N.1

Dimethyl *p*-tolylphosphonate (7b-*p*)

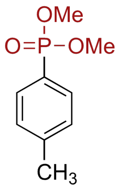

According to the general procedure A, 4-bromotoluene (171 mg, 123 µL) was irradiated for 12 h. The title compound (185 mg, 924 µmol, 92%) was obtained as a colorless oil.

R_f = 0.35 (*n*-pentane/EtOAc, 2:1 + 10% CH_3OH)

^{1}H NMR (500 MHz, $CDCl_3$, ppm) δ = 7.71 – 7.40 (m, 2H, H^{Ar}), 7.30 – 7.25 (m, 2H, H^{Ar}), 3.72 (d, J = 11.1 Hz, 6H, POCH_3), 2.38 (d, J = 1.1 Hz, 3H, CH_3).

^{13}C NMR (126 MHz, $CDCl_3$, ppm) δ = 143.4 (d, J = 3.1 Hz, C_q, C_{Ar}), 132.1 (+, d, J = 10.4 Hz, 2C, C_H, C_{Ar}), 129.4 (+, d, J = 15.5 Hz, 2C, C_H, C_{Ar}), 123.6 (d, J = 190.8 Hz, C_q, C_{Ar}), 52.7 (+, d, J = 5.5 Hz, 2C, CH_3, POCH_3), 21.8 (+, CH_3, CH_3).

^{31}P NMR (202 MHz, $CDCl_3$, ppm) δ = 22.39 (hept, J = 11.7 Hz).

IR (ATR, cm^{-1}) $\tilde{\nu}$ = 3506 (vw), 3489 (w), 3462 (w), 3449 (w), 3434 (w), 2995 (vw), 2989 (vw), 2982 (vw), 2952 (w), 2924 (vw), 2850 (vw), 1655 (w), 1647 (w), 1639 (w), 1606 (w), 1504 (w), 1449 (w), 1402 (w), 1381 (w), 1309 (w), 1237 (s), 1214 (m), 1181 (m), 1129 (s), 1050 (vs), 1017 (vs), 807 (vs), 766 (vs), 713 (m), 687 (m), 649 (vs), 609 (m), 592 (m), 581 (m), 569 (m), 561 (m), 533 (vs), 517 (vs), 465 (s), 453 (s), 433 (s), 426 (s), 408 (m), 398 (m), 384 (m).

MS (EI, 70 eV, 20 °C, %) *m/z* = 200 (100) $[M]^+$, 199 (81) $[M–H]^+$, 185 (3) $[C_8H_{10}O_3P]^+$, 91 (55) $[C_7H_7]^+$.

HRMS (EI, $[M]^+$, $C_9H_{13}O_3P$) calcd.: 200.0597; found: 200.0597.

Additional information on the chemical synthesis is available *via* the Chemotion repository: https://dx.doi.org/10.14272/reaction/SA-FUHFF-UHFFFADPSC-OJXAATCZRH-UHFFFADPSC-NUHFF-NUHFF-NUHFF-ZZZ

Additional information on the analysis of the target compound is available *via* the Chemotion repository:

https://dx.doi.org/10.14272/OJXAATCZRHUZDB-UHFFFAOYSA-N.1

Dimethyl (2,5-dimethylphenyl)phosphonate (7c)

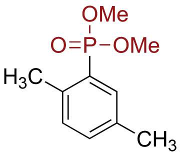

According to the general procedure A, 2-bromo-*p*-xylene (185 mg, 138 µL) was irradiated for 18 h. The title compound (172 mg, 803 µmol, 80%) was obtained as a colorless oil.

R_f = 0.38 (*n*-pentane/EtOAc, 2:1 + 10% CH_3OH)

^{1}H NMR (500 MHz, $CDCl_3$, ppm) δ = 7.72 (dd, J = 14.7, 2.0 Hz, 1H, H^{Ar}), 7.24 (ddt, J = 7.7, 2.0, 0.9 Hz, 1H, H^{Ar}), 7.14 (dd, J = 7.8, 5.9 Hz, 1H, H^{Ar}), 3.74 (d, J = 11.2 Hz, 6H, POCH_3), 2.49 (d, J = 1.7 Hz, 3H, CH_3), 2.33 (d, J = 0.8 Hz, 3H, CH_3).

^{13}C NMR (126 MHz, $CDCl_3$, ppm) δ = 138.8 (d, J = 10.0 Hz, C_q, C^{Ar}), 135.2 (d, J = 14.8 Hz, C_q, C^{Ar}), 134.8 (+, d, J = 10.1 Hz, CH, C^{Ar}), 133.5 (+, d, J = 2.8 Hz, CH, C^{Ar}), 131.4 (+, d, J = 16.3 Hz, CH, C^{Ar}), 125.2 (d, J = 183.4 Hz, C_q, C^{Ar}), 52.5 (+, d, J = 5.5 Hz, 2C, CH_3, POCH_3), 20.9 (+, CH_3, CH_3) ,20.7 (+, d, J = 3.6 Hz, CH_3, CH_3).

^{31}P NMR (202 MHz, $CDCl_3$, ppm) δ = 23.03 (p, J = 13.7, 13.3 Hz).

IR (ATR, cm^{-1}) $\tilde{\nu}$ = 3468 (vw), 2952 (w), 2927 (w), 2850 (w), 1639 (vw), 1564 (w), 1489 (w), 1451 (w), 1388 (w), 1286 (w), 1242 (vs), 1224 (s), 1207 (m), 1181 (m), 1152 (w), 1122 (w), 1086 (s), 1020 (vs), 894 (m), 820 (vs), 776 (s), 737 (m), 713 (w), 693 (m), 667 (w), 647 (w), 574 (vs), 554 (s), 487 (m), 472 (m), 462 (s), 436 (w), 411 (w), 394 (m).

MS (EI, 70 eV, 20 °C, %) *m/z* = 214 (97) $[M]^+$, 213 (100) $[M–H]^+$, 199 (41) $[M–CH_3]^+$, 183 (10) $[M–CH_3O]^+$, 105 (24) $[M–C_2H_6O_3P]^+$.

HRMS (EI, $[M]^+$, $C_{10}H_{15}O_3P$) calcd.: 214.0753; found: 214.0753.

Additional information on the chemical synthesis is available *via* the Chemotion repository:

https://dx.doi.org/10.14272/reaction/SA-FUHFF-UHFFFADPSC-PCUATYAZCD-UHFFFADPSC-NUHFF-NUHFF-NUHFF-ZZZ

Additional information on the analysis of the target compound is available *via* the Chemotion repository:

https://dx.doi.org/10.14272/PCUATYAZCDNKBF-UHFFFAOYSA-N.1

Dimethyl (2,4,6-trimethylphenyl)phosphonate (7d)

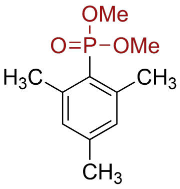

According to the general procedure A, 2-bromomesitylene (199 mg, 153 µL) was irradiated for 18 h. The title compound (177 mg, 776 µmol, 78%) was obtained as a colorless oil.

R_f = 0.46 (*n*-pentane/EtOAc, 2:1 + 10% CH_3OH)

^{1}H NMR (500 MHz, $CDCl_3$, ppm) δ = 6.91 (d, J = 4.7 Hz, 2H, H^{Ar}), 3.71 (d, J = 11.3 Hz, 6H, POCH_3), 2.57 (d, J = 1.8 Hz, 6H, o-CH_3), 2.28 (d, J = 1.0 Hz, 3H, p-CH_3).

^{13}C NMR (126 MHz, $CDCl_3$, ppm) δ = 144.1 (d, J = 11.9 Hz, 2C, C_q, C^{Ar}), 142.3 (d, J = 2.8 Hz, C_q, C^{Ar}), 130.5 (+, d, J = 16.3 Hz, 2C, CH, C^{Ar}), 121.0 (d, J = 182.1 Hz, C_q, C^{Ar}), 51.8 (+, d, J = 5.4 Hz, 2C, CH_3, POCH_3), 23.2 (+, d, J = 2.8 Hz, 2C, CH_3, o-CH_3), 21.2 (+, CH_3, p-CH_3).

^{31}P NMR (202 MHz, $CDCl_3$, ppm) δ = 23.56 (p, J = 11.6, 11.0 Hz).

IR (ATR, cm^{-1}) $\tilde{\nu}$ = 3468 (vw), 2951 (w), 2847 (vw), 1656 (vw), 1639 (vw), 1606 (w), 1557 (w), 1510 (w), 1449 (w), 1411 (w), 1381 (w), 1289 (w), 1244 (s), 1232 (s), 1184 (m), 1122 (w), 1085 (s), 1048 (vs), 1017 (vs), 929 (w), 851 (w), 822 (vs), 775 (vs), 721 (m), 697 (w), 645 (vs), 561 (s), 548 (m), 524 (vs), 486 (s), 453 (m), 442 (m), 432 (m), 411 (m), 387 (m), 380 (m).

MS (EI, 70 eV, 20 °C, %) m/z = 228 (77) $[M]^+$, 213 (100) $[M–CH_3]^+$, 197 (6) $[M–CH_3O]^+$, 119 (15) $[M–C_2H_6O_3P]^+$.

HRMS (EI, $[M]^+$, $C_{11}H_{17}O_3P$) calcd.: 228.0910; found: 228.0908.

Additional information on the chemical synthesis is available *via* the Chemotion repository:

https://dx.doi.org/10.14272/reaction/SA-FUHFF-UHFFFADPSC-SJCMNWBMJL-UHFFFADPSC-NUHFF-NUHFF-NUHFF-ZZZ

Additional information on the analysis of the target compound is available *via* the Chemotion repository:

https://dx.doi.org/10.14272/SJCMNWBMJLIBAW-UHFFFAOYSA-N.1

Tetramethyl 1,2-phenylenebis(phosphonate) (7e'-*o*)

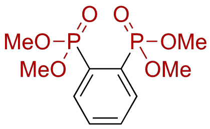

According to the general procedure A, 1-bromo-2-chlorobenzene (192 mg, 117 µL) was irradiated for 18 h. The title compound (184 mg, 626 µmol, 63%) was obtained as a colorless solid.

R_f = 0.11 (n-pentane/EtOAc, 2:1 + 10% CH_3OH)

^1H NMR (500 MHz, $CDCl_3$, ppm) δ = 8.19 – 8.09 (m, 2H, H^{Ar}), 7.69 – 7.60 (m, 2H, H^{Ar}), 3.82 (d, J = 11.2 Hz, 12H, POCH_3).

^{13}C NMR (126 MHz, $CDCl_3$, ppm) δ = 135.7 (+, t, J = 11.5 Hz, 2C, CH, C^{Ar}), 132.3 – 131.8 (+, m, 2C, CH, C^{Ar}), 130.6 (dd, J = 190.2, 10.3 Hz, 2C, C_q, C^{Ar}), 53.2 (+, t, J = 3.1 Hz, 4C, CH_3, POCH_3).

^{31}P NMR (202 MHz, $CDCl_3$, ppm) δ = 19.14 – 17.94 (m).

IR (ATR, cm^{-1}) $\tilde{\nu}$ = 3478 (vw), 3085 (vw), 3006 (w), 2955 (w), 2918 (vw), 2900 (vw), 2846 (vw), 1909 (vw), 1861 (vw), 1823 (vw), 1715 (vw), 1578 (vw), 1562 (vw), 1460 (w), 1438 (w), 1419 (w), 1232 (vs), 1177 (s), 1129 (s), 1021 (vs), 1016 (vs), 999 (vs), 832 (vs), 789 (vs), 731 (vs), 659 (m), 577 (vs), 544 (vs), 511 (vs), 465 (s), 442 (m), 414 (m).

MS (EI, 70 eV, 60 °C, %) m/z = 294 (2) $[M]^+$, 263 (100) $[M–CH_3O]^+$, 185 (11) $[M–C_2H_6O_3P]^+$.

HRMS (EI, $[M]^+$, $C_{10}H_{16}O_6P_2$) calcd.: 294.0417; found: 294.0415.

Additional information on the chemical synthesis is available *via* the Chemotion repository:

https://dx.doi.org/10.14272/reaction/SA-FUHFF-UHFFFADPSC-TUKTVDDATW-UHFFFADPSC-NUHFF-NUHFF-NUHFF-ZZZ

Additional information on the analysis of the target compound is available *via* the Chemotion repository:

https://dx.doi.org/10.14272/TUKTVDDATWNXSN-UHFFFAOYSA-N.1

Dimethyl (3-chlorophenyl)phosphonate (7e-*m*)

According to the general procedure A, 1-bromo-3-chlorobenzene (192 mg, 117 µL) was irradiated for 18 h. The title compound (79.0 mg, 358 µmol, 36%) and tetramethyl 1,3-phenylenebis(phosphonate) (113 mg, 384 µmol, 38%) was obtained as a colorless oil and colorless solid, respectively.

Dimethyl (3-chlorophenyl)phosphonate (**7e-*m***)

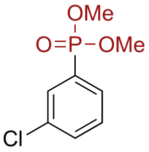

R_f = 0.37 (*n*-pentane/EtOAc, 2:1 + 10% CH_3OH)

^{1}H NMR (500 MHz, $CDCl_3$, ppm) δ = 7.76 (ddd, *J* = 13.8, 3.6, 0.5 Hz, 1H, H^{Ar}), 7.71 – 7.63 (m, 1H, H^{Ar}), 7.53 (dd, *J* = 8.1, 4.3 Hz, 1H, H^{Ar}), 7.41 (dddd, *J* = 8.0, 7.5, 4.8, 0.5 Hz, 1H, H^{Ar}), 3.76 (d, *J* = 11.1 Hz, 6H, POCH_3).

^{13}C NMR (126 MHz, $CDCl_3$, ppm) δ = 135.1 (d, *J* = 20.4 Hz, C_q, C^{Ar}), 132.9 (+, d, *J* = 3.1 Hz, CH, C^{Ar}), 131.9 (+, d, *J* = 10.7 Hz, CH, C^{Ar}), 130.3–129.9 (+, m, 2C, CH, C^{Ar}), 129.4 (d, *J* = 188.8 Hz, C_q, C^{Ar}), 53.0 (+, d, *J* = 5.6 Hz, 2C, CH_3, POCH_3).

^{31}P NMR (202 MHz, $CDCl_3$, ppm) δ = 19.68 – 19.01 (m).

IR (ATR, cm^{-1}) $\tilde{\nu}$ = 3486 (w), 3478 (w), 3461 (w), 3444 (w), 3422 (vw), 2953 (w), 2851 (vw), 1640 (w), 1587 (w), 1565 (w), 1534 (vw), 1517 (vw), 1469 (w), 1402 (w), 1300 (vw), 1244 (vs), 1183 (m), 1143 (s), 1092 (w), 1078 (m), 1048 (vs), 1021 (vs), 997 (s), 919 (w), 894 (w), 819 (vs), 790 (s), 748 (s), 687 (s), 667 (s), 629 (w), 620 (w), 561 (vs), 530 (s), 493 (m), 472 (m), 459 (m), 445 (m), 409 (m), 378 (w).

MS (EI, 70 eV, 20 °C, %) *m/z* = 222/220 (26/87) $[M]^+$, 221/119 (37/100) $[M–H]^+$, 114/112 (9/27) $[C_6H_5Cl]^+$, 109 (7) $[C_2H_6O_3P]^+$, 79 (42) $[C_6H_7]^+$.

HRMS (EI, $[M]^+$, $C_8H_{10}O_3{}^{35}ClP$) calcd.: 220.0051; found: 220.0052.

Tetramethyl 1,3-phenylenebis(phosphonate) (**7e'-*m***)

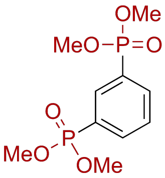

R_f = 0.13 (*n*-pentane/EtOAc, 2:1 + 10% CH_3OH)

^{1}H NMR (500 MHz, $CDCl_3$, ppm) δ = 8.21 – 8.11 (m, 1H, H^{Ar}), 8.00 – 7.91 (m, 2H, H^{Ar}), 7.61 – 7.51 (m, 1H, H^{Ar}), 3.73 (d, *J* = 11.1 Hz, 12H, POCH_3).

^{13}C NMR (126 MHz, $CDCl_3$, ppm) δ =136.0 – 135.6 (+, m, 2C, CH, C^{Ar}), 135.1 (+, t, *J* = 10.6 Hz, CH, C^{Ar}), 129.0 – 128.6 (+, m, CH, C^{Ar}), 128.1 (dd, *J* = 170.1, 13.8 Hz, 2C, C_q, C^{Ar}), 53.1 – 52.8 (+, m, 4C, CH_3, POCH_3).

^{31}P NMR (202 MHz, $CDCl_3$, ppm) δ = 19.74 – 19.22 (m).

IR (ATR, cm^{-1}) $\tilde{\nu}$ = 3478 (w), 3472 (w), 3463 (w), 3456 (w), 3442 (w), 2955 (w), 2853 (vw), 1655 (w), 1647 (w), 1642 (w), 1591 (w), 1460 (w), 1395 (w), 1242 (vs), 1181 (m), 1169 (m), 1109 (m), 1088 (w), 1017 (vs), 832 (vs), 800 (s), 782 (s), 741 (m), 697 (s), 674 (m), 653 (w), 551 (vs), 528 (s), 490 (s), 455 (m), 452 (m), 429 (m), 418 (m), 384 (w).

MS (EI, 70 eV, 50 °C, %) *m/z* = 294 (42) $[M]^+$, 293 (46) $[M–H]^+$, 200 (100) $[C_7H_7O_3P_2]^+$, 186 (57) $[M–C_2H_5O_3P]^+$, 110 (48) $[C_2H_7O_3P]^+$.

HRMS (EI, $[M]^+$, $C_{10}H_{16}O_6P_2$) calcd.: 294.0417; found: 294.0418.

Additional information on the chemical synthesis is available *via* the Chemotion repository:

https://dx.doi.org/10.14272/reaction/SA-FUHFF-UHFFFADPSC-FPRHXSAIDB-UHFFFADPSC-NUHFF-NUHFF-NUHFF-ZZZ

Additional information on the analysis of the target compound is available *via* the Chemotion repository:

https://dx.doi.org/10.14272/QHKAIMLZIPDGSR-UHFFFAOYSA-N.1

https://dx.doi.org/10.14272/VFQQKPYJNBHBDK-UHFFFAOYSA-N.1

Dimethyl (4-chlorophenyl)phosphonate (7e-*p*)

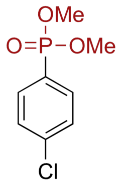

According to the general procedure A, 1-bromo-4-chlorobenzene (192 mg) was irradiated for 18 h. The title compound (27.0 mg, 122 µmol, 12%) and tetramethyl 1,4-phenylenebis(phosphonate) (140 mg, 476 µmol, 38%) was obtained as a colorless oil and colorless solid, respectively.

R_f = 0.41 (*n*-pentane/EtOAc, 2:1 + 10% CH_3OH)

^1H NMR (500 MHz, $CDCl_3$, ppm) δ =7.77 – 7.66 (m, 2H, H^{Ar}), 7.49 – 7.40 (m, 2H, H^{Ar}), 3.74 (d, *J* = 11.1 Hz, 6H, $POCH_3$).

^{13}C NMR (126 MHz, $CDCl_3$, ppm) δ = 139.3 (d, *J* = 4.3 Hz, C_q, C^{Ar}), 133.4 (+, d, *J* = 10.8 Hz, 2C, CH, C^{Ar}), 129.0 (+, d, *J* = 16.1 Hz, 2C, CH, C^{Ar}), 125.4 (d, *J* = 192.0 Hz, C_q, C^{Ar}), 52.89 (+, d, *J* = 5.5 Hz, 2C, CH_3, $POCH_3$).

^{31}P NMR (202 MHz, $CDCl_3$, ppm) δ = 20.52 (dp, *J* = 23.6, 11.1 Hz).

IR (ATR, cm^{-1}) $\tilde{\nu}$ = 3509 (vw), 3486 (vw), 3463 (vw), 3455 (vw), 3435 (vw), 3408 (vw), 2952 (w), 2918 (w), 2850 (w), 1737 (vw), 1721 (vw), 1655 (vw), 1647 (vw), 1584 (m), 1564 (w), 1536 (vw), 1528 (vw), 1485 (w), 1462 (w), 1392 (w), 1366 (w), 1324 (vw), 1244 (vs), 1181 (m), 1130 (s), 1089 (s), 1051 (vs), 1023 (vs), 1013 (vs), 817 (vs), 788 (vs), 738 (s), 711 (w), 674 (w), 670 (w), 660 (w), 646 (w), 628 (w), 596 (s), 574 (m), 521 (s), 466 (s), 429 (m).

MS (EI, 70 eV, 20 °C, %) *m/z* = 220 (100) $[M]^+$, 219 (69) $[M–H]^+$, 111 (23) $[C_2H_8O_3P]^+$, 79 (29) $[C_6H_7]^+$.

HRMS (EI, $[M]^+$, $C_8H_{10}O_3{}^{35}ClP$) calcd.: 220.0051; found: 220.0050.

Tetramethyl 1,4-phenylenebis(phosphonate) (**7e'/f'/g'-*p***)

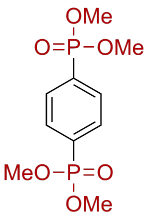

R_f = 0.10 (*n*-pentane/EtOAc, 2:1 + 10% CH_3OH)

^{1}H NMR (500 MHz, $CDCl_3$, ppm) *δ* = 8.01 – 7.81 (m, 4H, H^{Ar}), 3.77 (dd, *J* = 11.1, 1.5 Hz, 12H, POCH_3).

^{13}C NMR (126 MHz, $CDCl_3$, ppm) *δ* = 132.19 – 131.73 (+, m, 4C, CH, C^{Ar}), 131.86 (dd, *J* = 187.7, 3.2 Hz, 2C, C_q, C^{Ar}), 53.14 – 52.98 (+, m, 4C, CH_3, POCH_3).

^{31}P NMR (202 MHz, $CDCl_3$, ppm) *δ* = 20.48 – 18.59.

IR (ATR, cm^{-1}) $\tilde{\nu}$ = 3 3441 (vw), 2999 (vw), 2955 (w), 2853 (vw), 1458 (w), 1385 (w), 1367 (w), 1276 (w), 1242 (vs), 1179 (s), 1137 (s), 1096 (w), 1043 (vs), 1010 (vs), 854 (m), 817 (vs), 771 (vs), 649 (w), 598 (vs), 547 (vs), 459 (m), 414 (vs), 382 (vs).

MS (EI, 70 eV, 60 °C, %) *m/z* = 294 (59) $[M]^+$, 293 (56) $[M–H]^+$, 200 (100) $[C_7H_7O_3P_2]^+$, 186 (38) $[M–C_2H_5O_3P]^+$, 110 (15) $[C_2H_7O_3P]^+$.

HRMS (EI, $[M]^+$, $C_{10}H_{16}O_6P_2$) calcd.: 294.0417; found: 294.0418.

Additional information on the chemical synthesis is available *via* the Chemotion repository:

https://dx.doi.org/10.14272/reaction/SA-FUHFF-UHFFFADPSC-XGYPEDJHXF-UHFFFADPSC-NUHFF-NUHFF-NUHFF-ZZZ

Additional information on the analysis of the target compound is available *via* the Chemotion repository:

https://dx.doi.org/10.14272/UTGYZBPCVJGRBV-UHFFFAOYSA-N.1

https://dx.doi.org/10.14272/YJVXDIBSKYAUDH-UHFFFAOYSA-N.2

Dimethyl (2-fluorophenyl)phosphonate (7f-*o*)

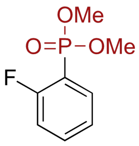

According to the general procedure A, 1-bromo-2-fluorobenzene (175 mg, 109 µL) was irradiated for 18 h. The title compound (122 mg, 598 µmol, 60%) was obtained as a colorless oil.

R_f = 0.33 (*n*-pentane/EtOAc, 2:1 + 10% CH_3OH)

^{1}H NMR (500 MHz, $CDCl_3$, ppm) *δ* = 7.91 – 7.80 (m, 1H, H^{Ar}), 7.61 – 7.53 (m, 1H, H^{Ar}), 7.27 – 7.22 (m, 1H, H^{Ar}), 7.17 – 7.10 (m, 1H, H^{Ar}), 3.81 (d, *J* = 11.4 Hz, 6H, POCH_3).

^{13}C NMR (126 MHz, $CDCl_3$, ppm) *δ* = 163.4 (d, *J* = 253.3 Hz, C_q, C^{Ar}), 135.8 – 134.4 (+, m, 2C, CH, C^{Ar}), 124.36 (+, dd, *J* = 13.7, 3.6 Hz, CH, C^{Ar}), 116.3 (+, dd, *J* = 22.7, 8.2 Hz, CH, C^{Ar}), 115.1 (dd, *J* = 189.0, 18.2 Hz, C_q, C^{Ar}), 53.1 (+, d, *J* = 5.5 Hz, 2C, CH_3, POCH_3).

^{19}F NMR (471 MHz, $CDCl_3$, ppm) *δ* = -103.68 – -103.85 (m).

^{31}P NMR (202 MHz, $CDCl_3$, ppm) *δ* = 16.85 (dq, *J* = 19.0, 9.6 Hz).

IR (ATR, cm^{-1}) $\tilde{\nu}$ = 3480 (vw), 3471 (vw), 3456 (vw), 3449 (vw), 3435 (vw), 3425 (vw), 2956 (w), 2853 (w), 1819 (vw), 1735 (vw), 1725 (vw), 1693 (vw), 1686 (vw), 1640 (w), 1635 (w), 1606 (m), 1578 (w), 1534 (vw), 1524 (vw), 1476 (s), 1445 (s), 1245 (vs), 1224 (s), 1183 (m), 1162 (m), 1136 (s), 1091 (s), 1023 (vs), 914 (w), 904 (w), 870 (w), 829 (vs), 766 (vs), 721 (w), 683 (m), 636 (w), 626 (w), 620 (w), 572 (s), 537 (s), 496 (s), 482 (s), 455 (m), 442 (m), 416 (m), 407 (m), 398 (m), 377 (w).

MS (EI, 70 eV, 20 °C, %) *m/z* = 204 (100) $[M]^+$, 203 (51) $[M–H]^+$, 185 (28) $[M–F]^+$, 173 (8) $[M–CH_3O]^+$, 109 (87) $[C_2H_6O_3P]^+$, 95 (28) $[C_6H_4F]^+$.

HRMS (EI, $[M]^+$, $C_8H_{10}O_3FP$) calcd.: 204.0346; found: 204.0346.

Additional information on the chemical synthesis is available *via* the Chemotion repository:

https://dx.doi.org/10.14272/reaction/SA-FUHFF-UHFFFADPSC-ZOZAJBLUHH-UHFFFADPSC-NUHFF-NUHFF-NUHFF-ZZZ

Additional information on the analysis of the target compound is available *via* the Chemotion repository:

https://dx.doi.org/10.14272/ZOZAJBLUHHYJBL-UHFFFAOYSA-N.1

Dimethyl (3-fluorophenyl)phosphonate (7f-*m*)

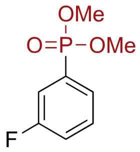

According to the general procedure A, 1-bromo-3-fluorobenzene (175 mg, 111 µL) was irradiated for 18 h. The title compound (104 mg, 510 µmol, 51%) was obtained as a colorless oil.

R_f = 0.37 (*n*-pentane/EtOAc, 2:1 + 10% CH_3OH)

^{1}H NMR (500 MHz, $CDCl_3$, ppm) δ = 7.57 (ddt, *J* = 12.9, 7.5, 1.2 Hz, 1H, H^{Ar}), 7.51 – 7.41 (m, 2H, H^{Ar}), 7.29 – 7.20 (m, 1H, H^{Ar}), 3.76 (d, *J* = 11.1 Hz, 6H, $POCH_3$).

^{13}C NMR (126 MHz, $CDCl_3$, ppm) δ = 162.5 (dd, *J* = 249.7, 21.7 Hz, C_q, C^{Ar}), 130.6 (+, dd, *J* = 17.9, 7.4 Hz, CH, C^{Ar}), 129.6 (dd, *J* = 189.7, 6.3 Hz, C_q, C^{Ar}), 127.7 (+, dd, *J* = 9.1, 3.5 Hz, CH, C^{Ar}), 119.9 (+, dd, *J* = 21.0, 2.9 Hz, CH, C^{Ar}), 118.8 (+, dd, *J* = 22.4, 10.4 Hz, CH, C^{Ar}), 52.9 (+, d, *J* = 5.6 Hz, 2C, CH_3, $POCH_3$).

^{19}F NMR (471 MHz, $CDCl_3$, ppm) δ = -106.39 – -116.06 (m).

^{31}P NMR (202 MHz, $CDCl_3$, ppm) δ = 19.47 (q, *J* = 15.4 Hz).

IR (ATR, cm^{-1}) $\tilde{\nu}$ = 3519 (vw), 3486 (vw), 3472 (vw), 3456 (vw), 3381 (vw), 2956 (vw), 1608 (vw), 1584 (w), 1480 (w), 1462 (w), 1421 (w), 1245 (s), 1224 (vs), 1183 (m), 1108 (m), 1018 (vs), 1000 (vs), 898 (m), 830 (vs), 790 (s), 768 (vs), 686 (vs), 630 (w), 619 (w), 558 (vs), 533 (s), 520 (s), 479 (s), 456 (s), 399 (s).

MS (EI, 70 eV, 20 °C, %) *m/z* = 204 (100) $[M]^+$, 203 (90) $[M–H]^+$, 185 (3) $[M–F]^+$, 173 (30) $[M–CH_3O]^+$, 109 (70) $[C_2H_6O_3P]^+$, 95 (38) $[C_6H_4F]^+$.

HRMS (EI, $[M]^+$, $C_8H_{10}O_3FP$) calcd.: 204.0346; found: 204.0348.

Additional information on the chemical synthesis is available *via* the Chemotion repository:

https://dx.doi.org/10.14272/reaction/SA-FUHFF-UHFFFADPSC-QEFKCPWDVH-UHFFFADPSC-NUHFF-NUHFF-NUHFF-ZZZ

Additional information on the analysis of the target compound is available *via* the Chemotion repository:

https://dx.doi.org/10.14272/QEFKCPWDVHVOQC-UHFFFAOYSA-N.1

Dimethyl (4-fluorophenyl)phosphonate (7f-*p*)

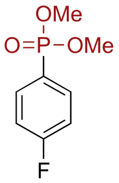

According to the general procedure A, 1-bromo-4-fluorobenzene (175 mg, 110 µL) was irradiated for 18 h. The title compound (125 mg, 612 µmol, 61%) and tetramethyl 1,4-phenylenebis (phosphonate) (110 mg, 374 µmol, 37%) was obtained as a colorless oil and colorless solid, respectively.

R_f = 0.35 (*n*-pentane/EtOAc, 2:1 + 10% CH_3OH)

^{1}H NMR (500 MHz, $CDCl_3$, ppm) δ = 7.86 – 7.75 (m, 2H, H^{Ar}), 7.20 – 7.10 (m, 2H, H^{Ar}), 3.75 (dd, J = 11.1, 1.4 Hz, 6H, H^{Ar}).

^{13}C NMR (126 MHz, $CDCl_3$, ppm) δ = 165.6 (dd, J = 253.9, 4.0 Hz, C_q, C^{Ar}), 135.2 – 133.9 (+, m, 2C, CH, C^{Ar}), 123.1 (dd, J = 193.5, 3.6 Hz, C_q, C^{Ar}), 116.0 (+, dd, J = 21.7, 16.3 Hz, 2C, CH, C^{Ar}), 52.8 (d, J = 5.4 Hz, 2C, CH_3, PO$C$$H_3$).

^{19}F NMR (471 MHz, $CDCl_3$, ppm) δ = -105.35 – -105.57 (m).

^{31}P NMR (202 MHz, $CDCl_3$, ppm) δ = 20.61 (dq, J = 23.4, 12.0 Hz).

IR (ATR, cm^{-1}) $\tilde{\nu}$ = 3479 (vw), 3471 (vw), 2955 (w), 2851 (vw), 1592 (m), 1503 (m), 1462 (w), 1401 (w), 1227 (vs), 1184 (m), 1162 (m), 1129 (vs), 1095 (w), 1050 (vs), 1016 (vs), 892 (vw), 882 (vw), 819 (vs), 773 (vs), 714 (w), 703 (w), 688 (w), 653 (m), 632 (w), 598 (w), 589 (w), 572 (w), 527 (vs), 506 (vs), 480 (s), 459 (m), 441 (s), 409 (m), 402 (m), 380 (w).

MS (EI, 70 eV, 20 °C, %) *m/z* = 204 (100) $[M]^+$, 203 (91) $[M–H]^+$, 185 (1) $[M–F]^+$, 173 (37) $[M–CH_3O]^+$, 109 (95) $[C_2H_6O_3P]^+$, 95 (36) $[C_6H_4F]^+$.

HRMS (EI, $[M]^+$, $C_8H_{10}O_3FP$) calcd.: 204.0346; found: 204.0348.

Additional information on the chemical synthesis is available *via* the Chemotion repository:

https://dx.doi.org/10.14272/reaction/SA-FUHFF-UHFFFADPSC-FWEAYEWDUH-UHFFFADPSC-NUHFF-NUHFF-NUHFF-ZZZ

Additional information on the analysis of the target compound is available *via* the Chemotion repository:

https://dx.doi.org/10.14272/VGJOAISDEPXNNN-UHFFFAOYSA-N.1

https://dx.doi.org/10.14272/YJVXDIBSKYAUDH-UHFFFAOYSA-N.1

Dimethyl (2-methoxyphenyl)phosphonate (4g-*o*)

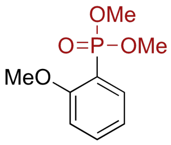

According to the general procedure A, 2-bromoansiole (187 mg, 125 µL) was irradiated for 18 h. The title compound (130 mg, 601 µmol, 60%) was obtained as a colorless oil.

R_f = 0.17 (*n*-pentane/EtOAc, 2:1 + 5% CH_3OH)

^1H NMR (500 MHz, $CDCl_3$, ppm) δ = 7.77 (ddd, J = 14.9, 7.6, 1.8 Hz, 1H, H^{Ar}), 7.49 (dddd, J = 8.4, 7.5, 1.8, 0.9 Hz, 1H, H^{Ar}), 6.98 (tdd, J = 7.5, 3.5, 0.9 Hz, 1H, H^{Ar}), 6.92 (ddd, J = 8.1, 6.8, 0.9 Hz, 1H, H^{Ar}), 3.87 (s, 3H, OCH_3), 3.76 (d, J = 11.3 Hz, 6H, $POCH_3$).

^{13}C NMR (126 MHz, $CDCl_3$, ppm) δ = 161.4 (d, J = 2.7 Hz, C_q, C^{Ar}), 135.2 (+, d, J = 6.6 Hz, CH, C^{Ar}), 134.6 (+, d, J = 2.3 Hz, CH, C^{Ar}), 120.5 (+, d, J = 14.5 Hz, CH, C^{Ar}), 115.3 (d, J = 188.9 Hz, C_q, C^{Ar}), 111.3 (+, d, J = 9.1 Hz, CH, C^{Ar}), 56.0 (+, CH_3, OCH_3), 52.90 (+, d, J = 5.5 Hz, 2C, CH_3, $POCH_3$).

^{31}P NMR (202 MHz, $CDCl_3$, ppm) δ = 20.84 – 19.97 (m).

IR (ATR, cm^{-1}) $\tilde{\nu}$ = 3461 (vw), 2953 (w), 2919 (w), 2849 (w), 1655 (vw), 1592 (m), 1577 (w), 1480 (s), 1463 (m), 1434 (m), 1377 (w), 1278 (s), 1249 (vs), 1181 (s), 1167 (m), 1143 (m), 1091 (m), 1021 (vs), 980 (vs), 827 (vs), 809 (vs), 764 (vs), 734 (m), 688 (m), 656 (w), 649 (w), 640 (w), 620 (w), 584 (s), 554 (s), 517 (s), 510 (s), 497 (s), 459 (m), 414 (m), 397 (m), 380 (w).

MS (EI, 70 eV, 30 °C, %) m/z = 216 (100) $[M]^+$, 185 (27) $[M–CH_3O]^+$, 155 (20) $[M–C_2H_5O_2]^+$, 110 (56) $[C_2H_7O_3P]^+$, 79 (21) $[C_6H_7]^+$.

HRMS (EI, $[M]^+$, $C_9H_{13}O_4P$) calcd.: 216.0546; found: 216.0547.

Additional information on the chemical synthesis is available *via* the Chemotion repository:

https://dx.doi.org/10.14272/reaction/SA-FUHFF-UHFFFADPSC-WWXKFQJWHM-UHFFFADPSC-NUHFF-NUHFF-NUHFF-ZZZ

Additional information on the analysis of the target compound is available *via* the Chemotion repository:

https://dx.doi.org/10.14272/WWXKFQJWHMHAIR-UHFFFAOYSA-N.1

Dimethyl (3-methoxyphenyl)phosphonate (7g-*o*)

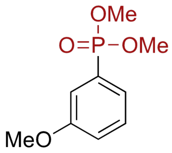

According to the general procedure A, 3-bromoanisole (187 mg, 127 µL) was irradiated for 18 h. The title compound (147 mg, 680 µmol, 68%) was obtained as a colorless oil.

R_f = 0.33 (*n*-pentane/EtOAc, 2:1 + 10% CH_3OH)

^1H NMR (500 MHz, $CDCl_3$, ppm) δ = 7.42 – 7.34 (m, 2H, H^{Ar}), 7.34 – 7.29 (m, 1H, H^{Ar}), 7.11 – 7.07 (m, 1H, H^{Ar}), 3.84 (s, 3H, OCH_3), 3.76 (d, J = 11.1 Hz, 6H, $POCH_3$).

^{13}C NMR (126 MHz, $CDCl_3$, ppm) δ = 159.6 (d, J = 19.0 Hz, C_q, C^{Ar}), 130.0 (+, d, J = 18.0 Hz, CH, C^{Ar}), 128.2 (d, J = 187.5 Hz, C_q, C^{Ar}), 124.2 (+, d, J = 9.3 Hz, CH, C^{Ar}), 119.2 (+, d, J = 3.3 Hz, CH, C^{Ar}), 116.5 (+, d, J = 11.5 Hz, CH, C^{Ar}), 55.5 (+, CH_3, OCH_3), 52.8 (+, d, J = 5.6 Hz, 2C, CH_3, POCH_3).

^{31}P NMR (202 MHz, $CDCl_3$, ppm) δ = 21.93 – 21.16 (m).

IR (ATR, cm^{-1}) $\tilde{\nu}$ = 3519 (vw), 3468 (w), 3452 (w), 3425 (vw), 3004 (vw), 2953 (w), 2850 (vw), 1643 (vw), 1595 (w), 1578 (w), 1486 (w), 1463 (w), 1422 (w), 1417 (w), 1320 (w), 1288 (w), 1235 (vs), 1183 (m), 1125 (w), 1115 (w), 1017 (vs), 992 (vs), 867 (m), 827 (vs), 765 (vs), 688 (s), 555 (vs), 526 (s), 480 (s), 411 (s).

MS (EI, 70 eV, 20 °C, %) m/z = 216 (100) $[M]^+$, 215 (66) $[M–H]^+$,185 (16) $[M–CH_3O]^+$, 108 (24) $[M–C_2H_6O_3P]^+$.

HRMS (EI, $[M]^+$, $C_9H_{13}O_4P$) calcd.: 216.0546; found: 216.0547.

Additional information on the chemical synthesis is available *via* the Chemotion repository:

https://dx.doi.org/10.14272/reaction/SA-FUHFF-UHFFFADPSC-GDSWYCZRSZ-UHFFFADPSC-NUHFF-NUHFF-NUHFF-ZZZ

Additional information on the analysis of the target compound is available *via* the Chemotion repository:

https://dx.doi.org/10.14272/GDSWYCZRSZXYHX-UHFFFAOYSA-N.1

Dimethyl (4-methoxyphenyl)phosphonate (7g-*p*)

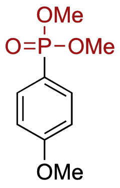

According to the general procedure A, 4-bromoanisole (187 mg, 126 µL) was irradiated for 18 h. The title compound (178, 823 µmol, 82%) was obtained as a colorless oil.

Under the reaction conditions of general procedure B, the title compound (110mg, 509 µmol, 51%) and tetramethyl 1,4-phenylenebis(phosphonate) (141 mg, 479 µmol, 48%) were obtained as a colorless oil and colorless solid, respectively.

R_f = 0.32 (n-pentane/EtOAc, 2:1 + 10% CH_3OH)

^{1}H NMR (500 MHz, $CDCl_3$, ppm) δ = 7.76 – 7.72 (m, 1H, H^{Ar}), 7.72 – 7.69 (m, 1H, H^{Ar}), 6.99 – 6.93 (m, 2H, H^{Ar}), 3.83 (s, 3H, OCH_3), 3.72 (d, J = 11.1 Hz, 6H, POCH_3).

^{13}C NMR (126 MHz, $CDCl_3$, ppm) δ = 163.1 (d, J = 3.2 Hz, C_q, C^{Ar}), 134.0 (+, d, J = 11.0 Hz, 2C, CH, C^{Ar}), 118.08 (d, J = 196.1 Hz, C_q, C^{Ar}), 114.21 (+, d, J = 16.2 Hz, 2C, CH, C^{Ar}), 55.45 (+, CH_3, OCH_3), 52.65 (d, J = 5.4 Hz, 2C, CH_3, POCH_3).

^{31}P NMR (202 MHz, $CDCl_3$, ppm) δ = 22.57 (hept, J = 11.4 Hz).

IR (ATR, cm^{-1}) $\tilde{\nu}$ = 3458 (vw), 2999 (vw), 2953 (w), 2919 (vw), 2905 (vw), 2849 (w), 1599 (s), 1571 (w), 1536 (vw), 1506 (m), 1459 (w), 1446 (w), 1411 (w), 1381 (vw), 1296 (m), 1244 (vs), 1179 (s), 1130 (vs), 1111 (m), 1020 (vs), 812 (vs), 769 (s), 722 (w), 700 (w), 683 (w), 654 (m), 629 (m), 605 (w), 594 (w), 544 (vs), 470 (m), 432 (m), 401 (w), 385 (w).

MS (EI, 70 eV, 20 °C, %) m/z = 216 (100) $[M]^+$, 215 (33) $[M–H]^+$,185 (20) $[M–CH_3O]^+$, 108 (19) $[M–C_2H_6O_3P]^+$.

HRMS (EI, $[M]^+$, $C_9H_{13}O_4P$) calcd.: 216.0546; found: 216.0547.

Additional information on the chemical synthesis is available *via* the Chemotion repository:

https://dx.doi.org/10.14272/reaction/SA-FUHFF-UHFFFADPSC-OPDVSBXFRV-UHFFFADPSC-NUHFF-NUHFF-NUHFF-ZZZ

Additional information on the analysis of the target compound is available *via* the Chemotion repository:

https://dx.doi.org/10.14272/OPDVSBXFRVFBFU-UHFFFAOYSA-N.1

Dimethyl (4-(trifluoromethyl)phenyl)phosphonate (7h)

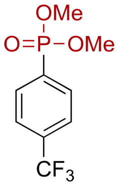

According to the general procedure A, 4-bromobenzotrifluoride (225 mg, 140 µL) was irradiated for 18 h. The title compound (177 mg, 697 µmol, 70%) was obtained as a colorless oil.

R_f = 0.30 (*n*-pentane/EtOAc, 2:1 + 10% CH_3OH)

^{1}H NMR (500 MHz, $CDCl_3$, ppm) δ = 7.96 – 7.89 (m, 2H, H^{Ar}), 7.81 – 7.71 (m, 2H, H^{Ar}), 3.78 (d, J = 11.1 Hz, 6H, POCH_3).

^{13}C NMR (126 MHz, $CDCl_3$, ppm) δ = 135.2 – 133.9 (m, C_q, $C^{Ar}CF_3$), 132.5 (+, d, J = 10.1 Hz, 2C, CH, C^{Ar}), 131.47(d, J = 188.7 Hz, C_q, C^{Ar}), 125.5 (+, dq, J = 15.2, 3.7 Hz, 2C, CH, C^{Ar}), 123.6 (q, J = 272.8 Hz, C_q, CF_3), 53.0 (+, d, J = 5.6 Hz, 2C, CH_3, POCH_3).

^{19}F NMR (471 MHz, $CDCl_3$, ppm) δ = -63.34.

^{31}P NMR (202 MHz, $CDCl_3$, ppm) δ = 19.10 (hept, J = 11.2 Hz).

IR (ATR, cm^{-1}) $\tilde{\nu}$ = 3516 (vw), 2958 (vw), 2854 (vw), 1655 (vw), 1647 (vw), 1638 (vw), 1618 (vw), 1543 (vw), 1534 (vw), 1507 (vw), 1460 (w), 1452 (w), 1400 (w), 1322 (vs), 1288 (w), 1255 (s), 1169 (s), 1126 (vs), 1106 (vs), 1052 (vs), 1017 (vs), 914 (w), 884 (w), 827 (vs), 805 (vs), 765 (s), 737 (w), 704 (s), 656 (w), 647 (w), 632 (w), 602 (s), 581 (s), 554 (m), 517 (m), 477 (w), 438 (m), 416 (s), 402 (m), 388 (w), 384 (w).

MS (EI, 70 eV, 20 °C, %) m/z = 254 (86) $[M]^+$, 253 (100) $[M–H]^+$, 223 (34) $[M–CH_3O]^+$, 145 (37) $[M– C_2H_6O_3P]$, 109 (9) $[C_2H_6O_3P]^+$, 79 (50) $[C_6H_7]^+$.

HRMS (EI, $[M]^+$, $C_9H_{10}O_3F_3P$) calcd.: 254.0314; found: 254.0314.

Additional information on the chemical synthesis is available *via* the Chemotion repository:

https://dx.doi.org/10.14272/reaction/SA-FUHFF-UHFFFADPSC-MCZBOCRWDQ-UHFFFADPSC-NUHFF-NUHFF-NUHFF-ZZZ

Additional information on the analysis of the target compound is available *via* the Chemotion repository:

https://dx.doi.org/10.14272/MCZBOCRWDQOCCV-UHFFFAOYSA-N.1

Dimethyl (4-hydroxyphenyl)phosphonate (7i)

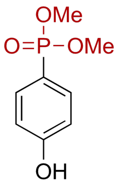

According to the general procedure A, 3-bromoanisole (173 mg, 116 µL) was irradiated for 18 h. The title compound (155 mg, 767 µmol, 77%) was obtained as a colorless oil.

R_f = 0.25 (*n*-pentane/EtOAc, 2:1 + 10% CH_3OH)

^{1}H NMR (500 MHz, $CDCl_3$, ppm) δ = 7.67 – 7.57 (m, 2H, H^{Ar}), 6.99 (dd, J = 8.7, 3.7 Hz, 2H, H^{Ar}), 3.73 (d, J = 11.2 Hz, 6H, POCH_3).

^{13}C NMR (126 MHz, $CDCl_3$, ppm) δ = 161.9 (d, J = 3.5 Hz, C_q, C^{Ar}), 134.0 (+, d, J = 11.7 Hz, 2C, CH, C^{Ar}), 116.2 (+, d, J = 16.5 Hz, 2C, CH, C^{Ar}), 115.0 (d, J = 198.0 Hz, C_q, C^{Ar}), 52.9 (+, d, J = 5.5 Hz, 2C, CH_3, POCH_3).

^{31}P NMR (202 MHz, $CDCl_3$, ppm) δ = 24.46 – 23.40 (m).

IR (ATR, cm^{-1}) $\tilde{\nu}$ = 3386 (vw), 3374 (vw), 3354 (w), 3347 (w), 3322 (w), 3116 (w), 3091 (w), 3078 (w), 3010 (w), 2953 (w), 2873 (w), 2851 (w), 2806 (vw), 2679 (vw), 1604 (m), 1584 (m), 1509 (m), 1441 (w), 1377 (w), 1360 (w), 1319 (vw), 1286 (m), 1210 (vs), 1173 (vs), 1129 (vs), 1106 (m), 1021 (vs), 885 (w), 824 (vs), 773 (vs), 722 (m), 686 (m), 662 (s), 636 (m), 613 (m), 602 (m), 571 (w), 531 (vs), 510 (s), 484 (s), 475 (s), 463 (s), 428 (s), 398 (m), 385 (m), 375 (m).

MS (EI, 70 eV, 60 °C, %) m/z = 202 (5) $[M]^+$, 110 (100) $[M–C_6H_5O]^+$, 109 (60) $[M–C_6H_5OH]^+$, 79 (21) $[C_6H_7]^+$.

HRMS (EI, $[M]^+$, $C_8H_{11}O_4P$) calcd.: 202.0389; found: 202.0391.

Additional information on the chemical synthesis is available *via* the Chemotion repository:

https://dx.doi.org/10.14272/reaction/SA-FUHFF-UHFFFADPSC-YCYLCVMIVJ-UHFFFADPSC-NUHFF-NUHFF-NUHFF-ZZZ

Additional information on the analysis of the target compound is available *via* the Chemotion repository:

https://dx.doi.org/10.14272/YCYLCVMIVJNHOV-UHFFFAOYSA-N.1

Dimethyl (4-cyanophenyl)phosphonate (7j)

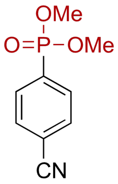

According to the general procedure A, 4-bromobenzonitrile (182 mg) was irradiated for 18 h. The title compound (129 mg, 611 µmol, 61%) was obtained as a colorless oil.

R_f = 0.33 (*n*-pentane/EtOAc, 2:1 + 10% CH_3OH)

^{1}H NMR (500 MHz, $CDCl_3$, ppm) δ = 7.90 (dd, J = 13.1, 8.3 Hz, 2H, H^{Ar}), 7.81 – 7.72 (m, 2H, H^{Ar}), 3.78 (d, J = 11.2 Hz, 6H, POCH_3).

^{13}C NMR (126 MHz, $CDCl_3$, ppm) δ = 132.5 (d, J = 188.6 Hz, C_q, C^{Ar}), 132.5 (+, d, J = 9.8 Hz, 2C, CH, C^{Ar}), 132.2 (+, d, J = 15.0 Hz, 2C, CH, C^{Ar}), 117.8 (C_q, *C*N), 116.4 (d, J = 3.5 Hz, C_q, C^{Ar}), 53.1 (d, J = 5.7 Hz, 2C, CH_3, POCH_3).

^{31}P NMR (202 MHz, $CDCl_3$, ppm) δ = 20.92 – 17.05 (m).

IR (ATR, cm^{-1}) $\tilde{\nu}$ = 3473 (vw), 2955 (w), 2851 (vw), 2232 (w), 1655 (vw), 1647 (w), 1638 (w), 1630 (w), 1601 (w), 1555 (vw), 1494 (w), 1459 (w), 1451 (w), 1394 (w), 1248 (vs), 1183 (m), 1122 (s), 1047 (vs), 1018 (vs), 918 (w), 884 (w), 824 (vs), 809 (vs), 790 (s), 759 (s), 722 (w), 710 (w), 679 (w), 671 (w), 662 (w), 616 (s), 560 (vs), 527 (w), 504 (s), 489 (s), 459 (m), 442 (m), 426 (m), 415 (m), 385 (w).

MS (EI, 70 eV, 20 °C, %) *m/z* = 211 (68) $[M]^+$, 210 (62) $[M–H]^+$, 181 (33) $[M–CH_2O]^+$, 180 (27) $[M–CH_3O]^+$, 79 (47) $[C_6H_7]^+$.

HRMS (EI, $[M]^+$, $C_9H_{10}O_3NP$) calcd.: 211.0393; found: 211.0394.

Additional information on the chemical synthesis is available *via* the Chemotion repository: https://dx.doi.org/10.14272/reaction/SA-FUHFF-UHFFFADPSC-ZEAOUDSHMV-UHFFFADPSC-NUHFF-NUHFF-NUHFF-ZZZ

Additional information on the analysis of the target compound is available *via* the Chemotion repository:

https://dx.doi.org/10.14272/ZEAOUDSHMVQDDV-UHFFFAOYSA-N.1

Dimethyl (4-aminophenyl)phosphonate (7k)

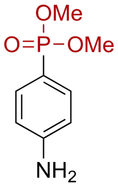

According to the general procedure B, 4-bromoaniline (172 mg) was irradiated for 18 h. The title compound (97.0 mg, 482 µmol, 48%) was obtained as a colorless solid.

R_f = 0.17 (*n*-pentane/EtOAc, 2:1 + 10% CH_3OH)

^{1}H NMR (500 MHz, $CDCl_3$, ppm) δ = 7.55 (dd, *J* = 12.8, 8.5 Hz, 2H, H^{Ar}), 6.68 (dd, *J* = 8.5, 3.6 Hz, 2H, H^{Ar}), 4.00 (s, 2H, NH_2), 3.70 (d, *J* = 11.2 Hz, 6H, POCH_3).

^{13}C NMR (126 MHz, $CDCl_3$, ppm) δ = 150.7 (d, *J* = 2.8 Hz, C_q, C^{Ar}), 133.9 (+, d, *J* = 11.0 Hz, 2C, CH, C^{Ar}), 114.4 (+, d, *J* = 15.7 Hz, 2C, CH, C^{Ar}), 114.1 (d, *J* = 198.8 Hz, C_q, C^{Ar}), 52.6 (+, d, *J* = 5.3 Hz, 2C, CH_3, POCH_3).

^{31}P NMR (202 MHz, $CDCl_3$, ppm) δ = 24.14 – 23.65 (m).

IR (ATR, cm^{-1}) $\tilde{\nu}$ = 3411 (w), 3339 (m), 3228 (w), 3116 (vw), 3078 (vw), 3067 (vw), 3040 (vw), 3012 (vw), 2989 (w), 2948 (w), 2904 (vw), 2847 (vw), 1643 (m), 1598 (vs), 1561 (w), 1531 (w), 1511 (m), 1459 (w), 1445 (w), 1434 (w), 1364 (vw), 1323 (w), 1292 (w), 1220 (vs), 1177 (s), 1126 (vs), 1024 (vs), 952 (m), 918 (w), 836 (s), 816 (vs), 775 (vs), 721 (m), 664 (s), 650 (m), 635 (m), 599 (w), 578 (m), 565 (m), 530 (vs), 500 (vs), 469 (vs), 458 (vs), 412 (vs), 398 (vs).

MS (EI, 70 eV, 20 °C, %) *m/z* = 201 (3) $[M]^+$, 124 (41) $[C_6H_5OP]^+$, 110 (58) $[C_2H_7O_3P]^+$, 94 (100) $[C_6H_8N]^+$, 79 (100) $[C_6H_7]^+$.

HRMS (EI, $[M]^+$, $C_8H_{12}O_3NP$) calcd.: 201.0549; found: 201.0549.

Additional information on the chemical synthesis is available *via* the Chemotion repository:

https://dx.doi.org/10.14272/reaction/SA-FUHFF-UHFFFADPSC-CXGDPEBXNO-UHFFFADPSC-NUHFF-NUHFF-NUHFF-ZZZ

Additional information on the analysis of the target compound is available *via* the Chemotion repository:

https://dx.doi.org/10.14272/CXGDPEBXNOAMCA-UHFFFAOYSA-N.1

Methyl 4-(dimethoxyphosphoryl)benzoate (7o)

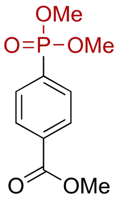

According to the general procedure A, methyl 4-bromobenzoate (215 mg) was irradiated for 18 h. The title compound (179 mg, 733 µmol, 73%) was obtained as a colorless solid.

R_f = 0.33 (*n*-pentane/EtOAc, 2:1 + 10% CH_3OH)

^{1}H NMR (500 MHz, $CDCl_3$, ppm) δ = 8.17 – 8.09 (m, 2H, H^{Ar}), 7.91 – 7.81 (m, 2H, H^{Ar}), 3.94 (s, 3H, CO_2CH_3), 3.78 (d, J = 11.2 Hz, 6H, $POCH_3$).

^{13}C NMR (126 MHz, $CDCl_3$, ppm) δ = 166.1 (C_q, CO_2CH_3), 133.9 (d, J = 3.3 Hz, C_q, C^{Ar}), 131.9 (d, J = 10.1 Hz, C_q, C^{Ar}), 131.3 (+, d, J = 188.2 Hz, 2C, CH, C^{Ar}), 129.5 (+, d, J = 15.3 Hz, 2C, CH, C^{Ar}), 53.0 (+, d, J = 6.0 Hz, 2C, CH_3, $POCH_3$), 52.6 (+, CH_3, OCH_3).

^{31}P NMR (202 MHz, $CDCl_3$, ppm) δ = 20.12 – 19.72 (m).

IR (ATR, cm^{-1}) $\tilde{\nu}$ = 3530 (vw), 3520 (vw), 3503 (vw), 3480 (vw), 3475 (vw), 3462 (vw), 3438 (vw), 3428 (vw), 3000 (vw), 2955 (w), 2853 (vw), 1724 (m), 1643 (vw), 1565 (vw), 1458 (w), 1438 (w), 1397 (w), 1385 (w), 1368 (w), 1309 (w), 1276 (s), 1244 (vs), 1181 (s), 1136 (m), 1103 (m), 1014 (vs), 854 (w), 823 (s), 778 (vs), 762 (vs), 722 (m), 697 (m), 652 (w), 585 (vs), 547 (s), 531 (s), 492 (m), 460 (m), 415 (s), 384 (s).

MS (EI, 70 eV, 30 °C, %) *m/z* = 244 (77) $[M]^+$, 243 (62) $[M–H]^+$, 213 (100) $[M–CH_3O]^+$, 185 (23) $[C_8H_{10}O_3P]^+$.

HRMS (EI, $[M]^+$, $C_{10}H_{13}O_5P$) calcd.: 244.0495; found: 244.0595.

Additional information on the chemical synthesis is available *via* the Chemotion repository:

https://dx.doi.org/10.14272/reaction/SA-FUHFF-UHFFFADPSC-QJGWDEWFFP-UHFFFADPSC-NUHFF-NUHFF-NUHFF-ZZZ

Additional information on the analysis of the target compound is available *via* the Chemotion repository:

https://dx.doi.org/10.14272/QJGWDEWFFPKFQG-UHFFFAOYSA-N.1

Dimethyl (4'-bromo-[1,1'-biphenyl]-4-yl)phosphonate (7p)

According to the general procedure A, 4,4'-dibromo-1,1'-biphenyl (312 mg) was irradiated for 48 h. Dimethyl (4'-bromo-[1,1'-biphenyl]-4-yl)phosphonate (137 mg, 403 µmol, 40%) and tetramethyl [1,1'-biphenyl]-4,4'-diylbis(phosphonate) (220 mg, 594 µmol, 59%) were obtained as colorless solids.

Under the reaction conditions of general procedure B, tetramethyl [1,1'-biphenyl]-4,4'-diylbis(phosphonate) (300 mg, 810 µmol, 81%) was obtained as a colorless solid.

Dimethyl (4'-bromo-[1,1'-biphenyl]-4-yl)phosphonate (**7p**)

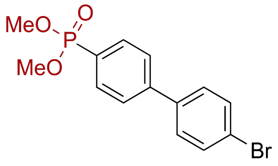

R_f = 0.40 (*n*-pentane/EtOAc, 2:1 + 10% CH_3OH)

^{1}H NMR (500 MHz, $CDCl_3$, ppm) δ = 7.91 – 7.82 (m, 2H, H^{Ar}), 7.68 – 7.62 (m, 2H, H^{Ar}), 7.62 – 7.56 (m, 2H, H^{Ar}), 7.52 – 7.44 (m, 2H, H^{Ar}), 3.78 (d, *J* = 11.1 Hz, 6H, POCH_3).

^{13}C NMR (126 MHz, $CDCl_3$, ppm) δ = 144.3 (d, *J* = 2.9 Hz, C_q, C^{Ar}P), 138.8 (C_q, C^{Ar}), 132.6 (+, d, *J* = 10.1 Hz, 2C, CH, C^{Ar}), 132.2 (+, 2C, CH, C^{Ar}), 128.9 (+, 2C, CH, C^{Ar}), 127.1 (+, d, *J* = 15.4 Hz, 2C, CH, C^{Ar}), 126.0 (d, *J* = 190.6 Hz, C_q, C^{Ar}), 122.8 (C_q, C^{Ar}Br), 52.8 (+, d, *J* = 5.5 Hz, 2C, CH_3, POCH_3).

^{31}P NMR (202 MHz, $CDCl_3$, ppm) δ = 21.51 (dq, *J* = 23.7, 12.2 Hz).

IR (ATR, cm^{-1}) $\tilde{\nu}$ = 3468 (vw), 2951 (w), 2849 (w), 1655 (w), 1647 (w), 1640 (w), 1602 (m), 1548 (w), 1524 (w), 1506 (w), 1480 (m), 1460 (w), 1407 (w), 1384 (w), 1245 (vs), 1181 (m), 1133 (vs), 1051 (vs), 1021 (vs), 1001 (vs), 829 (s), 809 (vs), 796 (vs), 755 (s), 734 (w), 690 (m), 664 (w), 639 (w), 625 (w), 579 (vs), 557 (s), 511 (s), 487 (w), 450 (m), 408 (w), 394 (w).

MS (EI, 70 eV, 50 °C, %) *m/z* = 342/340 (31/31) $[M+H]^+$, 262 (40) $[M+H,–Br]^+$.

HRMS (EI, $[M]^+$, $C_{14}H_{14}O_6{}^{79}BrP$) calcd.: 339.9858; found: 339.9857.

Tetramethyl [1,1'-biphenyl]-4,4'-diylbis(phosphonate) (**7p'**)

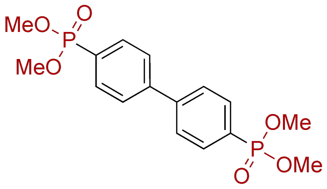

R_f = 0.13 (*n*-pentane/EtOAc, 2:1 + 10% CH_3OH)

^{1}H NMR (500 MHz, $CDCl_3$, ppm) δ = 7.92 – 7.85 (m, 4H, H^{Ar}), 7.72 – 7.66 (m, 4H, H^{Ar}), 3.78 (d, *J* = 11.2 Hz, 12H, POCH_3).

^{13}C NMR (126 MHz, $CDCl_3$, ppm) δ = 144.17 (d, *J* = 3.2 Hz, 2C, C_q, C^{Ar}), 132.67 (+, d, *J* = 10.3 Hz, 4C, CH, C^{Ar}), 127.54 (d, *J* = 15.3 Hz, 4C, CH, C^{Ar}), 125.97 (2C, C_q, C^{Ar}), 52.89 (+, d, *J* = 5.5 Hz, 4C, CH_3, POCH_3).

^{31}P NMR (202 MHz, $CDCl_3$, ppm) δ = 21.17 (hept, *J* = 13.0 Hz).

IR (ATR, cm^{-1}) $\tilde{\nu}$ = 3091 (w), 3031 (w), 2989 (w), 2958 (w), 2854 (w), 1833 (vw), 1737 (vw), 1599 (w), 1541 (w), 1483 (w), 1456 (w), 1388 (w), 1249 (vs), 1183 (m), 1130 (vs), 1044 (vs), 1013 (vs), 994 (vs), 849 (s), 819 (vs), 773 (vs), 720 (vs), 696 (vs), 555 (vs), 523 (vs), 489 (s), 473 (vs), 441 (s), 414 (vs), 380 (s).

MS (FAB, 3-NBA, %) *m/z* = 371 (100) $[M+H]^+$.

HRMS (FAB, $[M+H]^+$, $C_{16}H_{21}O_6P_2$) calcd.: 371.0808; found: 371.0810.

Additional information on the chemical synthesis is available *via* the Chemotion repository:

https://dx.doi.org/10.14272/reaction/SA-FUHFF-UHFFFADPSC-IPTUBKHCCW-UHFFFADPSC-NUHFF-NUHFF-NUHFF-ZZZ

Additional information on the analysis of the target compound is available *via* the Chemotion repository:

https://dx.doi.org/10.14272/RMPIGMRSWDIOMP-UHFFFAOYSA-N.1

https://dx.doi.org/10.14272/GPLCJDYPRFGKAH-UHFFFAOYSA-N.1

Dimethyl (7'-bromo-[1,1'-binaphthalen]-7-yl)phosphonate (4q)

According to the general procedure B, 2,2'-dibromo-1,1'-binaphthalene (412 mg) was irradiated for 18 h. Dimethyl (7'-bromo-[1,1'-binaphthalen]-7-yl)phosphonate (207 mg, 469 µmol, 47%) and tetramethyl [1,1'-binaphthalene]-7,7'-diylbis(phosphonate) (247 mg, 525 µmol, 53%) were obtained as colorless solids.

Dimethyl (7'-bromo-[1,1'-binaphthalen]-7-yl)phosphonate (**7q**)

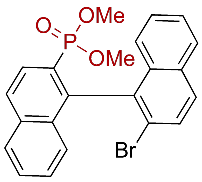

R_f = 0.28 (*n*-pentane/EtOAc, 2:1 + 5% CH_3OH)

^{1}H NMR (500 MHz, $CDCl_3$, ppm) δ = 8.11 (dd, J = 12.0, 8.6 Hz, 1H, H^{Ar}), 8.00 (dd, J = 8.6, 3.7 Hz, 1H, H^{Ar}), 7.90 (dt, J = 8.2, 0.9 Hz, 1H, H^{Ar}), 7.82 (d, J = 8.2 Hz, 1H, H^{Ar}), 7.78 (d, J = 8.8 Hz, 1H, H^{Ar}), 7.73 (d, J = 8.8 Hz, 1H, H^{Ar}), 7.50 (ddd, J = 8.2, 6.7, 1.2 Hz, 1H, H^{Ar}), 7.38 (ddd, J = 8.1, 6.8, 1.2 Hz, 1H, H^{Ar}), 7.26 (ddd, J = 8.2, 6.8, 1.3 Hz, 1H, H^{Ar}), 7.18 (ddd, J = 8.3, 6.8, 1.3 Hz, 1H, H^{Ar}), 7.11 (d, J = 8.7 Hz, 1H, H^{Ar}), 6.93 (d, J = 8.6 Hz, 1H, H^{Ar}), 3.38 (d, J = 11.2 Hz, 3H, POCH_3), 3.11 (d, J = 11.1 Hz, 3H, POCH_3).

^{13}C NMR (126 MHz, $CDCl_3$, ppm) δ = 142.5 (d, J = 9.1 Hz, C_q, C^{Ar}), 136.1 (d, J = 5.4 Hz, C_q, C^{Ar}), 135.3 (d, J = 2.7 Hz, C_q, C^{Ar}), 134.6 (C_q, C^{Ar}), 132.1 (d, J = 16.3 Hz, C_q, C^{Ar}), 131.9 (C_q, C^{Ar}), 129.7 (+, CH, C^{Ar}), 129.7 (+, CH, C^{Ar}), 128.8 (+, d, J = 9.9 Hz, CH, C^{Ar}), 128.3 (+, CH, C^{Ar}), 128.3 (+, CH, C^{Ar}), 128.2 (+, CH, C^{Ar}), 127.9 (+, CH, C^{Ar}), 127.3 (+, CH, C^{Ar}), 126.9 (+, CH, C^{Ar}), 126.8 (+, CH, C^{Ar}), 126.7 (+, CH, C^{Ar}), 126.0 (+, CH, C^{Ar}), 125.4 (d, J = 189.7 Hz, C_q, C^{Ar}), 123.4 (d, J = 1.3 Hz, C_q, C^{Ar}), 52.9 (+, d, J = 6.2 Hz, CH_3, POCH_3), 52.3 (+, d, J = 6.3 Hz, CH_3, POCH_3).

^{31}P NMR (202 MHz, $CDCl_3$, ppm) δ = 19.58 – 19.14 (m).

IR (ATR, cm^{-1}) $\tilde{\nu}$ = 3497 (w), 3432 (w), 3381 (vw), 3055 (w), 2989 (w), 2951 (w), 2904 (w), 2846 (w), 1721 (vw), 1616 (vw), 1579 (w), 1554 (vw), 1502 (w), 1460 (w), 1419 (vw), 1375 (vw), 1347 (vw), 1305 (w), 1262 (w), 1237 (m), 1215 (w), 1169 (w), 1143 (w), 1126 (w), 1112 (w), 1045 (vs), 1016 (vs), 967 (m), 946 (m), 881 (m), 868 (w), 854 (w), 832 (m), 815 (vs), 806 (vs), 782 (m), 768 (vs), 745 (vs), 691 (s), 676 (m), 669 (m), 640 (s), 616 (m), 606 (m), 575 (m), 555 (m), 526 (vs), 516 (vs), 475 (m), 466 (m), 453 (m), 422 (s), 411 (m), 392 (s).

MS (EI, 70 eV, 130 °C, %) m/z = 440/442 (0.5/0.5) $[M]^+$, 361 (100) $[M-Br]^+$.

HRMS (EI, $[M]^+$, $C_{22}H_{18}O_3{}^{79}BrP$) calcd.: 440.0173; found: 440.0173.

Tetramethyl [1,1'-binaphthalene]-7,7'-diylbis(phosphonate) (**7q'**)

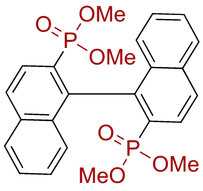

R_f = 0.03 (*n*-pentane/EtOAc, 2:1 + 5% CH_3OH)

^{1}H NMR (500 MHz, $CDCl_3$, ppm) δ = 8.10 – 8.03 (m, 4H, H^{Ar}), 7.94 (d, J = 8.1 Hz, 2H, H^{Ar}), 7.53 (ddd, J = 8.1, 6.7, 1.2 Hz, 2H, H^{Ar}), 7.30 – 7.25 (m, 2H, H^{Ar}), 7.12 (d, J = 8.6 Hz, 2H, H^{Ar}), 3.33 (d, J = 11.1 Hz, 6H, $POCH_3$), 3.29 (d, J = 11.1 Hz, 6H, $POCH_3$).

^{13}C NMR (126 MHz, $CDCl_3$, ppm) δ = 141.7 (dd, J = 9.9, 5.4 Hz, 2C, C_q, C^{Ar}), 134.7 (d, J = 2.8 Hz, 2C, C_q, C^{Ar}), 133.3 (d, J = 16.2 Hz, 2C, C_q, C^{Ar}), 128.2 (+, 2C, CH, C^{Ar}), 128.1 (+, 2C, CH, C^{Ar}), 128.0 (+, d, J = 9.0 Hz, 2C, CH, C^{Ar}), 128.0 (+, 2C, CH, C^{Ar}), 127.9 (+, 2C, CH, C^{Ar}), 127.7 (+, 2C, CH, C^{Ar}), 126.6 (+, 2C, CH, C^{Ar}), 125.9 (d, J = 191.2 Hz, 2C, C_q, C^{Ar}), 52.5 (+, d, J = 6.2 Hz, 2C, CH_3, $POCH_3$), 52.3 (+, d, J = 6.2 Hz, 2C, CH_3, $POCH_3$).

^{31}P NMR (202 MHz, $CDCl_3$, ppm) δ = 19.58 – 19.14 (m).

IR (ATR, cm^{-1}) $\tilde{\nu}$ = 3486 (w), 3437 (w), 3058 (vw), 3010 (vw), 2948 (w), 2846 (w), 1646 (vw), 1587 (w), 1557 (vw), 1499 (w), 1449 (w), 1375 (vw), 1347 (vw), 1309 (w), 1224 (vs), 1180 (m), 1167 (s), 1122 (w), 1099 (w), 1031 (vs), 966 (m), 952 (m), 885 (s), 824 (vs), 800 (s), 783 (m), 766 (vs), 759 (vs), 698 (s), 687 (m), 645 (s), 615 (m), 582 (s), 520 (vs), 465 (s), 426 (s), 399 (vs), 377 (m).

MS (EI, 70 eV, 140 °C, %) *m/z* = 470 (100) $[M]^+$, 361 (90) $[M–C_2H_6O_3P]^+$.

HRMS (EI, $[M]^+$, $C_{24}H_{24}O_6P_2$) calcd.: 470.1043; found: 470.1041.

Additional information on the chemical synthesis is available *via* the Chemotion repository:

https://dx.doi.org/10.14272/reaction/SA-FUHFF-UHFFFADPSC-UJJRSRFDDG-UHFFFADPSC-NUHFF-NUHFF-NUHFF-ZZZ

Additional information on the analysis of the target compound is available *via* the Chemotion repository:

https://dx.doi.org/10.14272/BXJCTHVLVSCBIG-UHFFFAOYSA-N.1

https://dx.doi.org/10.14272/PUAVCXPYXIPSRS-UHFFFAOYSA-N.1

Tetramethyl biphenylene-2,7-diylbis(phosphonate) (7r)

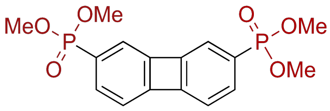

According to the general procedure B, 2,7-dibromobiphenylene (155 mg, 0.50 mmol) was irradiated for 48 h. The title compound (131 mg, 456 µmol, 71%) was obtained as a colorless solid.

R_f = 0.08 (*n*-pentane/EtOAc, 2:1 + 5% CH_3OH)

^{1}H NMR (500 MHz, $CDCl_3$, ppm) δ = 7.40 (ddd, J = 15.3, 7.0, 1.0 Hz, 2H, H^{Ar}), 7.01 (dt, J = 10.9, 1.1 Hz, 2H, H^{Ar}), 6.83 (ddd, J = 7.1, 2.8, 1.0 Hz, 2H, H^{Ar}), 3.76 (d, J = 11.1 Hz, 12H, $POCH_3$).

^{13}C NMR (126 MHz, $CDCl_3$, ppm) δ = 154.6 (d, J = 2.7 Hz, 2C, C_q, C^{Ar}), 151.0 (dd, J = 16.9, 3.1 Hz, 2C, C_q, C^{Ar}), 135.2 (+, d, J = 11.1 Hz, 2C, CH, C^{Ar}), 128.1 (d, J = 188.1 Hz, 2C, C_q, C^{Ar}), 119.6 (+, d, J = 10.1 Hz, 2C, CH, C^{Ar}), 118.3 (+, d, J = 17.2 Hz, 2C, CH, C^{Ar}), 52.9 (+, d, J = 5.6 Hz, 4C, CH_3, POCH_3).

^{31}P NMR (202 MHz, $CDCl_3$, ppm) δ = 21.30 – 20.81 (m).

IR (ATR, cm^{-1}) $\tilde{\nu}$ = 3458 (vw), 3071 (vw), 3053 (vw), 3004 (w), 2983 (w), 2951 (w), 2847 (w), 1748 (vw), 1601 (vw), 1570 (vw), 1460 (w), 1445 (w), 1384 (w), 1356 (w), 1283 (vw), 1247 (vs), 1230 (vs), 1181 (s), 1152 (m), 1129 (m), 1111 (w), 1099 (w), 1078 (vs), 1044 (vs), 1014 (vs), 972 (vs), 909 (m), 817 (vs), 778 (vs), 749 (vs), 703 (vs), 642 (w), 622 (w), 601 (m), 591 (m), 554 (vs), 531 (vs), 482 (m), 460 (s), 435 (s), 419 (s), 382 (s).

MS (EI, 70 eV, 130 °C, %) m/z = 368 (100) $[M]^+$.

HRMS (EI, $[M]^+$, $C_{16}H_{18}O_6P_2$) calcd.: 368.0573; found: 368.0573.

Additional information on the chemical synthesis is available *via* the Chemotion repository:

https://dx.doi.org/10.14272/reaction/SA-FUHFF-UHFFFADPSC-GKOVDFONJF-UHFFFADPSC-NUHFF-NUHFF-NUHFF-ZZZ

Additional information on the analysis of the target compound is available *via* the Chemotion repository:

https://dx.doi.org/10.14272/GKOVDFONJFCCTM-UHFFFAOYSA-N.1

Hexamethyl benzene-1,3,5-triyltris(phosphonate) (7s)

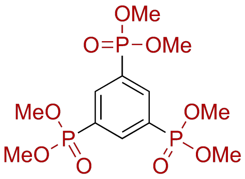

According to the general procedure B, 1,3,5-tribromobenzene (315 mg) and trimethylphospite (11.8 mL, 100 mmol, 100 equiv.) were irradiated for 48 h. The title compound (330 mg, 821 µmol, 82%) was obtained as a colorless solid.

R_f = 0.06 (n-pentane/EtOAc, 2:1 + 5% CH_3OH)

^1H NMR (400 MHz, $CDCl_3$, ppm) δ = 8.45 – 8.28 (m, 3H, H^{Ar}), 3.80 (d, J = 10.9 Hz, 18H, POCH_3).

^{13}C NMR (101 MHz, $CDCl_3$, ppm) δ = 139.3 – 138.2 (+, m, 3C, CH, C^{Ar}), 129.3 (dt, J = 191.7, 13.5 Hz, 3C, C_q, C^{Ar}), 53.3 – 53.2 (+, m, 6C, CH_3, POCH_3).

^{31}P NMR (202 MHz, $CDCl_3$, ppm) δ = 18.33 – 17.23 (m).

IR (ATR, cm^{-1}) $\tilde{\nu}$ = 3054 (vw), 2999 (vw), 2956 (w), 2853 (vw), 1579 (w), 1462 (w), 1448 (w), 1402 (w), 1309 (vw), 1252 (vs), 1183 (m), 1126 (w), 1007 (vs), 849 (vs), 826 (vs), 788 (m), 779 (m), 741 (vs), 700 (s), 545 (vs), 523 (vs), 475 (s), 418 (s), 412 (s), 375 (m).

MS (FAB, 3-NBA, %) m/z = 403 (100) $[M+H]^+$.

HRMS (FAB, $[M+H]^+$, $C_{12}H_{22}O_9P_3$) calcd.: 403.0471; found: 403.0470.

Additional information on the chemical synthesis is available *via* the Chemotion repository:

https://dx.doi.org/10.14272/reaction/SA-FUHFF-UHFFFADPSC-SZWWJNWOTT-UHFFFADPSC-NUHFF-NUHFF-NUHFF-ZZZ

Additional information on the analysis of the target compound is available *via* the Chemotion repository:

https://dx.doi.org/10.14272/SZWWJNWOTTYFSA-UHFFFAOYSA-N.1

Dimethyl pyrazin-2-ylphosphonate (7t)

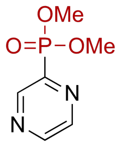

According to the general procedure A, 2-bromopyrazine (159 mg) was irradiated for 18 h. The title compound (50.4 mg, 268 µmol, 27%) was obtained as a colorless solid.

R_f = 0.13 (*n*-pentane/EtOAc, 2:1 + 10% CH_3OH)

^{1}H NMR (500 MHz, $CDCl_3$, ppm) δ = 9.10 (t, *J* = 1.3 Hz, 1H, H^{Ar}), 8.82 – 8.76 (m, 1H, H^{Ar}), 8.74 (dd, *J* = 3.8, 2.5 Hz, 1H, H^{Ar}), 3.91 (d, *J* = 11.0 Hz, 6H, POCH_3).

^{13}C NMR (126 MHz, $CDCl_3$, ppm) δ = 148.3 (+, d, *J* = 26.4 Hz, CH, C^{Ar}), 147.3 (+, d, *J* = 3.5 Hz, CH, C^{Ar}), 147.2 (d, *J* = 226.3 Hz, C_q, C^{Ar}), 145.4 (+, d, *J* = 18.5 Hz, CH, C^{Ar}), 54.0 (+, d, *J* = 6.3 Hz, 2C, CH_3, POCH_3).

^{31}P NMR (202 MHz, $CDCl_3$, ppm) δ = 11.19 – 10.63 (m)

IR (ATR, cm^{-1}) $\tilde{\nu}$ = 3394 (m), 3381 (m), 3373 (m), 3361 (m), 3325 (m), 3313 (m), 3293 (m), 3278 (m), 3272 (m), 3108 (w), 3050 (w), 3030 (w), 3016 (w), 2955 (w), 2919 (w), 2849 (w), 1656 (w), 1452 (w), 1392 (w), 1231 (vs), 1186 (s), 1169 (m), 1135 (w), 1077 (vs), 1034 (vs), 1017 (vs), 875 (w), 837 (w), 775 (s), 739 (m), 694 (w), 679 (w), 673 (w), 662 (w), 649 (w), 622 (w), 560 (s), 527 (s), 484 (w), 459 (w), 452 (w), 424 (w), 415 (w), 394 (vw).

MS (EI, 70 eV, 50 °C, %) *m/z* = 188 (18) $[M]^+$, 173 (23) $[M–CH_3]^+$, 158 (36) $[M–CH_2O]^+$, 109 (22) $[C_2H_6O_3P]^+$, 80 (100) $[M–C_2H_6O_3P]^+$.

HRMS (EI, $[M]^+$, $C_6H_9O_3N_2P$) calcd.: 188.0345; found: 188.0344.

Additional information on the chemical synthesis is available *via* the Chemotion repository:

https://dx.doi.org/10.14272/reaction/SA-FUHFF-UHFFFADPSC-DKGZMEBNFM-UHFFFADPSC-NUHFF-NUHFF-NUHFF-ZZZ

Additional information on the analysis of the target compound is available *via* the Chemotion repository:

https://dx.doi.org/10.14272/DKGZMEBNFMMTSE-UHFFFAOYSA-N.1

Dimethyl benzo[*b*]thiophen-5-ylphosphonate (7u)

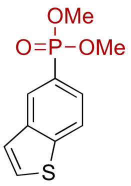

According to the general procedure A, 5-bromo-1-benzothiophene (213 mg) was irradiated for 18 h. The title compound (166 mg, 684 µmol, 68%) was obtained as a colorless solid.

R_f = 0.26 (*n*-pentane/EtOAc, 2:1 + 10% CH_3OH)

^{1}H NMR (500 MHz, $CDCl_3$, ppm) δ = 8.33 (d, *J* = 14.6 Hz, 1H, SC*H*), 8.04 – 7.94 (m, 1, H^{Ar}), 7.69 (ddd, *J* = 11.9, 8.3, 1.3 Hz, 1H, H^{Ar}), 7.54 (d, *J* = 5.5 Hz, 1H, H^{Ar}), 7.42 (dd, *J* = 5.5, 0.6 Hz, 1H, H^{Ar}), 3.78 (d, *J* = 11.1 Hz, 6H, POCH_3).

^{13}C NMR (126 MHz, $CDCl_3$, ppm) δ = 144.0 (d, *J* = 3.4 Hz, C_q, C^{Ar}), 139.3 (d, *J* = 17.2 Hz, C_q, C^{Ar}), 128.4 (+, d, *J* = 10.9 Hz, CH, S*C*H), 128.0 (+, CH, C^{Ar}), 126.2 (+, d, *J* = 11.5 Hz, CH, C^{Ar}), 124.2 (+, d, *J* = 1.1 Hz, CH, SCH*C*H), 123.0 (+, d, *J* = 16.2 Hz, CH, C^{Ar}), 122.4 (d, *J* = 190.5 Hz, C_q, C^{Ar}POCH_3), 52.8 (+, d, *J* = 5.4 Hz, 2C, CH_3, POCH_3).

^{31}P NMR (202 MHz, $CDCl_3$, ppm) δ = 22.96 – 22.46 (m).

IR (ATR, cm^{-1}) $\tilde{\nu}$ = 3497 (vw), 3482 (vw), 3468 (vw), 3454 (vw), 3439 (vw), 3072 (vw), 2992 (vw), 2949 (w), 2847 (vw), 1589 (vw), 1459 (w), 1445 (w), 1434 (w), 1405 (w), 1322 (w), 1307 (w), 1244 (vs), 1203 (m), 1181 (m), 1108 (m), 1088 (w), 1055 (s), 1020 (vs), 912 (m), 823 (s), 773 (vs), 734 (w), 701 (m), 681 (w), 599 (s), 523 (m), 493 (m), 433 (w), 381 (vw).

MS (EI, 70 eV, 20 °C, %) *m/z* = 242 (36) $[M]^+$, 227 (3) $[M–CH_2]^+$, 134 (16) $[M–C_2H_5O_3P]^+$, 110 (100) $[C_2H_7O_3P]^+$.

HRMS (EI, $[M]^+$, $C_{10}H_{11}O_3PS$) calcd.: 242.0161; found: 242.0163.

Additional information on the chemical synthesis is available *via* the Chemotion repository:

https://dx.doi.org/10.14272/reaction/SA-FUHFF-UHFFFADPSC-VLRAYJRKPY-UHFFFADPSC-NUHFF-NUHFF-NUHFF-ZZZ

Additional information on the analysis of the target compound is available *via* the Chemotion repository:

https://dx.doi.org/10.14272/VLRAYJRKPYKDDY-UHFFFAOYSA-N.1

Dimethyl benzofuran-5-ylphosphonate (7v)

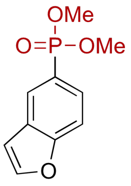

According to the general procedure A, 5-bromo-1-benzofuran (197 mg) was irradiated for 18 h. The title compound (161 mg, 713 µmol, 71%) was obtained as a colorless solid.

R_f = 0.34 (*n*-pentane/EtOAc, 2:1 + 10% CH_3OH)

^{1}H NMR (500 MHz, $CDCl_3$, ppm) δ = 8.16 – 8.08 (m, 1H, OC*H*), 7.75 – 7.68 (m, 2H, H^{Ar}), 7.62 – 7.56 (m, 1H, H^{Ar}), 6.84 (dd, *J* = 2.2, 0.9 Hz, 1H, H^{Ar}), 3.76 (d, *J* = 11.1 Hz, 6H, POCH_3).

^{13}C NMR (126 MHz, $CDCl_3$, ppm) δ = 157.2 (d, *J* = 3.5 Hz, C_q, C^{Ar}), 146.4 (+, CH, OCH*C*H), 127.8 (+, d, *J* = 12.0 Hz, CH, C^{Ar}), 127.8 (d, *J* = 17.4 Hz, C_q, C^{Ar}), 126.5 (+, d, *J* = 11.7 Hz, CH, C^{Ar}), 121.0 (d, *J* = 190.9 Hz, C_q, C^{Ar}POCH_3), 112.0 (+, d, *J* = 16.5 Hz, CH, C^{Ar}), 106.9 (+, CH, O*C*H), 52.8 (+, d, *J* = 5.4 Hz, 2C, CH_3, POCH_3).

^{31}P NMR (202 MHz, $CDCl_3$, ppm) δ = 23.17 – 22.61 (m).

IR (ATR, cm^{-1}) $\tilde{\nu}$ = 3472 (vw), 3463 (vw), 3455 (vw), 3441 (vw), 3108 (vw), 2952 (w), 2850 (vw), 1608 (vw), 1537 (w), 1458 (w), 1429 (w), 1334 (vw), 1317 (vw), 1254 (vs), 1242 (vs), 1181 (m), 1133 (w), 1115 (w), 1084 (s), 1048 (vs), 1020 (vs), 924 (w), 905 (w), 880 (m), 817 (s), 769 (vs), 742 (m), 698 (w), 619 (w), 594 (m), 541 (s), 518 (w), 486 (w), 452 (w), 424 (w), 409 (w), 399 (w).

MS (EI, 70 eV, 20 °C, %) *m/z* = 226 (100) $[M]^+$, 211 (15) $[M–CH_3]^+$, 195 (15) $[M–CH_3O]^+$, 118 (42) $[M–C_2H_5O_3P]^+$.

HRMS (EI, $[M]^+$, $C_{10}H_{11}O_4P$) calcd.: 226.0389; found: 226.0391.

Additional information on the chemical synthesis is available *via* the Chemotion repository:

https://dx.doi.org/10.14272/reaction/SA-FUHFF-UHFFFADPSC-IBWSCSMJGL-UHFFFADPSC-NUHFF-NUHFF-NUHFF-ZZZ

Additional information on the analysis of the target compound is available *via* the Chemotion repository:

https://dx.doi.org/10.14272/IBWSCSMJGLEUPW-UHFFFAOYSA-N.1

Dimethyl benzofuran-3-ylphosphonate (7w)

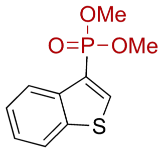

According to the general procedure A, 3-bromo-1-benzothiophene (213 mg) was irradiated for 5 h. The title compound (165 mg, 680 µmol, 68%) was obtained as a colorless solid.

R_f = 0.25 (*n*-pentane/EtOAc, 2:1 + 10% CH_3OH)

^1H NMR (500 MHz, $CDCl_3$, ppm) δ = 8.26 (d, *J* = 9.6 Hz, 1H, SC*H*), 8.15 – 8.06 (m, 1H, H^{Ar}), 7.94 – 7.89 (m, 1H, H^{Ar}), 7.49 – 7.44 (m, 1H, H^{Ar}), 7.44 – 7.40 (m, 1H, H^{Ar}), 3.79 (d, *J* = 11.4 Hz, 6H, $POCH_3$).

^{13}C NMR (126 MHz, $CDCl_3$, ppm) δ = 140.8 (d, *J* = 17.4 Hz, C_q, C^{Ar}), 140.0 (+, d, *J* = 17.7 Hz, CH, C^{Ar}), 138.2 (d, *J* = 16.3 Hz, C_q, C^{Ar}), 125.4 (+, CH, C^{Ar}), 125.3 (+, CH, C^{Ar}), 124.1 (+, CH, O*C*H), 122.7 (+, CH, C^{Ar}), 122.6 (d, *J* = 20.5 Hz, C_q, C^{Ar}), 52.8 (+, d, *J* = 5.4 Hz, 2C, CH_3, $POCH_3$).

^{31}P NMR (202 MHz, $CDCl_3$, ppm) δ = 15.33 (h, *J* = 11.2 Hz).

IR (ATR, cm^{-1}) $\tilde{\nu}$ = 3524 (vw), 3514 (vw), 3493 (vw), 3480 (vw), 3465 (vw), 3424 (vw), 3094 (vw), 3060 (vw), 2993 (vw), 2951 (w), 2849 (vw), 1487 (w), 1456 (w), 1422 (m), 1324 (w), 1242 (vs), 1181 (m), 1137 (w), 1047 (vs), 1018 (vs), 989 (s), 824 (s), 778 (s), 759 (s), 734 (s), 704 (w), 605 (w), 572 (s), 551 (m), 528 (m), 503 (w), 470 (w), 429 (w).

MS (EI, 70 eV, 20 °C, %) *m/z* = 242 (22) $[M]^+$, 227 (4) $[M–CH_2]^+$, 134 (16) $[M–C_2H_5O_3P]^+$, 110 (100) $[C_2H_7O_3P]^+$.

HRMS (EI, $[M]^+$, $C_{10}H_{11}O_3PS$) calcd.: 242.0161; found: 242.0160.

Additional information on the chemical synthesis is available *via* the Chemotion repository:

https://dx.doi.org/10.14272/reaction/SA-FUHFF-UHFFFADPSC-OXFZBTGIHM-UHFFFADPSC-NUHFF-NUHFF-NUHFF-ZZZ

Additional information on the analysis of the target compound is available *via* the Chemotion repository:

https://dx.doi.org/10.14272/OXFZBTGIHMLUTO-UHFFFAOYSA-N.1

Dimethyl benzofuran-3-ylphosphonate (7x)

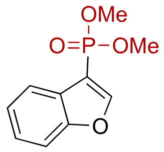

According to the general procedure A, 5-bromo-1-benzofuran (197 mg) was irradiated for 5 h. The title compound (105 mg, 505 µmol, 51%) was obtained as a colorless solid.

R_f = 0.37 (*n*-pentane/EtOAc, 2:1 + 10% CH_3OH)

^{1}H NMR (500 MHz, $CDCl_3$, ppm) δ = 8.10 (d, J = 2.6 Hz, 1H, OC*H*), 7.78 – 7.72 (m, 1H, H^{Ar}), 7.60 – 7.53 (m, 1H, H^{Ar}), 7.38 (td, J = 7.8, 1.4 Hz, 1H, H^{Ar}), 7.34 (td, J = 7.5, 1.0 Hz, 1H, H^{Ar}), 3.80 (d, J = 11.5 Hz, 6H, POCH_3).

^{13}C NMR (126 MHz, $CDCl_3$, ppm) δ = 155.7 (d, J = 14.5 Hz, C_q, C^{Ar}), 153.2 (+, d, J = 26.3 Hz, CH, O*C*H), 125.9 (d, J = 10.5 Hz, C_q, C^{Ar}), 125.7 (+, CH, C^{Ar}), 124.2 (+, CH, C^{Ar}), 121.5 (+, CH, C^{Ar}), 111.9 (+, CH, C^{Ar}), 107.6 (d, J = 214.5 Hz, C_q, *C*PO*C*H_3), 52.8 (+, d, J = 4.9 Hz, 2C, CH_3, PO*C*H_3).

^{31}P NMR (202 MHz, $CDCl_3$, pp1m) δ = 16.13 – 15.66 (m).

IR (ATR, cm^{-1}) $\tilde{\nu}$ = 3479 (vw), 3119 (vw), 3057 (vw), 2953 (w), 2850 (vw), 1611 (vw), 1587 (vw), 1540 (m), 1472 (w), 1451 (s), 1317 (w), 1268 (s), 1247 (vs), 1181 (m), 1171 (m), 1152 (w), 1111 (s), 1051 (vs), 1020 (vs), 939 (w), 878 (w), 858 (m), 827 (vs), 799 (m), 785 (s), 751 (vs), 654 (s), 612 (m), 572 (vs), 554 (s), 534 (s), 487 (m), 476 (m), 424 (m), 404 (w).

MS (EI, 70 eV, 20 °C, %) *m/z* = 226 (100) $[M]^+$, 211 (27) $[M–CH_3]^+$, 118 (25) $[M–C_2H_5O_3P]^+$.

HRMS (EI, $[M]^+$, $C_{10}H_{11}O_4P$) calcd.: 226.0389; found: 226.0387.

Additional information on the chemical synthesis is available *via* the Chemotion repository:

https://dx.doi.org/10.14272/reaction/SA-FUHFF-UHFFFADPSC-YCMNRDITMR-UHFFFADPSC-NUHFF-NUHFF-NUHFF-ZZZ

Additional information on the analysis of the target compound is available *via* the Chemotion repository:

https://dx.doi.org/10.14272/YCMNRDITMRMPHO-UHFFFAOYSA-N.1

Phenylphosphonic acid (8a)

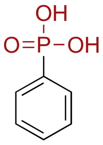

Dimethyl phenylphosphonate (559 mg, 3.00 mmol, 1.00 equiv.) was dissolved in hydrochloric acid (12 M aq. solution, 4.2 mL, 51.0 mmol, 17.0 equiv.) and heated to 100 °C for 12 h. The solvent was removed under reduced pressure to obtain the title compound (474 mg, 2.99 mmol, 99%) as a colorless solid.

^{1}H NMR (400 MHz, DMSO-d6, ppm) δ = 9.39 (bs, 2H, PO*H*), 7.71–7.65 (m, 2H, H^{Ar}), 7.53–7.42 (m, 3H, H^{Ar}).

^{13}C NMR (100 MHz, DMSO-d6, ppm) δ = 134.1 (d, *J* = 180.9 Hz, C_q, C^{Ar}), 130.9 (+, d, *J* = 2.9 Hz, CH, C^{Ar}), 130.5 (+, d, *J* = 9.8 Hz, 2C, CH, C^{Ar}), 128.1 (+, d, *J* = 14.1 Hz, 2C, CH, C^{Ar}).

^{31}P NMR (162 MHz, DMSO-d6, ppm) δ = 12.81 (s).

IR (ATR, cm^{-1}) $\tilde{\nu}$ = 2687 (m), 2213 (m), 2126 (m), 1592 (w), 1486 (w), 1438 (m), 1215 (m), 1142 (s), 1115 (s), 1074 (vs), 1010 (vs), 997 (vs), 935 (vs), 922 (vs), 783 (s), 752 (vs), 714 (vs), 693 (vs), 543 (vs), 528 (vs), 511 (vs), 484 (vs), 441 (vs), 418 (vs).

MS (EI, 70 eV, 150 °C, %) *m/z* = 158 (100) [M]$^+$.

HRMS (EI, [M]$^+$, $C_6H_7O_3P$) calcd.: 158.0127; found: 158.0126.

Additional information on the chemical synthesis is available *via* the Chemotion repository:

https://dx.doi.org/10.14272/reaction/SA-FUHFF-UHFFFADPSC-QLZHNIAADX-UHFFFADPSC-NUHFF-NUHFF-NUHFF-ZZZ

Additional information on the analysis of the target compound is available *via* the Chemotion repository:

https://dx.doi.org/10.14272/QLZHNIAADXEJJP-UHFFFAOYSA-N.1

(4-Phosphonophenyl)phosphonic acid (8b)

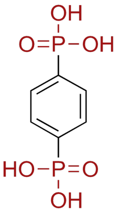

Tetramethyl 1,4-phenylenebis(phosphonate) (147 mg, 0.50 mmol, 1.00 equiv.) was dissolved in hydrochloric acid (12 M aq. solution, 1.42 mL, 17.0 mmol, 34.0 equiv.) and heated to 100 °C for 12 h. The solvent was removed under reduced pressure to obtain the title compound (119 mg, 0.49 mmol, 99%) as a colorless solid.

^{1}H NMR (400 MHz, DMSO-d6, ppm) δ = 8.06 (bs, 4H), 7.74 (s, 4H).

^{13}C NMR (100 MHz, DMSO-d6, ppm) δ = 136.7 (d, *J* = 179.2 Hz, 2C, C_q, C^{Ar}), 130.14 (+, t, *J* = 11.8 Hz, 4C, CH, C^{Ar}).

^{31}P NMR (162 MHz, DMSO-d6, ppm) δ = 11.74 (s).

IR (ATR, cm^{-1}) $\tilde{\nu}$ = 2597 (w), 1383 (w), 1122 (s), 1095 (s), 1024 (s), 992 (vs), 931 (vs), 827 (vs), 727 (s), 591 (vs), 547 (vs), 445 (vs), 392 (vs).

HRMS (ESI, [M-H]$^-$, $C_6H_7O_6P_2$) calcd.: 236.9723, found: 236.9719.

Additional information on the chemical synthesis is available *via* the Chemotion repository:

https://dx.doi.org/10.14272/reaction/SA-FUHFF-UHFFFADPSC-JHDJUJAFXN-UHFFFADPSC-NUHFF-NUHFF-NUHFF-ZZZ

Additional information on the analysis of the target compound is available *via* the Chemotion repository:

https://dx.doi.org/10.14272/JHDJUJAFXNIIIW-UHFFFAOYSA-N.1

[4-(4-Phosphonophenyl)phenyl]phosphonic acid (8c)

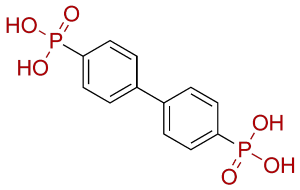

1-Dimethoxyphosphoryl-4-(4-dimethoxyphosphorylphenyl) benzene (122 mg, 0.33 mmol, 1.00 equiv.) was dissolved in hydrochloric acid (12 M aq. solution, 0.94 mL, 11.2 mmol, 34.0 equiv.) and heated to 100 °C for 12 h. The solvent was removed under reduced pressure to obtain the title compound (103 mg, 0.33 mmol, 99%) as a colorless solid.

^{1}H NMR (500 MHz, DMSO-d6, ppm) δ = 7.83 – 7.72 (m, 8H).

^{13}C NMR (126 MHz, DMSO-d6, ppm) δ = 141.7 (d, *J* = 3.1 Hz, 2C, C_q, C^{Ar}), 133.6 (d, *J* = 181.9 Hz, 2C, C_q, C^{Ar}), 131.2 (+, d, *J* = 10.0 Hz, 4C, CH, C^{Ar}), 126.6 (+, d, *J* = 14.4 Hz, 4C, CH, C^{Ar}).

^{31}P NMR (202 MHz, DMSO-d6, ppm) *δ* = 12.47 (s).

IR (ATR, cm^{-1}) $\tilde{\nu}$ = 2721 (w), 1602 (w), 1387 (w), 1145 (m), 1098 (s), 1007 (vs), 997 (vs), 942 (vs), 853 (w), 833 (m), 815 (vs), 711 (vs), 545 (vs), 520 (vs), 460 (vs), 405 (m), 390 (m).

HRMS (ESI, [M-H]$^-$, $C_6H_7O_6P_2$) calcd.: 313.0036, found: 313.0036.

Additional information on the chemical synthesis is available *via* the Chemotion repository:

https://dx.doi.org/10.14272/reaction/SA-FUHFF-UHFFFADPSC-QWSZMKCGMD-UHFFFADPSC-NUHFF-NUHFF-NUHFF-ZZZ

Additional information on the analysis of the target compound is available *via* the Chemotion repository:

https://dx.doi.org/10.14272/QWSZMKCGMDROKE-UHFFFAOYSA-N.1

3. NMR Spectra

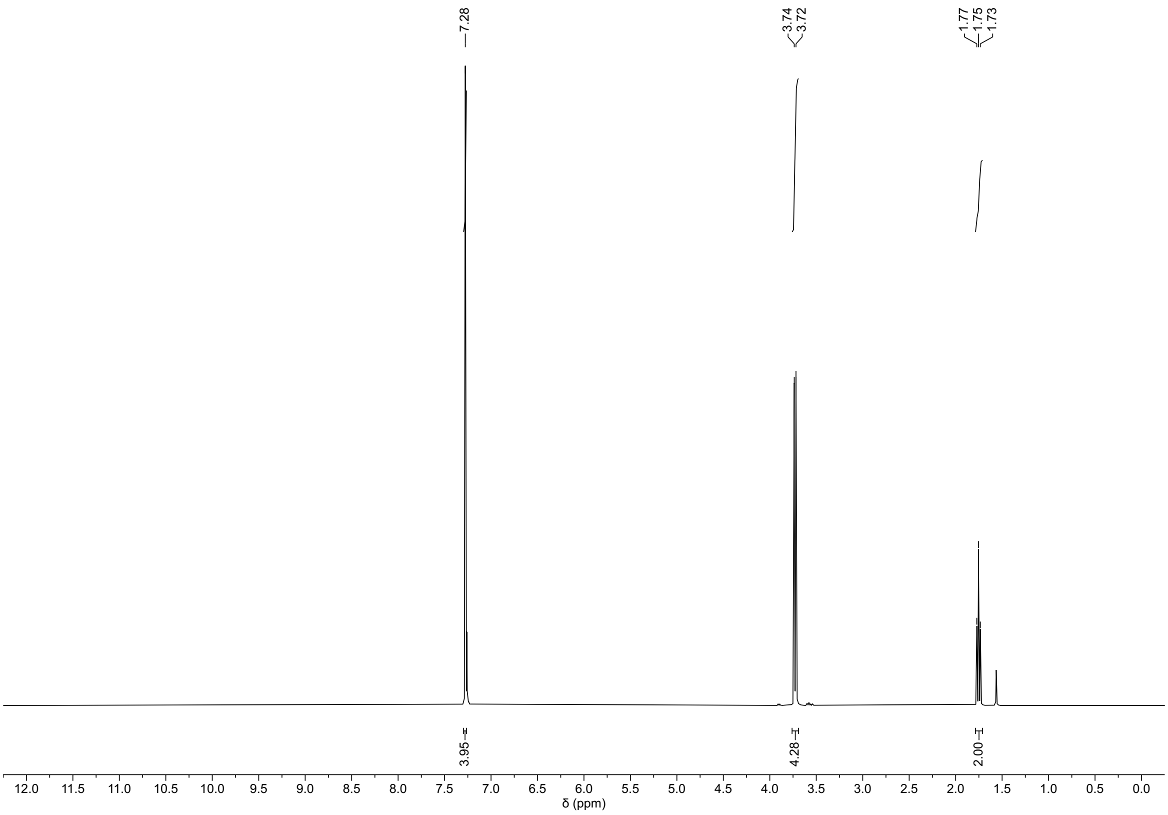

Figure 1. ^{1}H NMR – 1,4-Phenylenedimethanethiol (**1**), 400 MHz, $CDCl_3$.

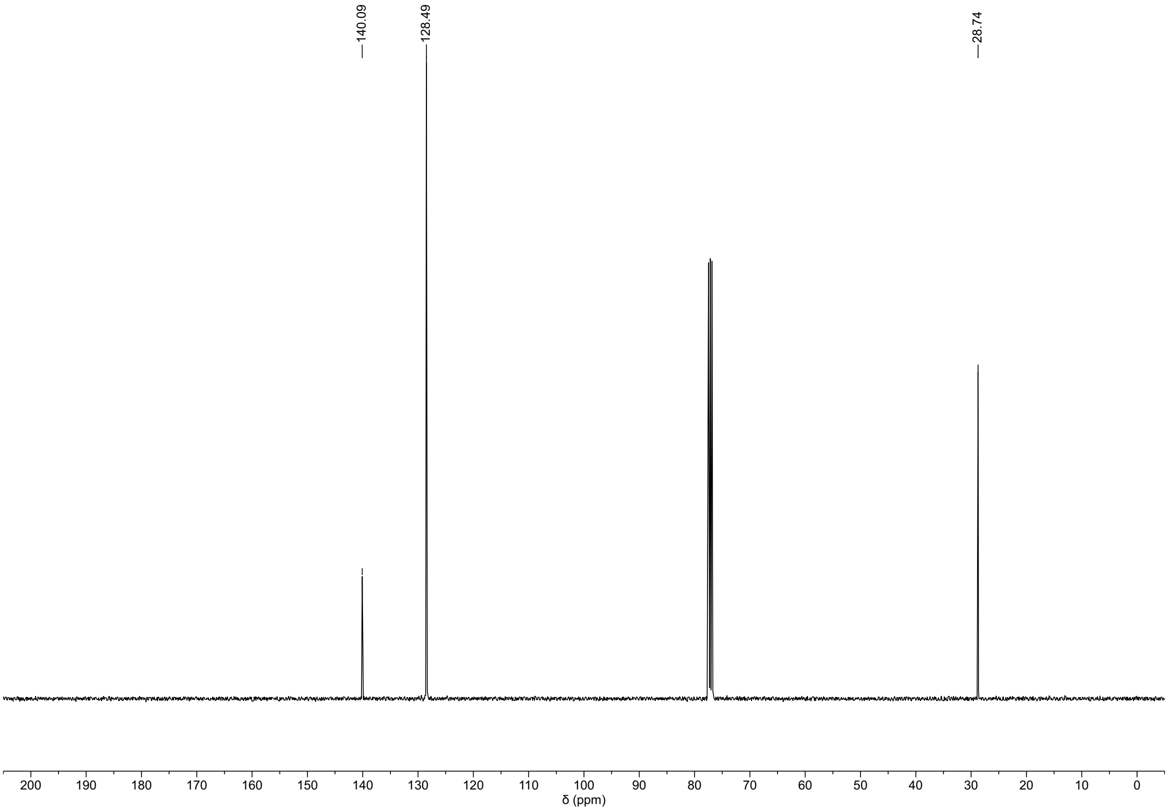

Figure 2. ^{13}C NMR – 1,4-Phenylenedimethanethiol (**1**), 101 MHz, $CDCl_3$.

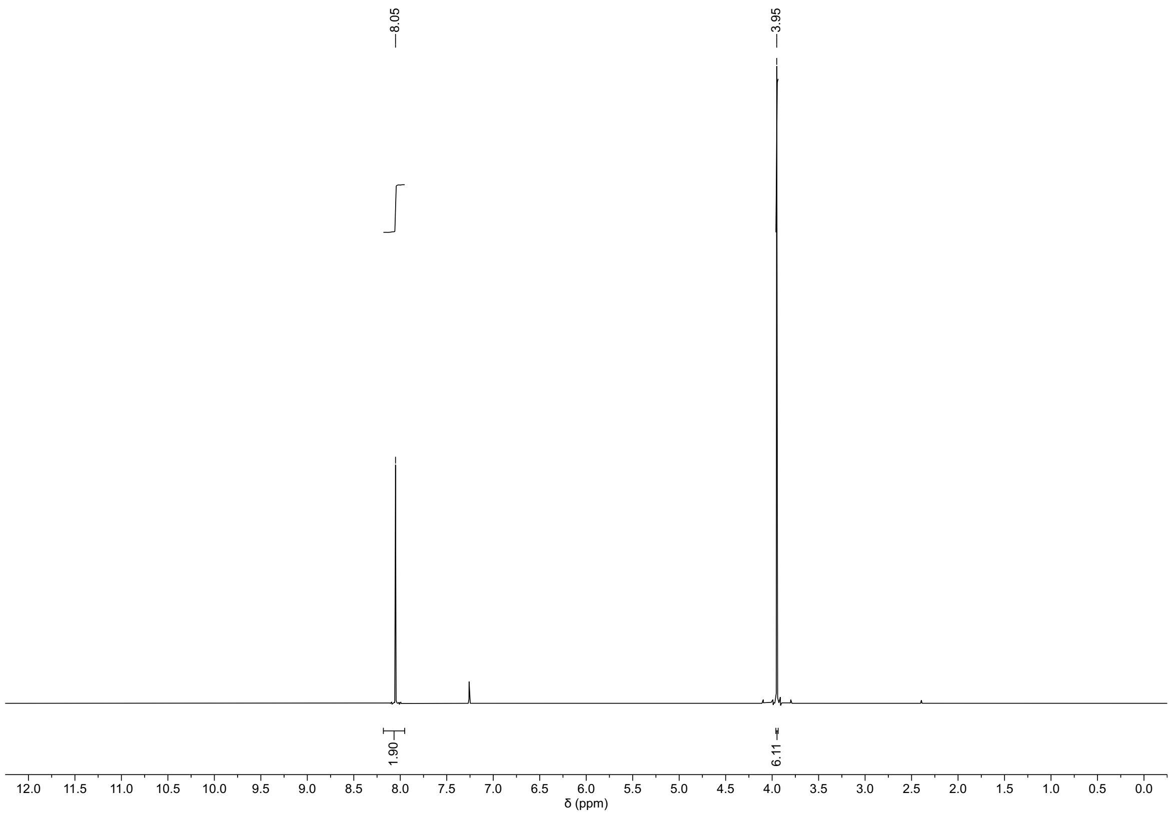

Figure 3. ^{1}H NMR – Dimethyl 2,5-dibromobenzene-1,4-dicarboxylate, 500 MHz, $CDCl_3$.

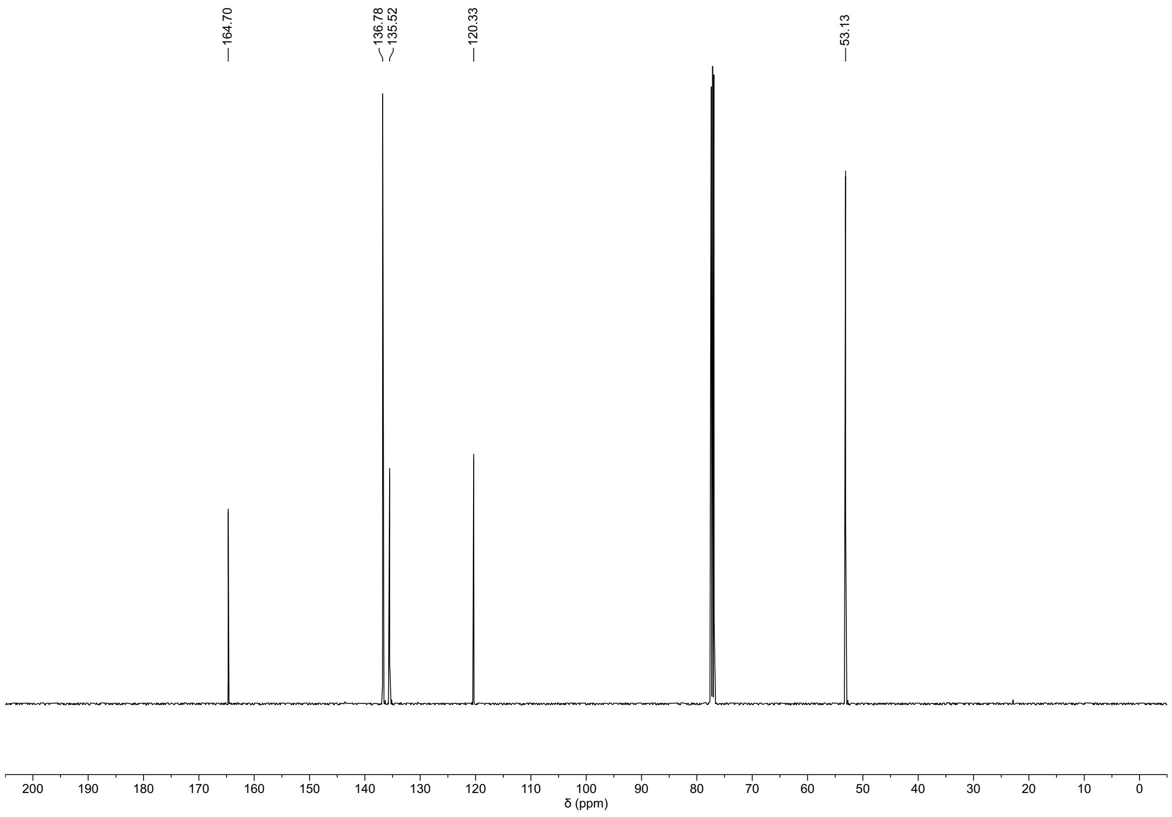

Figure 4. ^{13}C NMR – Dimethyl 2,5-dibromobenzene-1,4-dicarboxylate, 126 MHz, $CDCl_3$.

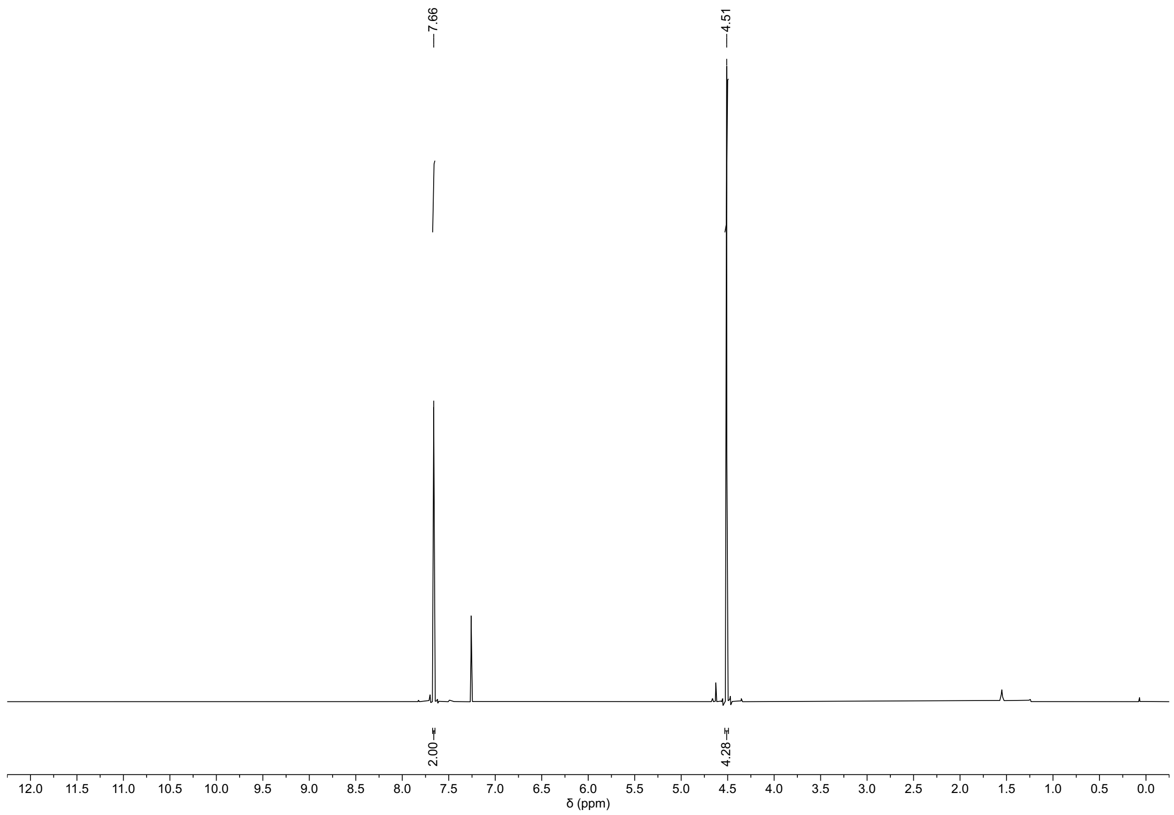

Figure 5. ^{1}H NMR – 1,4-Dibromo-2,5-bis(bromomethyl)benzene (**2**), 500 MHz, $CDCl_3$.

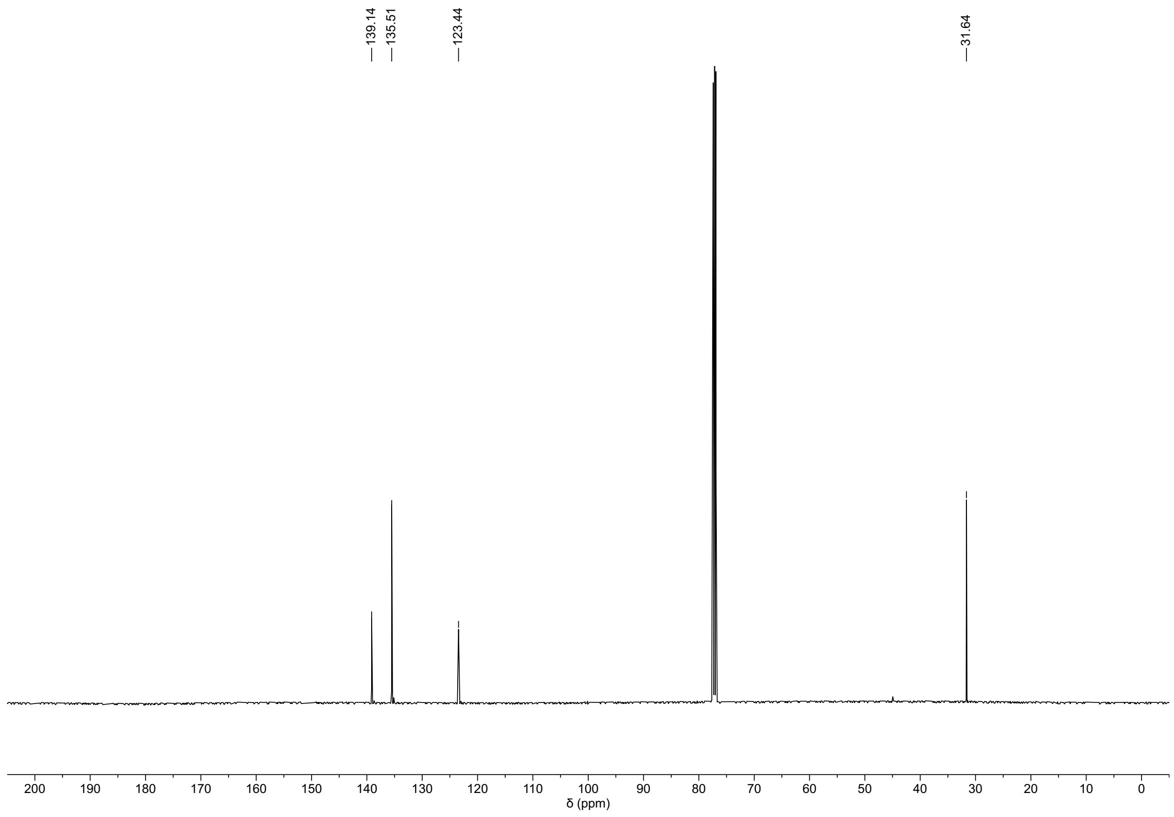

Figure 6. ^{13}C NMR – 1,4-Dibromo-2,5-bis(bromomethyl)benzene (**2**), 126 MHz, $CDCl_3$.

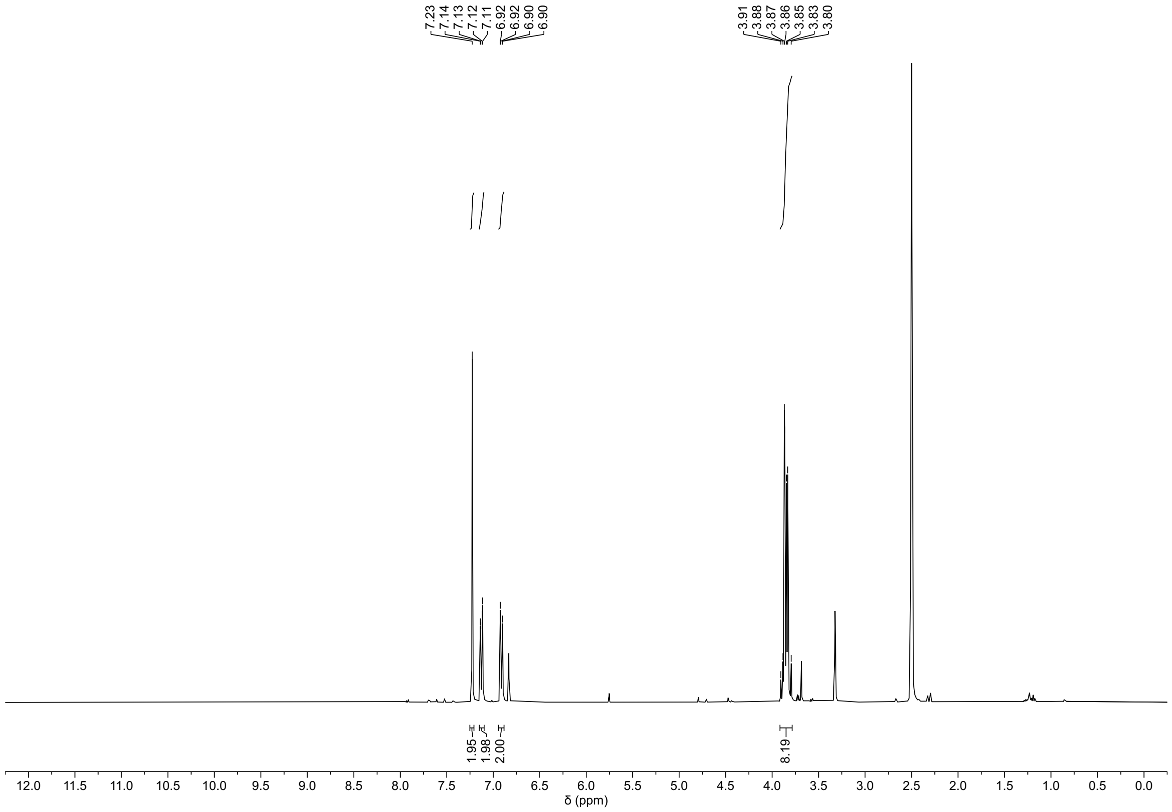

Figure 7. ^{1}H NMR – 5,8-Dibromo-2,11-dithia[3.3]paracyclophane (**3**), 400 MHz, DMSO-d_6.

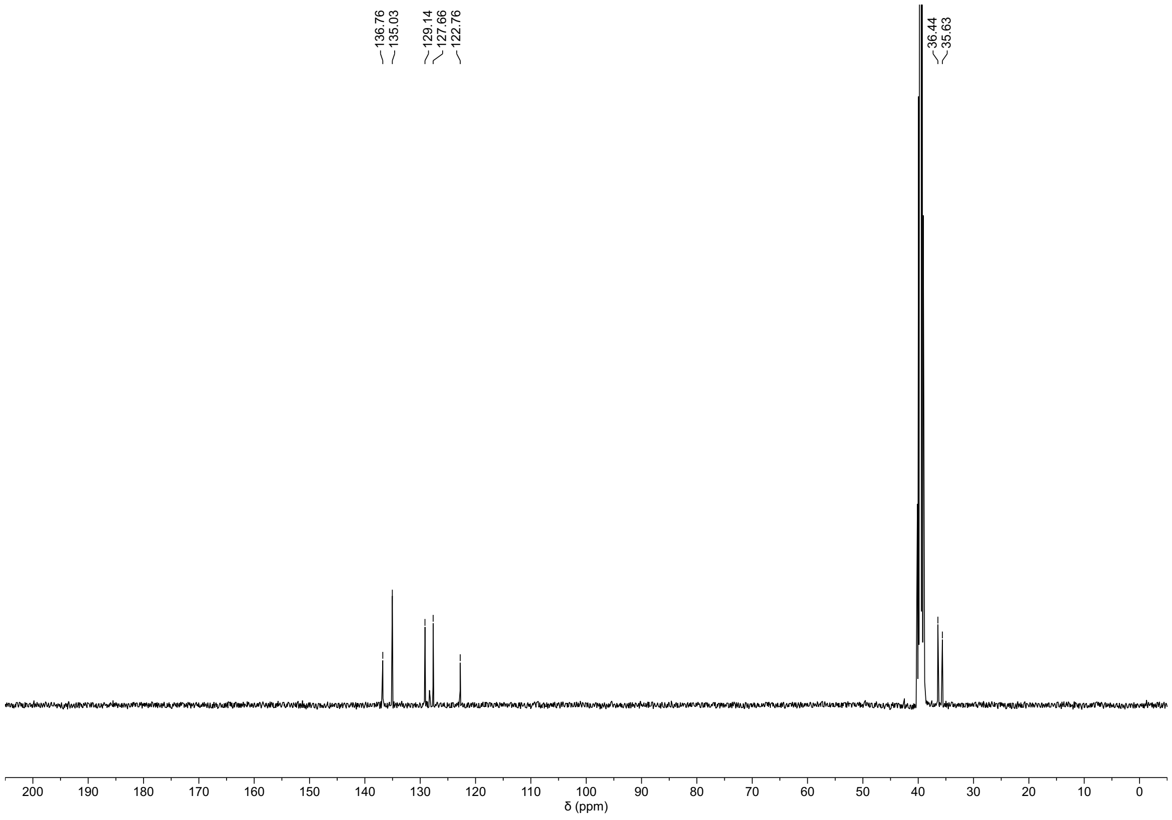

Figure 8. ^{13}C NMR – 5,8-Dibromo-2,11-dithia[3.3]paracyclophane (**3**), 101 MHz, DMSO-d_6.

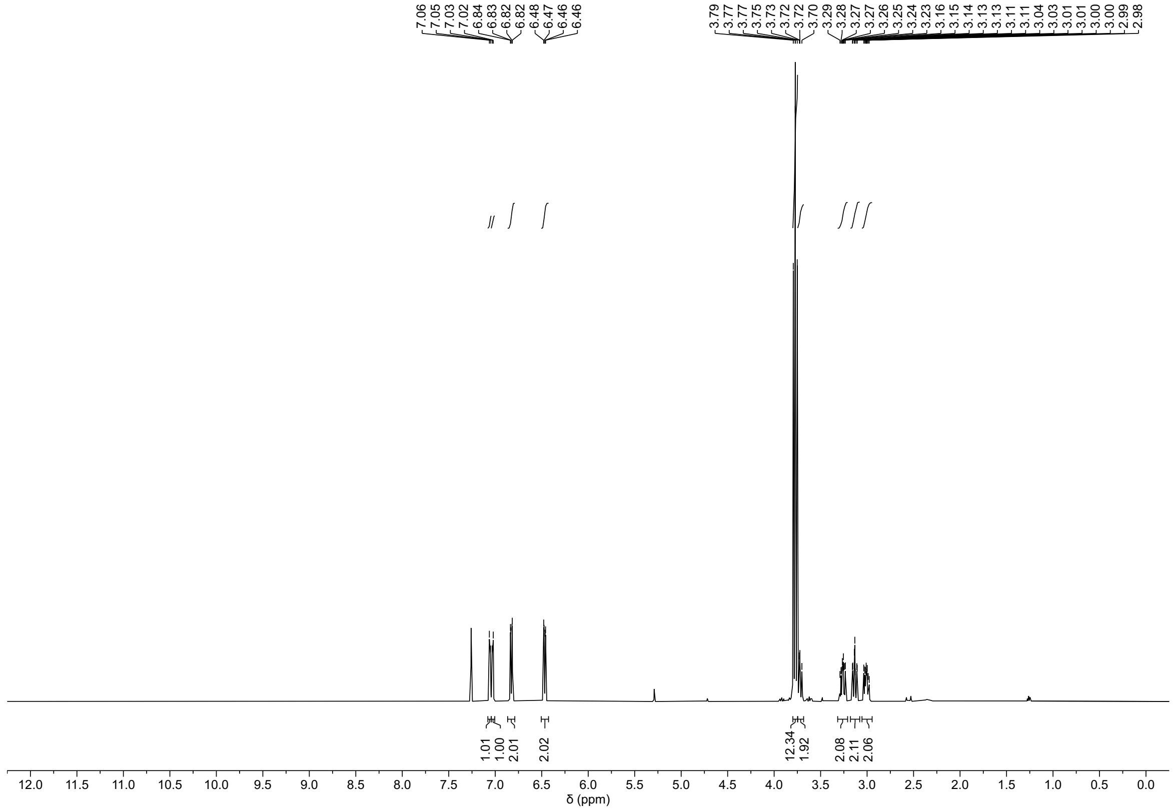

Figure 9. ^{1}H NMR – Tetramethyl[2.2]paracyclophane-2,7-diylbis(phosphonate) (**4**), 500 MHz, $CDCl_3$.

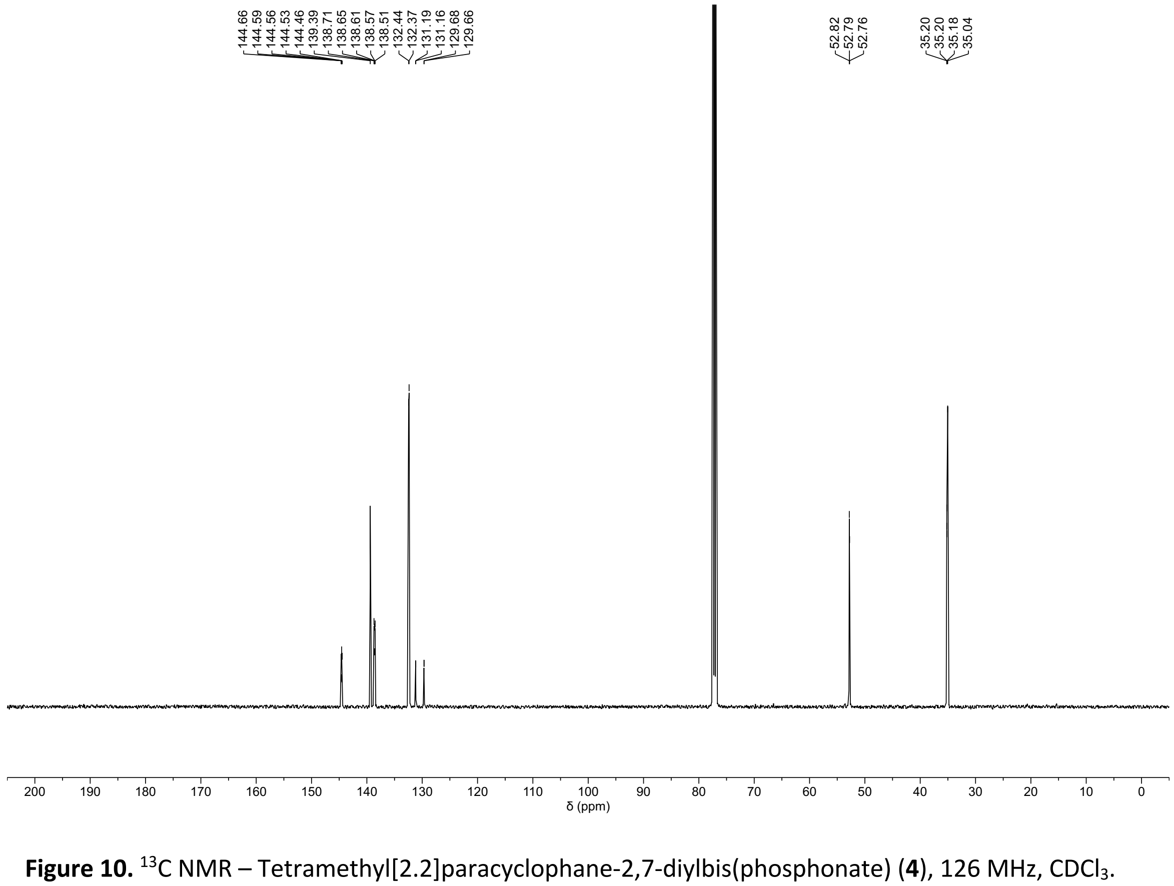

Figure 10. ^{13}C NMR – Tetramethyl[2.2]paracyclophane-2,7-diylbis(phosphonate) (**4**), 126 MHz, $CDCl_3$.

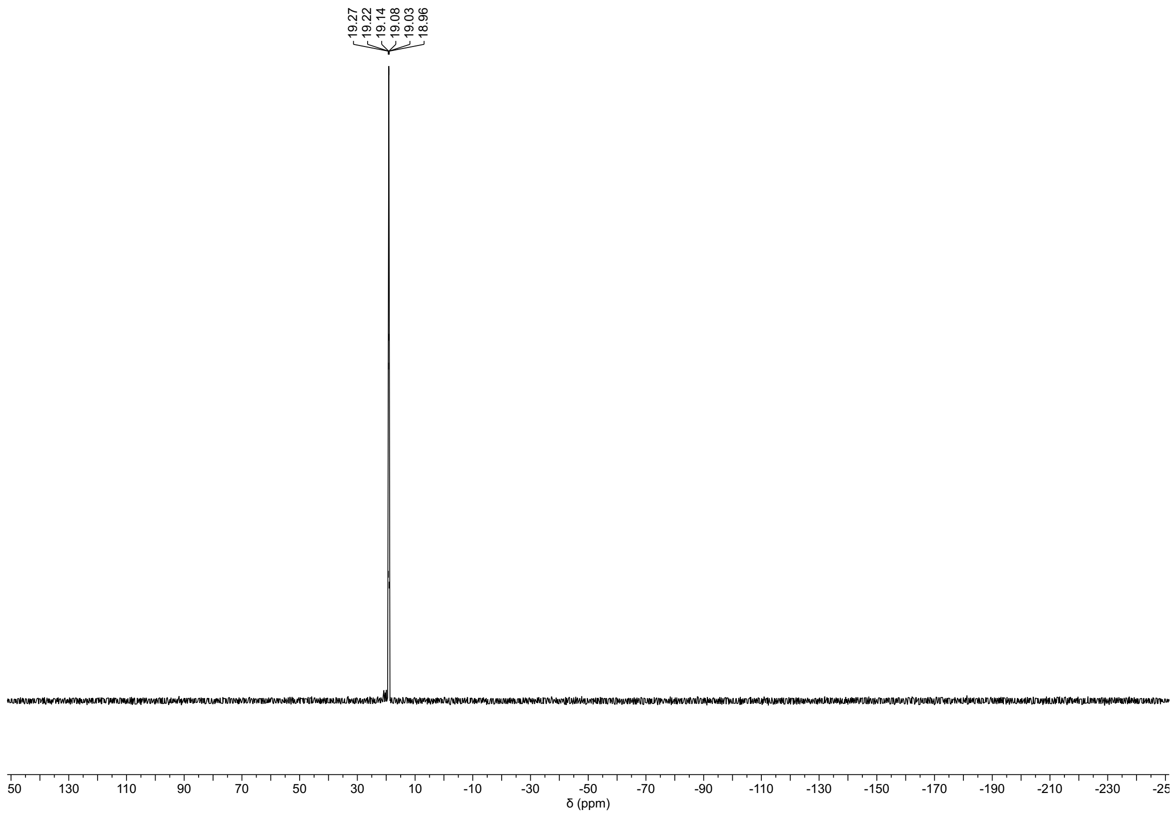

Figure 11. ^{31}P NMR – Tetramethyl[2.2]paracyclophane-2,7-diylbis(phosphonate) (**4**), 202 MHz, $CDCl_3$.

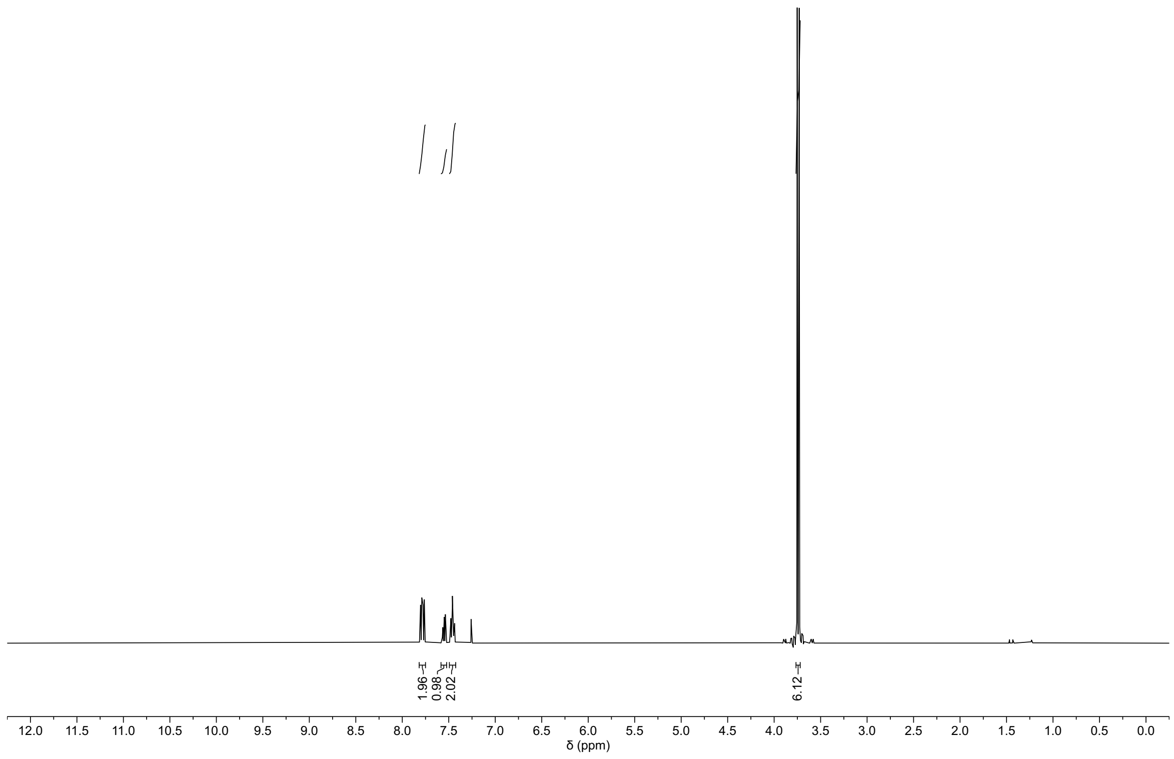

Figure 12. ^{1}H NMR – Dimethyl phenylphosphonate (**7a**), 500 MHz, $CDCl_3$.

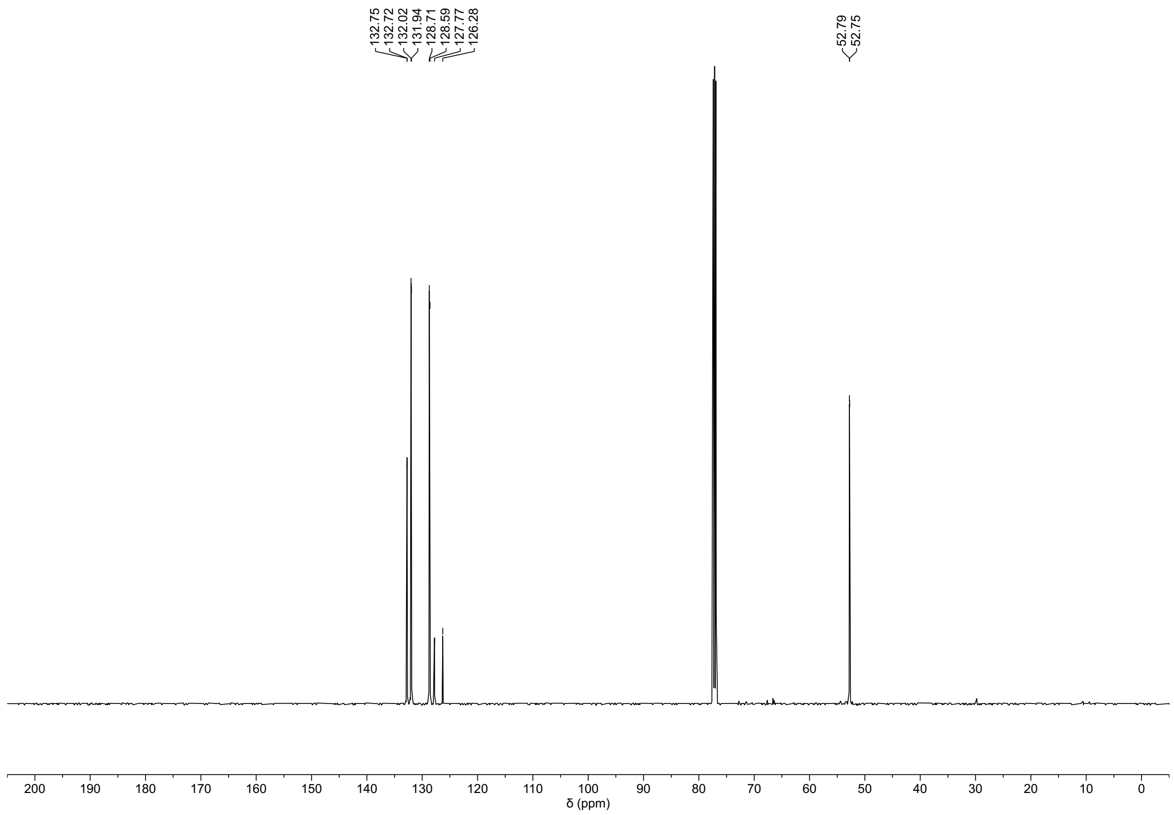

Figure 13. ^{13}C NMR – Dimethyl phenylphosphonate (**7a**), 126 MHz, $CDCl_3$.

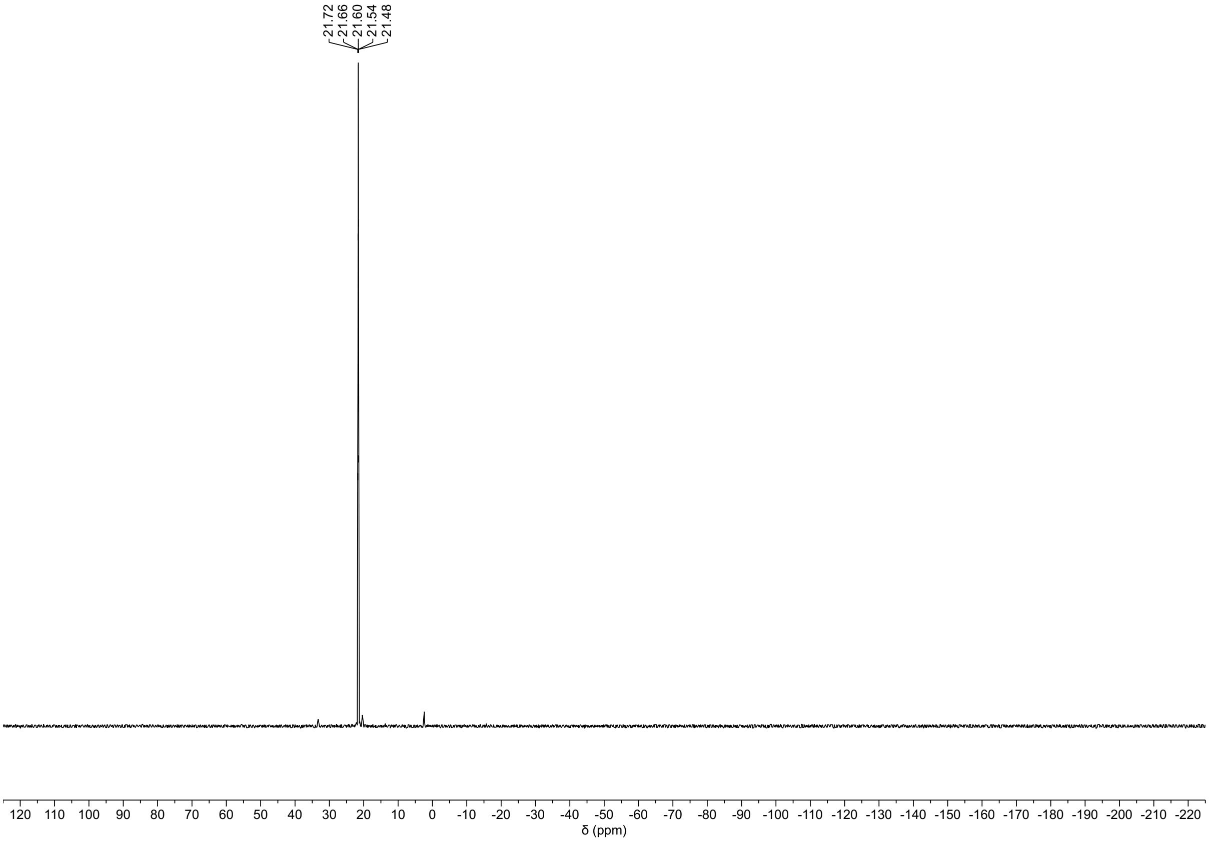

Figure 14. ^{31}P NMR – Dimethyl phenylphosphonate (**7a**), 202 MHz, $CDCl_3$.

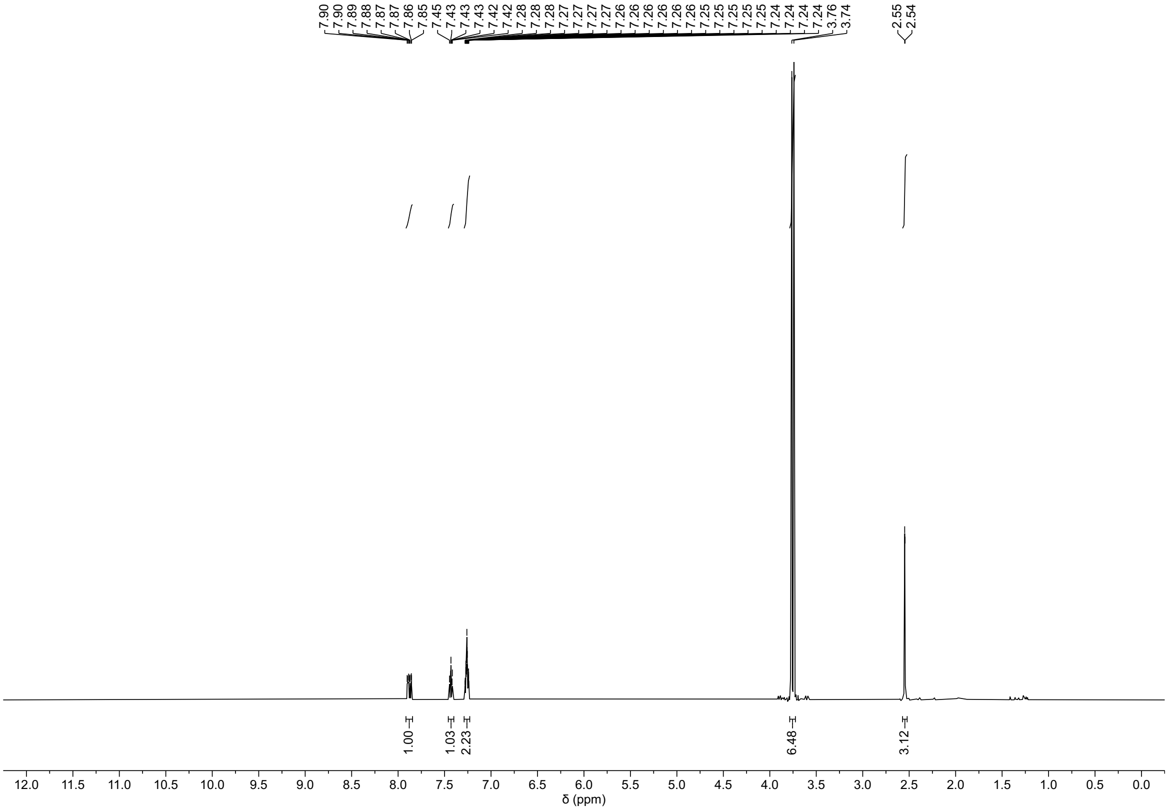

Figure 15. ^{1}H NMR – Dimethyl *o*-tolylphosphonate (**7b-*o***), 500 MHz, $CDCl_3$.

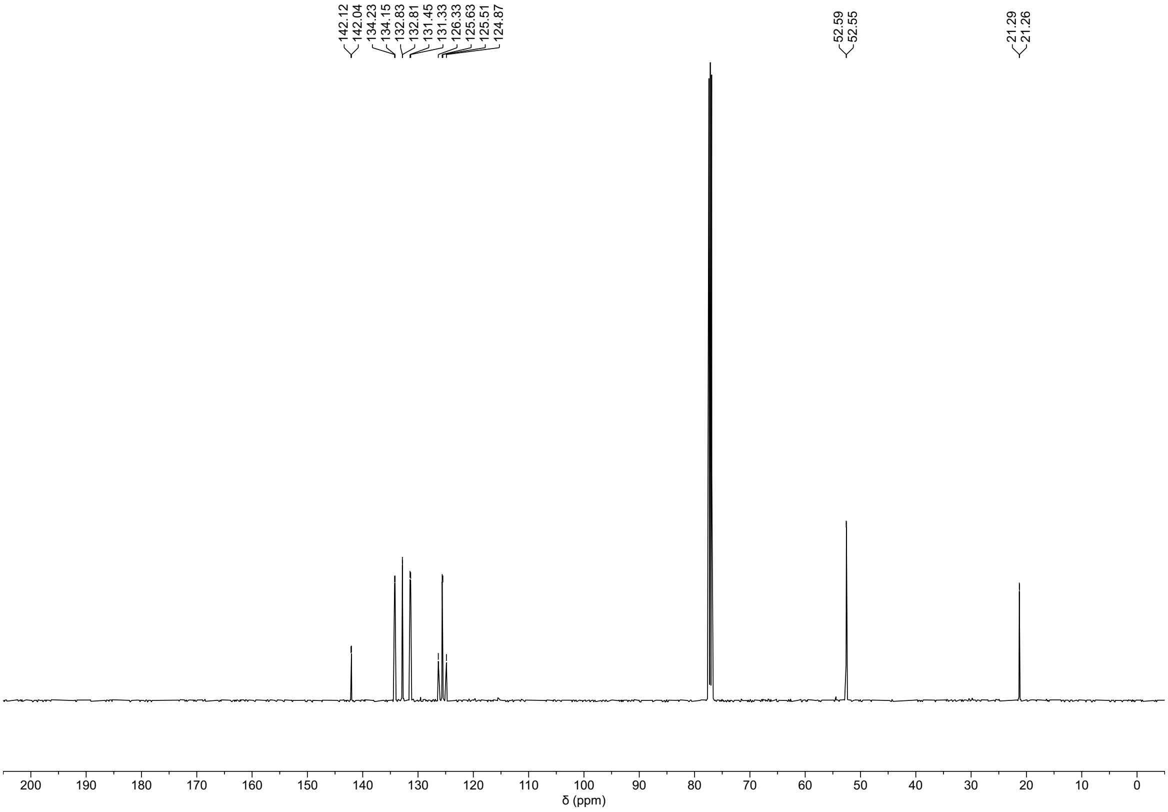

Figure 16. ^{13}C NMR – Dimethyl *o*-tolylphosphonate (**7b-*o***), 126 MHz, $CDCl_3$.

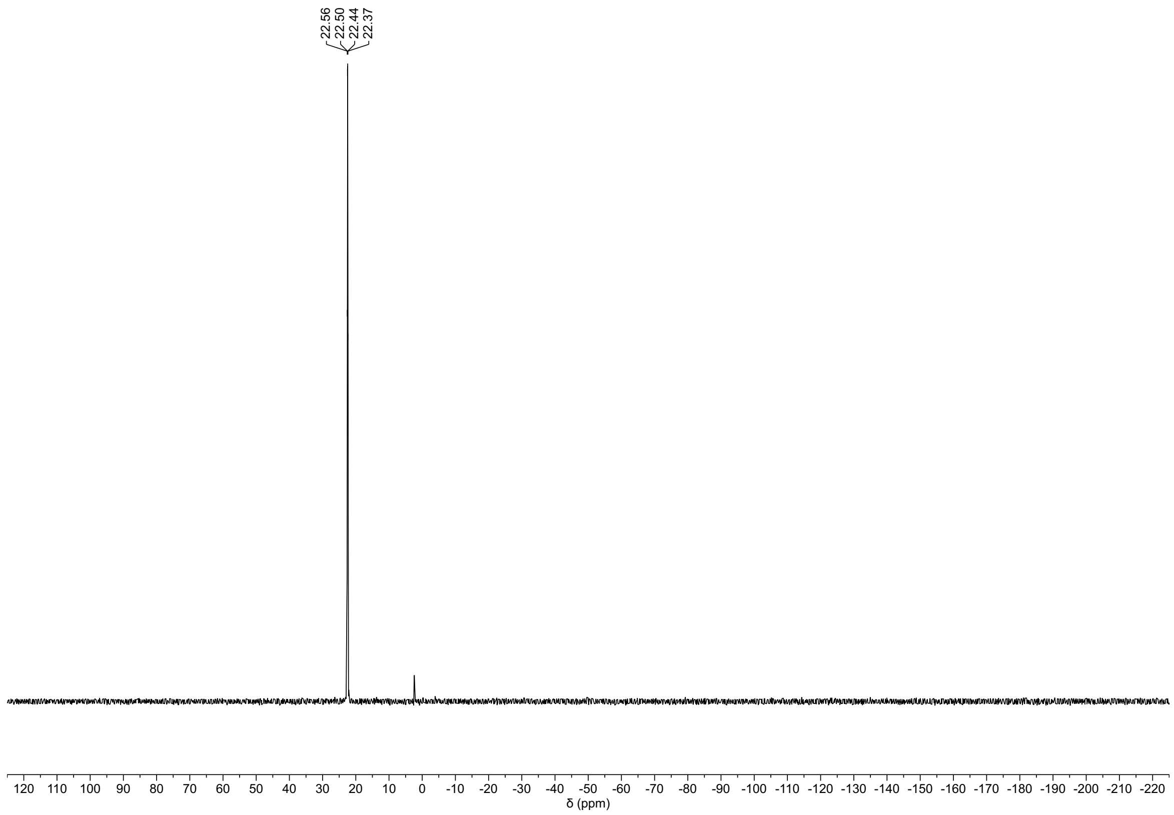

Figure 17. ^{31}P NMR – Dimethyl *o*-tolylphosphonate (**7b-*o***), 202 MHz, $CDCl_3$.

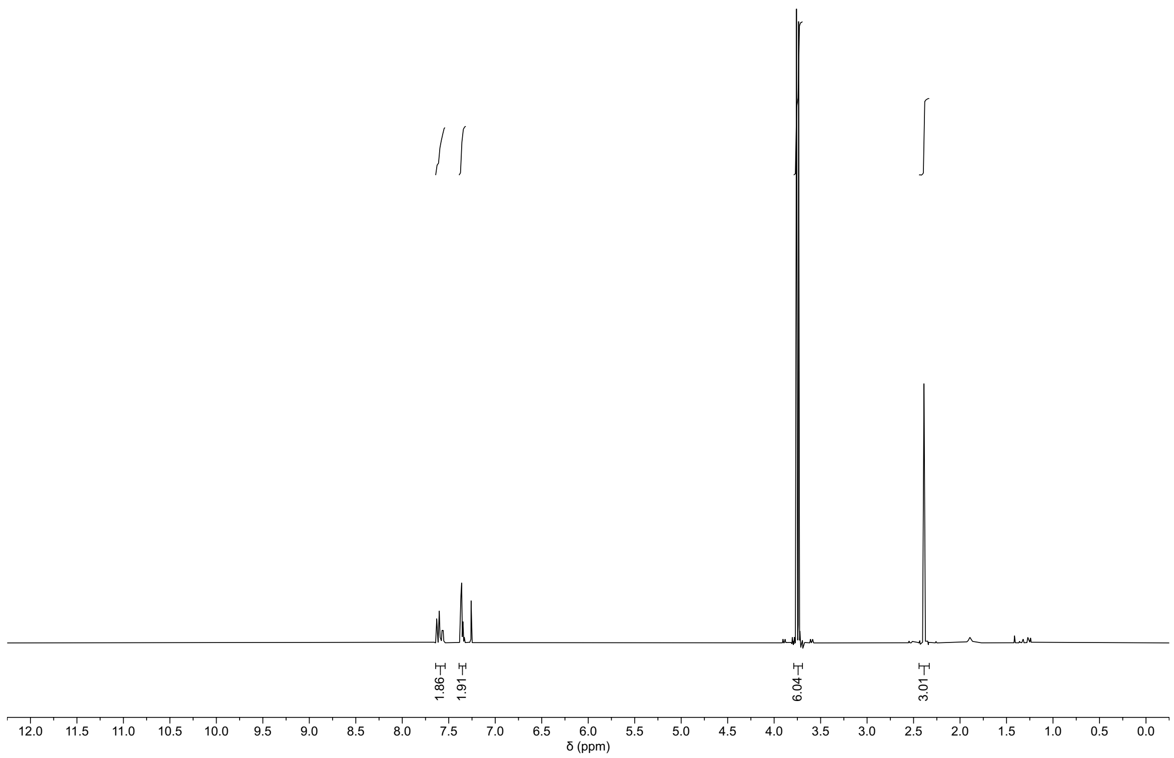

Figure 18. ^{1}H NMR – Dimethyl *m*-tolylphosphonate (**7b-*m***), 500 MHz, $CDCl_3$.

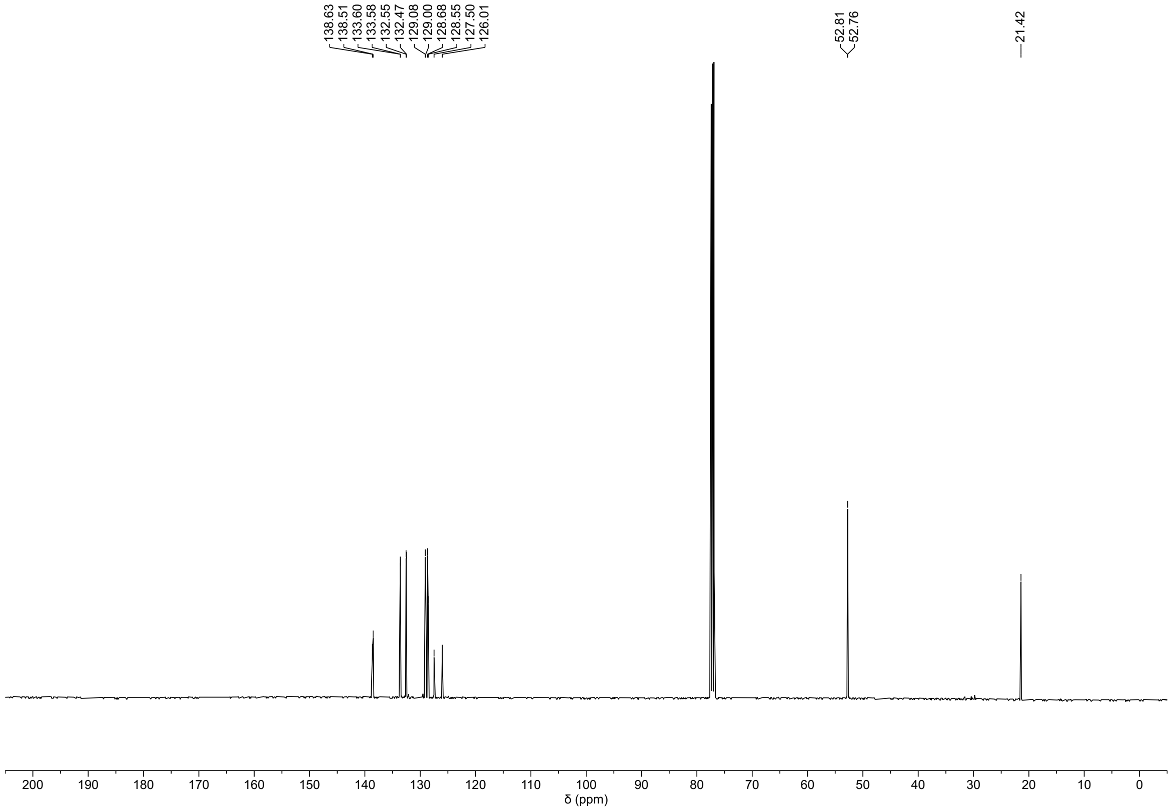

Figure 19. ^{13}C NMR – Dimethyl *m*-tolylphosphonate (**7b-*m***), 126 MHz, $CDCl_3$.

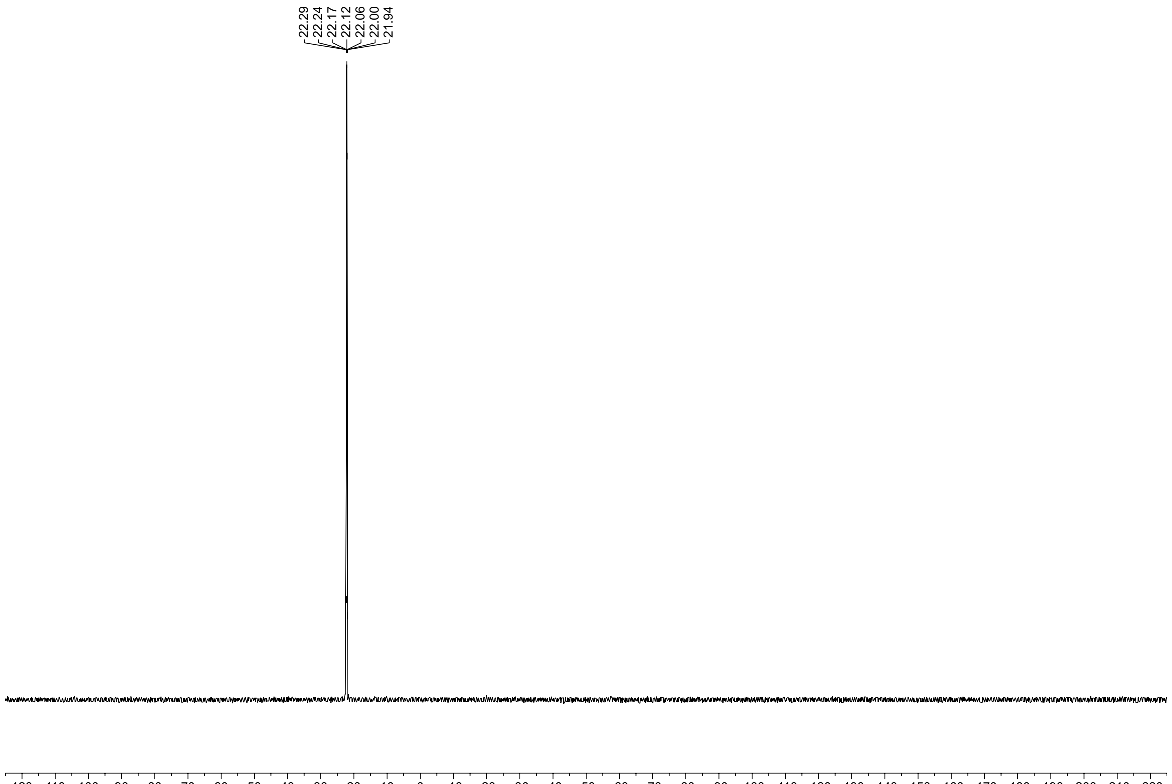

Figure 20. ^{31}P NMR – Dimethyl *m*-tolylphosphonate (**7b-*m***), 202 MHz, $CDCl_3$.

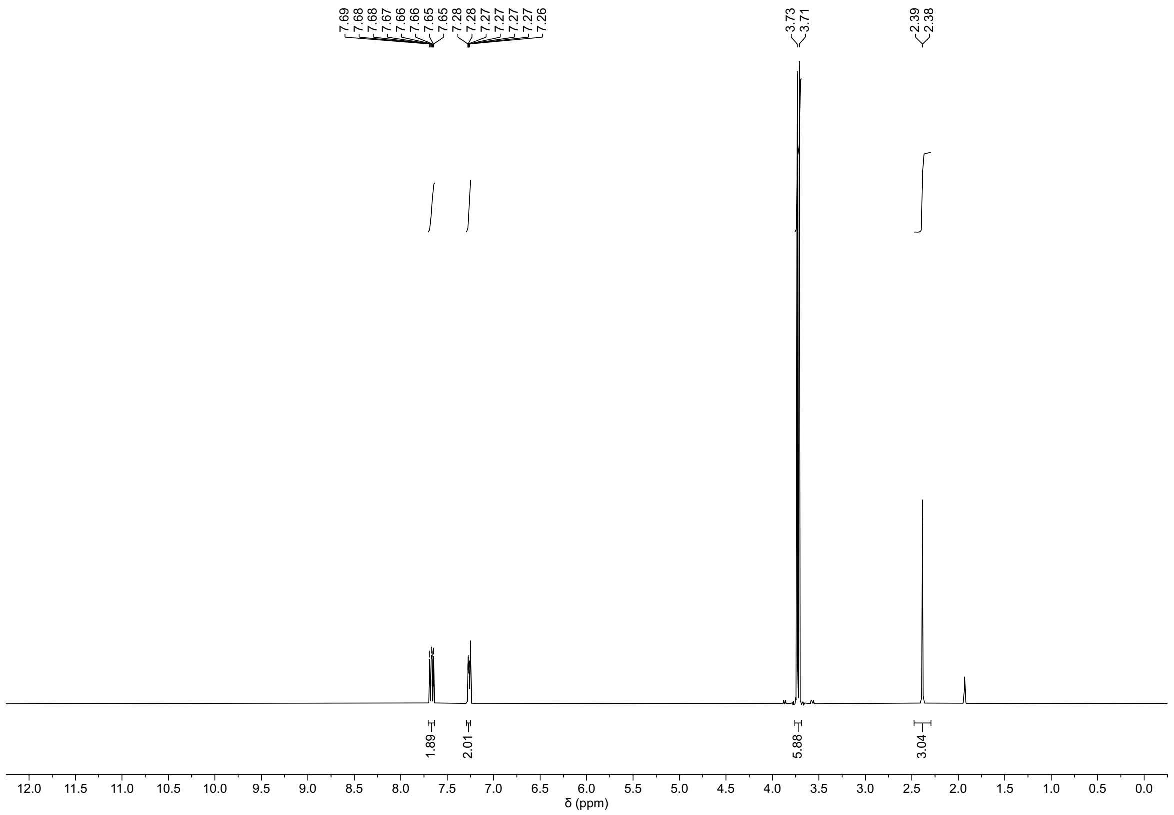

Figure 21. ^{1}H NMR – Dimethyl *p*-tolylphosphonate (**7b-*p***), 500 MHz, $CDCl_3$.

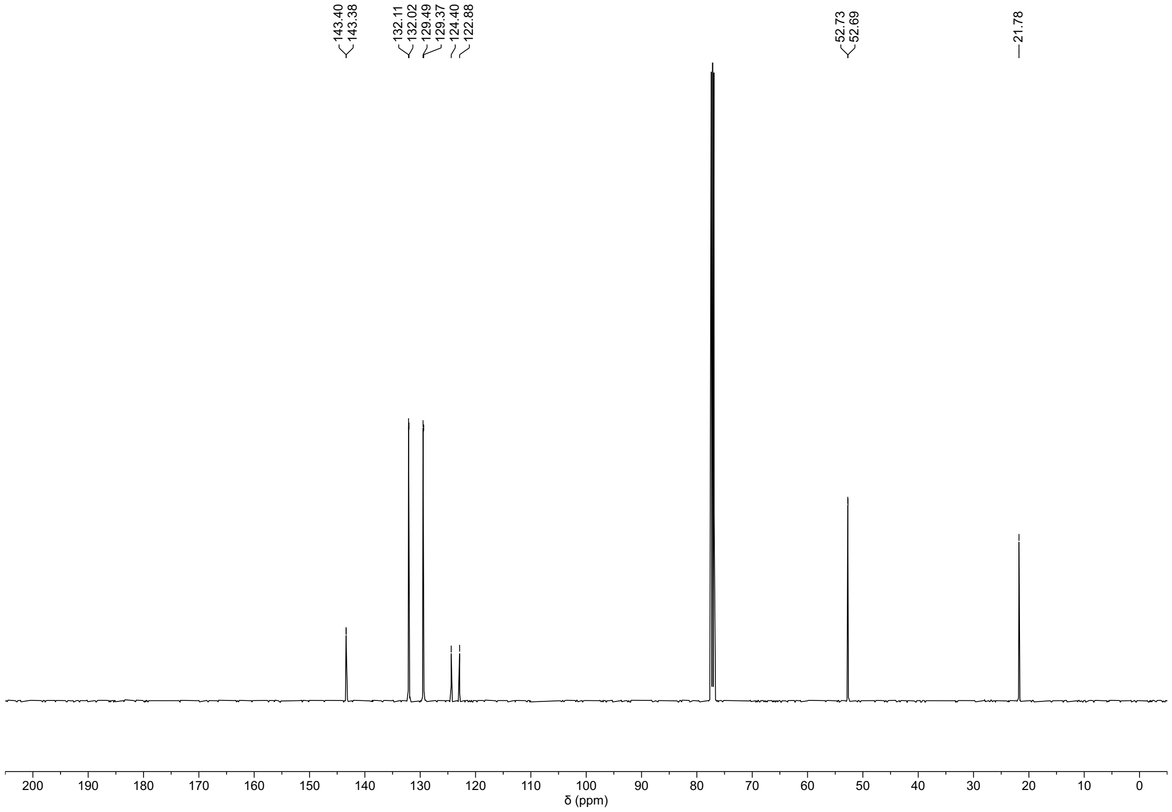

Figure 22. ^{13}C NMR – Dimethyl *p*-tolylphosphonate (**7b-*p***), 126 MHz, $CDCl_3$.

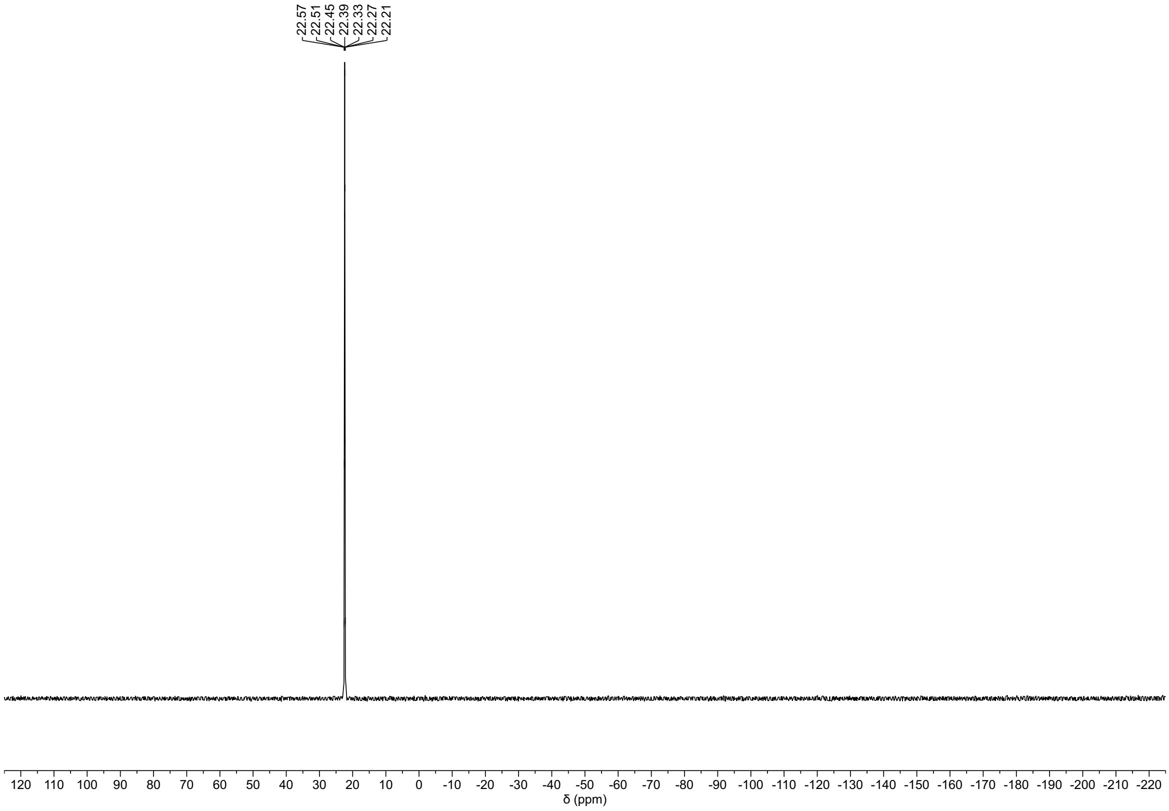

Figure 23. ^{31}P NMR – Dimethyl *p*-tolylphosphonate (**7b-*p***), 202 MHz, $CDCl_3$.

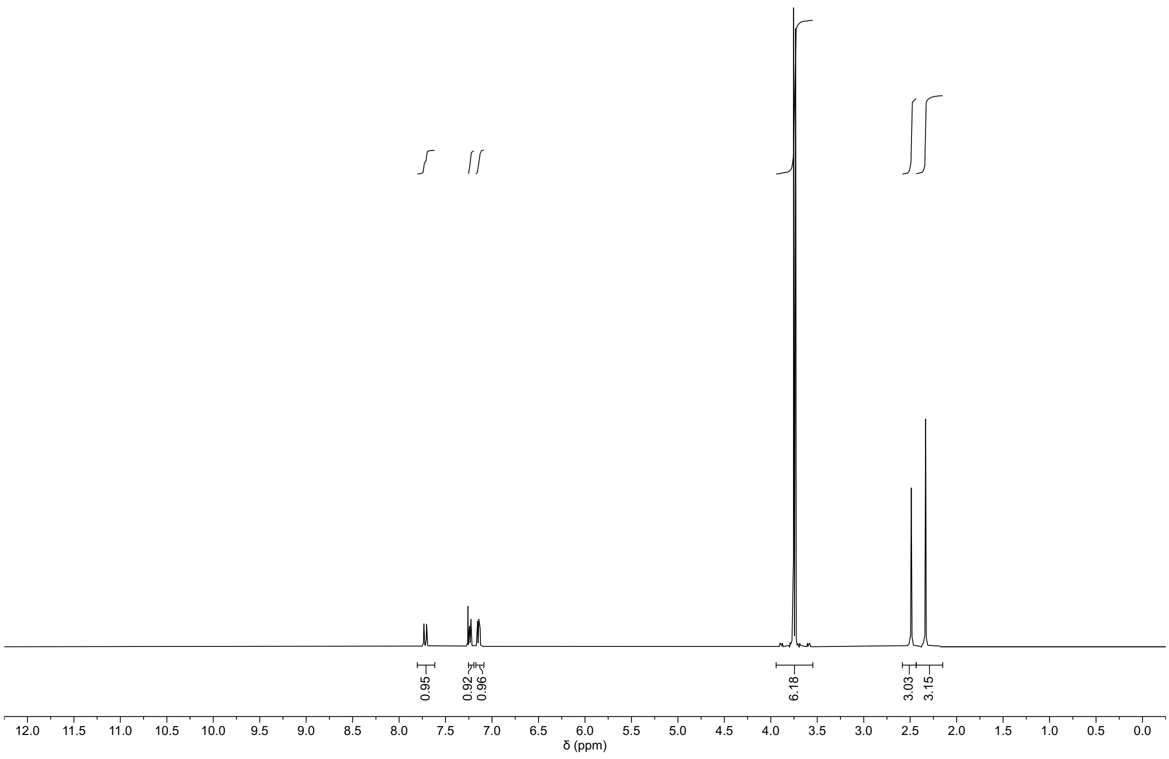

Figure 24. ^{1}H NMR – Dimethyl (2,5-dimethylphenyl)phosphonate (**7c**), 500 MHz, $CDCl_3$.

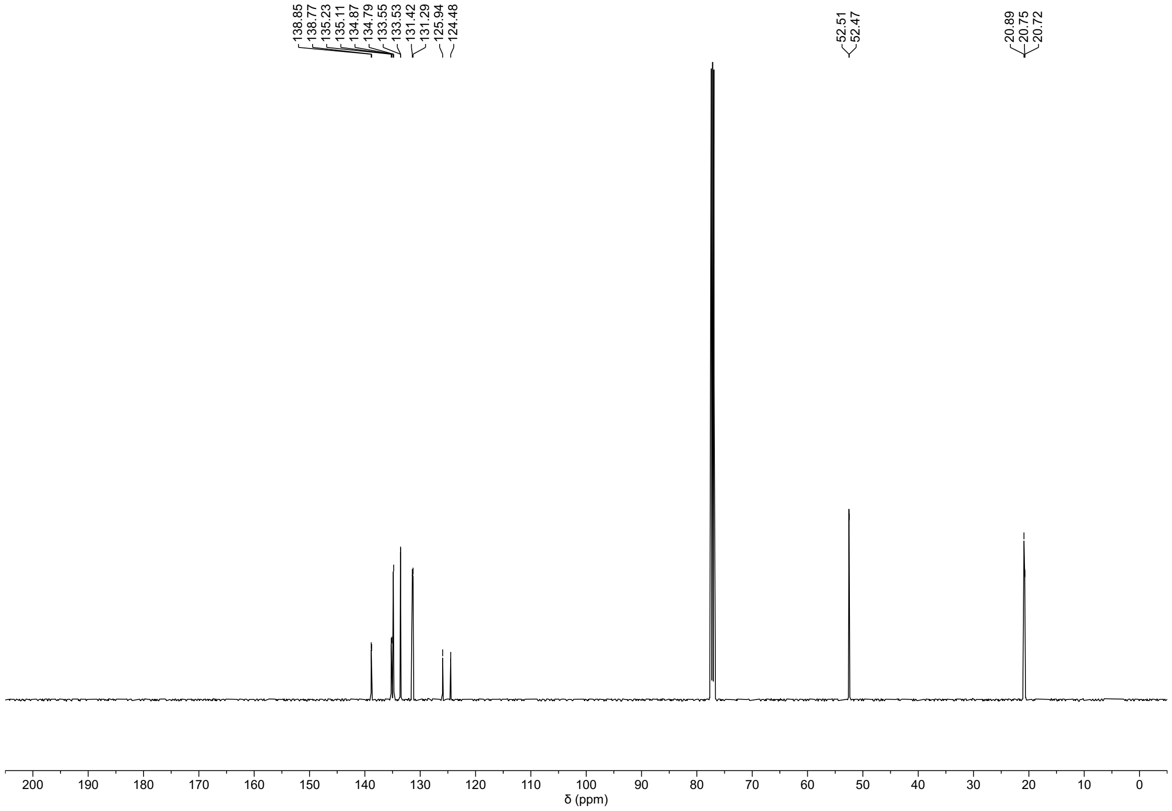

Figure 25. ^{13}C NMR – Dimethyl (2,5-dimethylphenyl)phosphonate (**7c**), 126 MHz, $CDCl_3$.

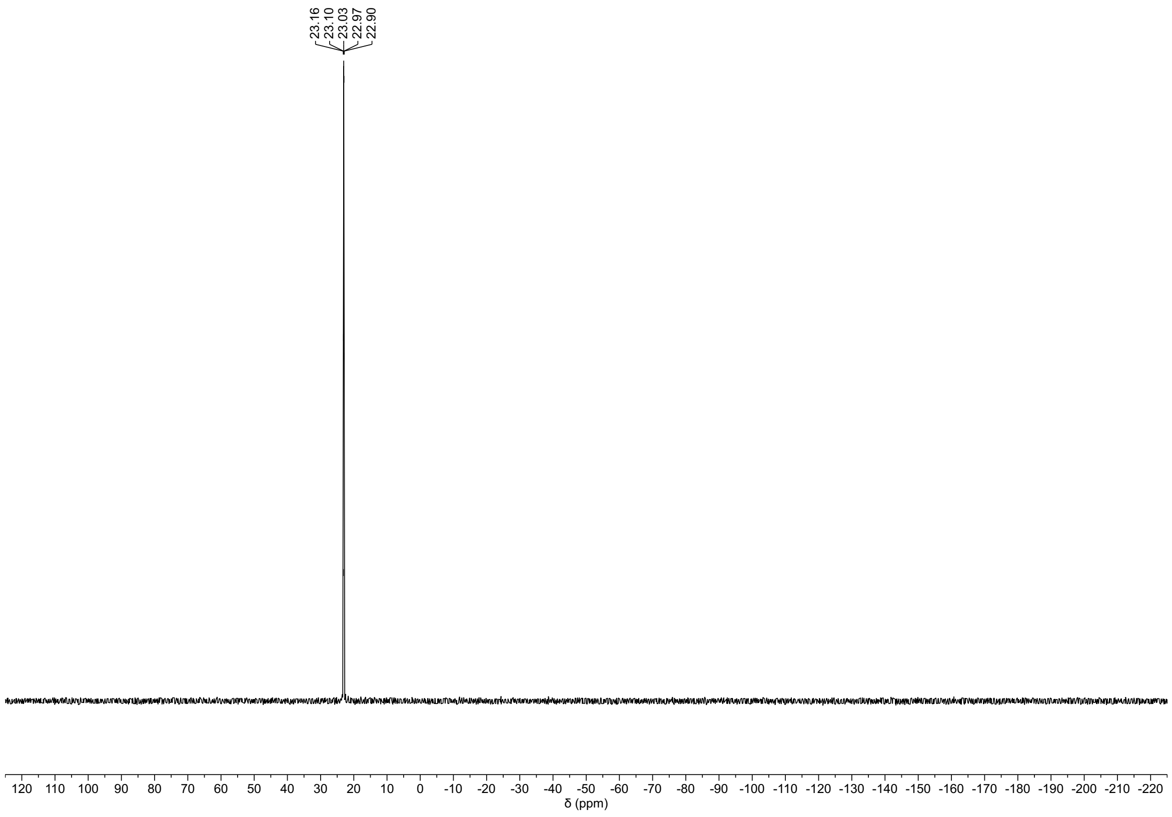

Figure 26. ^{31}P NMR – Dimethyl (2,5-dimethylphenyl)phosphonate (**7c**), 202 MHz, $CDCl_3$.

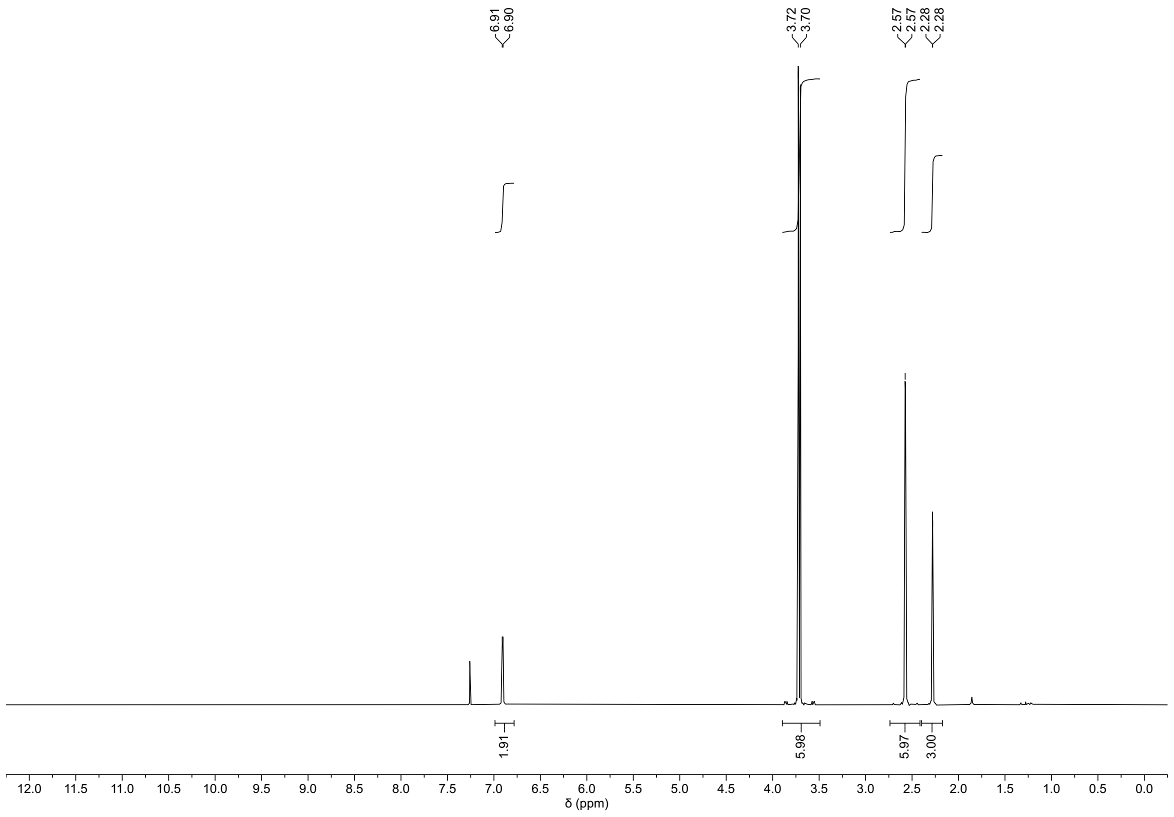

Figure 27. ^{1}H NMR – Dimethyl (2,4,6-trimethylphenyl)phosphonate (**7d**), 500 MHz, $CDCl_3$.

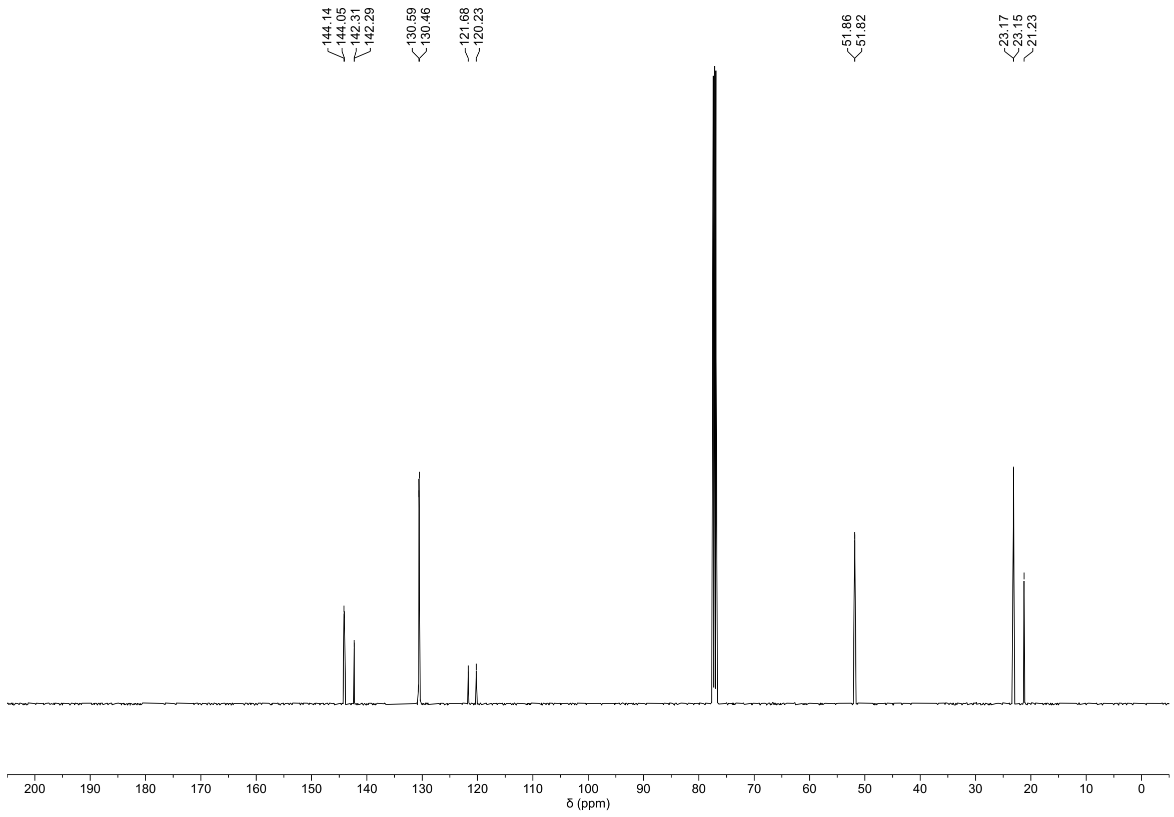

Figure 28. ^{13}C NMR – Dimethyl (2,4,6-trimethylphenyl)phosphonate (**7d**), 126 MHz, $CDCl_3$.

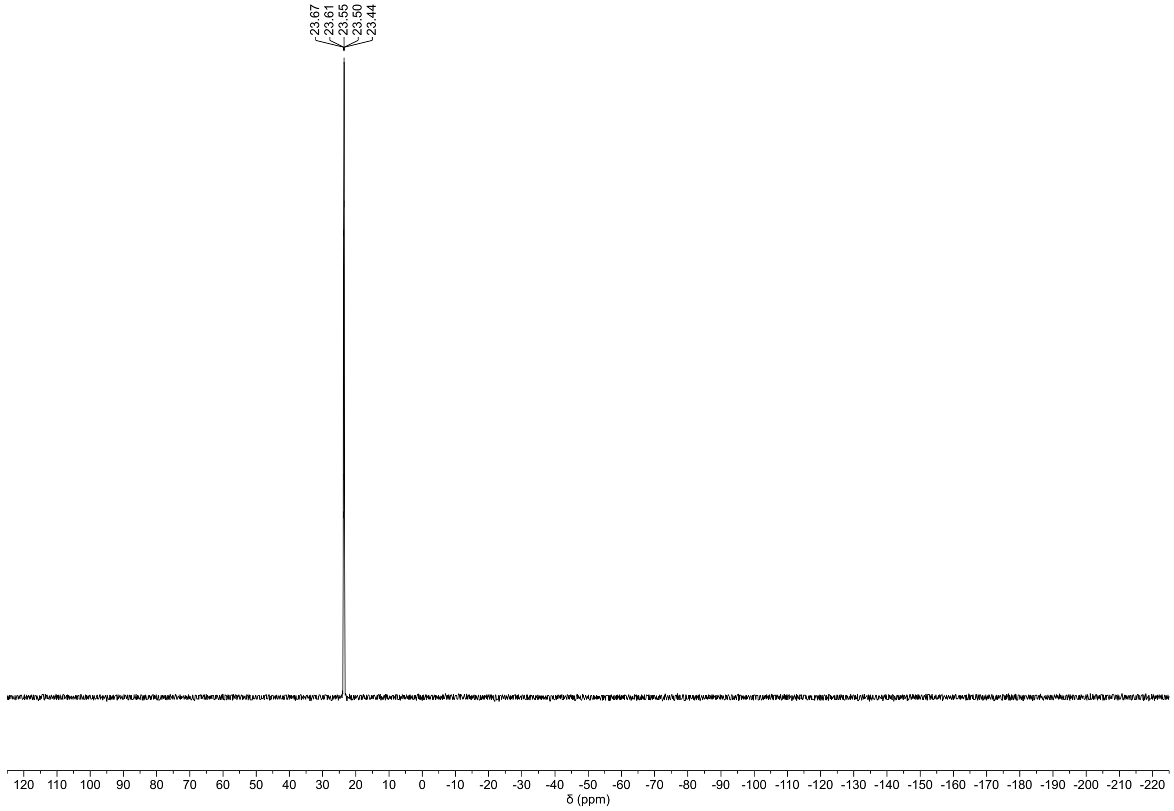

Figure 29. ^{31}P NMR – Dimethyl (2,4,6-trimethylphenyl)phosphonate (**7d**), 202 MHz, $CDCl_3$.

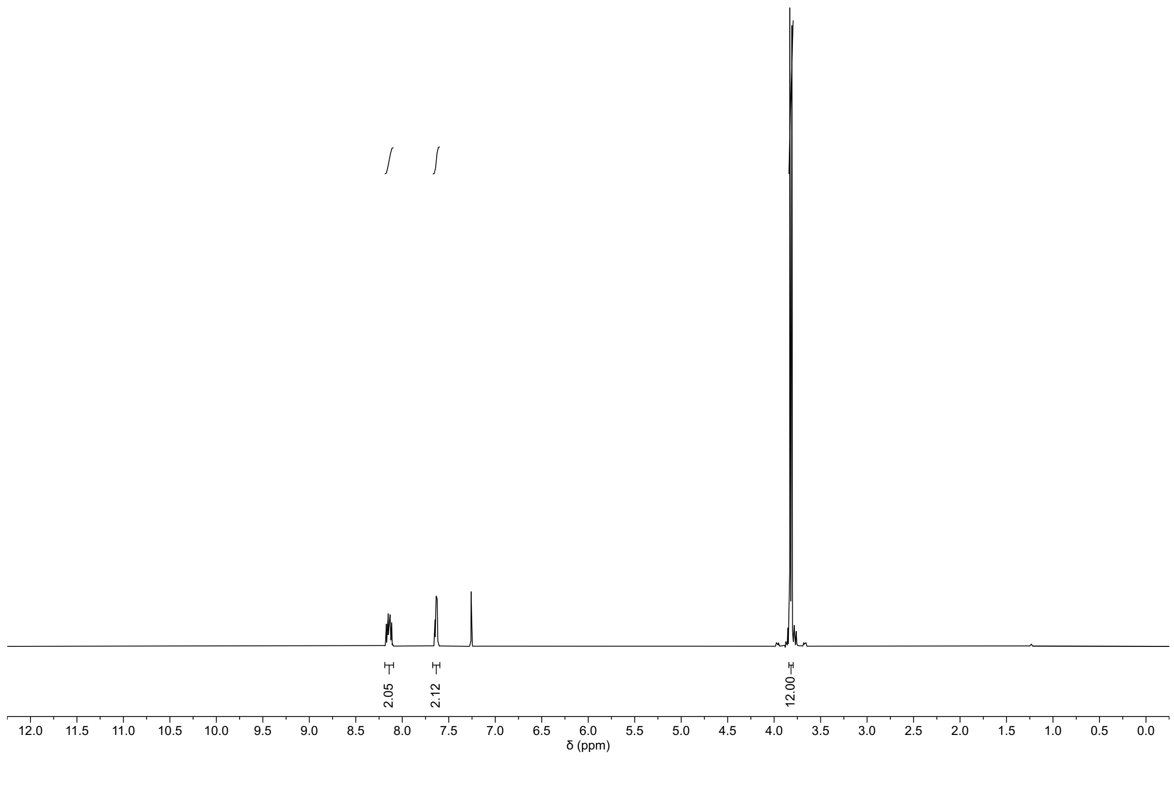

Figure 30. ^{1}H NMR – Tetramethyl 1,2-phenylenebis(phosphonate) (**7e'-*o***), 500 MHz, $CDCl_3$.

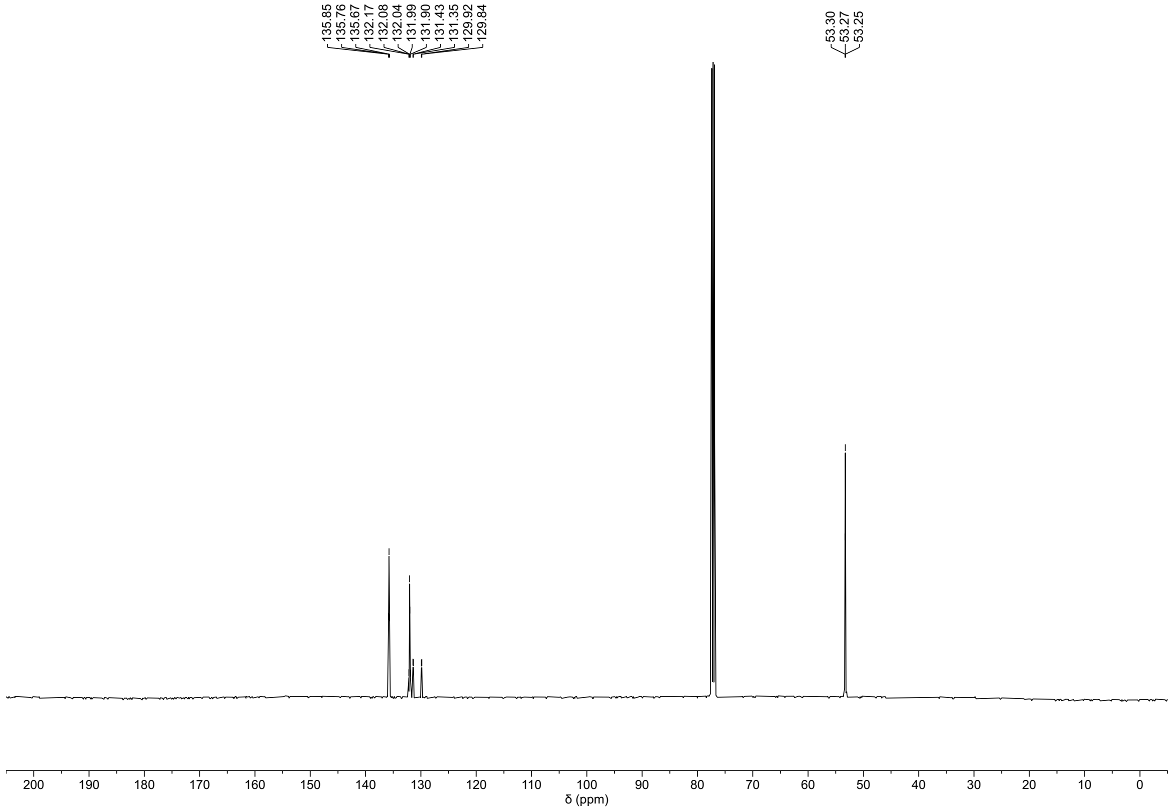

Figure 31. ^{13}C NMR – Tetramethyl 1,2-phenylenebis(phosphonate) (**7e'-*o***), 126 MHz, $CDCl_3$.

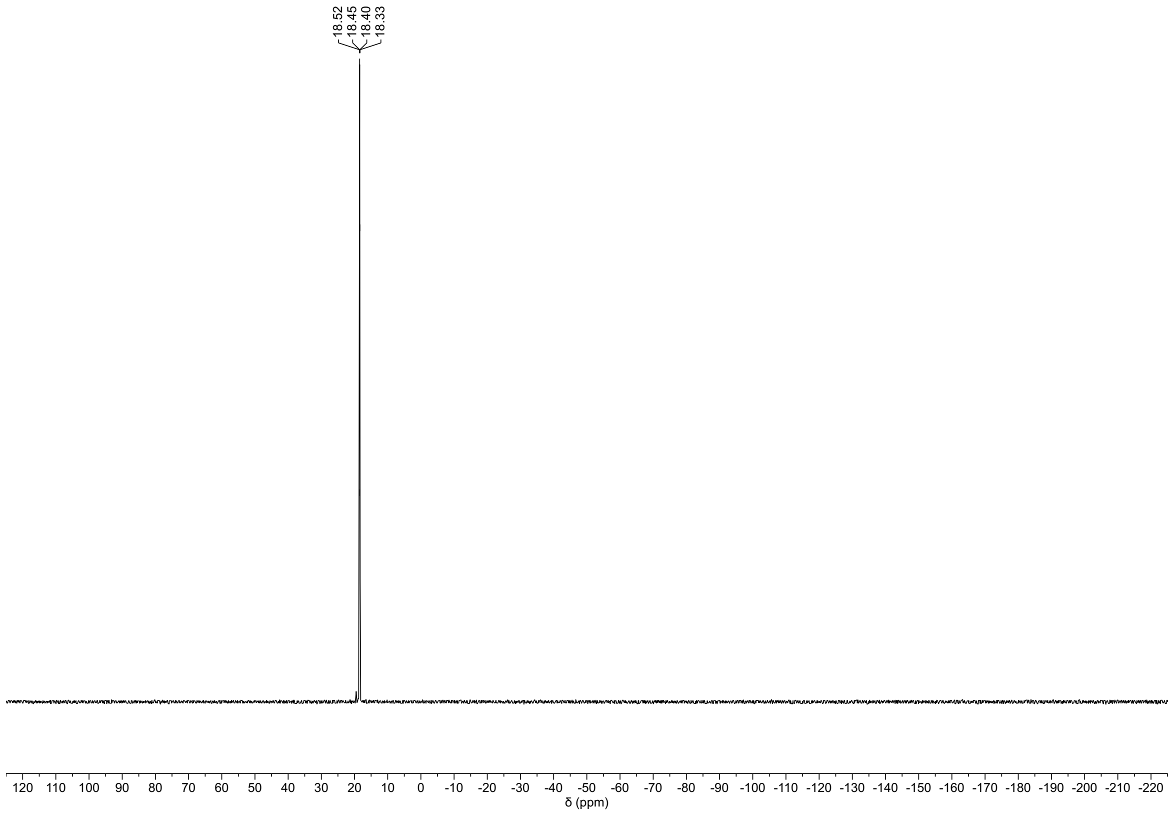

Figure 32. ^{31}P NMR – Tetramethyl 1,2-phenylenebis(phosphonate) (**7e'-*o***), 202 MHz, $CDCl_3$.

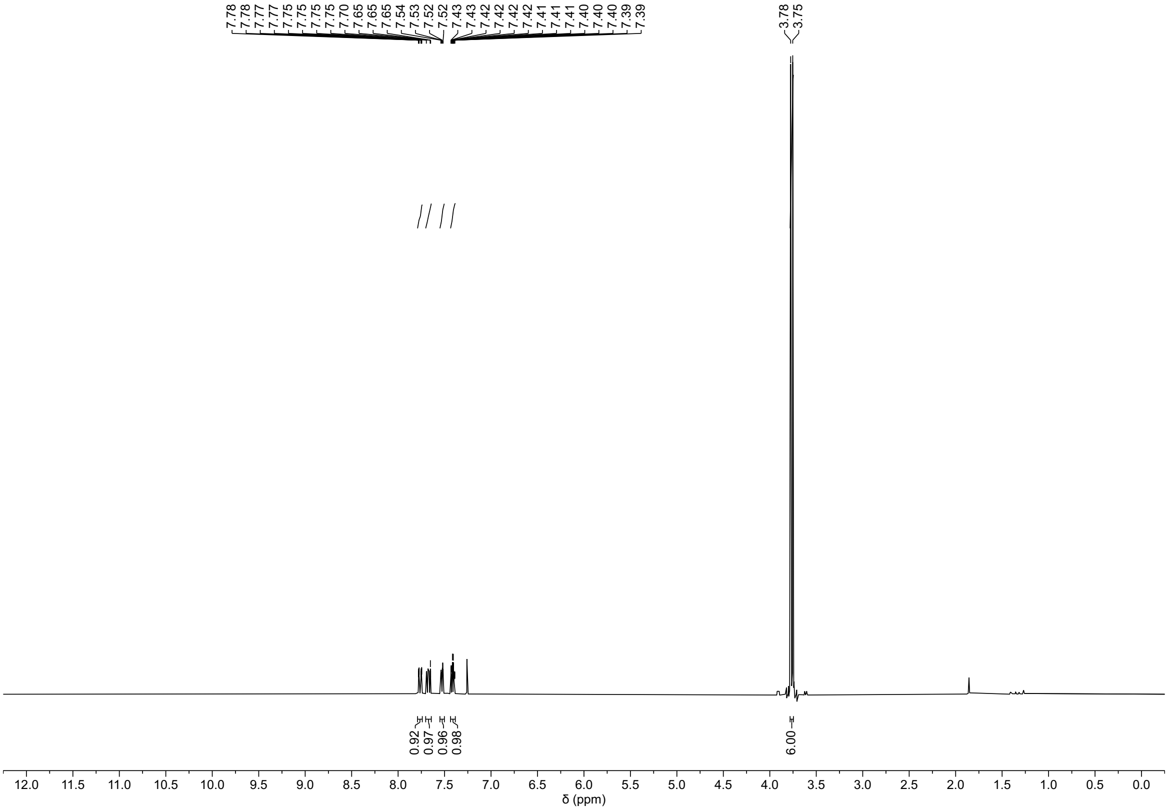

Figure 33. ^{1}H NMR – Dimethyl (3-chlorophenyl)phosphonate (**7e-*m***), 500 MHz, $CDCl_3$.

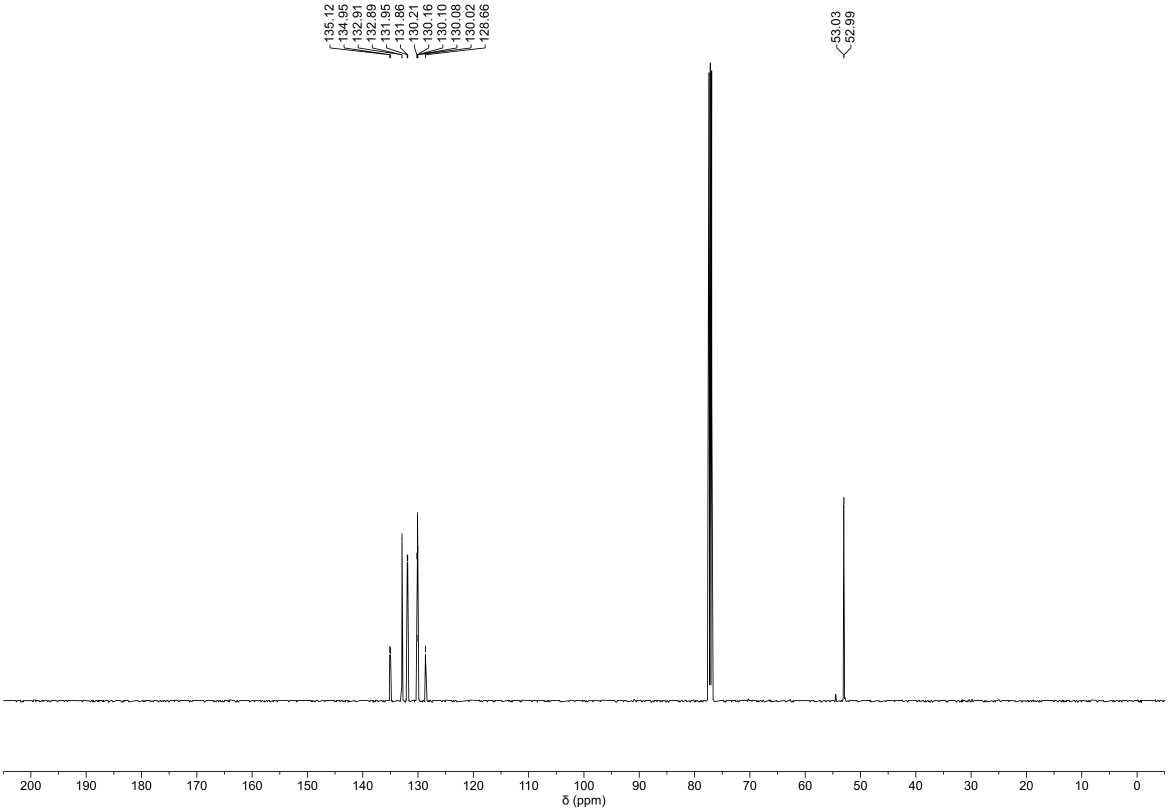

Figure 34. ^{13}C NMR – Dimethyl (3-chlorophenyl)phosphonate (**7e-*m***), 126 MHz, $CDCl_3$.

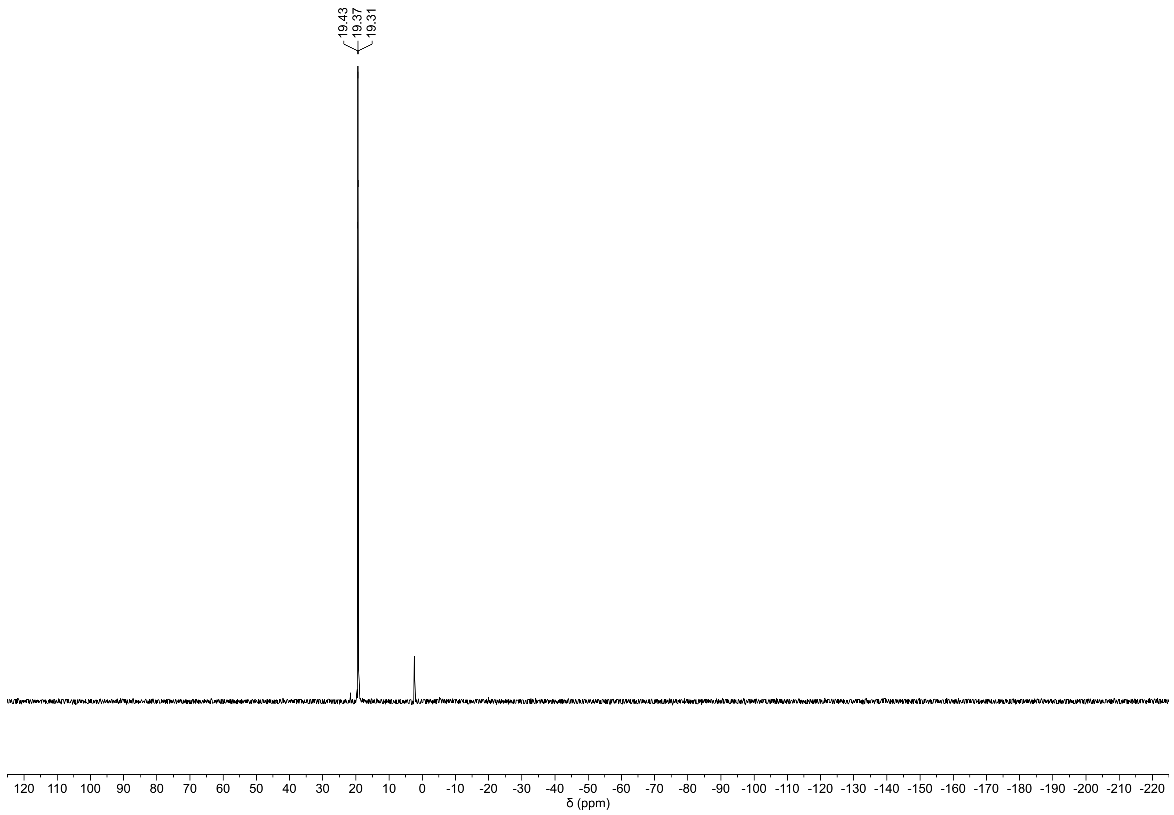

Figure 35. ^{31}P NMR – Dimethyl (3-chlorophenyl)phosphonate (**7e-*m***), 202 MHz, $CDCl_3$.

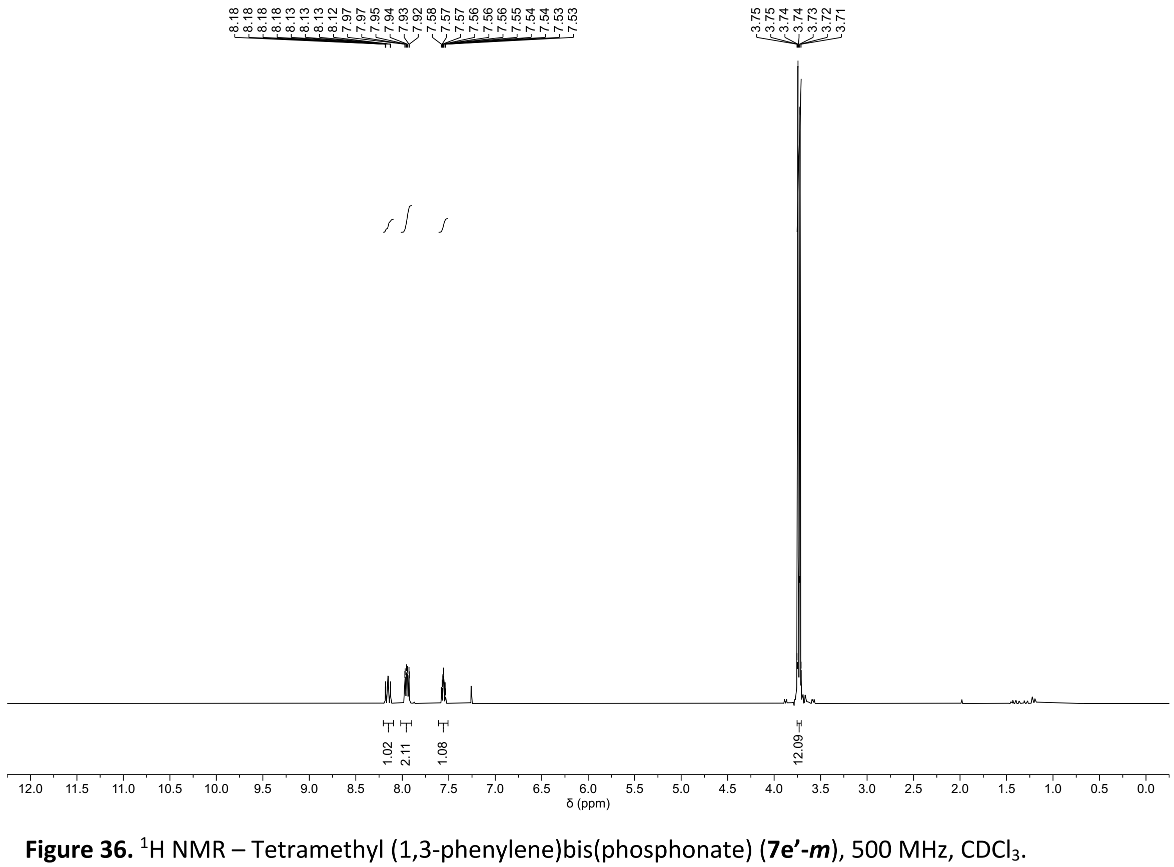

Figure 36. ^{1}H NMR – Tetramethyl (1,3-phenylene)bis(phosphonate) (**7e'-*m***), 500 MHz, $CDCl_3$.

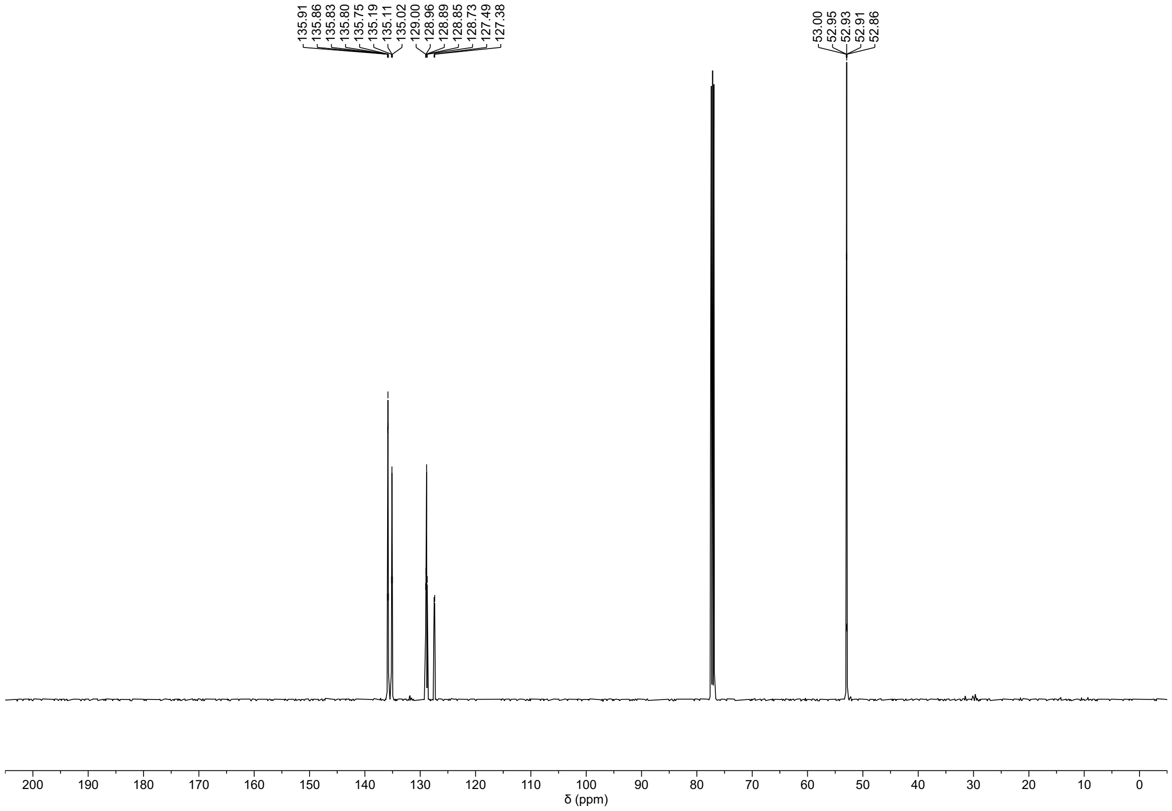

Figure 37. ^{13}C NMR – Tetramethyl (1,3-phenylene)bis(phosphonate) (**7e'-*m***), 126 MHz, $CDCl_3$.

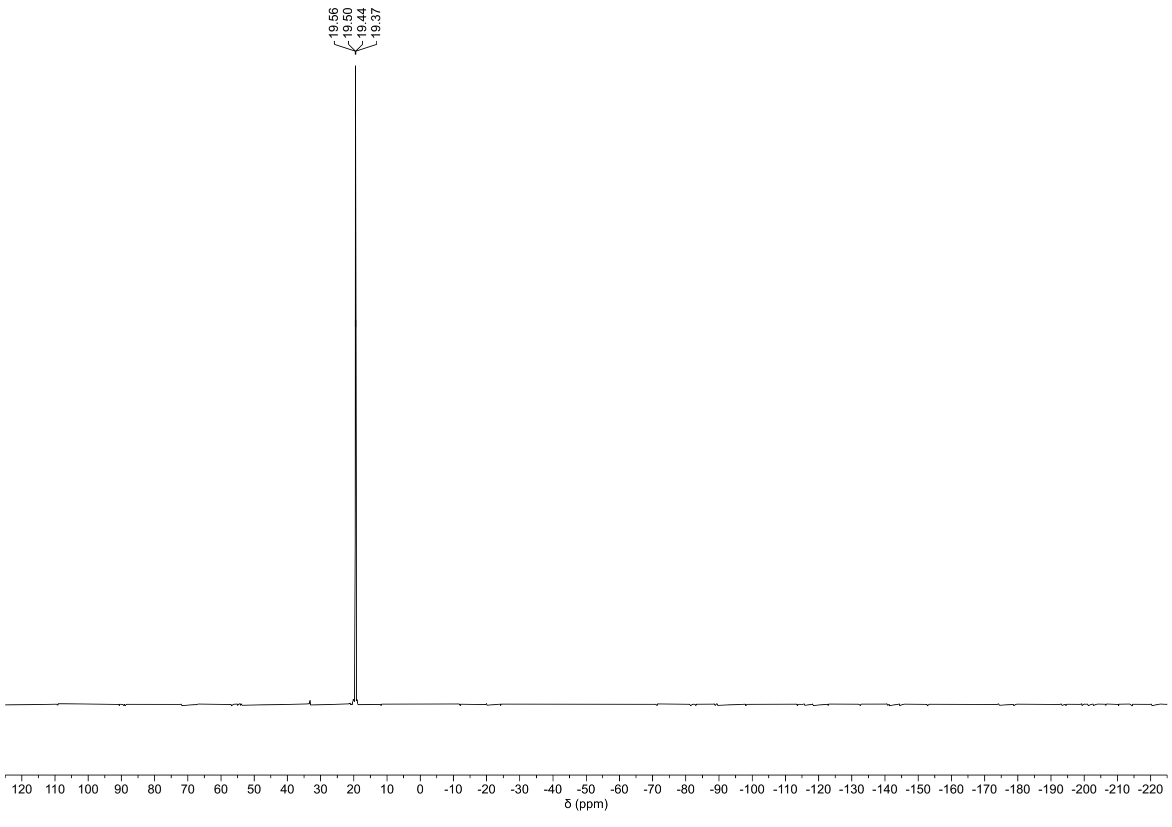

Figure 38. ^{31}P NMR – Tetramethyl (1,3-phenylene)bis(phosphonate) (**7e'-*m***), 202 MHz, $CDCl_3$.

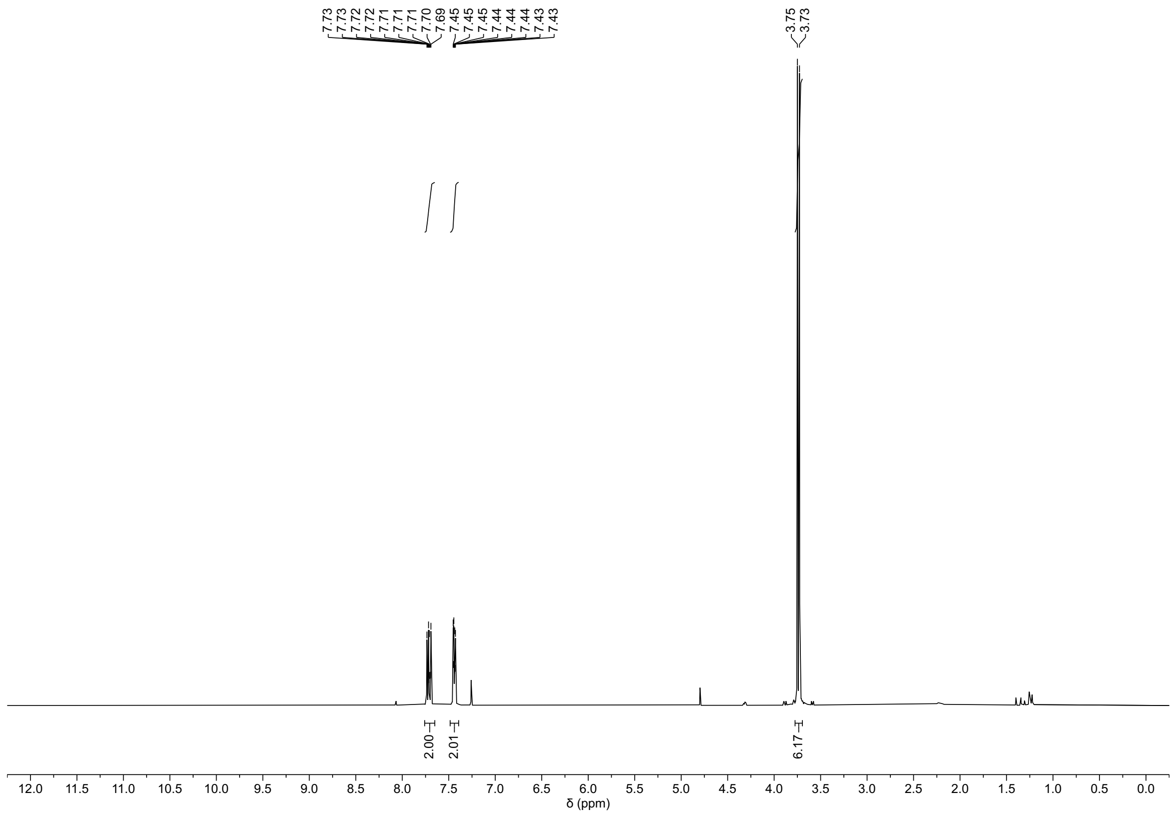

Figure 39. ^{1}H NMR – Dimethyl (4-chlorophenyl)phosphonate (**7e-*p***), 500 MHz, $CDCl_3$.

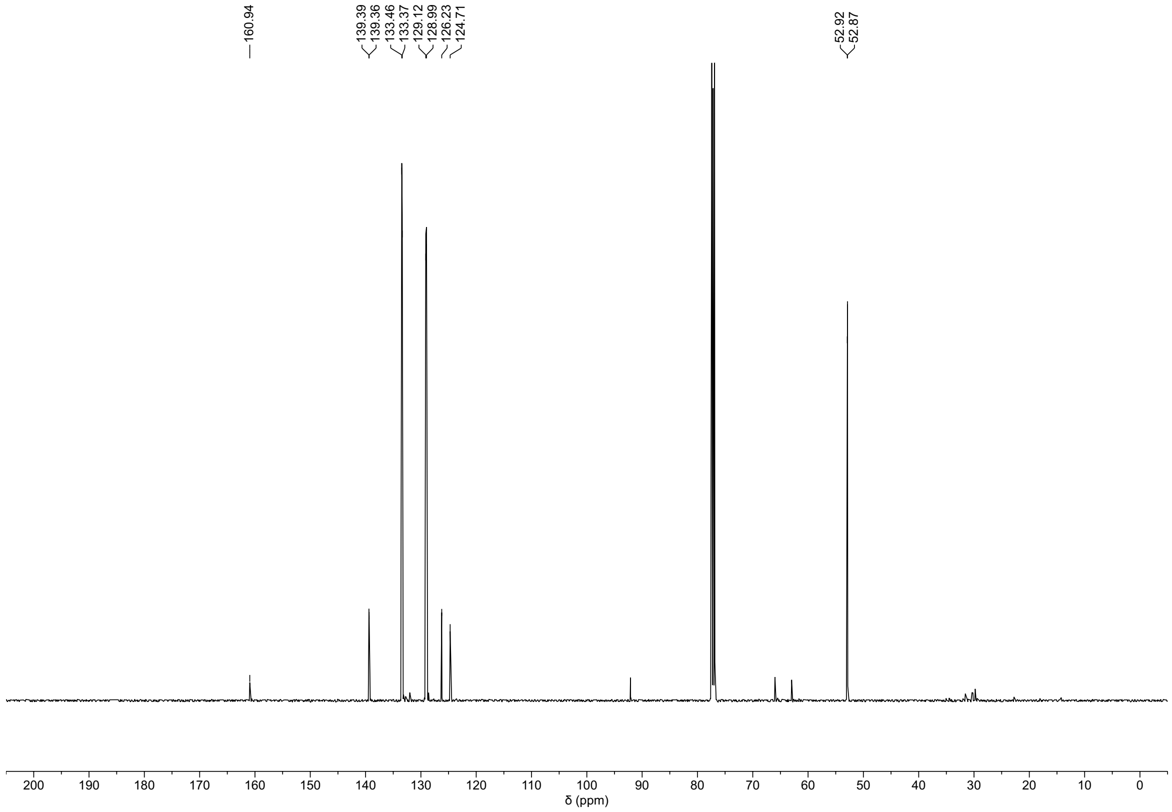

Figure 40. ^{13}C NMR – Dimethyl (4-chlorophenyl)phosphonate (**7e-*p***), 126 MHz, $CDCl_3$.

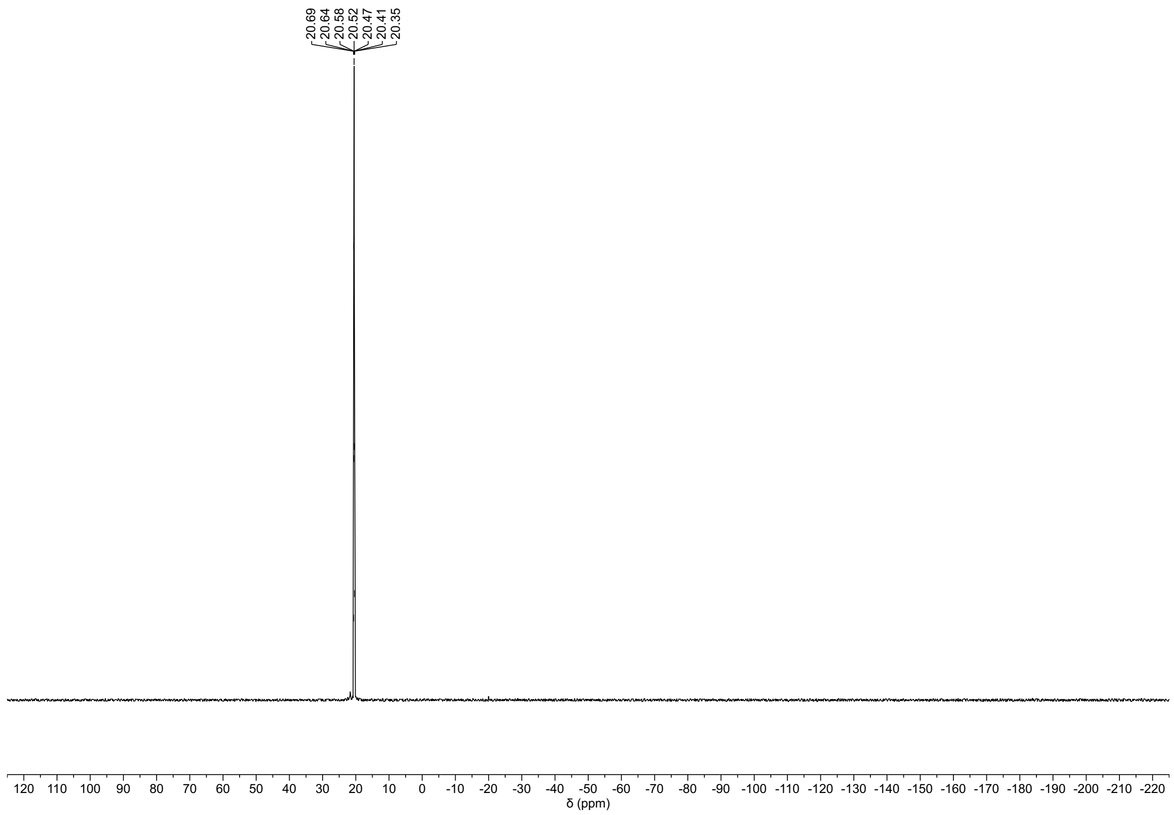

Figure 41. ^{31}P NMR – Dimethyl (4-chlorophenyl)phosphonate (**7e-*p***), 202 MHz, $CDCl_3$.

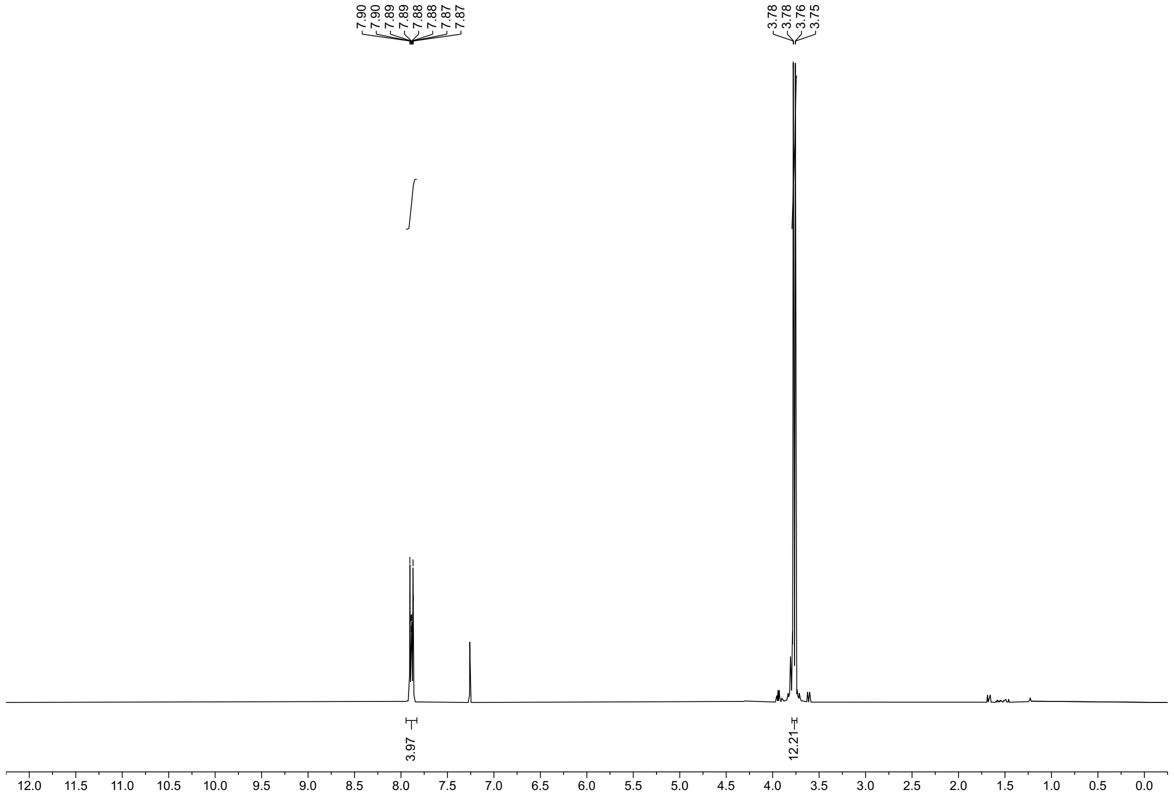

Figure 42. ^{1}H NMR – Tetramethyl 1,4-phenylenebis(phosphonate) (**7e’/f’/g’-*p***), 500 MHz, $CDCl_3$.

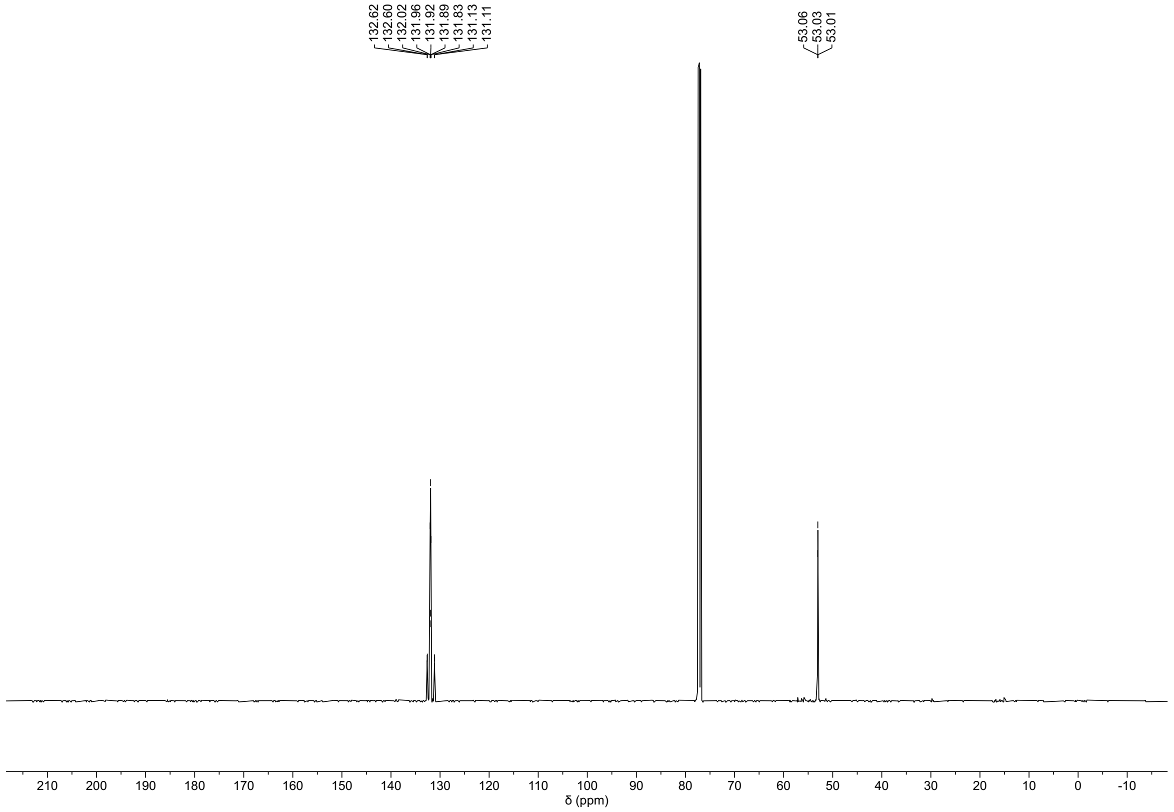

Figure 43. ^{13}C NMR – Tetramethyl 1,4-phenylenebis(phosphonate) (**7e’/f’/g’-*p***), 126 MHz, $CDCl_3$.

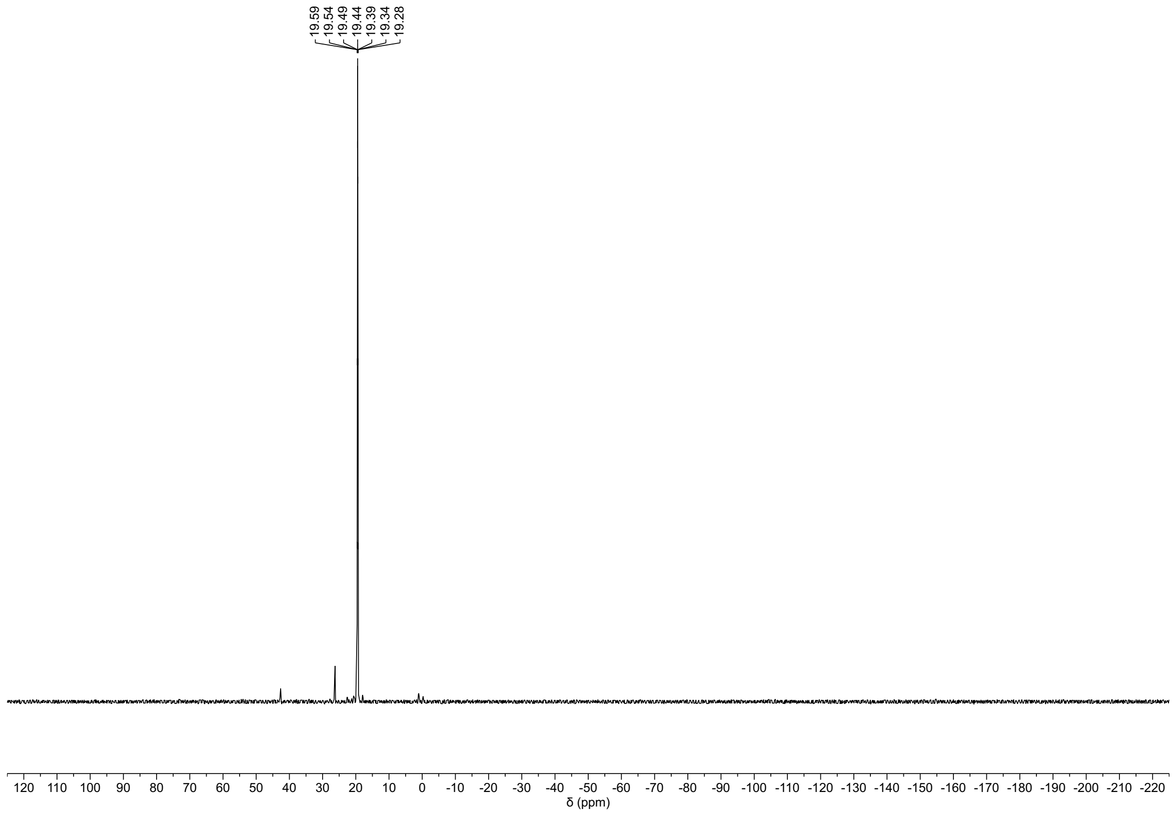

Figure 44. ^{31}P NMR – Tetramethyl 1,4-phenylenebis(phosphonate) (**7e’/f’/g’-*p***), 202 MHz, $CDCl_3$.

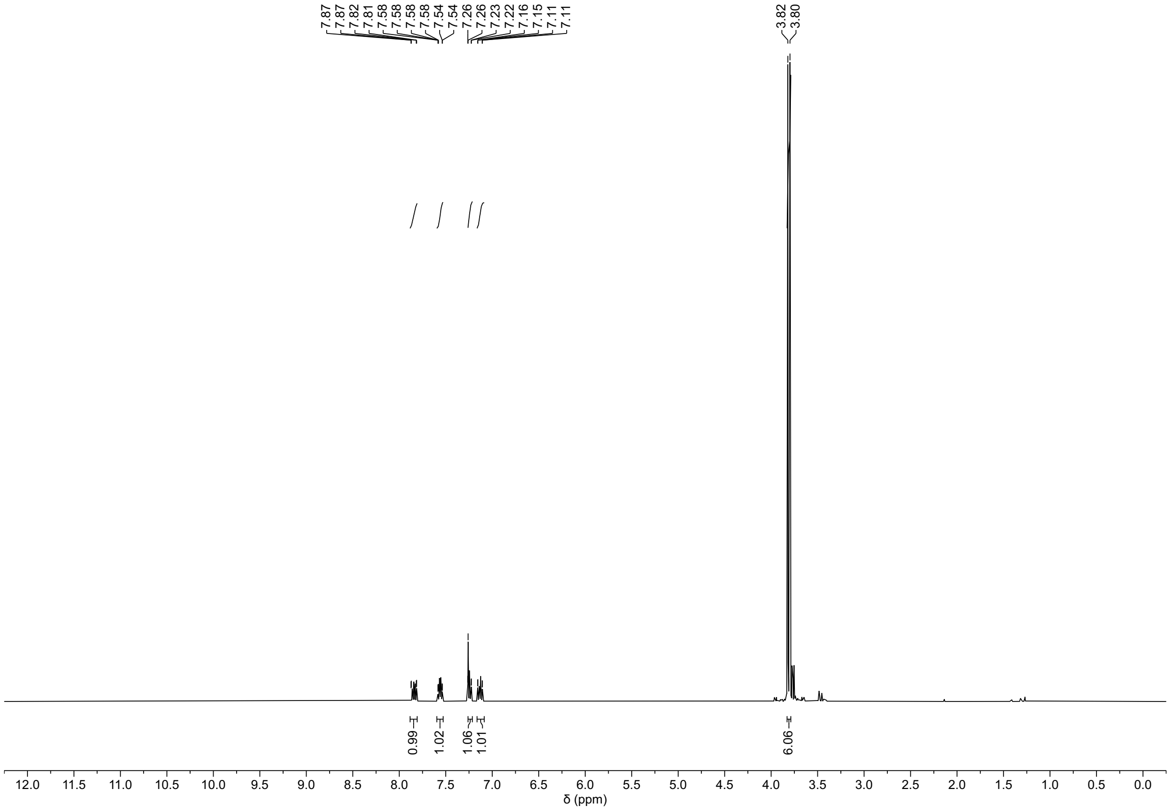

Figure 45. ^{1}H NMR – Dimethyl (2-fluorophenyl)phosphonate (**7f-*o***), 500 MHz, $CDCl_3$.

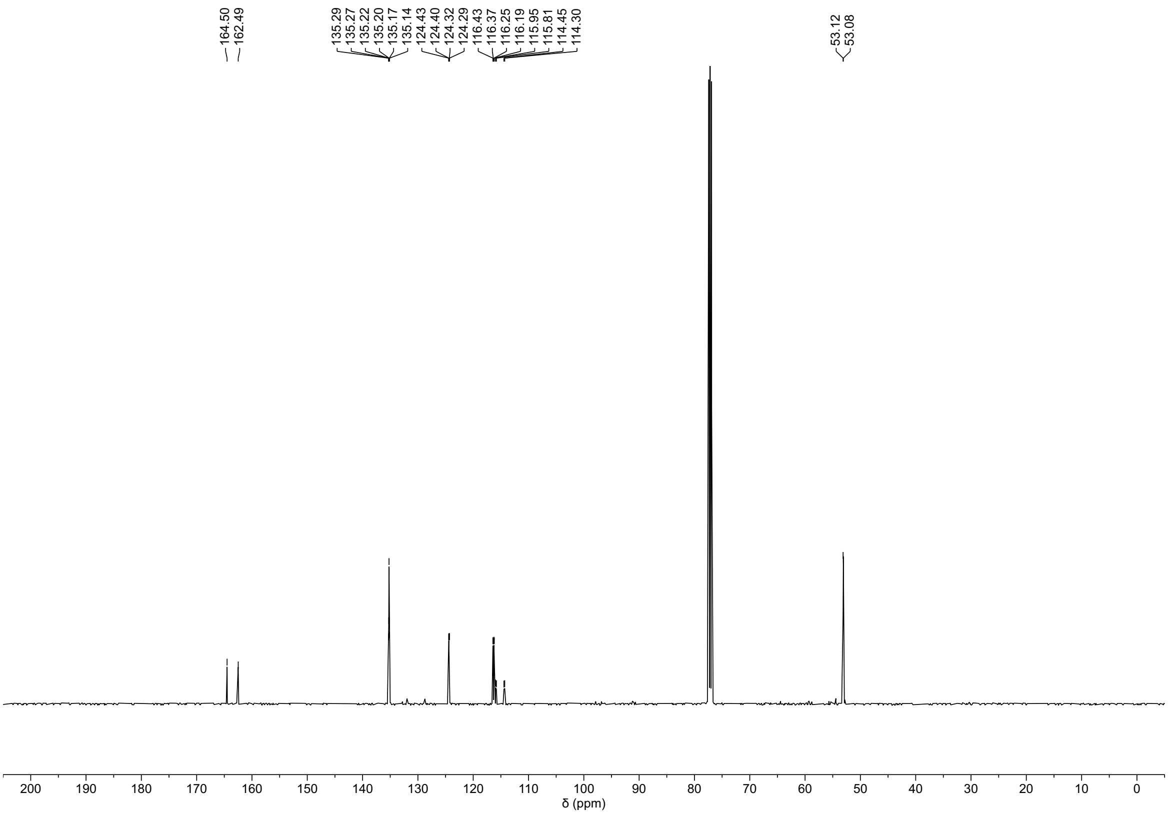

Figure 46. ^{13}C NMR – Dimethyl (2-fluorophenyl)phosphonate (**7f-*o***), 126 MHz, $CDCl_3$.

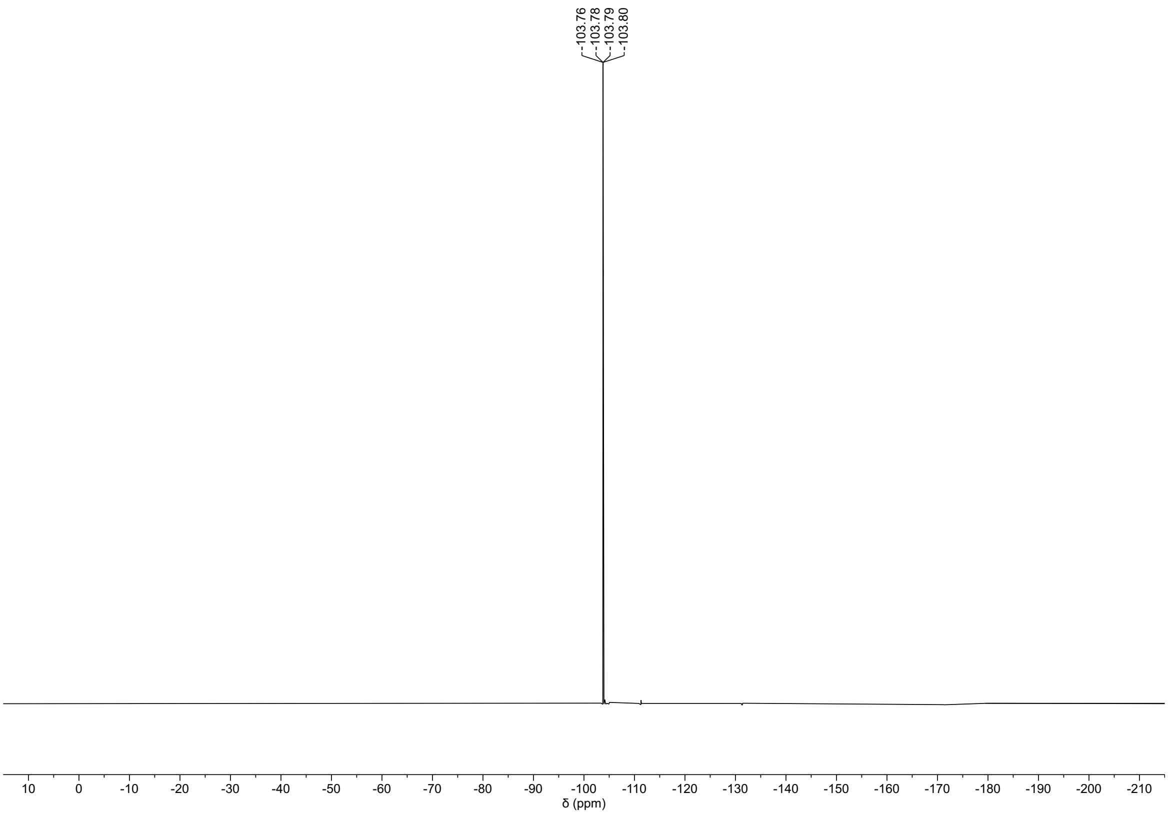

Figure 47. ^{19}F NMR – Dimethyl (2-fluorophenyl)phosphonate (**7f-*o***), 471 MHz, $CDCl_3$.

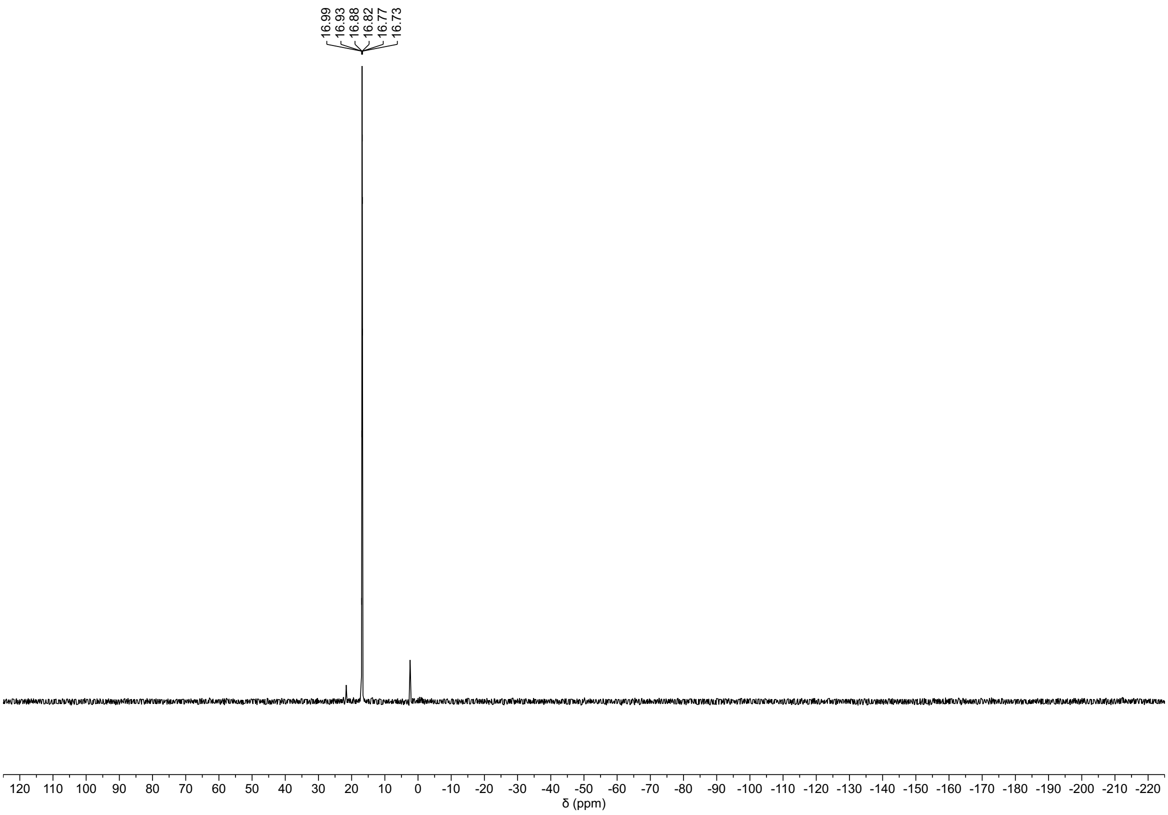

Figure 48. ^{31}P NMR – Dimethyl (2-fluorophenyl)phosphonate (**7f-*o***), 202 MHz, $CDCl_3$.

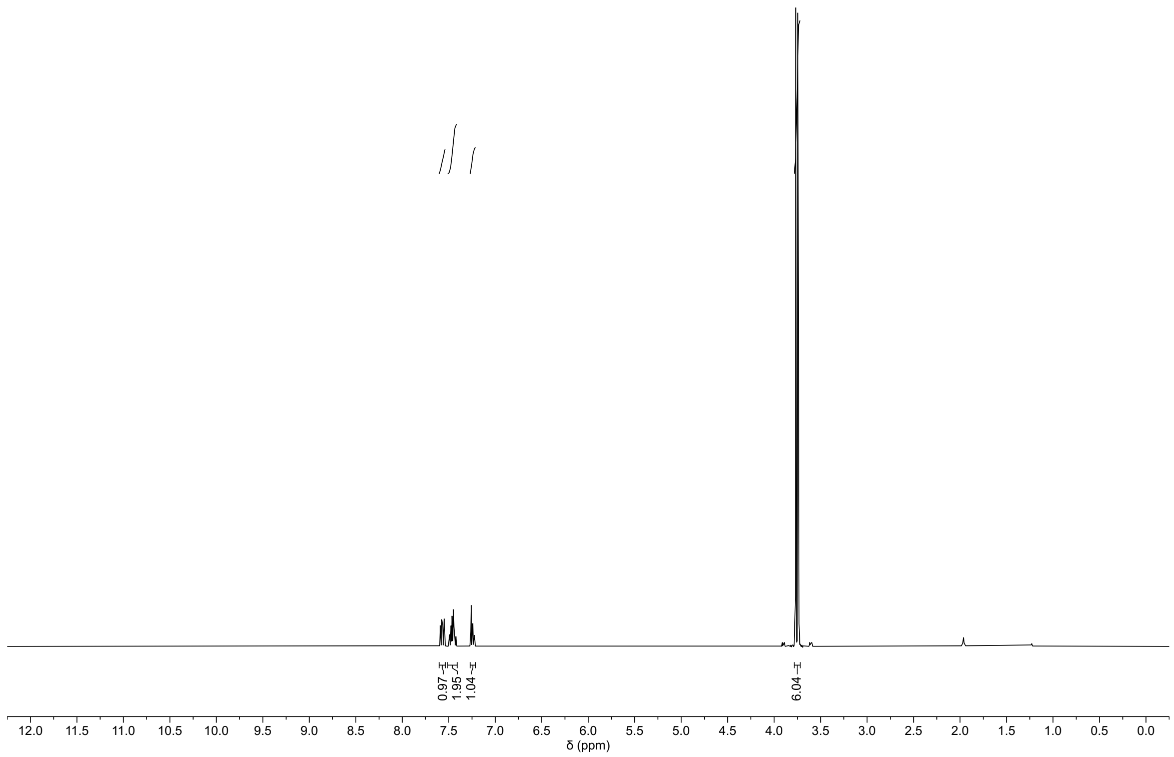

Figure 49. ^{1}H NMR – Dimethyl (3-fluorophenyl)phosphonate (**7f-*m***), 500 MHz, $CDCl_3$.

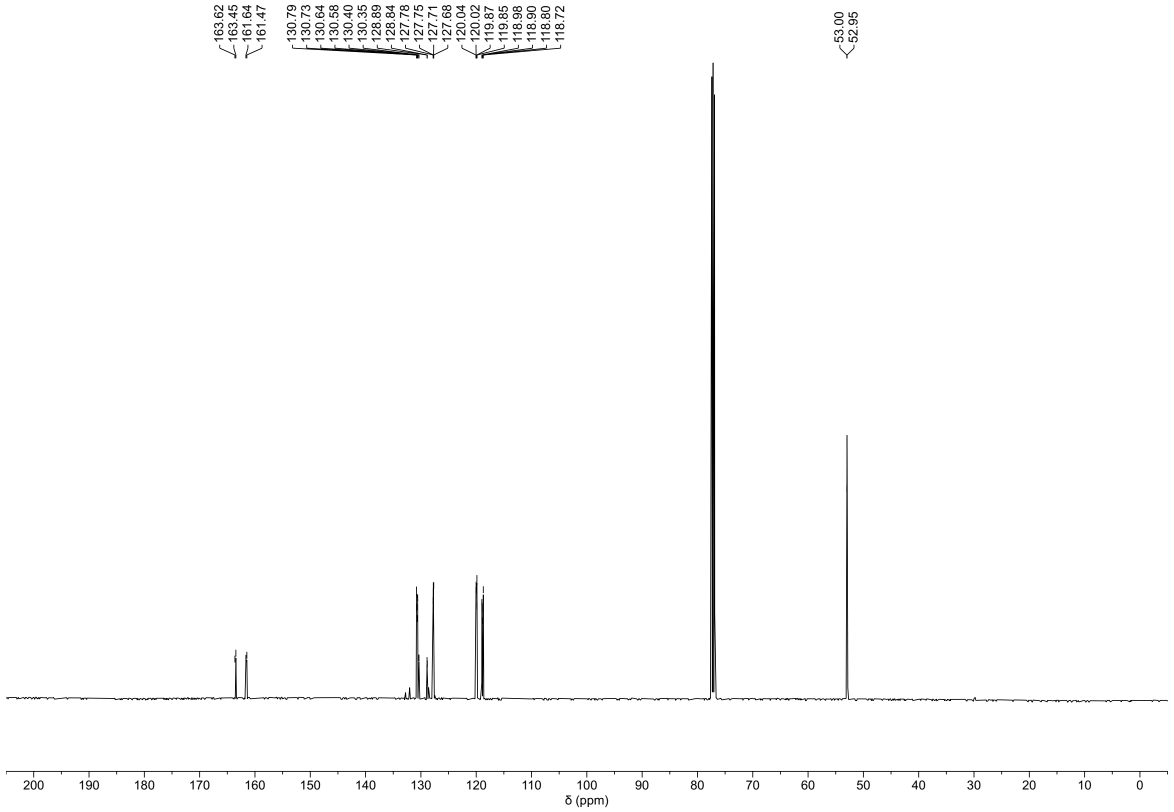

Figure 50. ^{13}C NMR – Dimethyl (3-fluorophenyl)phosphonate (**7f-*m***), 126 MHz, $CDCl_3$.

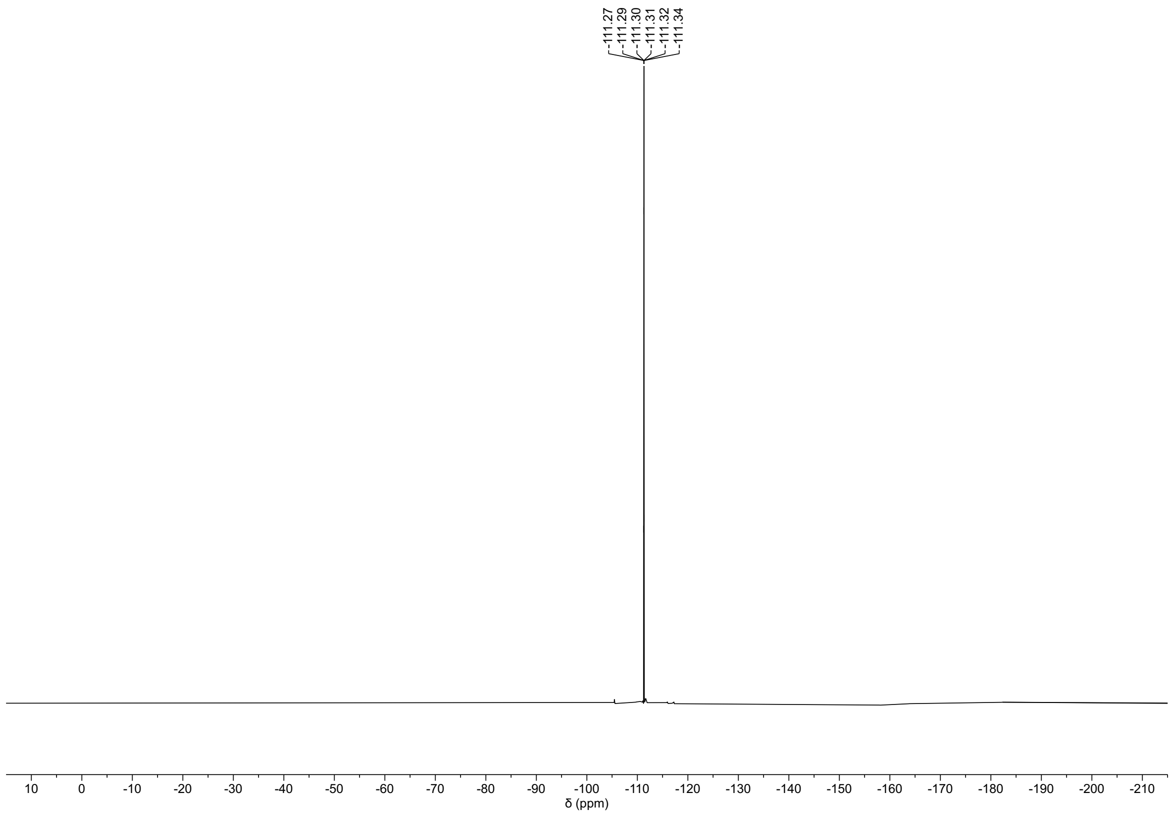

Figure 51. ^{19}F NMR – Dimethyl (3-fluorophenyl)phosphonate (**7f-*m***), 471 MHz, $CDCl_3$.

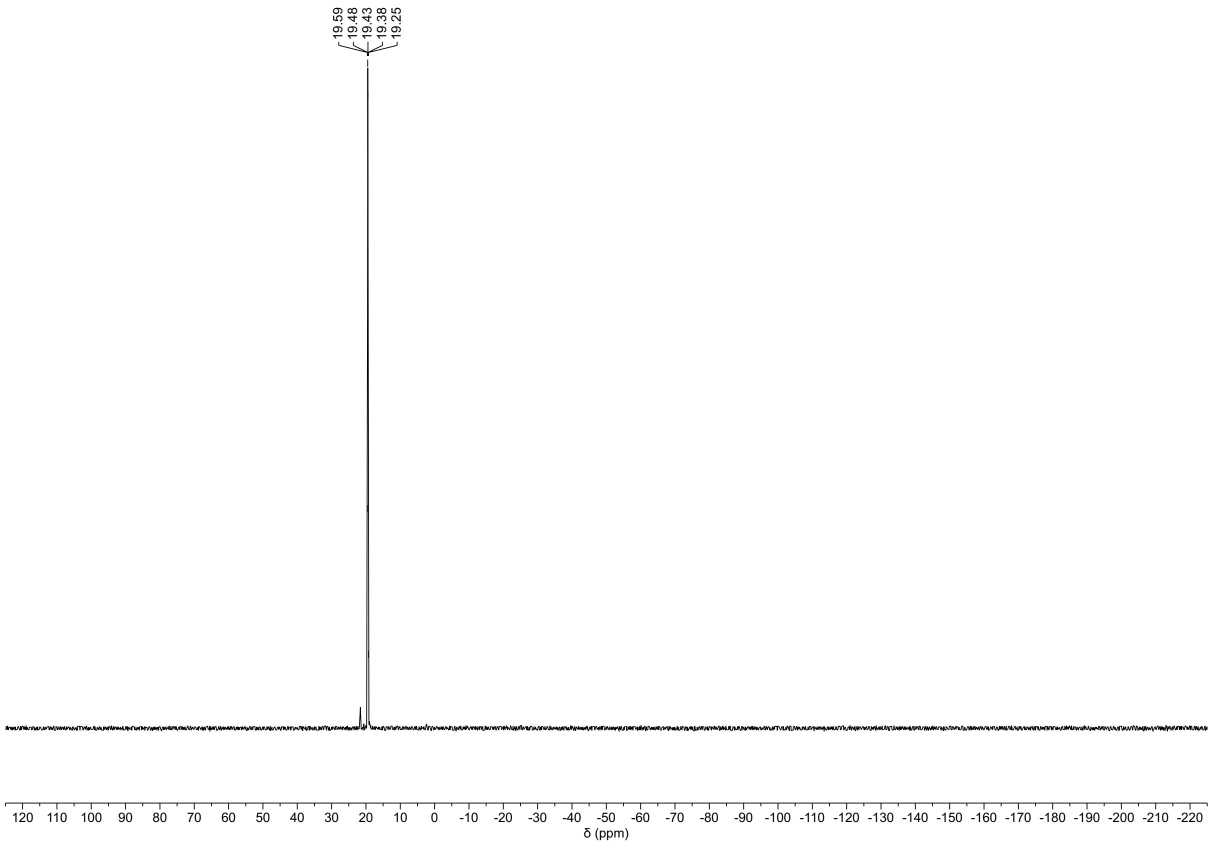

Figure 52. ^{31}P NMR – Dimethyl (3-fluorophenyl)phosphonate (**7f-*m***), 202 MHz, $CDCl_3$.

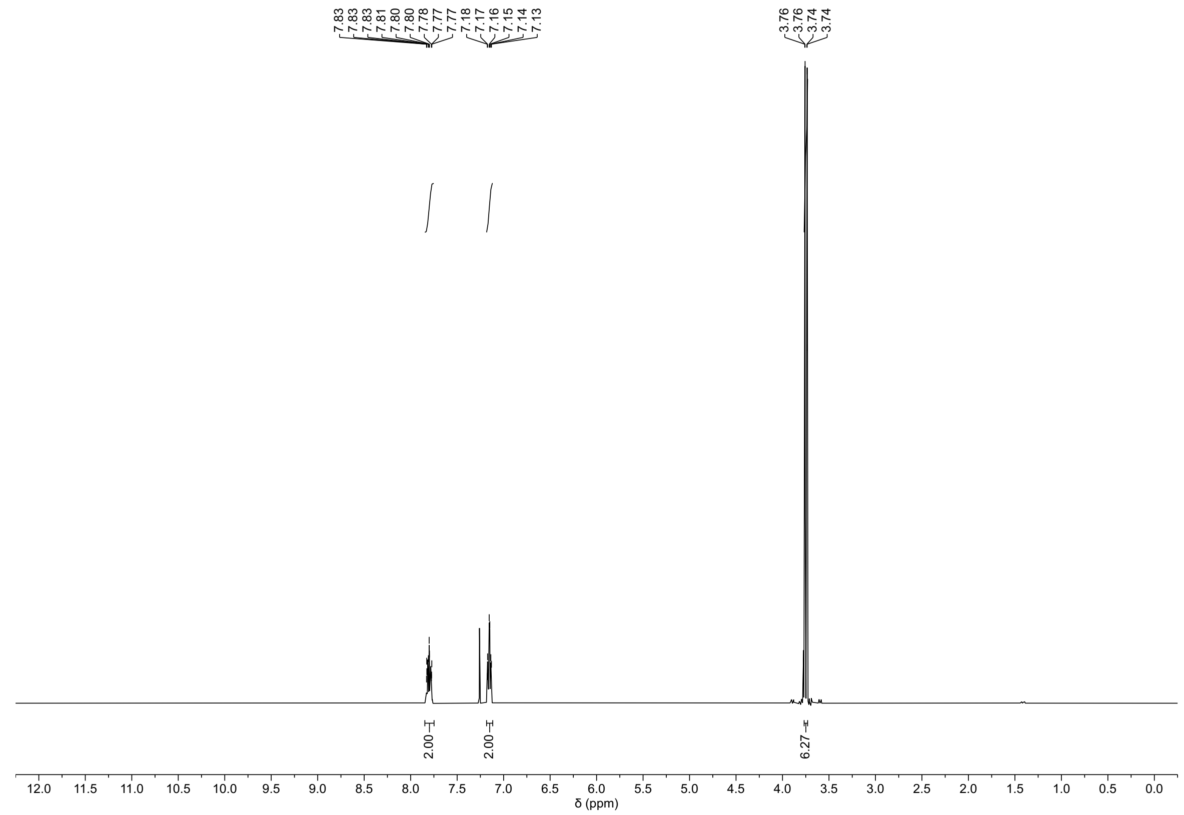

Figure 53. ^{1}H NMR – Dimethyl (4-fluorophenyl)phosphonate (**7f-*p***), 500 MHz, $CDCl_3$.

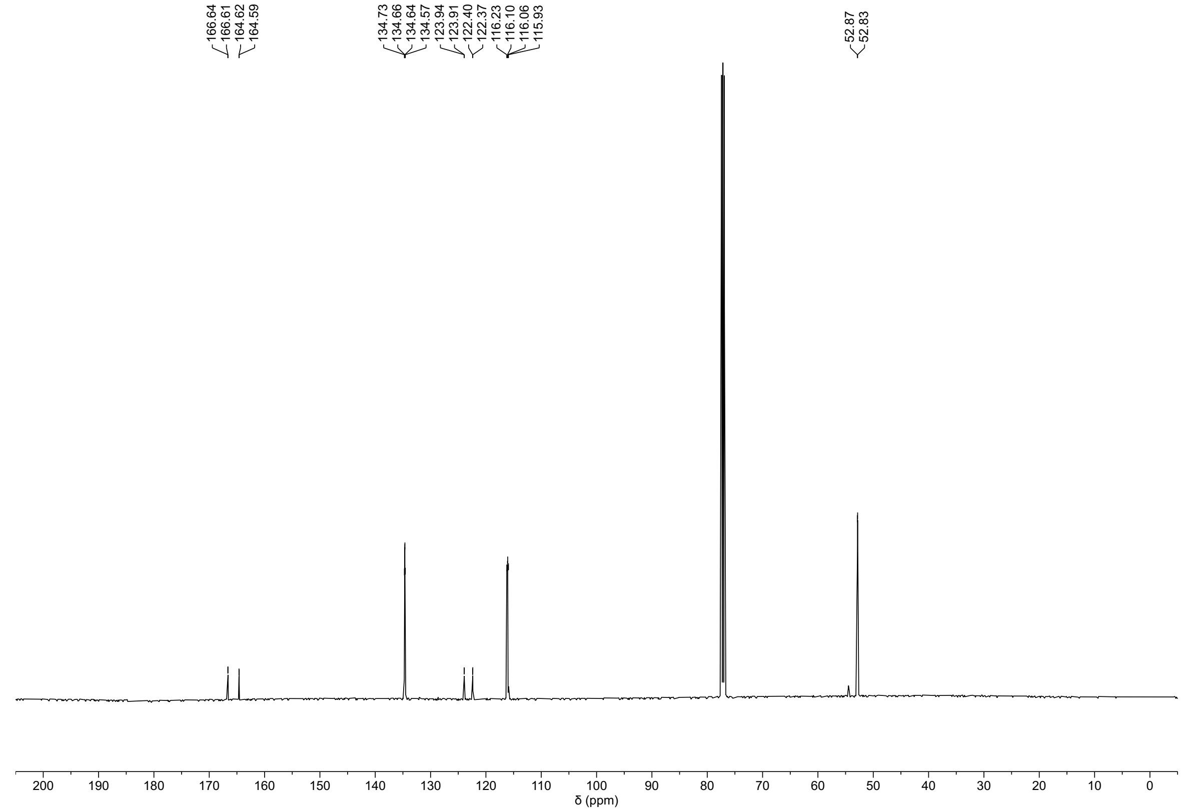

Figure 54. ^{13}C NMR – Dimethyl (4-fluorophenyl)phosphonate (**7f-*p***), 126 MHz, $CDCl_3$.

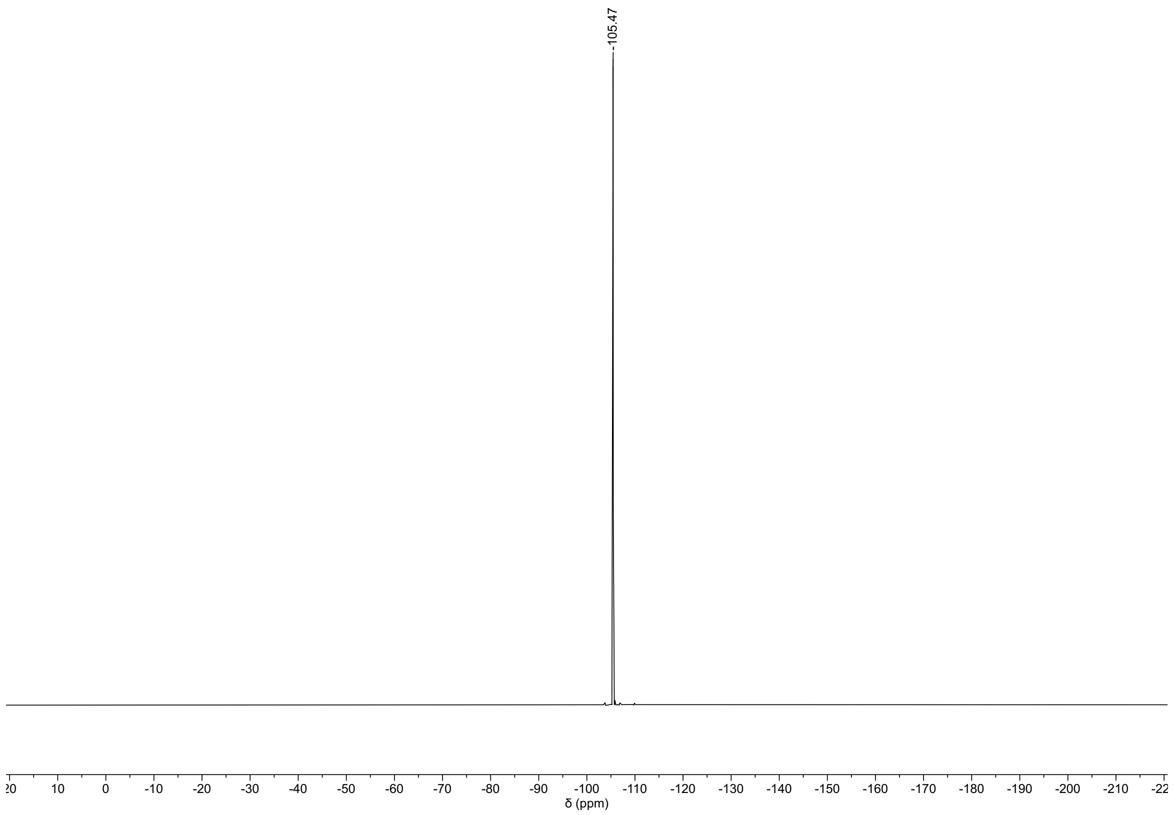

Figure 55. ^{19}F NMR – Dimethyl (4-fluorophenyl)phosphonate (**7f-*p***), 471 MHz, $CDCl_3$.

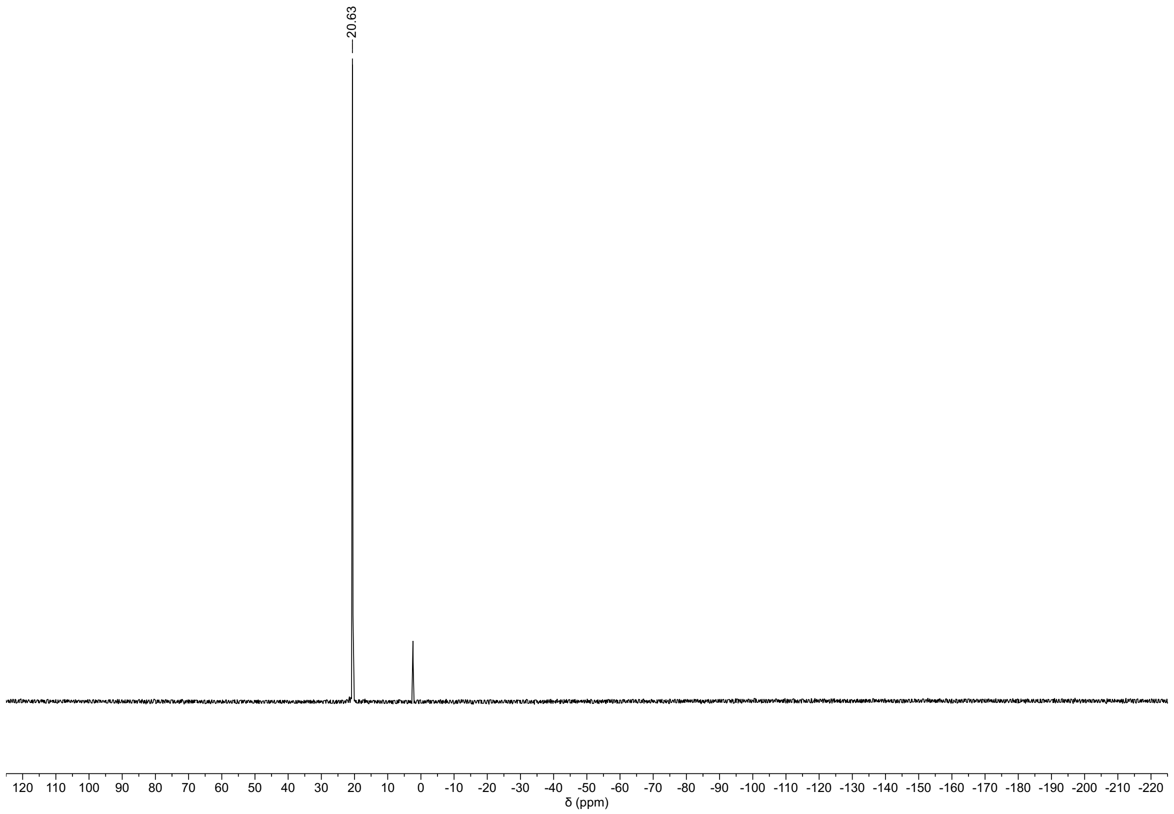

Figure 56. ^{31}P NMR – Dimethyl (4-fluorophenyl)phosphonate (**7f-*p***), 202 MHz, $CDCl_3$.

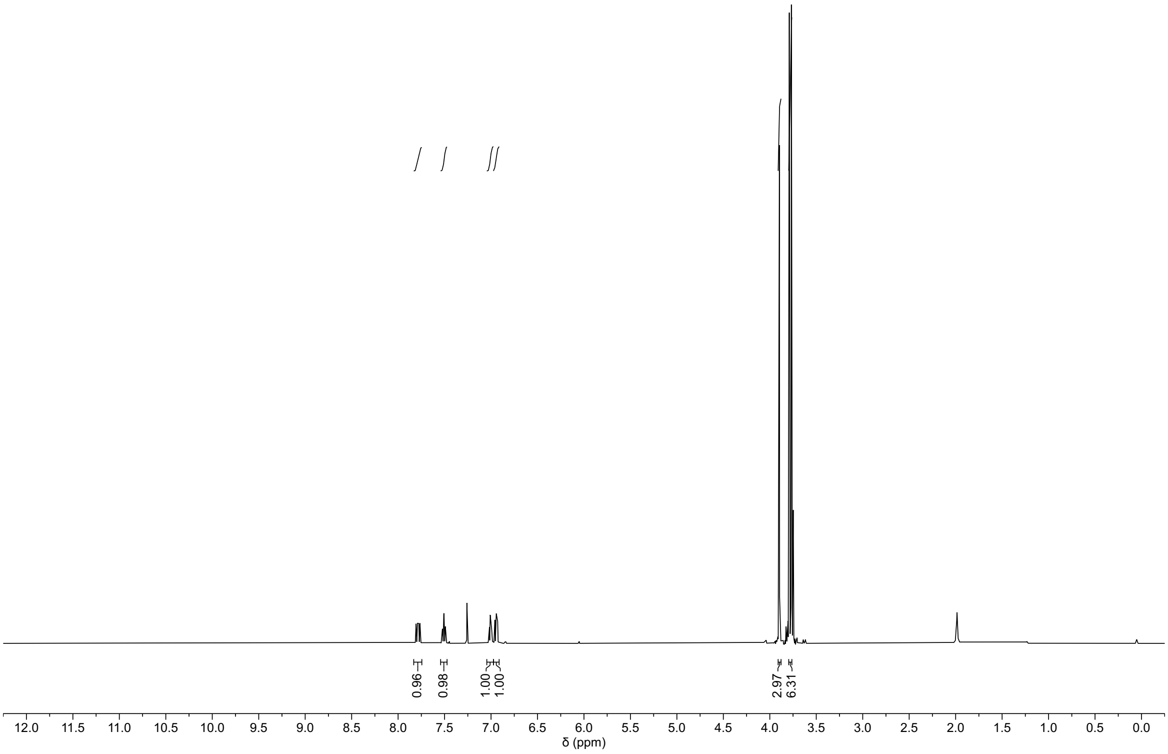

Figure 57. ^{1}H NMR – Dimethyl (2-methoxyphenyl)phosphonate (**7g-*o***), 500 MHz, $CDCl_3$.

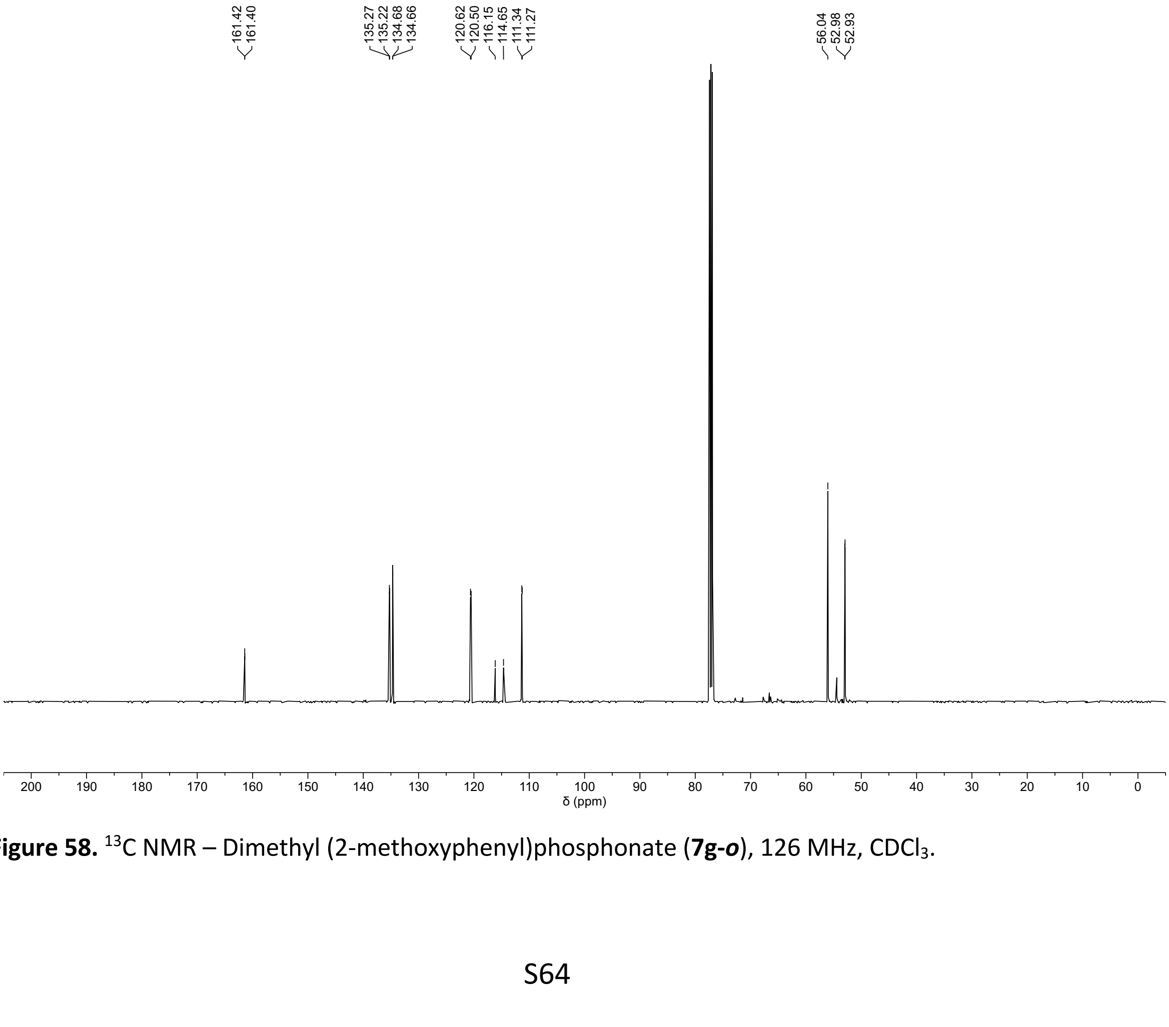

Figure 58. ^{13}C NMR – Dimethyl (2-methoxyphenyl)phosphonate (**7g-*o***), 126 MHz, $CDCl_3$.

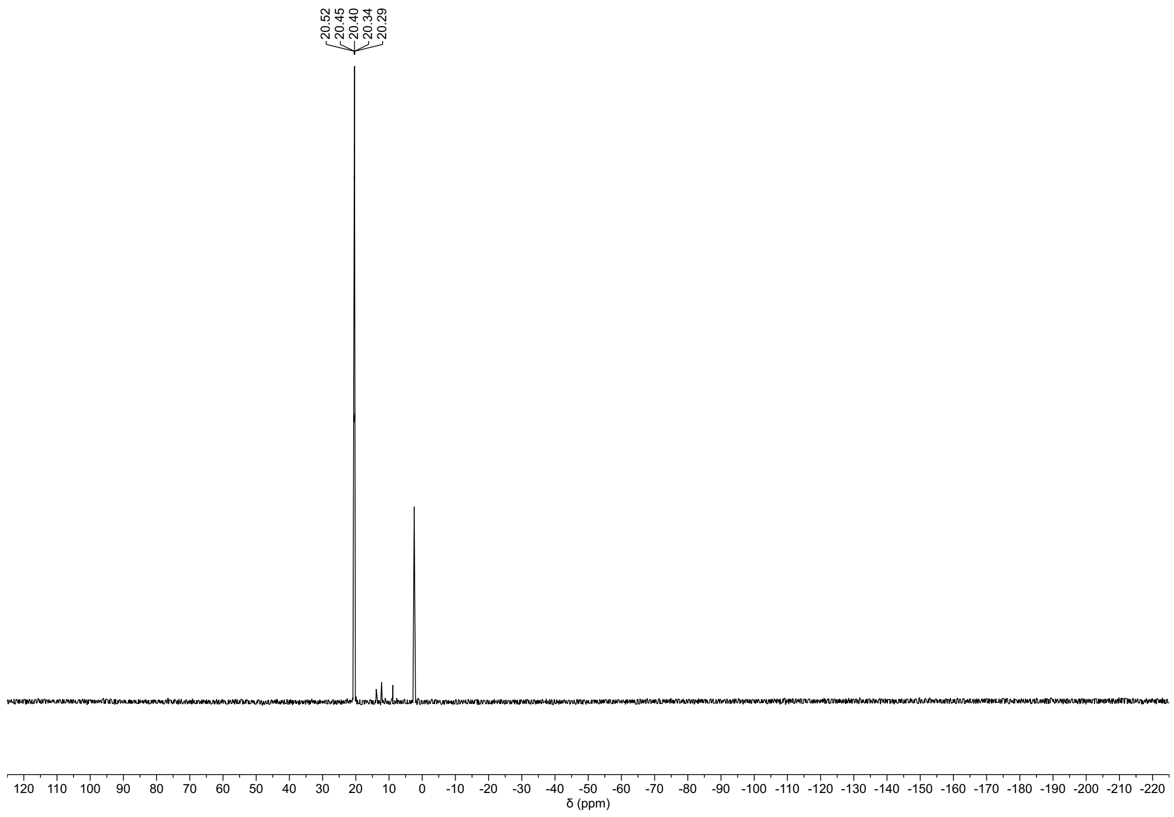

Figure 59. ^{31}P NMR – Dimethyl (2-methoxyphenyl)phosphonate (**7g-*o***), 202 MHz, $CDCl_3$.

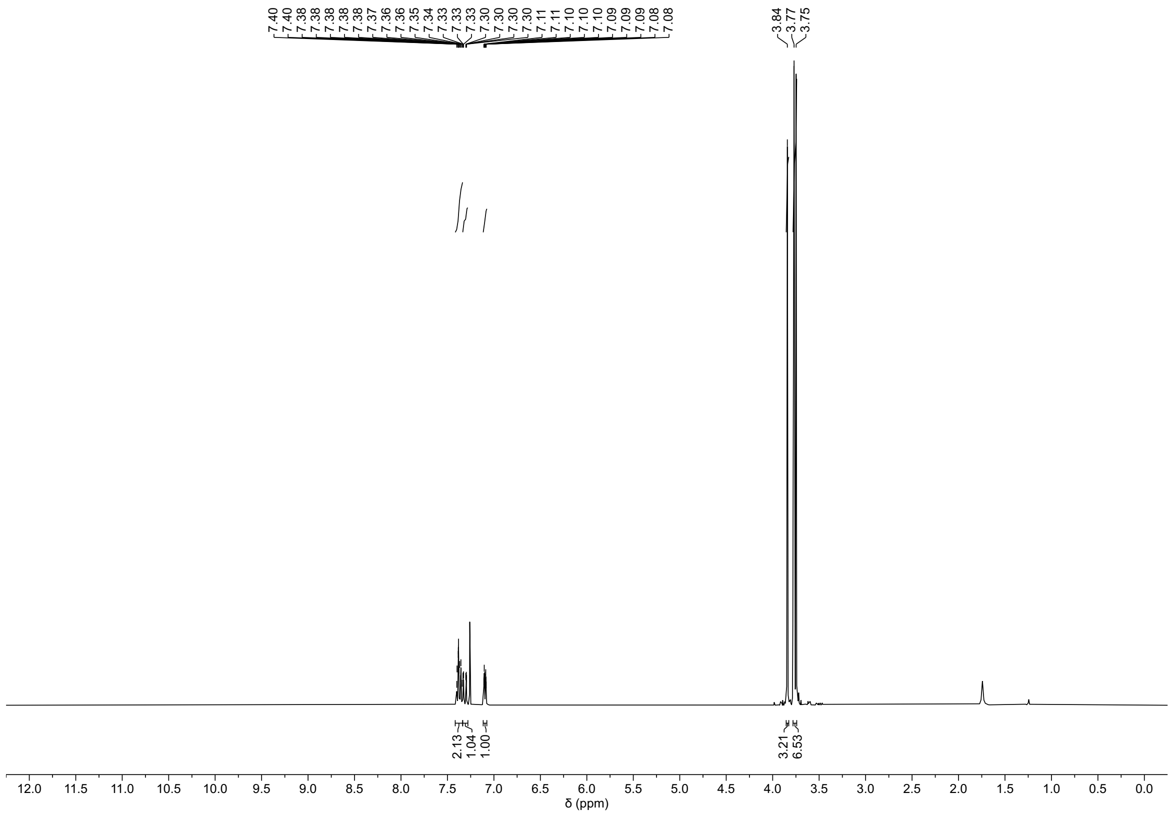

Figure 60. ^{1}H NMR – Dimethyl (3-methoxyphenyl)phosphonate (**7g-*m***), 500 MHz, $CDCl_3$.

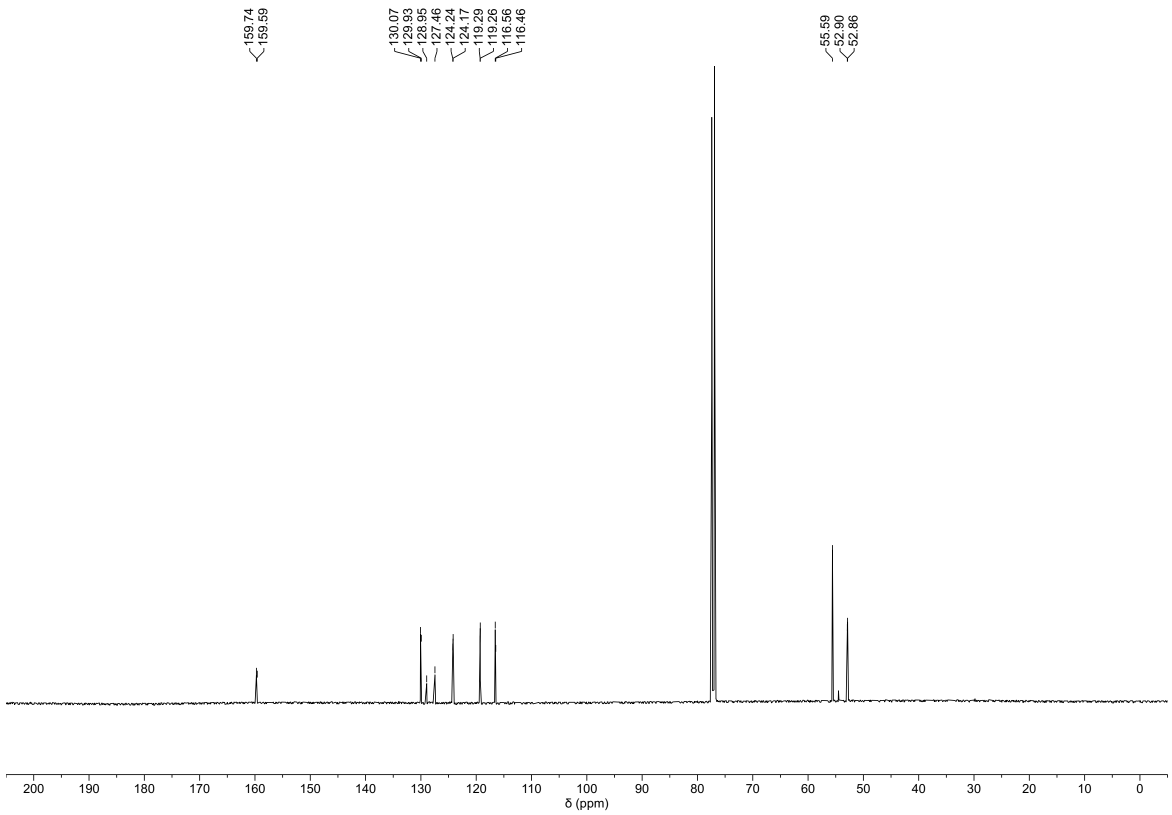

Figure 61. ^{13}C NMR – Dimethyl (3-methoxyphenyl)phosphonate (**7g-*m***), 126 MHz, $CDCl_3$.

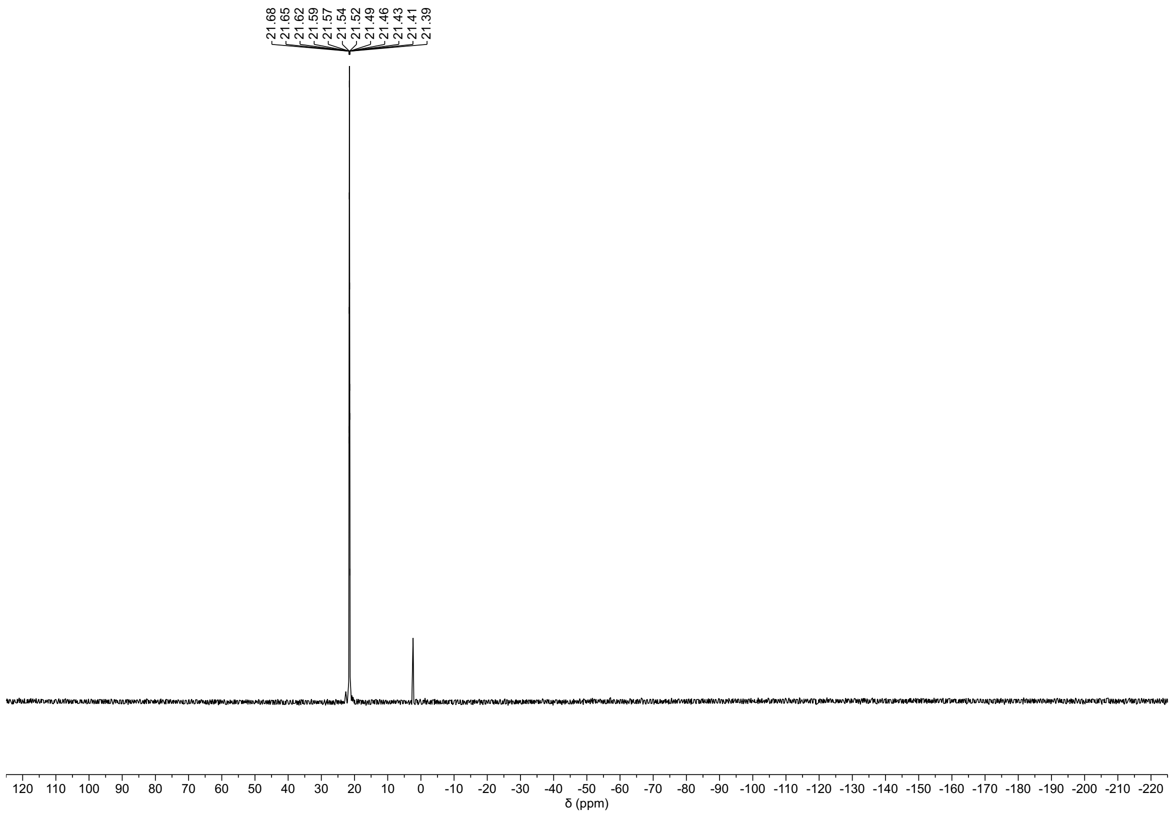

Figure 62. ^{31}P NMR – Dimethyl (3-methoxyphenyl)phosphonate (**7g-*m***), 202 MHz, $CDCl_3$.

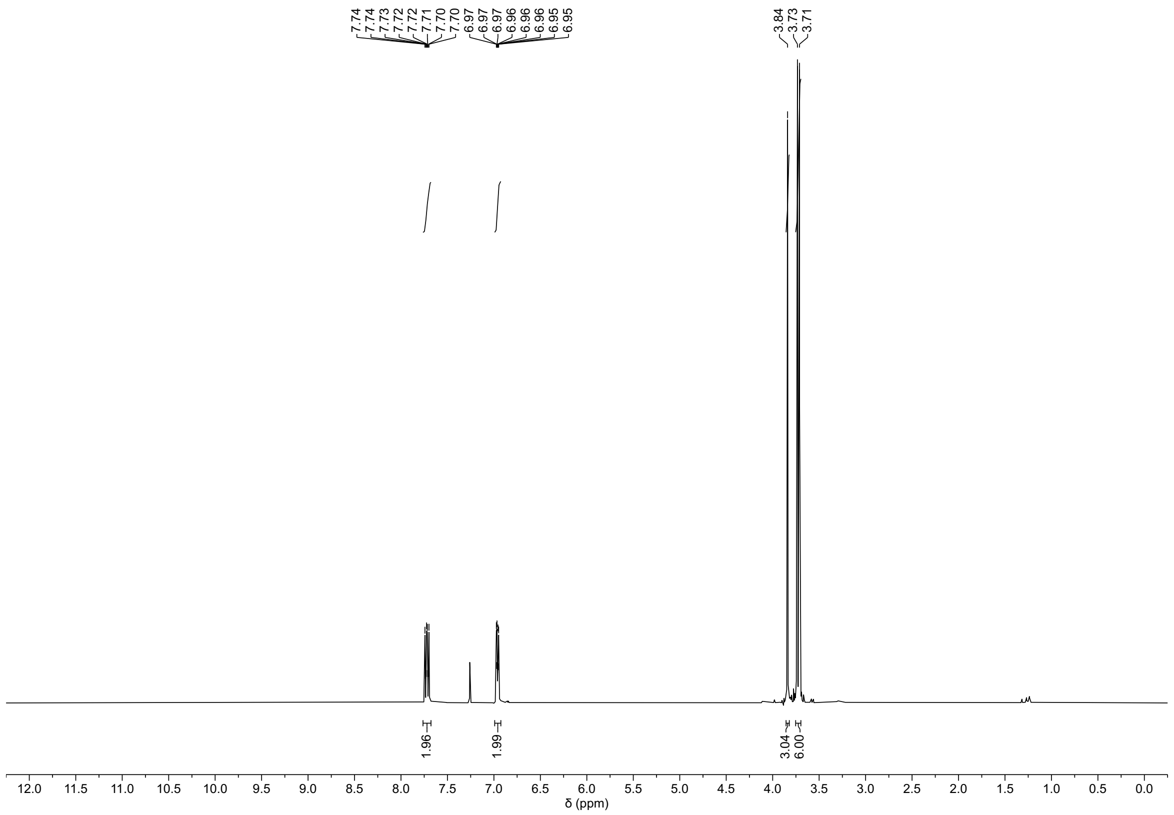

Figure 63. ^{1}H NMR – Dimethyl (4-methoxyphenyl)phosphonate (**7g-*p***), 500 MHz, $CDCl_3$.

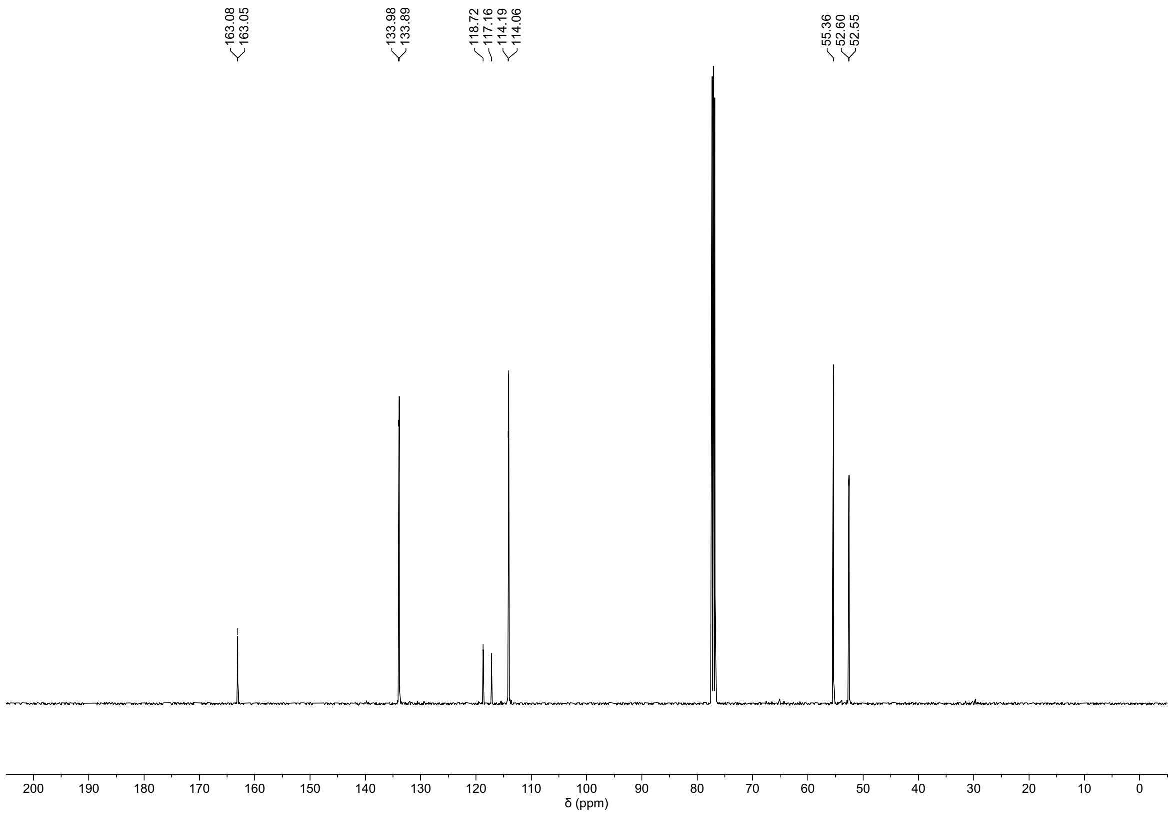

Figure 64. ^{13}C NMR – Dimethyl (4-methoxyphenyl)phosphonate (**7g-*p***), 126 MHz, $CDCl_3$.

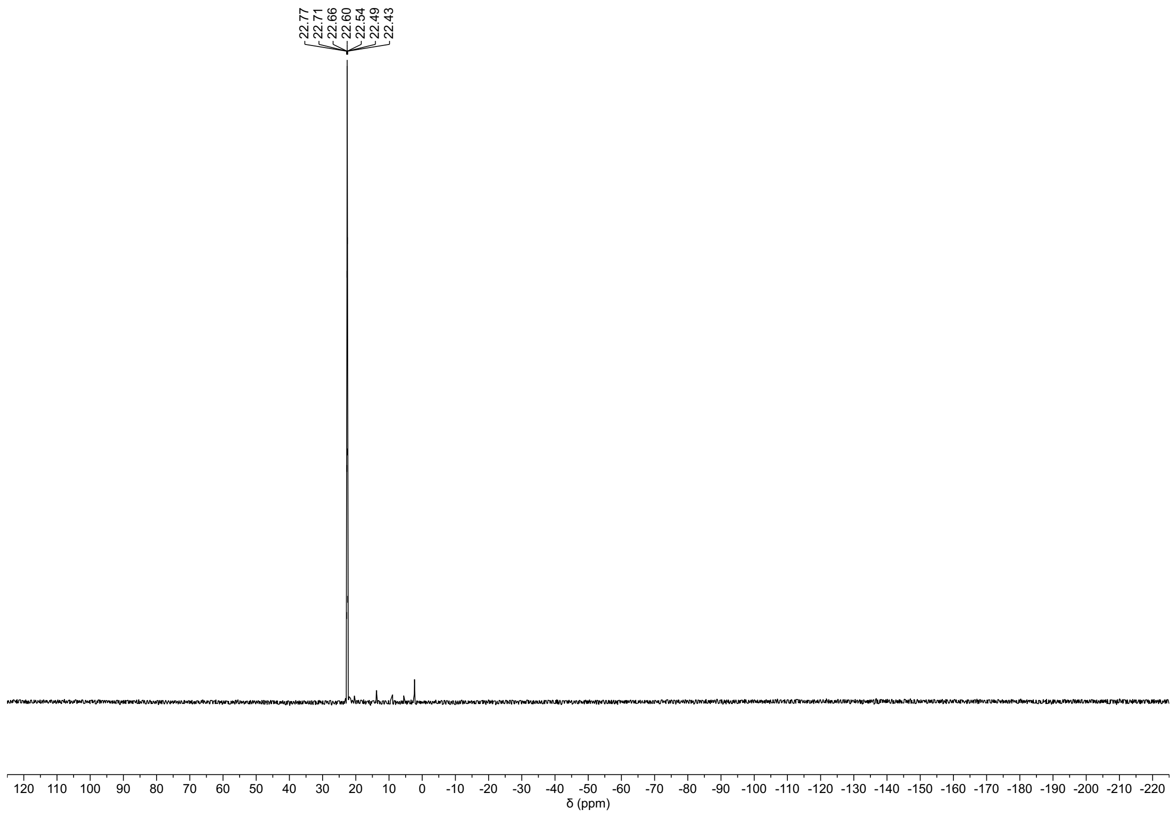

Figure 65. ^{31}P NMR – Dimethyl (4-methoxyphenyl)phosphonate (***7g-p***), 202 MHz, $CDCl_3$.

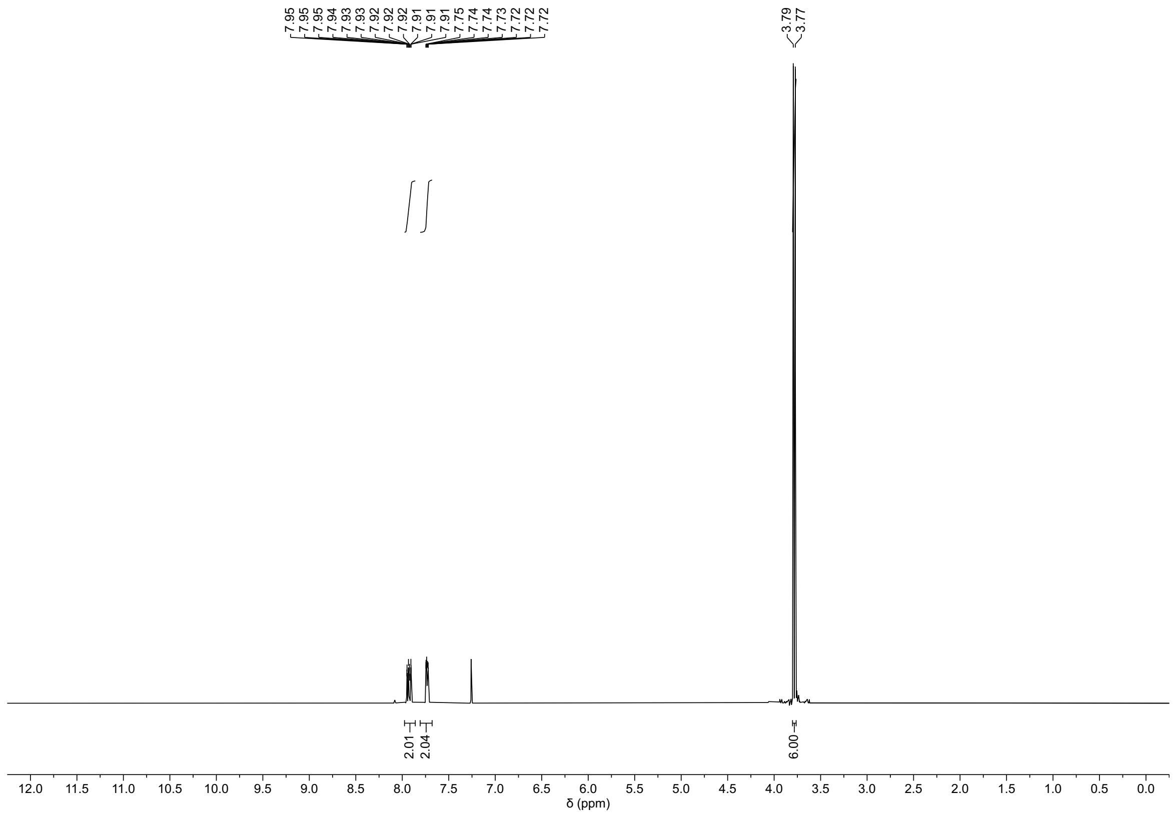

Figure 66. ^{1}H NMR – Dimethyl (4-(trifluoromethyl)phenyl)phosphonate (**7h**), 500 MHz, $CDCl_3$.

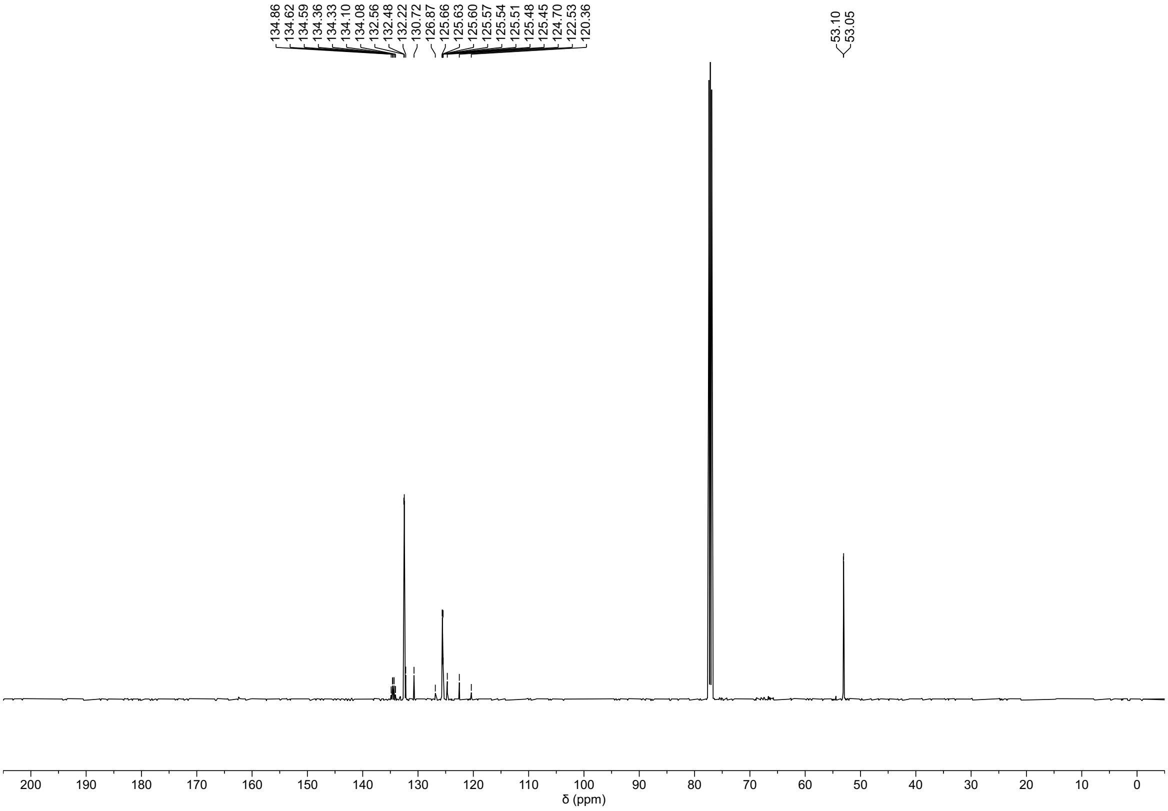

Figure 67. ^{13}C NMR – Dimethyl (4-(trifluoromethyl)phenyl)phosphonate (**7h**), 126 MHz, $CDCl_3$.

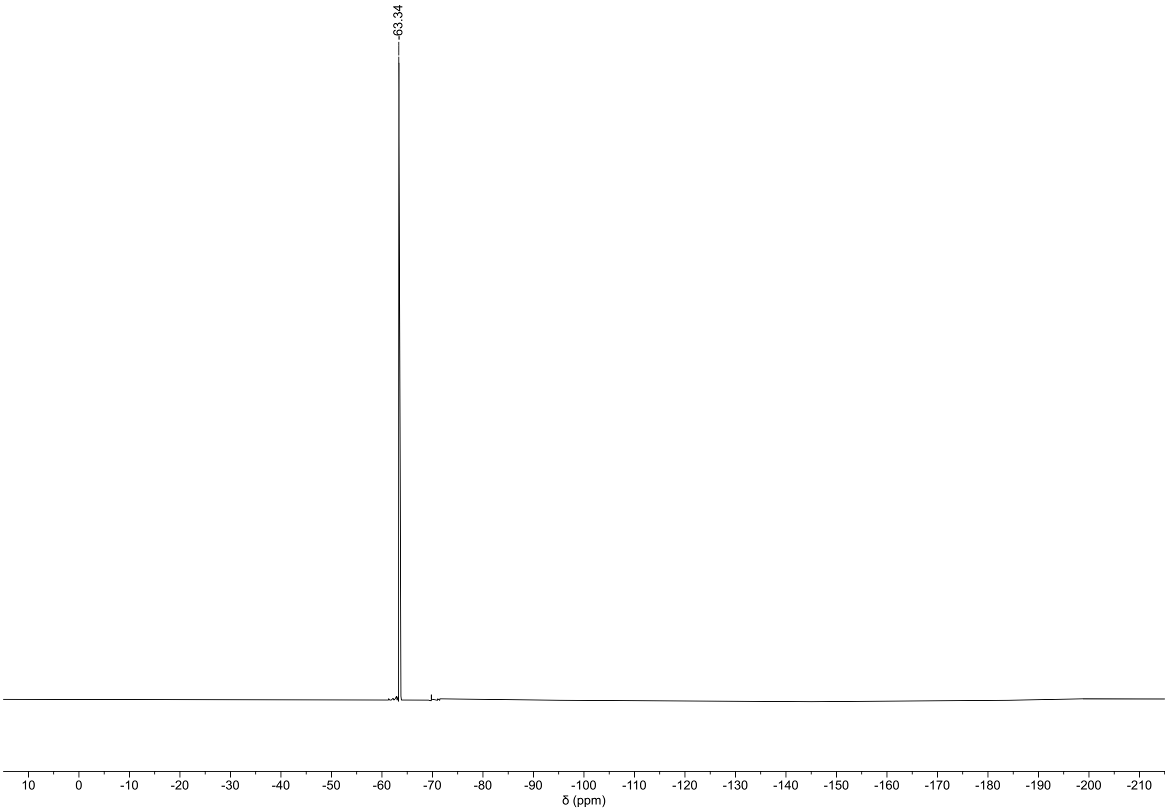

Figure 68. ^{19}F NMR – Dimethyl (4-(trifluoromethyl)phenyl)phosphonate (**7h**), 471 MHz, $CDCl_3$.

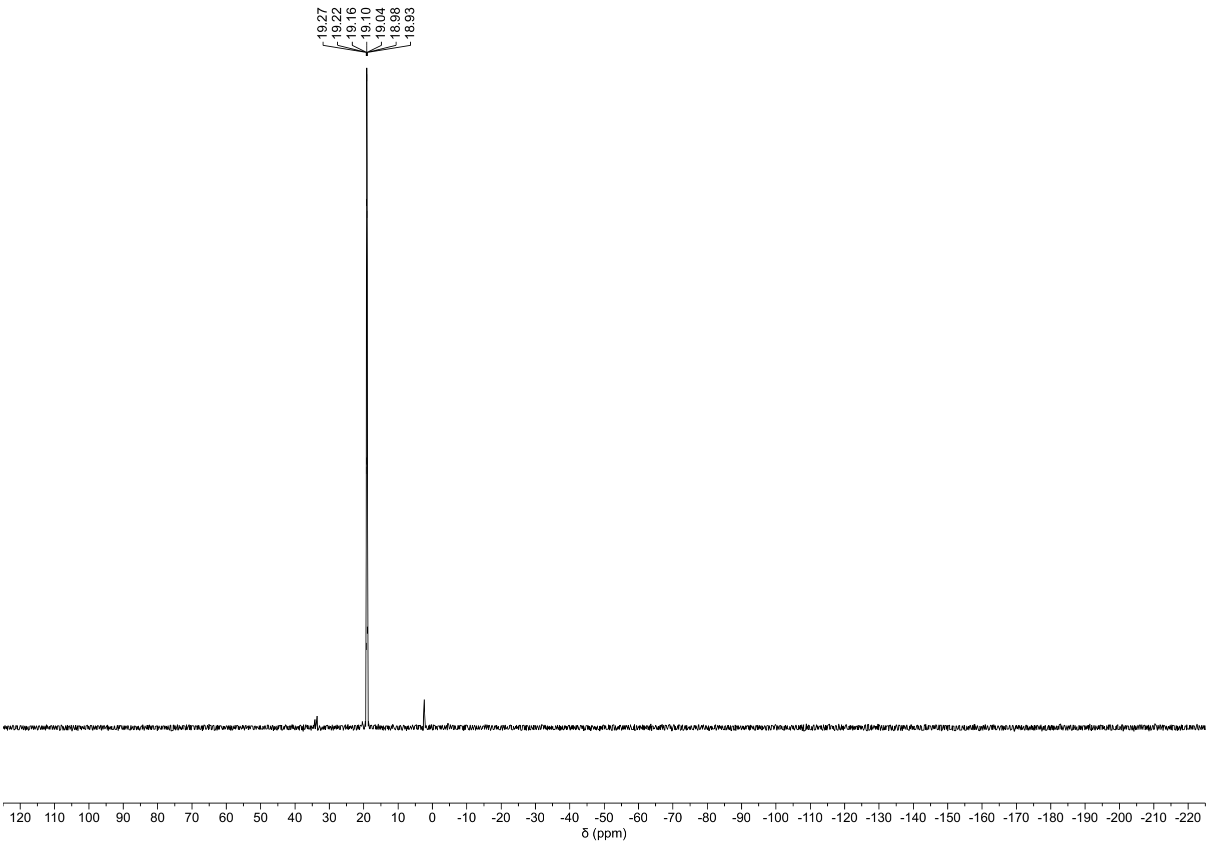

Figure 69. ^{31}P NMR – Dimethyl (4-(trifluoromethyl)phenyl)phosphonate (**7h**), 202 MHz, $CDCl_3$.

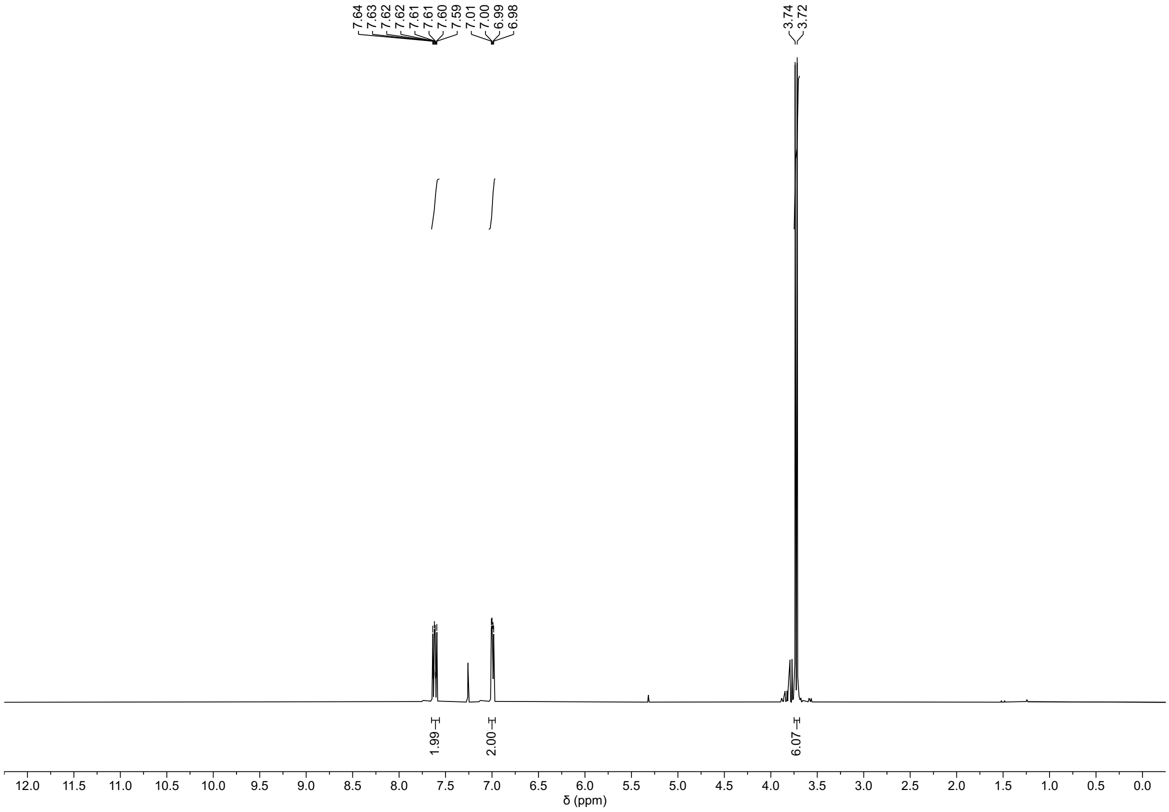

Figure 70. ^{1}H NMR – Dimethyl (4-hydroxyphenyl)phosphonate (**7i**), 500 MHz, $CDCl_3$.

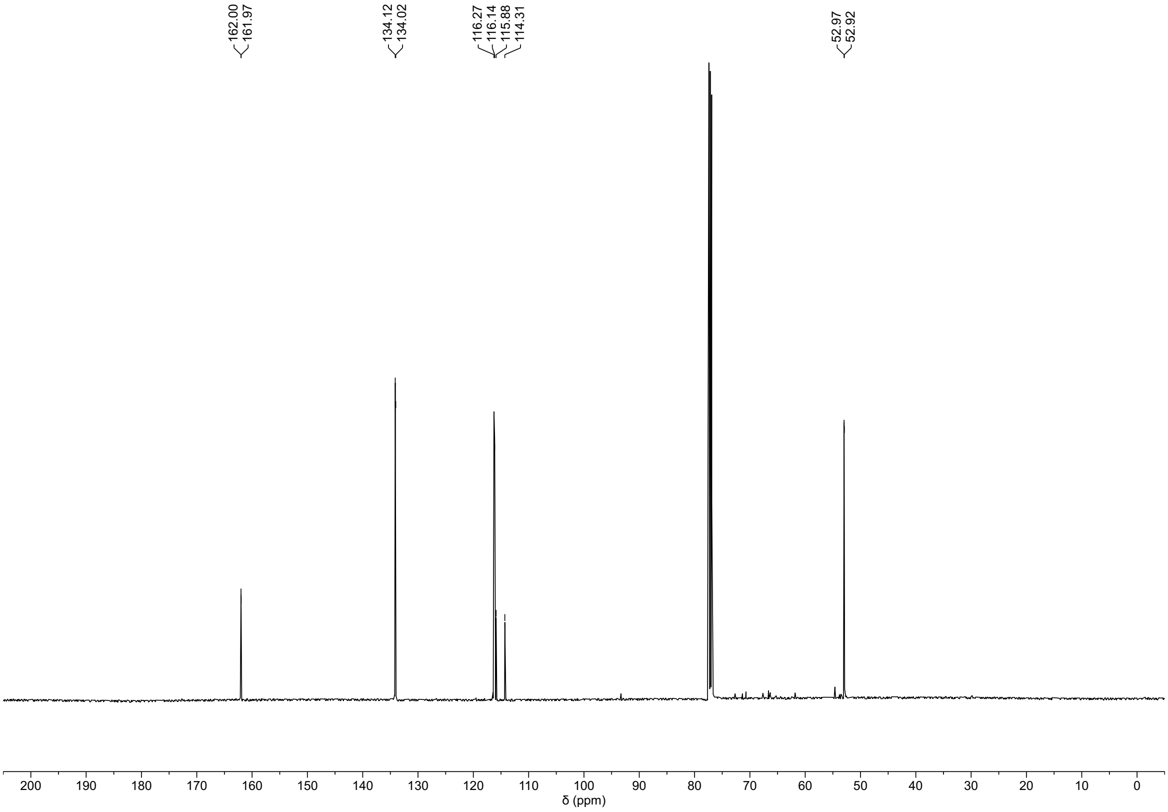

Figure 71. ^{13}C NMR – Dimethyl (4-hydroxyphenyl)phosphonate (**7i**), 126 MHz, $CDCl_3$.

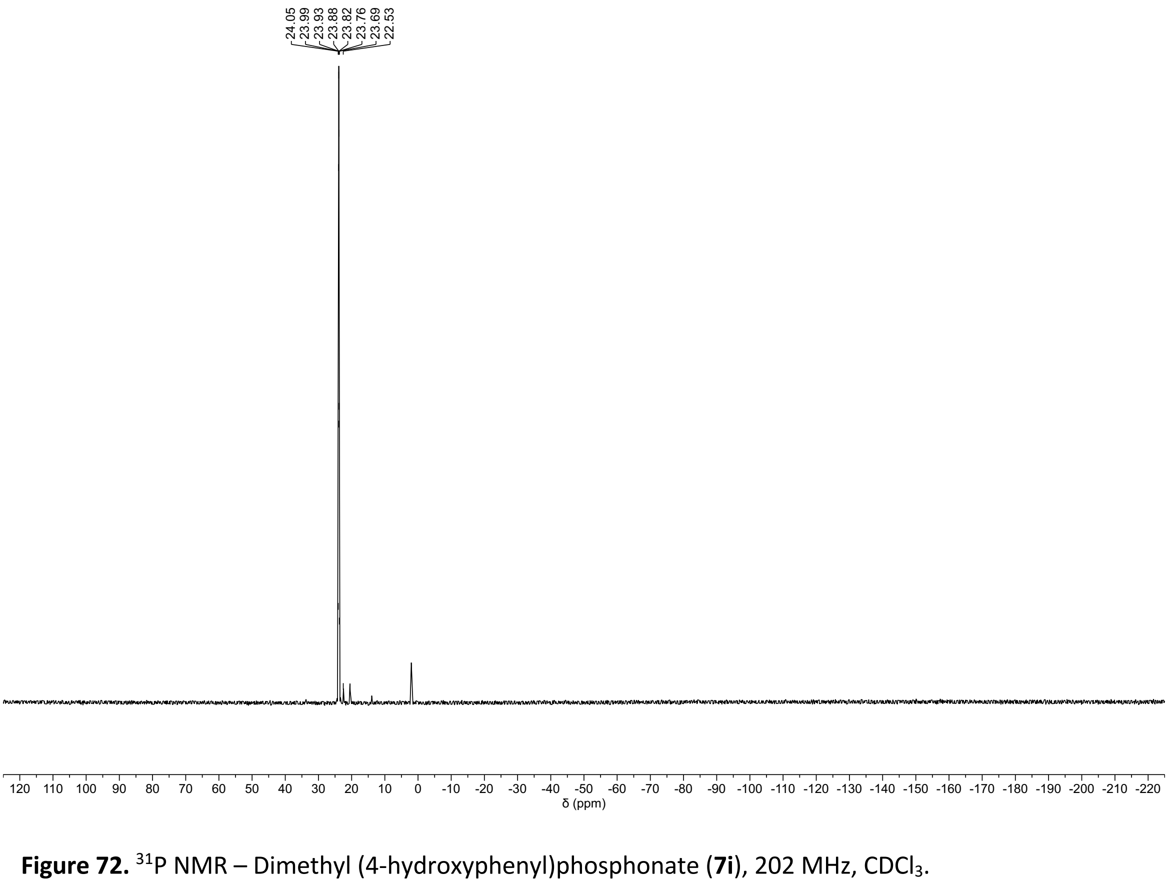

Figure 72. ^{31}P NMR – Dimethyl (4-hydroxyphenyl)phosphonate (**7i**), 202 MHz, $CDCl_3$.

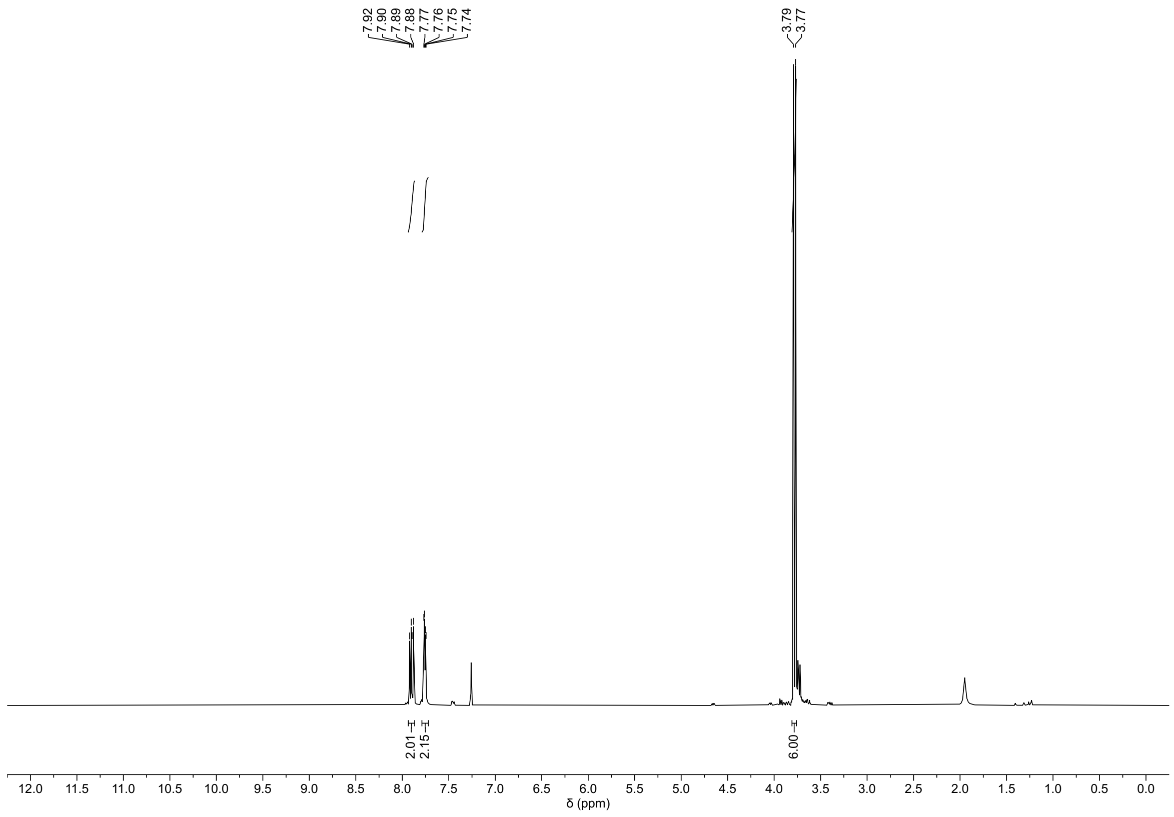

Figure 73. ^{1}H NMR – Dimethyl (4-cyanophenyl)phosphonate (**7j**), 500 MHz, $CDCl_3$.

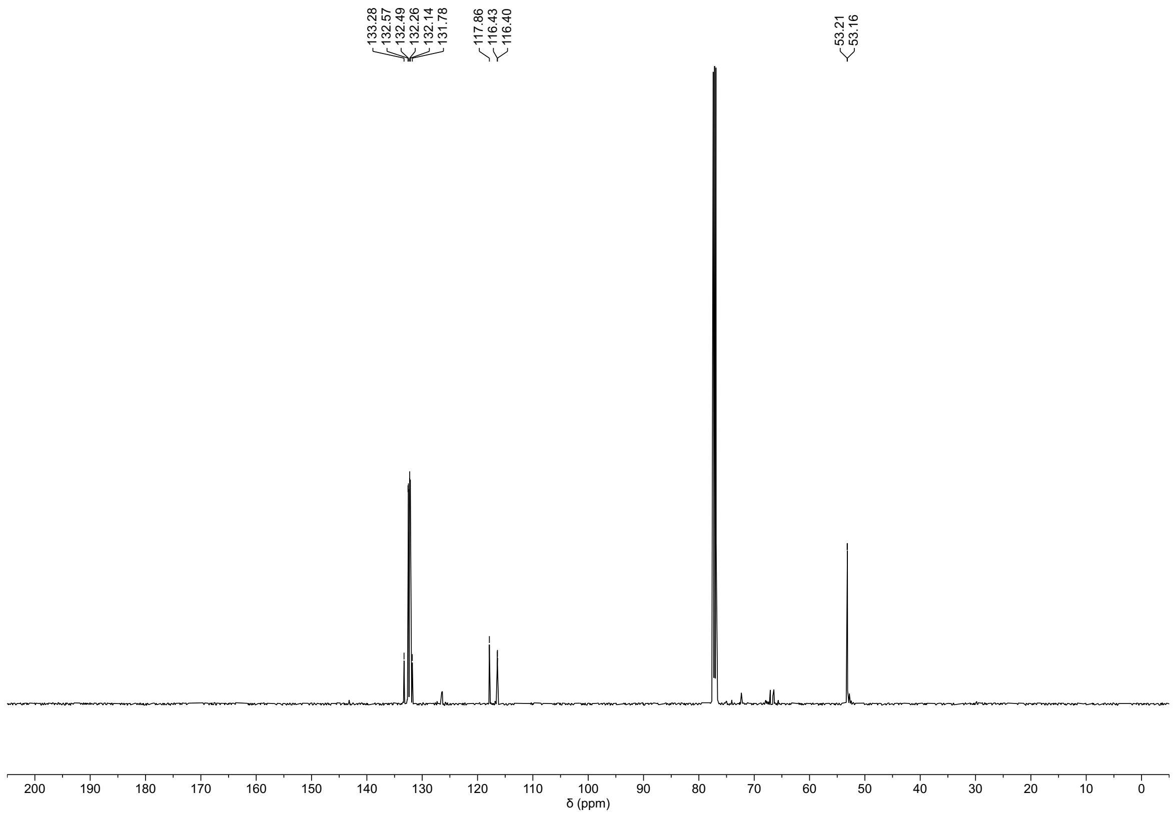

Figure 74. ^{13}C NMR – Dimethyl (4-cyanophenyl)phosphonate (**7j**), 126 MHz, $CDCl_3$.

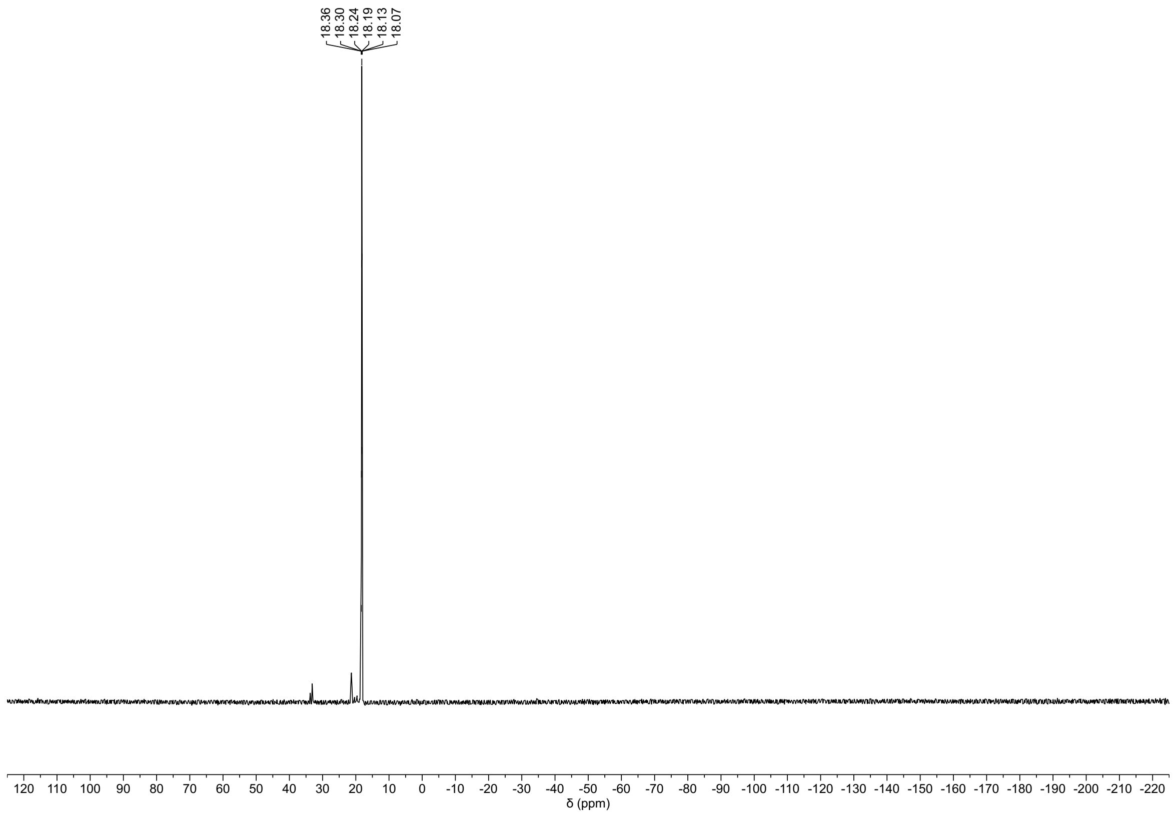

Figure 75. ^{31}P NMR – Dimethyl (4-cyanophenyl)phosphonate (**7j**), 202 MHz, $CDCl_3$.

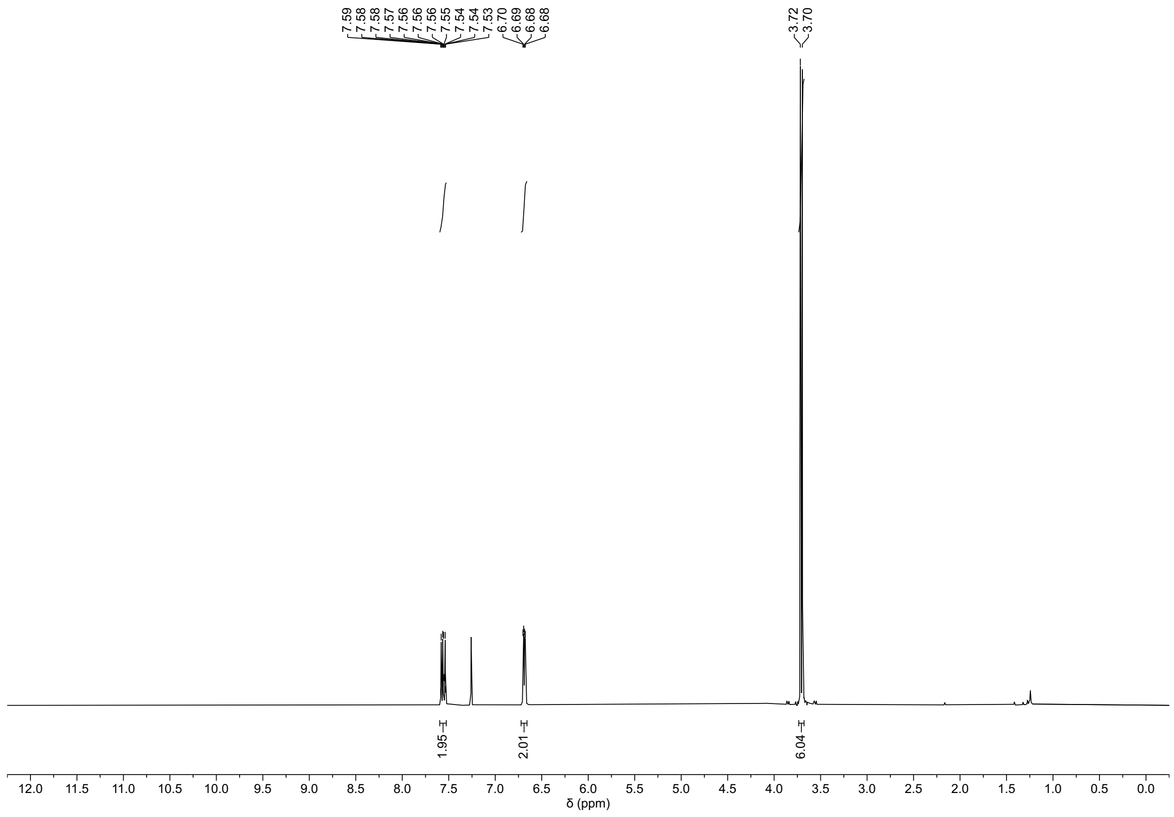

Figure 76. ^{1}H NMR – Dimethyl (4-aminophenyl)phosphonate (**7k**), 500 MHz, $CDCl_3$.

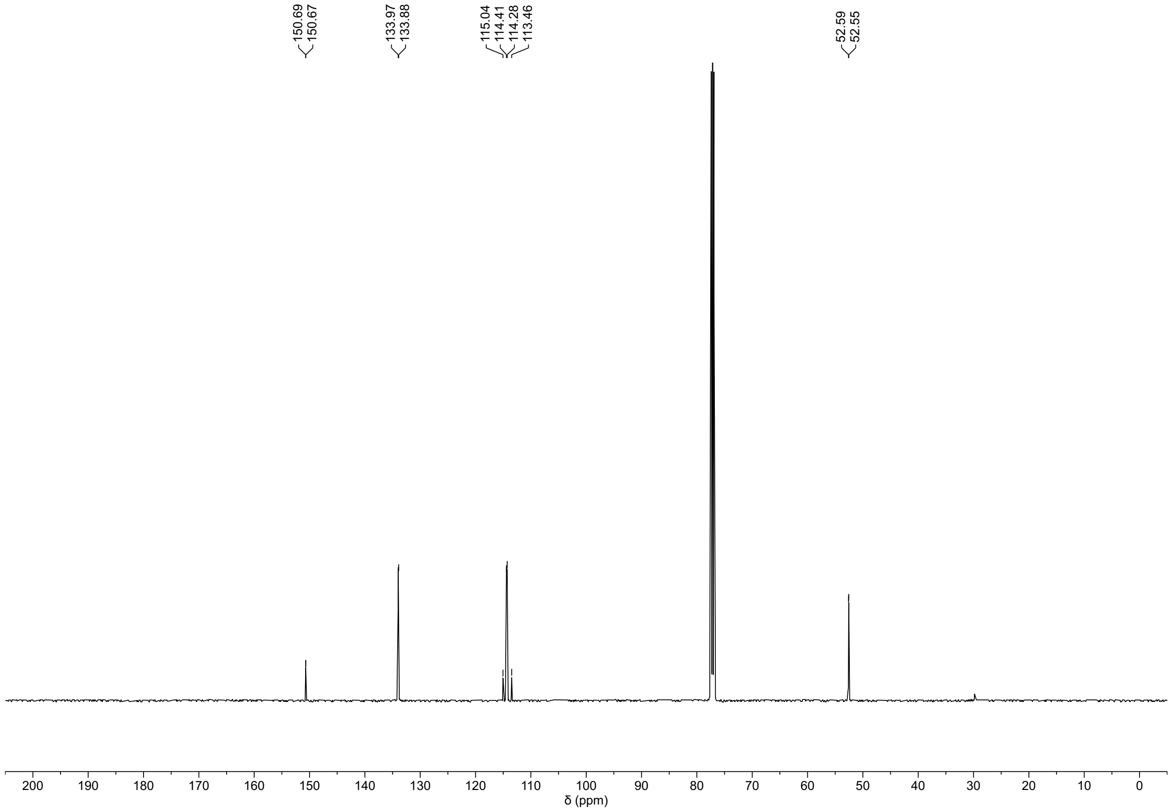

Figure 77. ^{13}C NMR – Dimethyl (4-aminophenyl)phosphonate (**7k**), 126 MHz, $CDCl_3$.

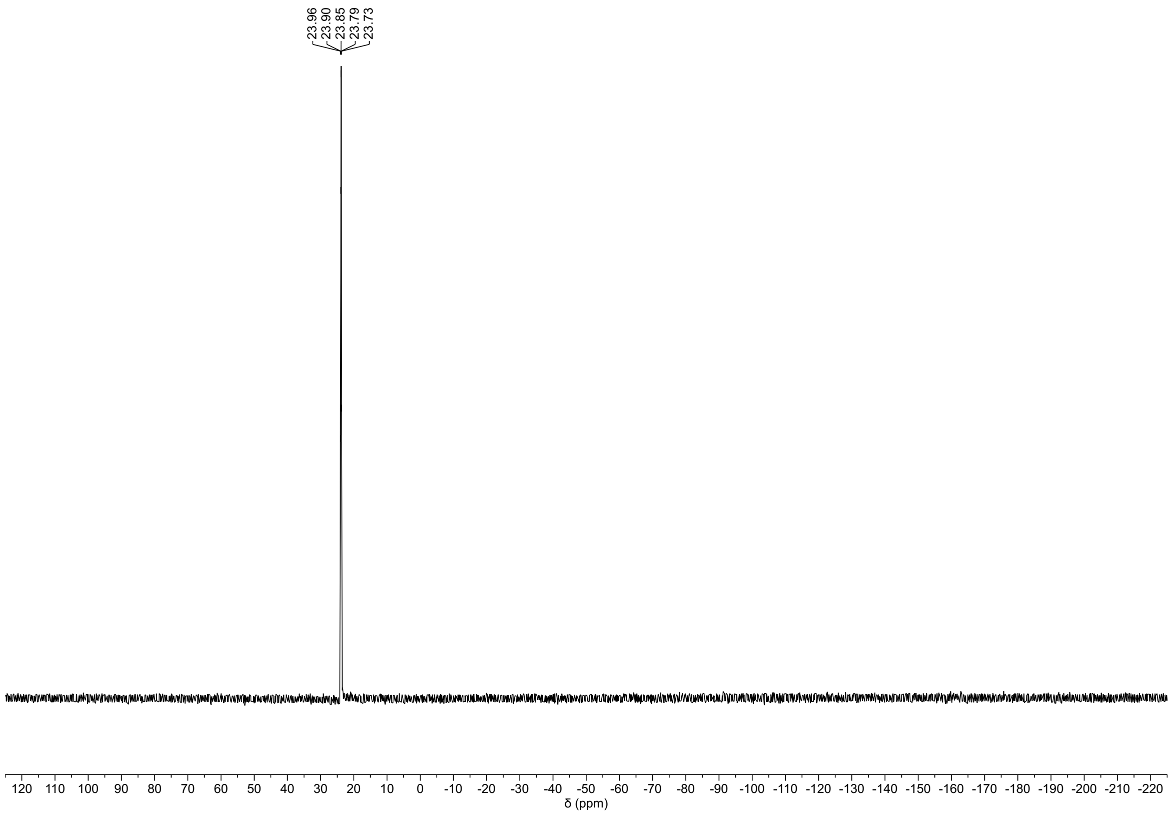

Figure 78. ^{31}P NMR – Dimethyl (4-aminophenyl)phosphonate (**7k**), 202 MHz, $CDCl_3$.

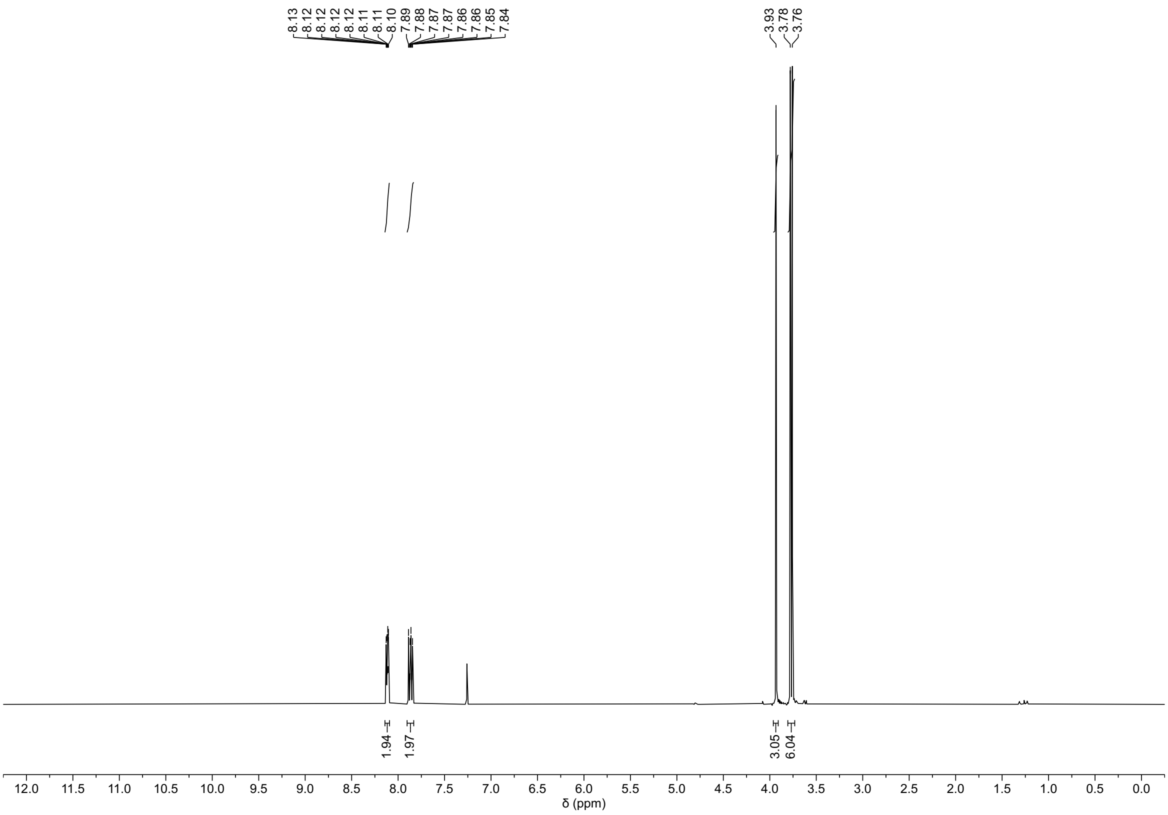

Figure 79. ^{1}H NMR – Methyl 4-(dimethoxyphosphoryl)benzoate (**7o**), 500 MHz, $CDCl_3$.

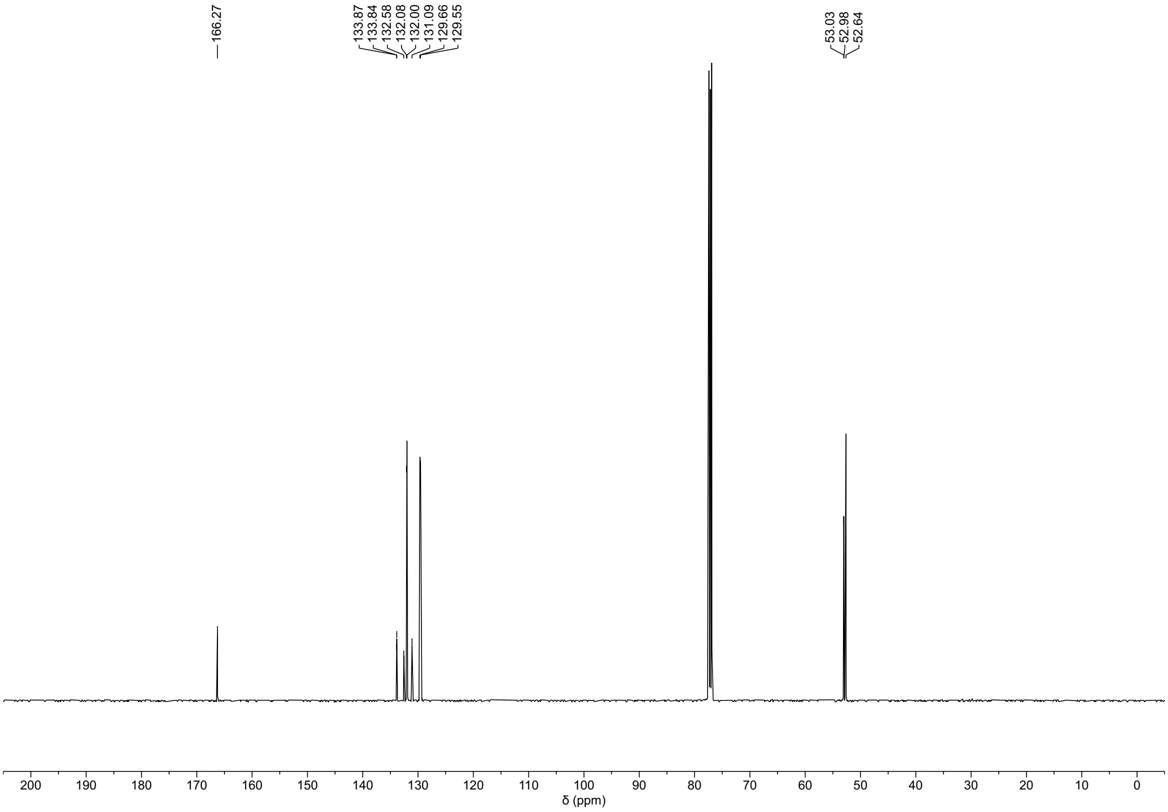

Figure 80. ^{13}C NMR – Methyl 4-(dimethoxyphosphoryl)benzoate (**7o**), 126 MHz, $CDCl_3$.

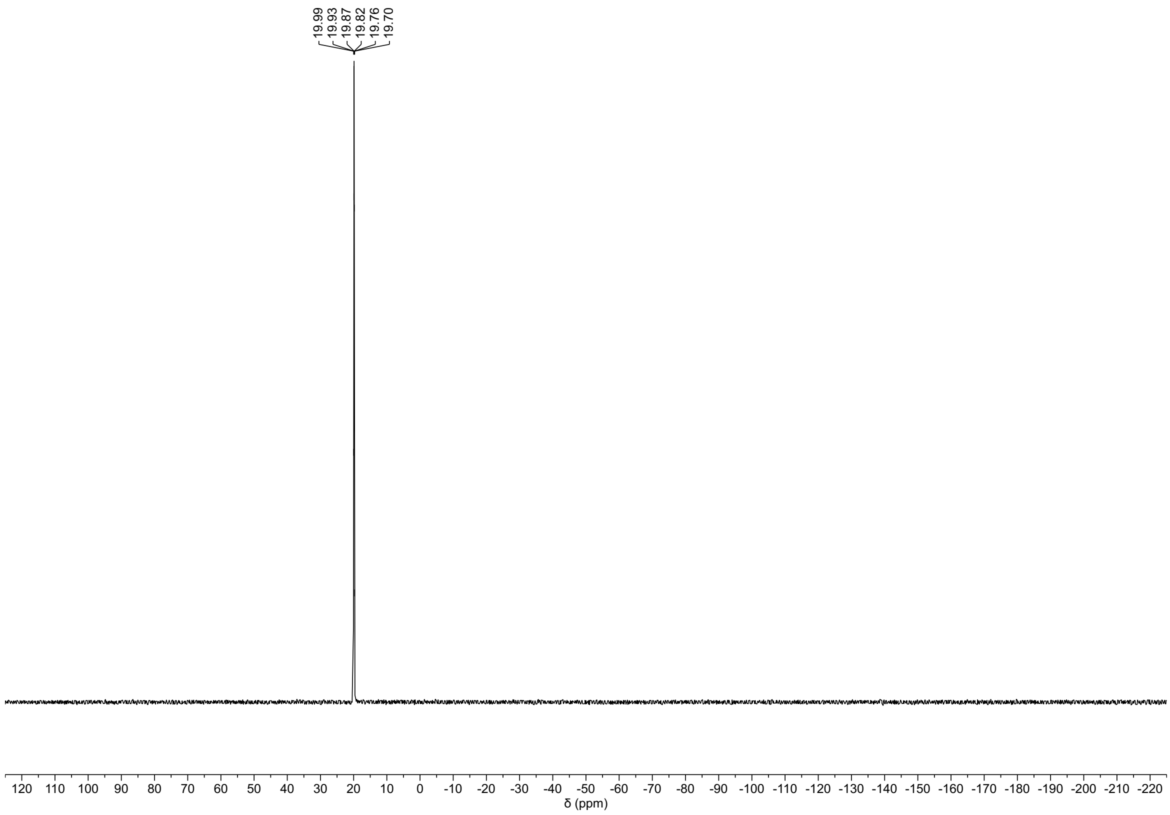

Figure 81. ^{31}P NMR – Methyl 4-(dimethoxyphosphoryl)benzoate (**7o**), 202 MHz, $CDCl_3$.

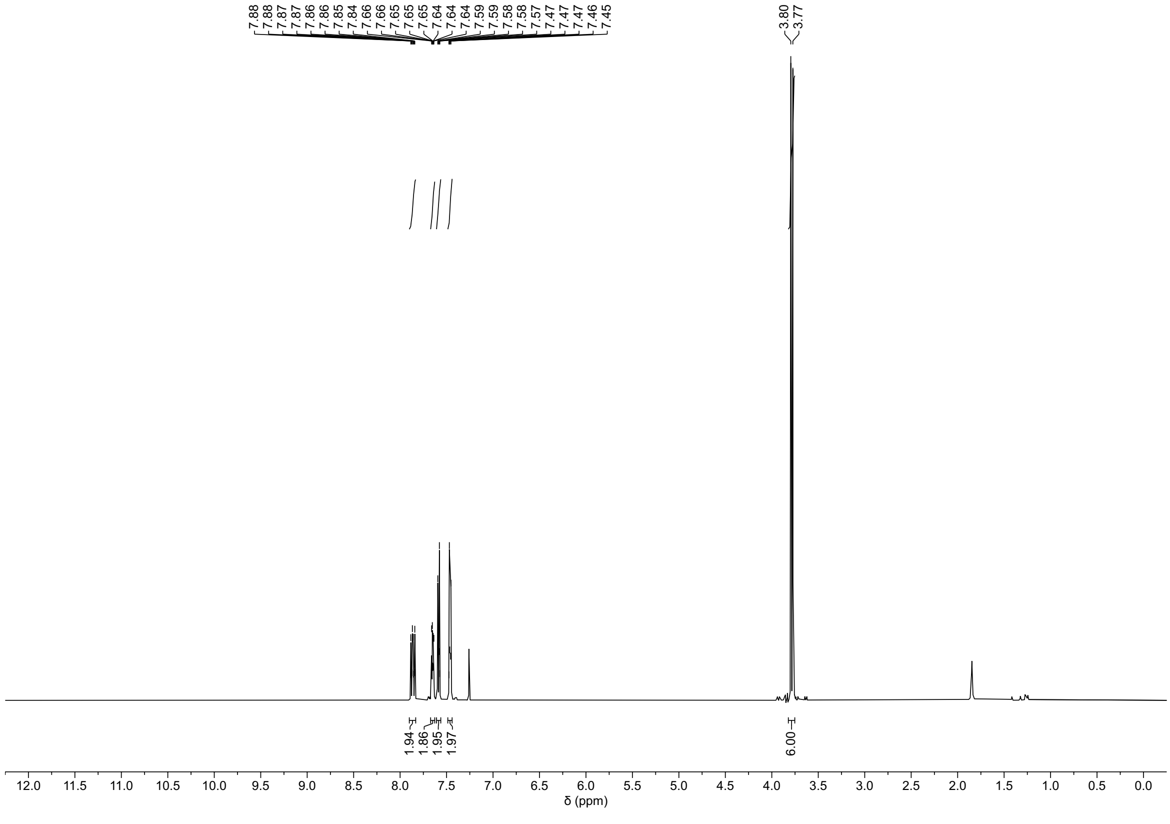

Figure 82. ^{1}H NMR – Dimethyl (4'-bromo-[1,1'-biphenyl]-4-yl)phosphonate (**7p**), 500 MHz, $CDCl_3$.

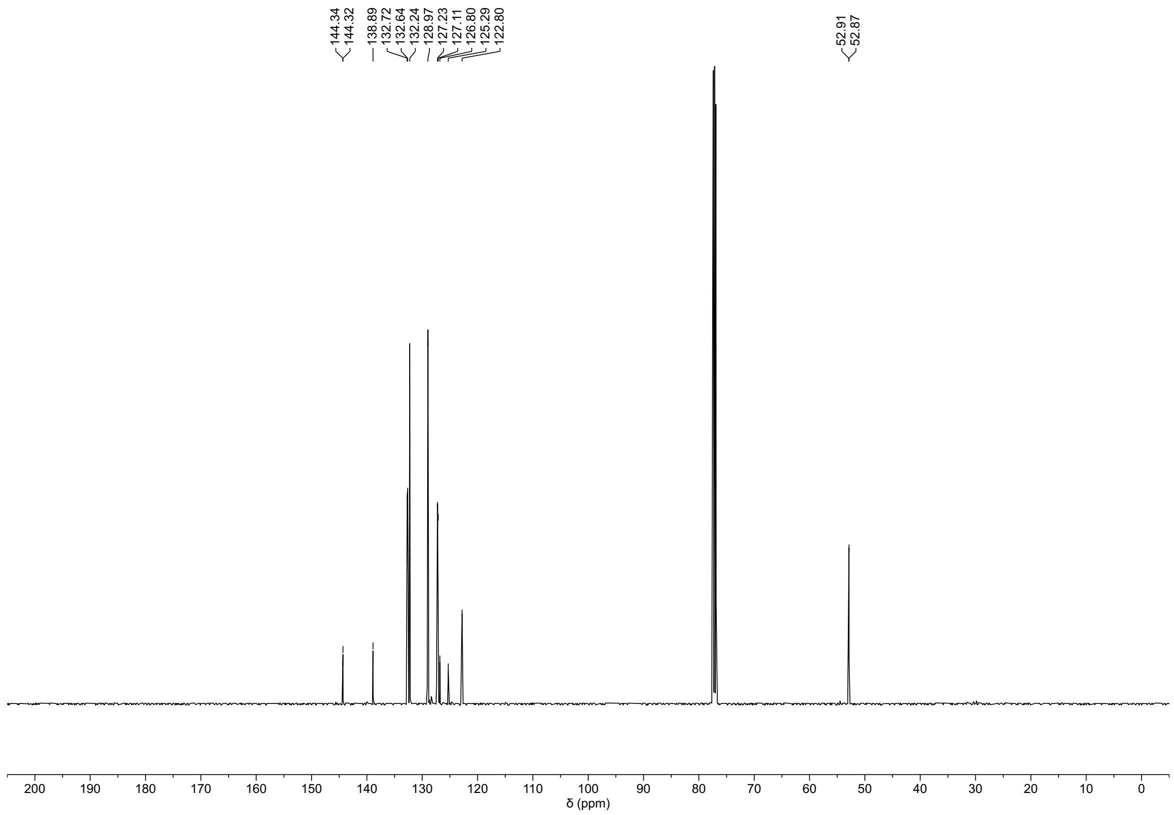

Figure 83. ^{13}C NMR – Dimethyl (4'-bromo-[1,1'-biphenyl]-4-yl)phosphonate (**7p**), 126 MHz, $CDCl_3$.

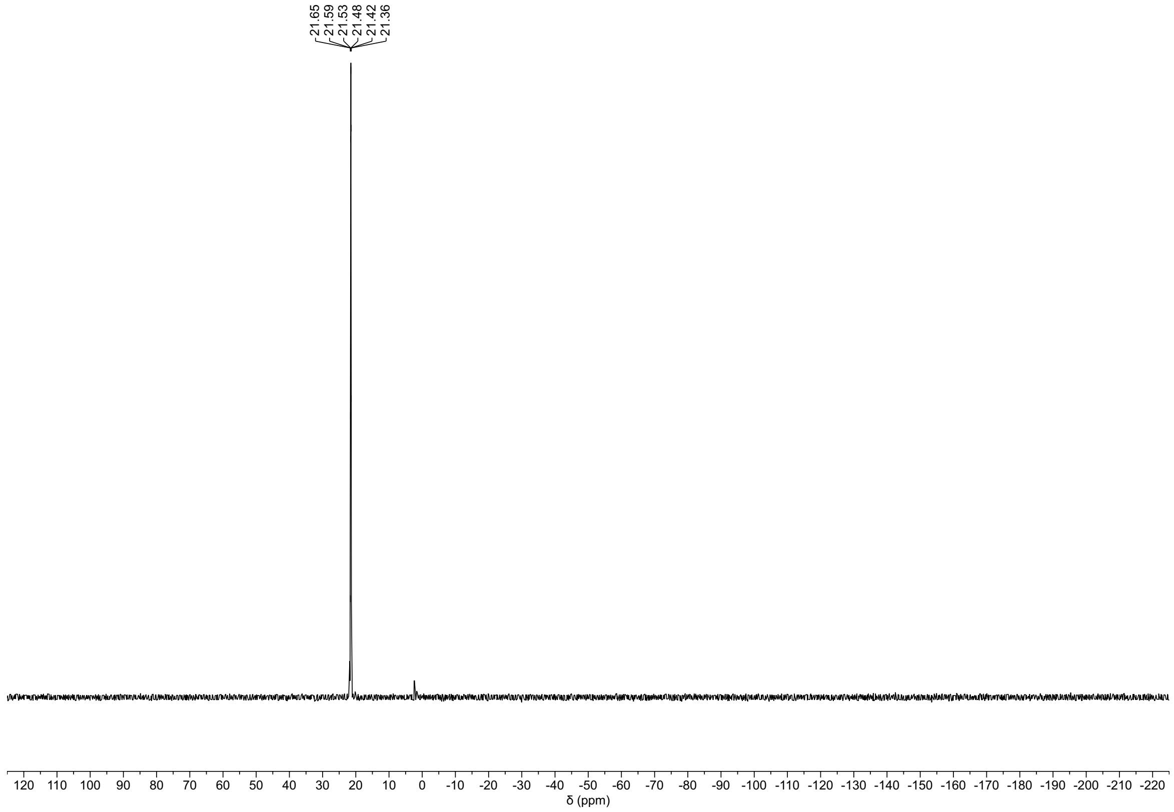

Figure 84. ^{31}P NMR – Dimethyl (4'-bromo-[1,1'-biphenyl]-4-yl)phosphonate (**7p**), 202 MHz, $CDCl_3$.

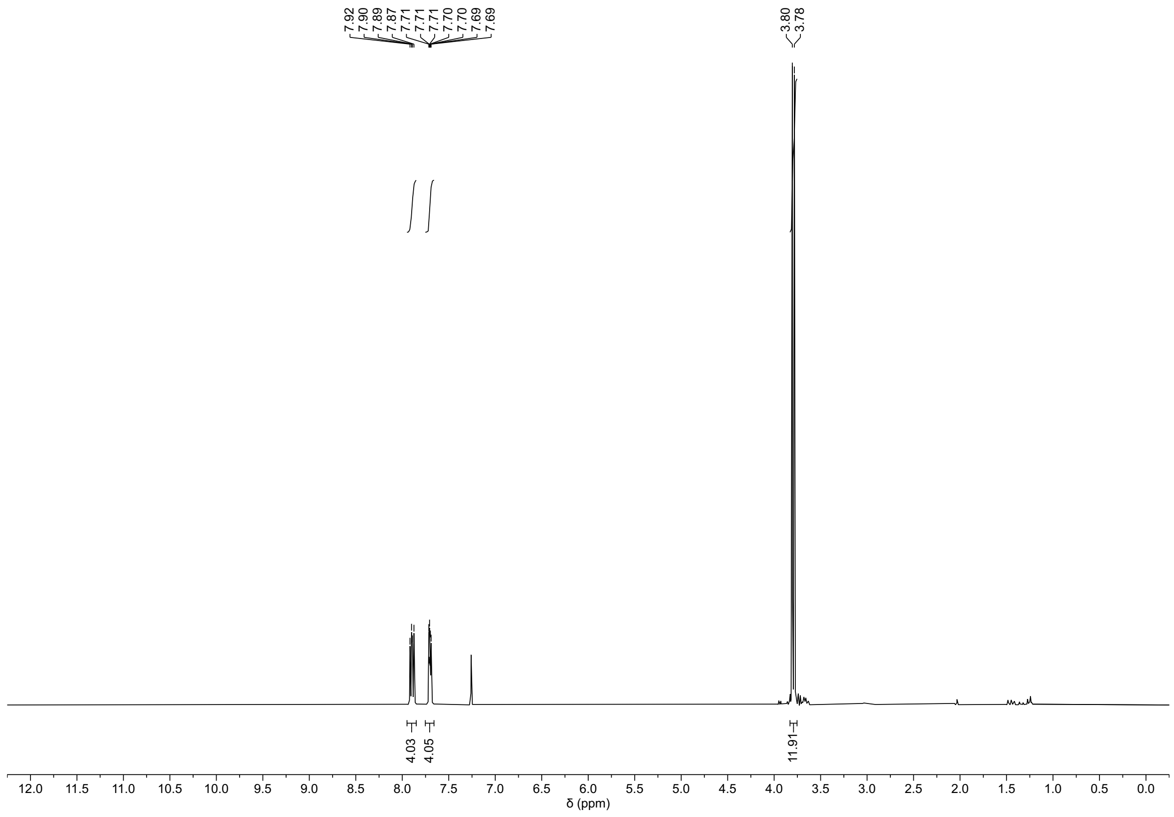

Figure 85. ^{1}H NMR – Tetramethyl [1,1'-biphenyl]-4,4'-diylbis(phosphonate) (**7p'**), 500 MHz, $CDCl_3$.

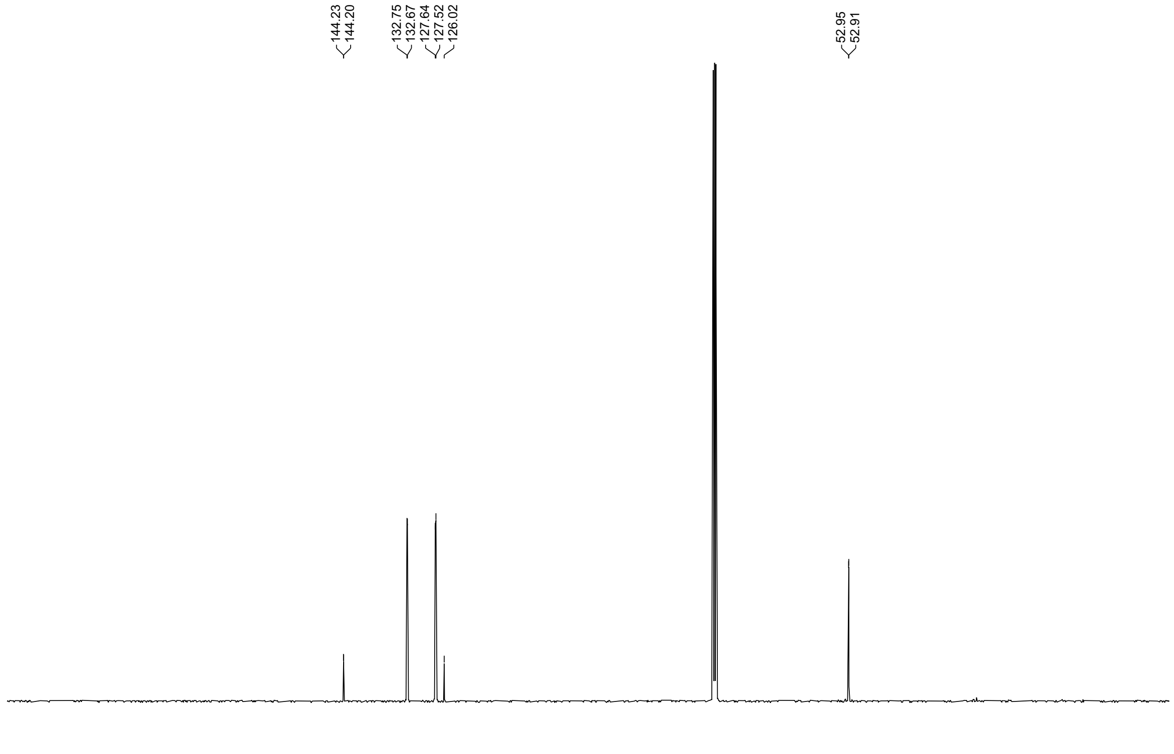

Figure 86. ^{13}C NMR – Tetramethyl [1,1'-biphenyl]-4,4'-diylbis(phosphonate) (**7p'**), 126 MHz, $CDCl_3$.

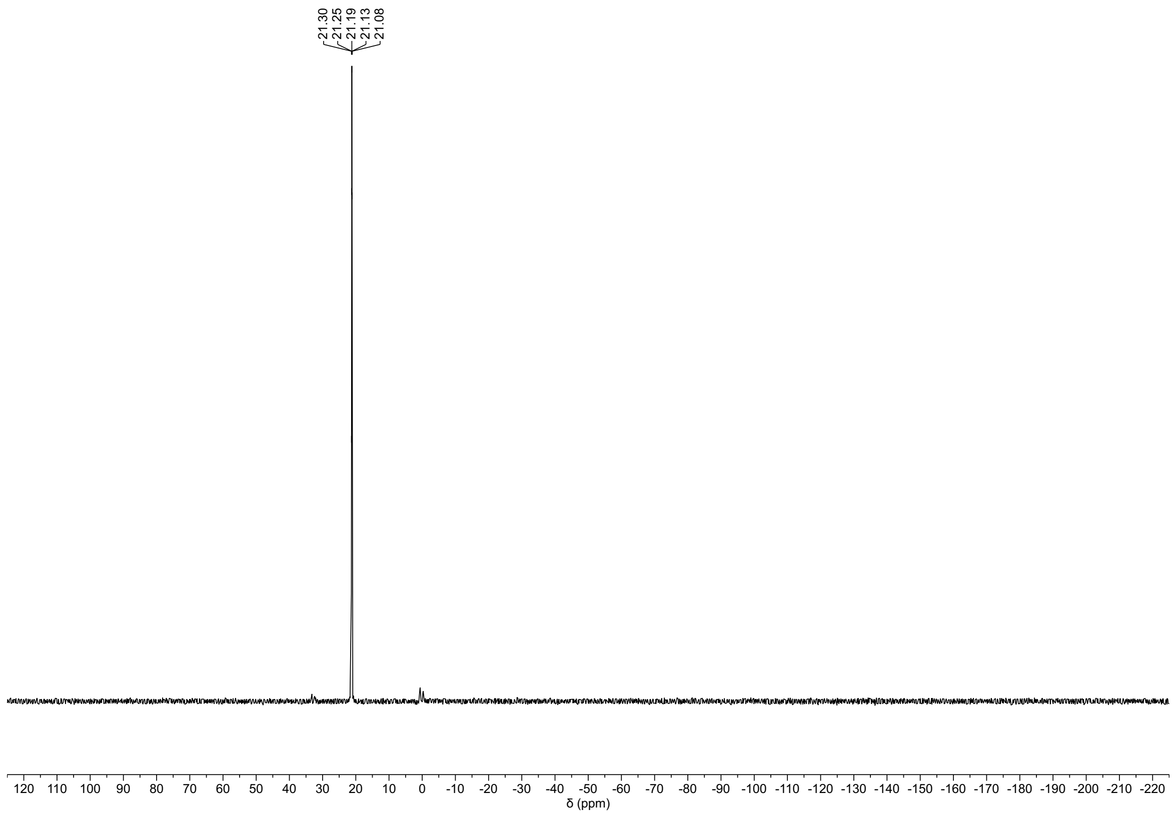

Figure 87. ^{31}P NMR – Tetramethyl [1,1'-biphenyl]-4,4'-diylbis(phosphonate) (**7p'**), 202 MHz, $CDCl_3$.

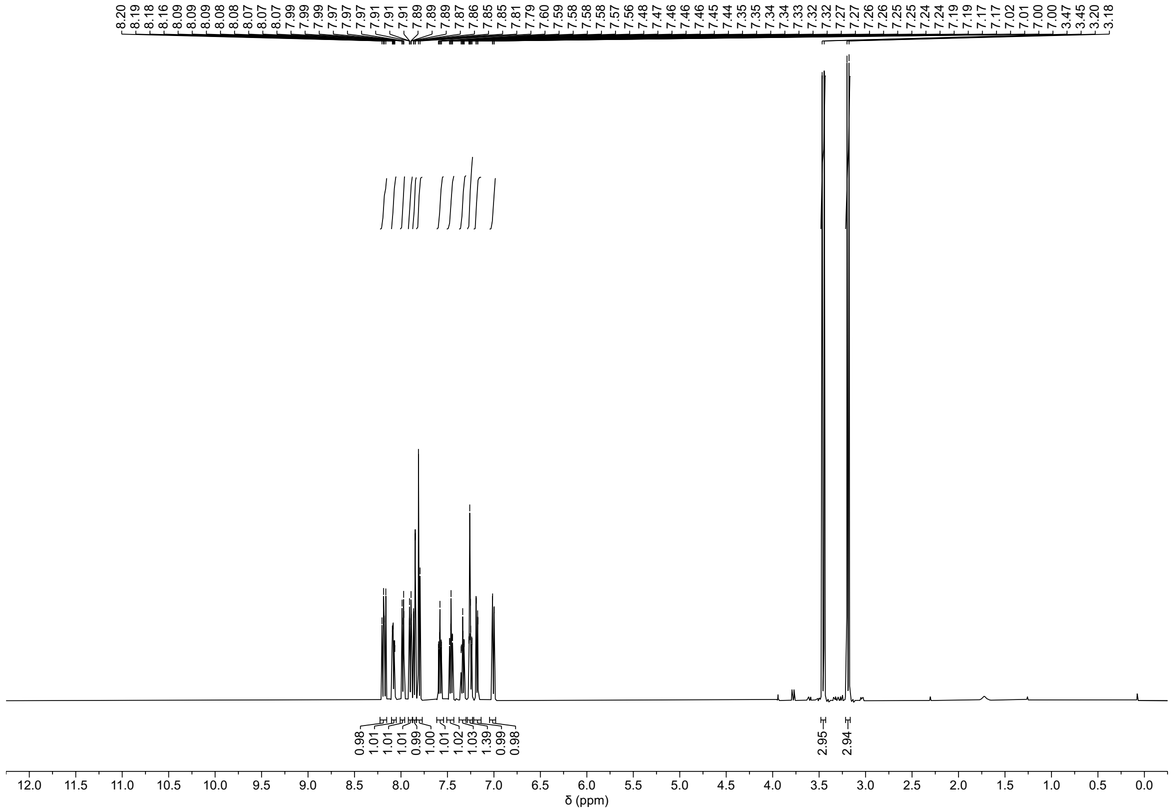

Figure 88. ^{1}H NMR – Dimethyl (7'-bromo-[1,1'-binaphthalen]-7-yl)phosphonate (**7q**), 500 MHz, $CDCl_3$.

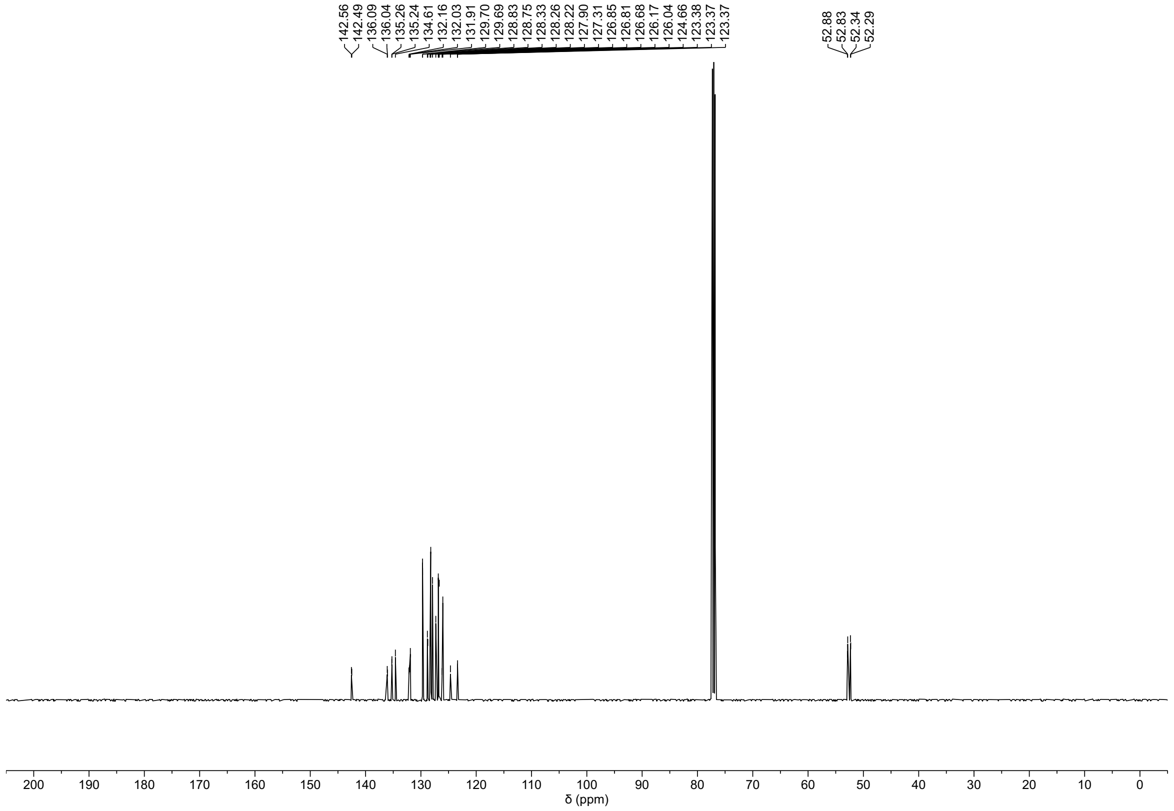

Figure 89. ^{13}C NMR – Dimethyl (7'-bromo-[1,1'-binaphthalen]-7-yl)phosphonate (**7q**), 126 MHz, $CDCl_3$.

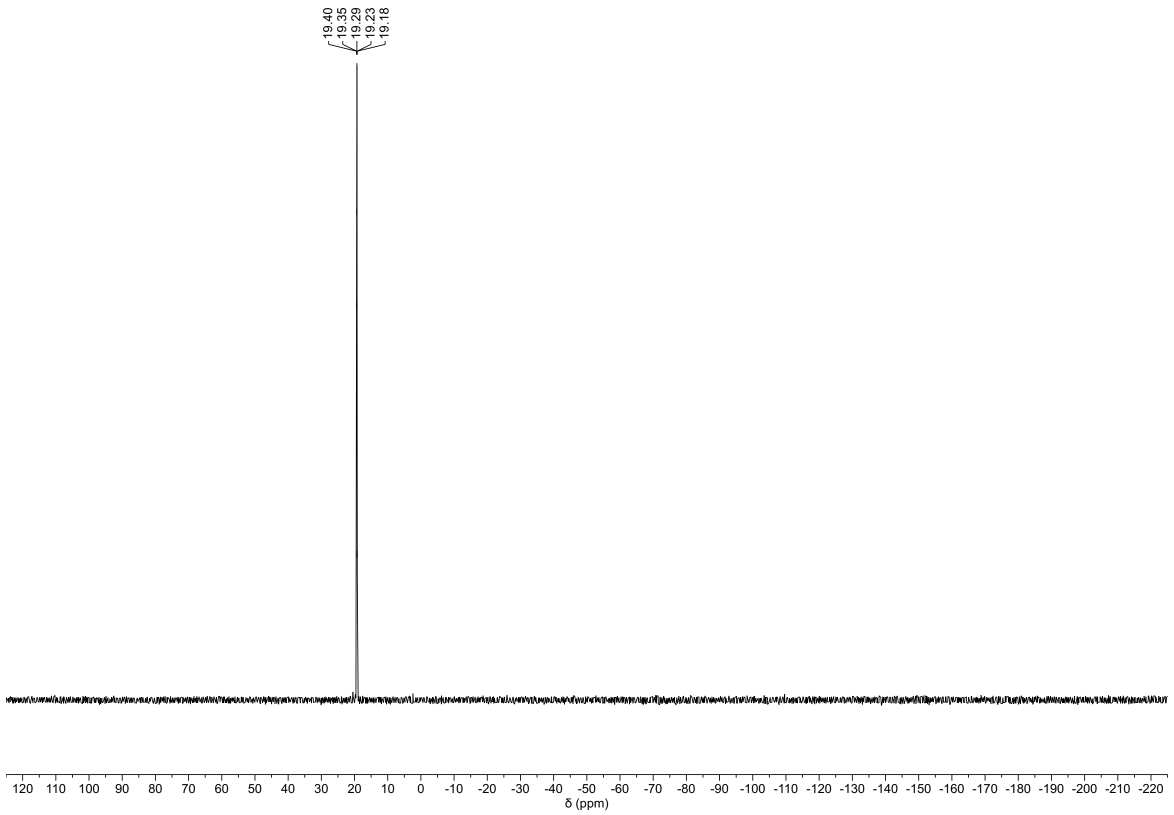

Figure 90. ^{31}P NMR – Dimethyl (7'-bromo-[1,1'-binaphthalen]-7-yl)phosphonate (**7q**), 202 MHz, $CDCl_3$.

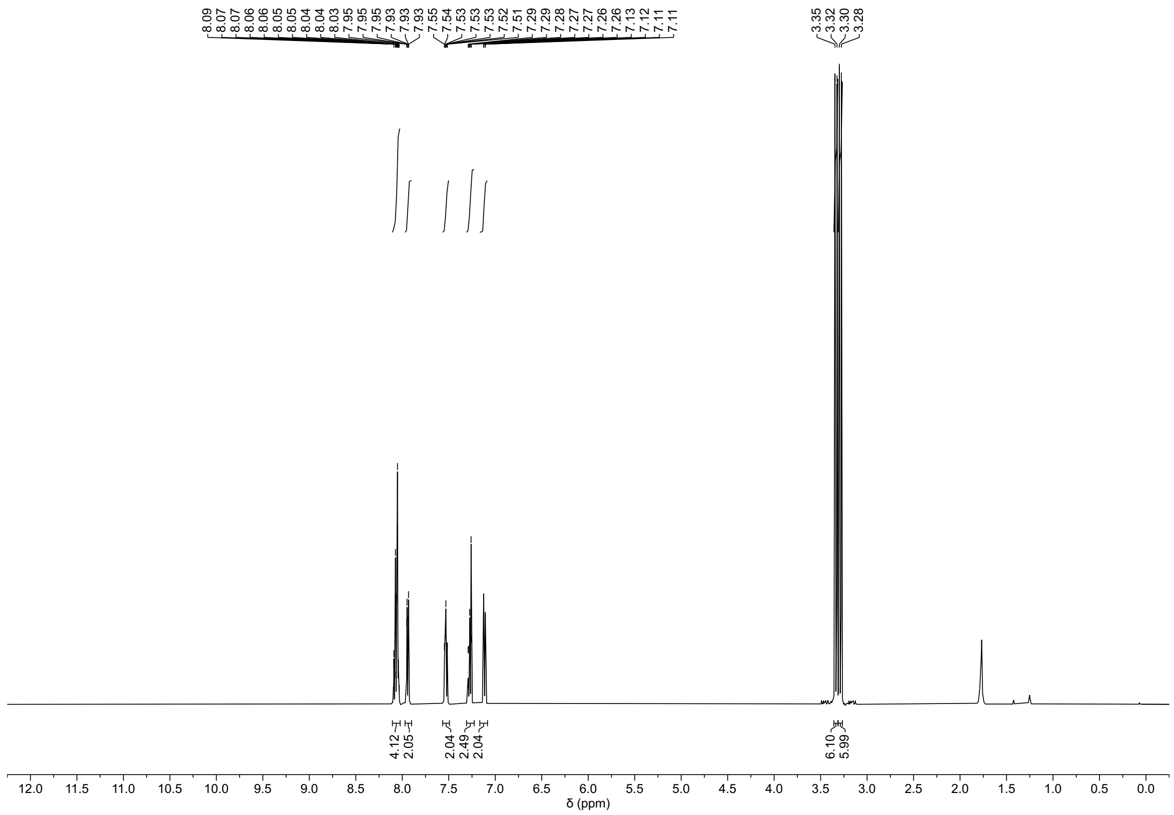

Figure 91. ^{1}H NMR – Tetramethyl [1,1'-binaphthalene]-7,7'-diylbis(phosphonate) (**7q'**), 500 MHz, $CDCl_3$.

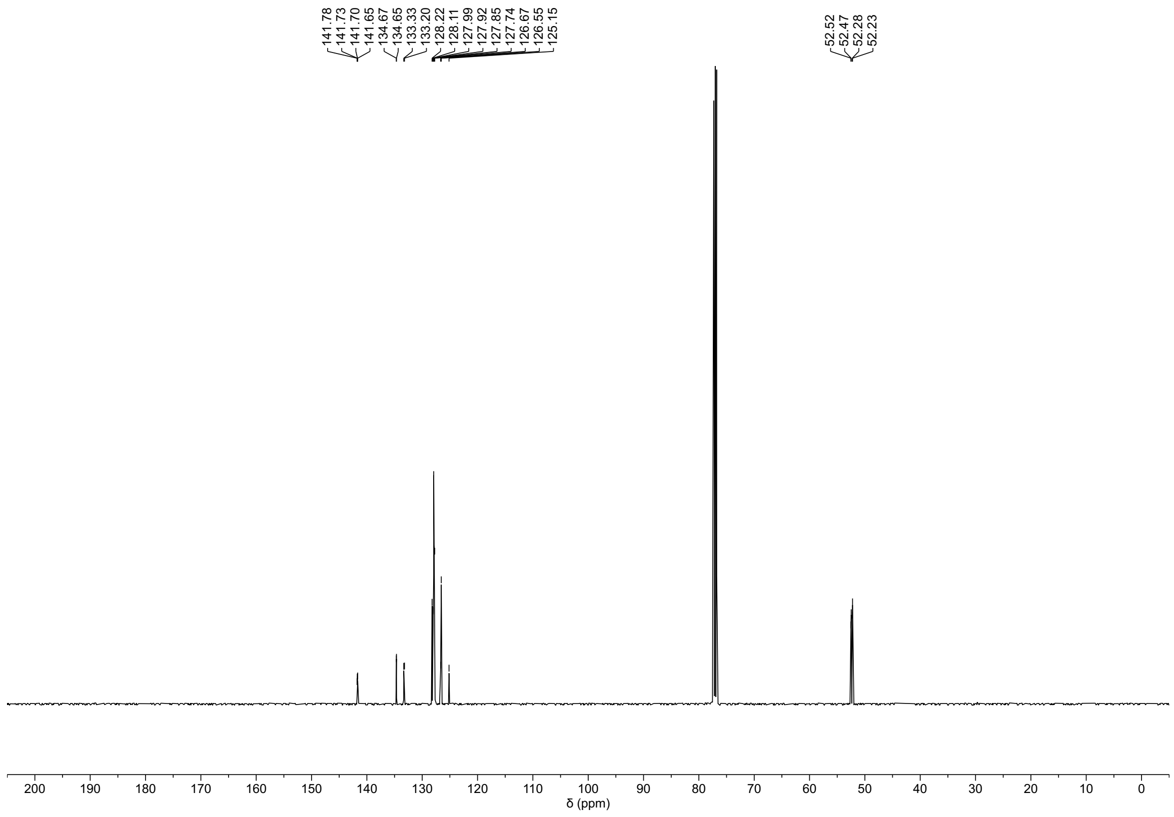

Figure 92. ^{13}C NMR – Tetramethyl [1,1'-binaphthalene]-7,7'-diylbis(phosphonate) (**7q'**), 126 MHz, $CDCl_3$.

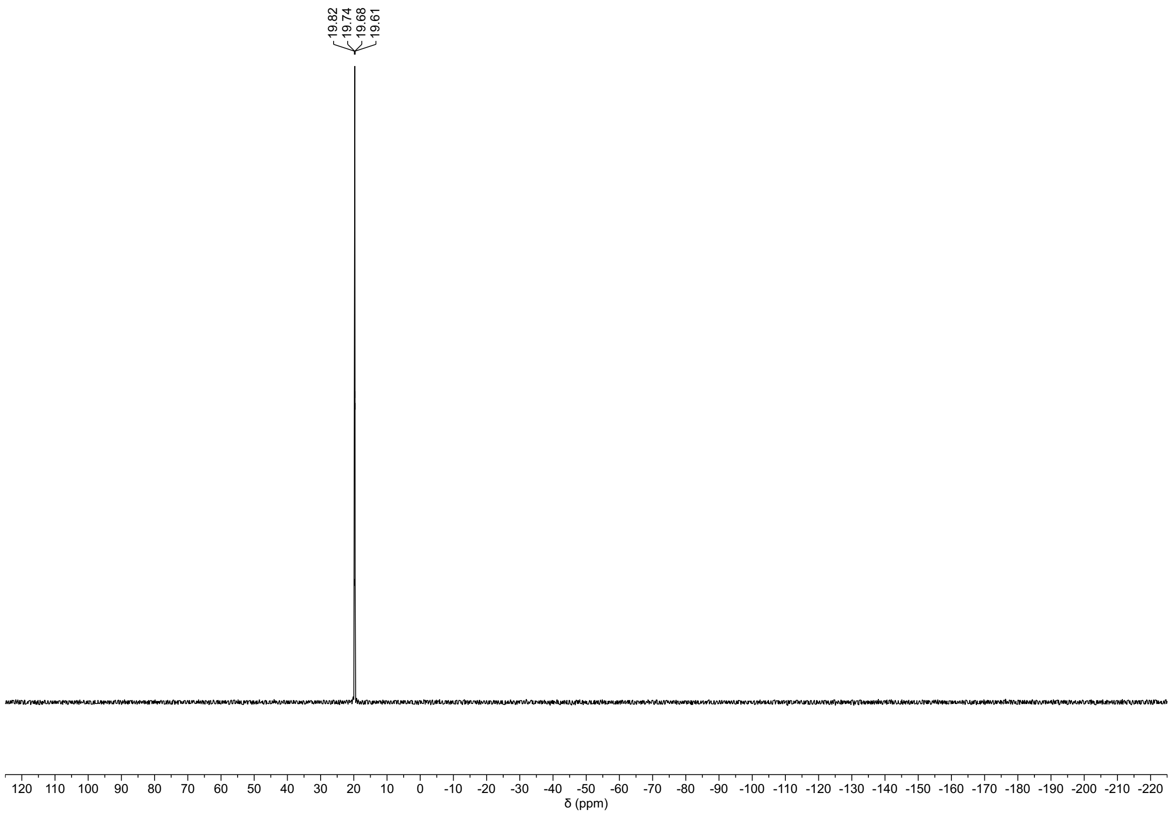

Figure 93. ^{31}P NMR – Tetramethyl [1,1'-binaphthalene]-7,7'-diylbis(phosphonate) (**7q'**), 202 MHz, $CDCl_3$.

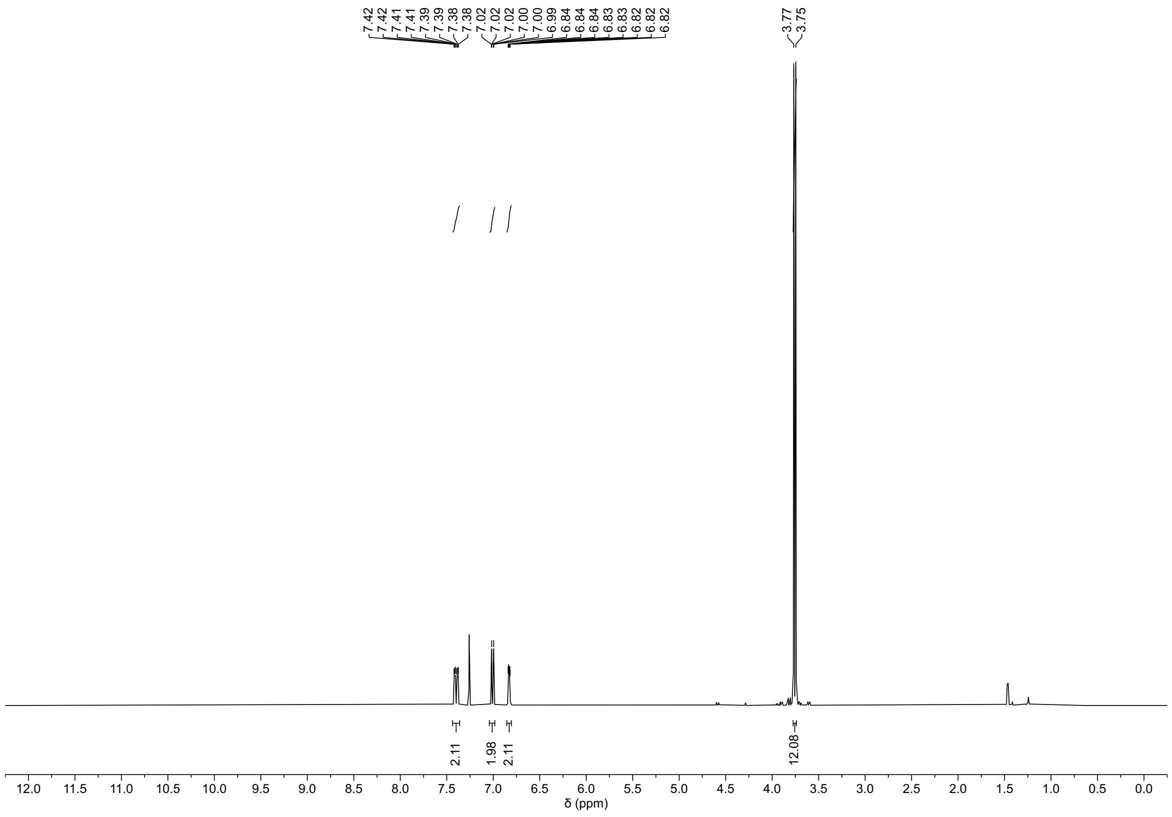

Figure 94. ^{1}H NMR – Tetramethyl biphenylene-2,7-diylbis(phosphonate) (**7r**), 500 MHz, $CDCl_3$.

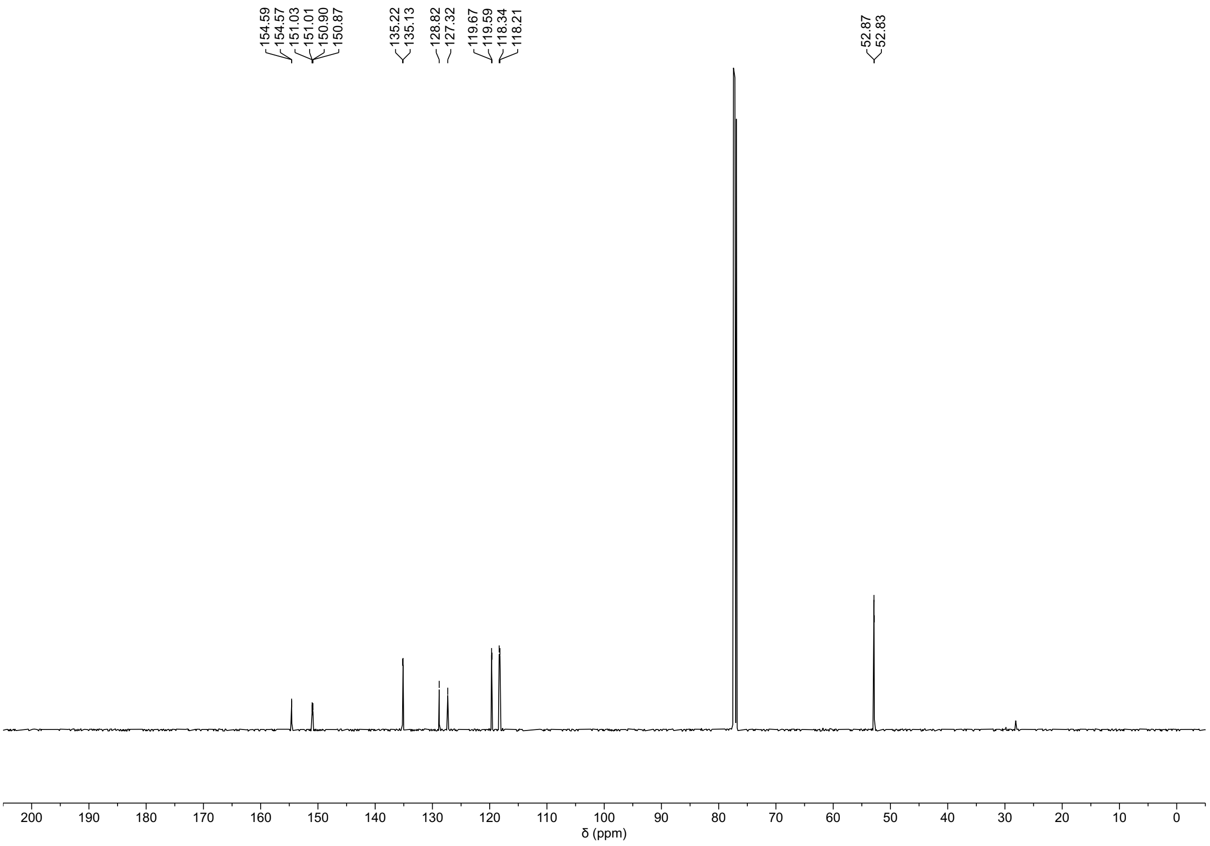

Figure 95. ^{13}C NMR – Tetramethyl biphenylene-2,7-diylbis(phosphonate) (**7r**), 126 MHz, $CDCl_3$.

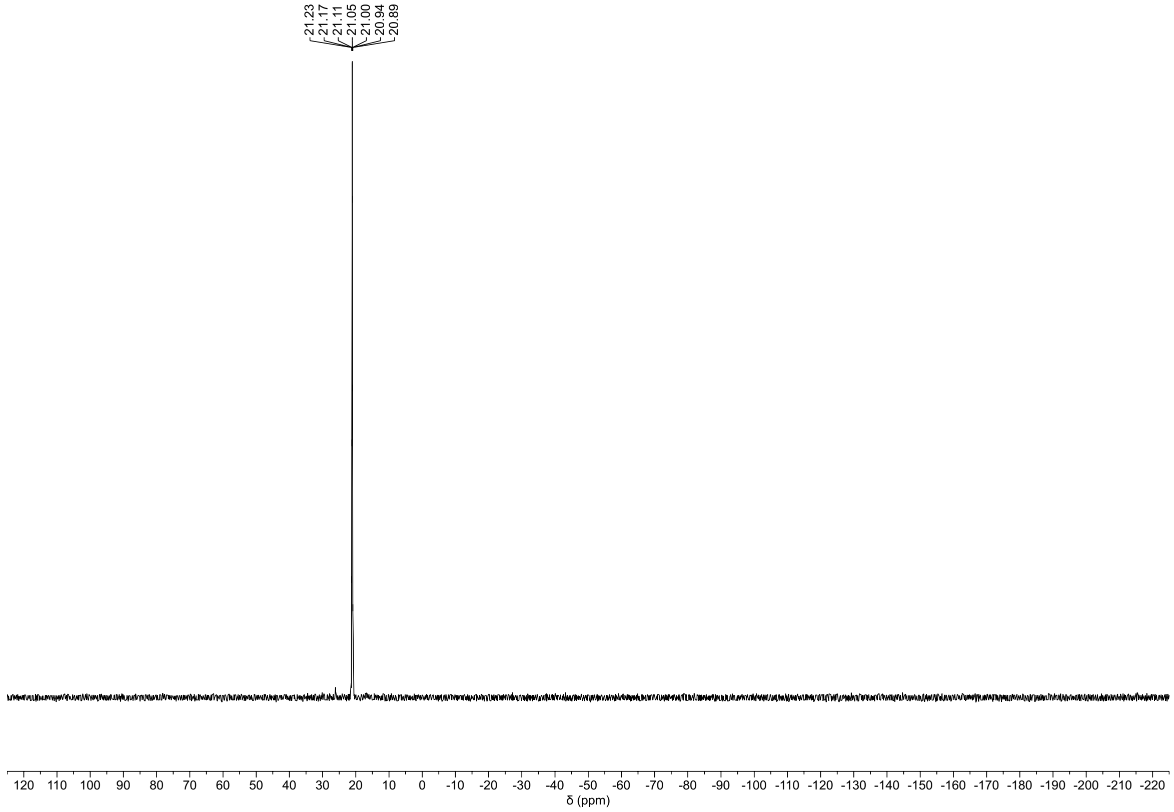

Figure 96. ^{31}P NMR – Tetramethyl biphenylene-2,7-diylbis(phosphonate) (**7r**), 202 MHz, $CDCl_3$.

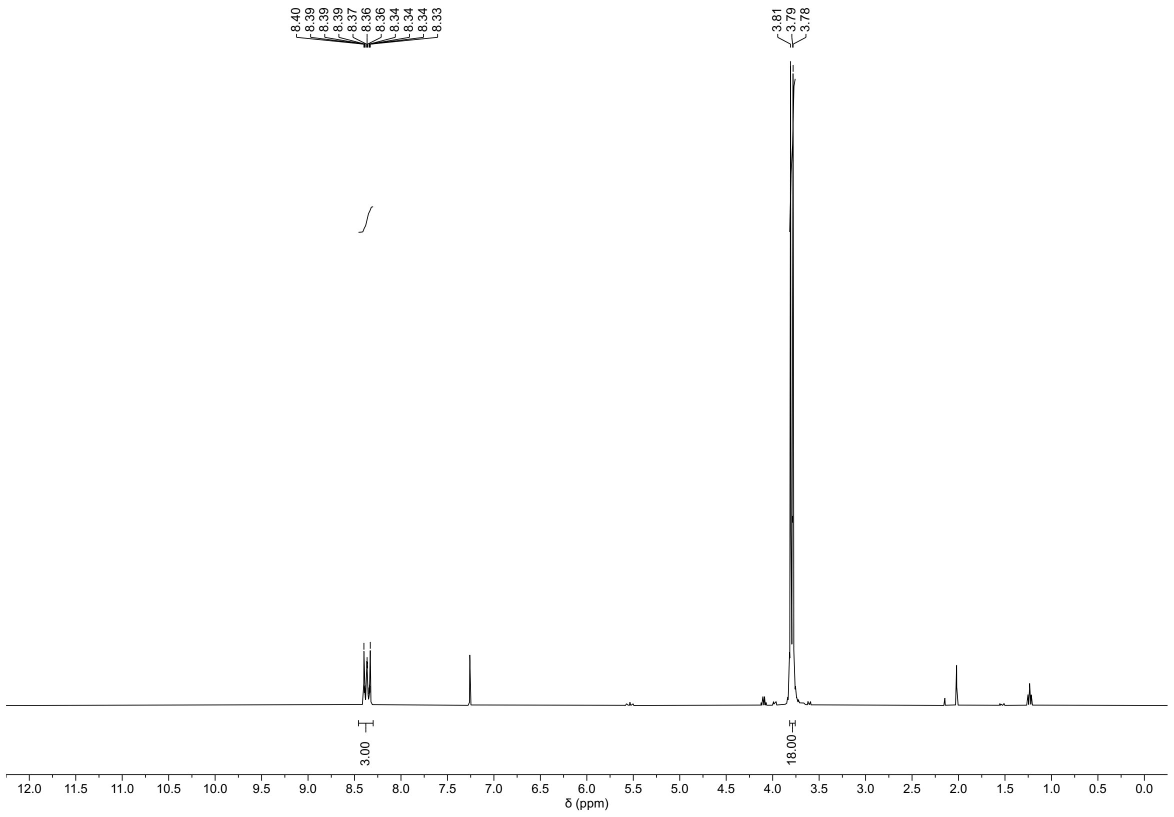

Figure 97. ^{1}H NMR – Hexamethyl benzene-1,3,5-triyltris(phosphonate) (**4s**), 400 MHz, $CDCl_3$.

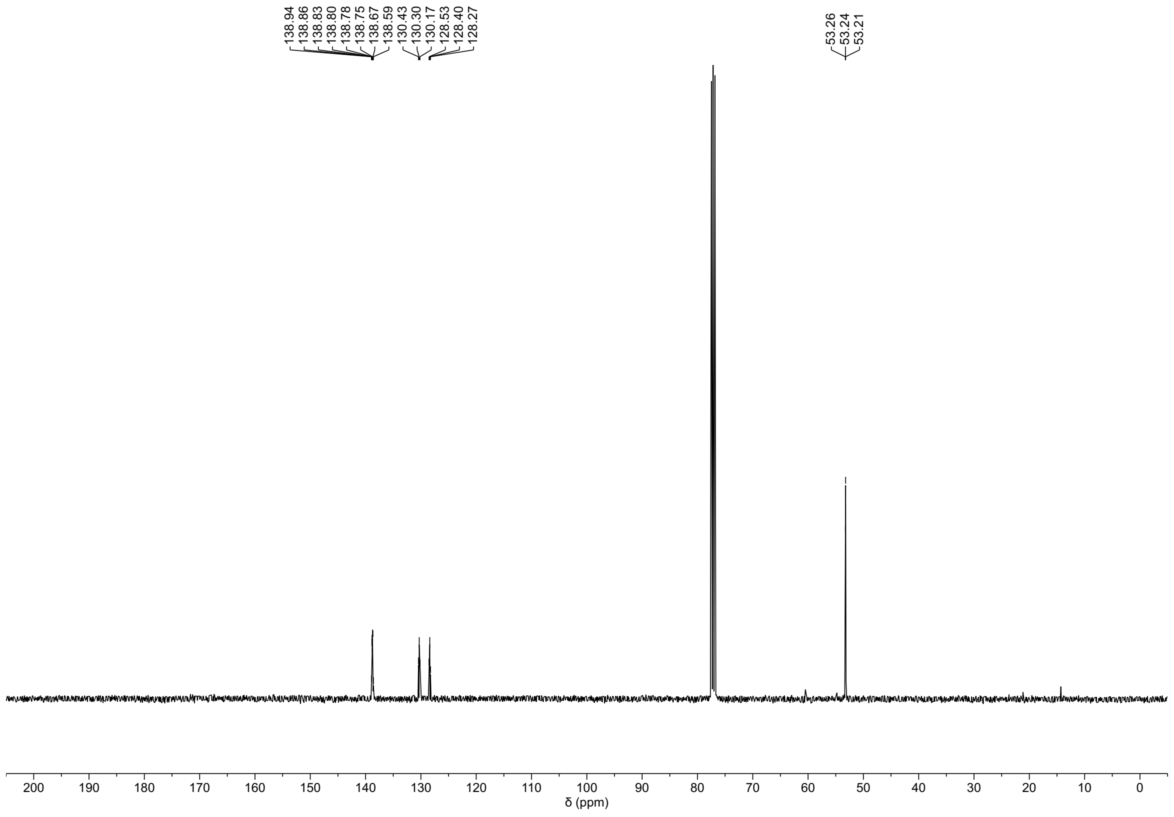

Figure 98. ^{13}C NMR – Hexamethyl benzene-1,3,5-triyltris(phosphonate) (**7s**), 101 MHz, $CDCl_3$.

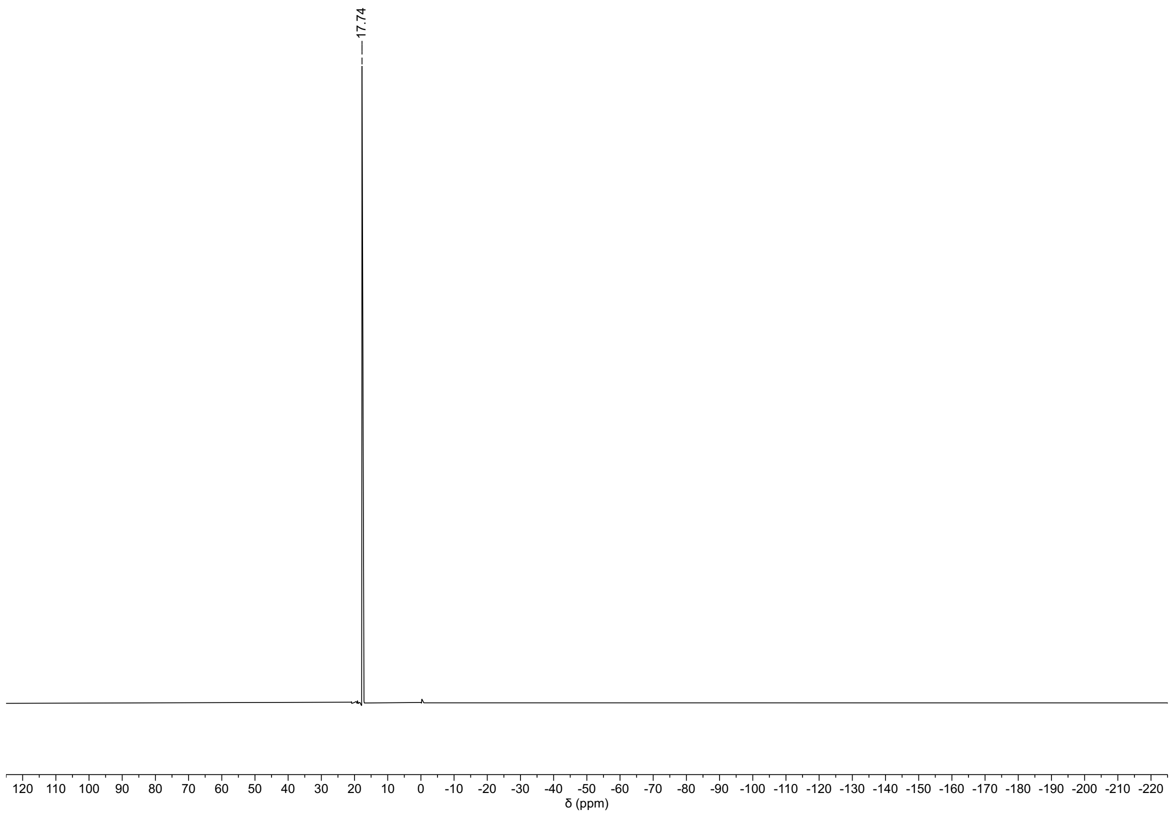

Figure 99. ^{31}P NMR – Hexamethyl benzene-1,3,5-triyltris(phosphonate) (**7s**), 162 MHz, $CDCl_3$.

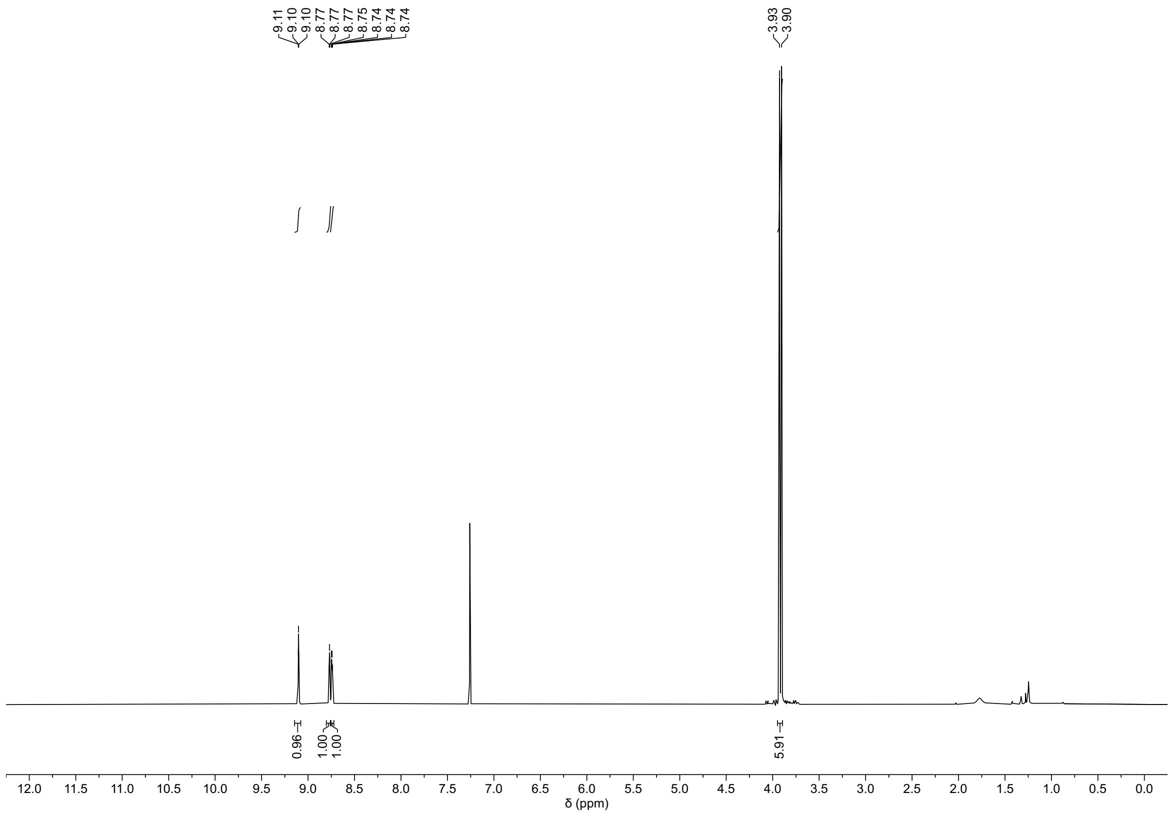

Figure 100. ^{1}H NMR – Dimethyl pyrazin-2-ylphosphonate (**7t**), 500 MHz, $CDCl_3$.

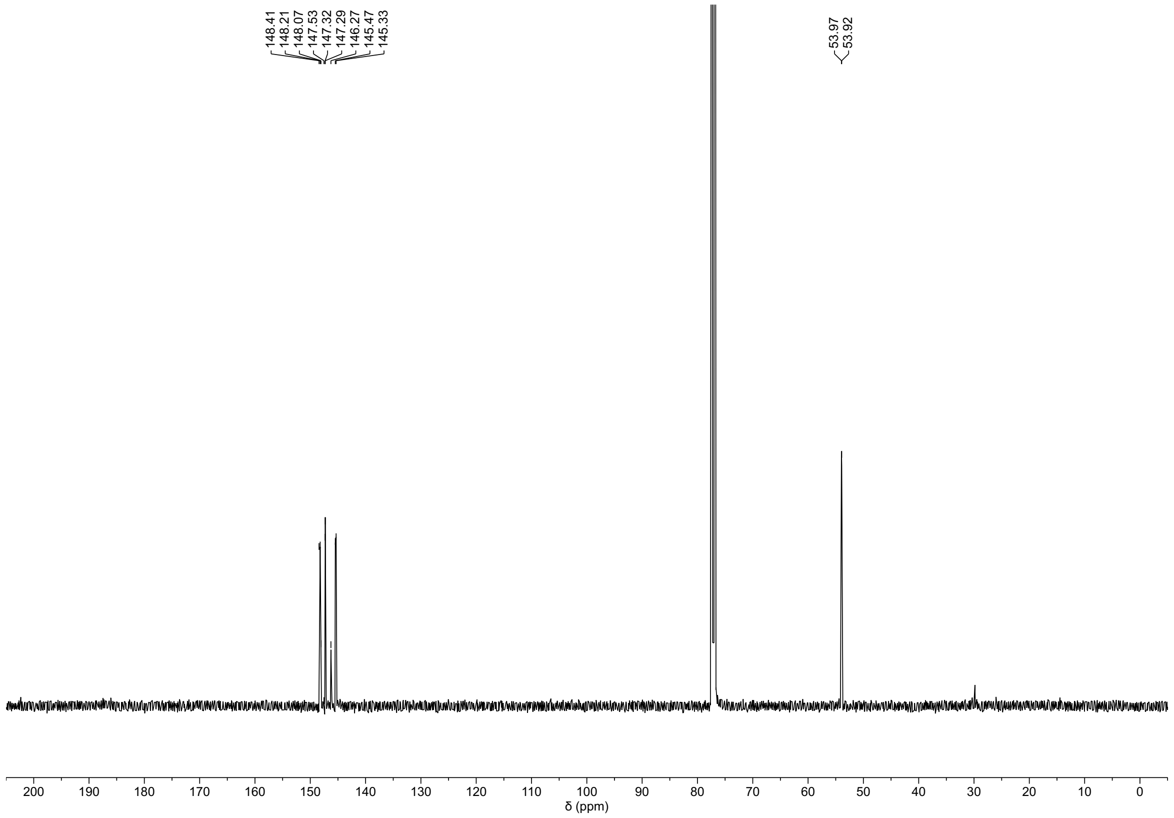

Figure 101. ^{13}C NMR – Dimethyl pyrazin-2-ylphosphonate (**7t**), 126 MHz, $CDCl_3$.

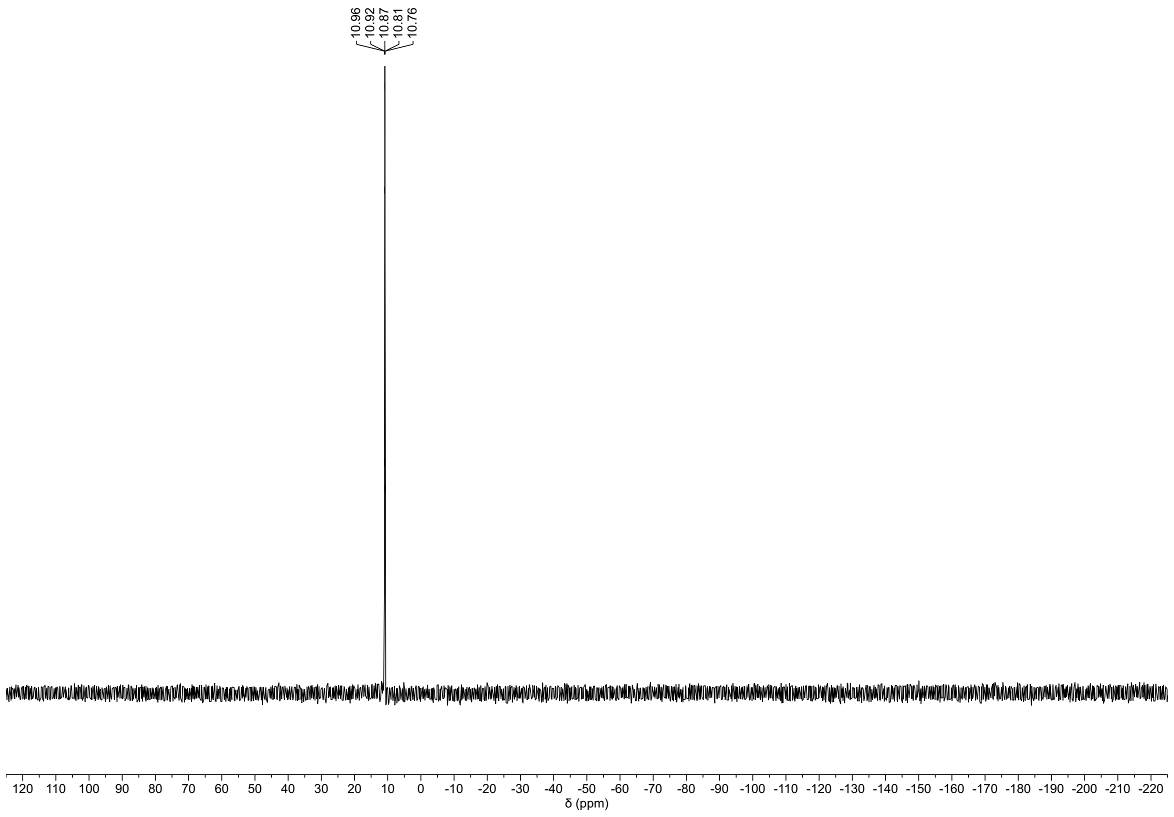

Figure 102. ^{31}P NMR – Dimethyl pyrazin-2-ylphosphonate (**7t**), 202 MHz, $CDCl_3$.

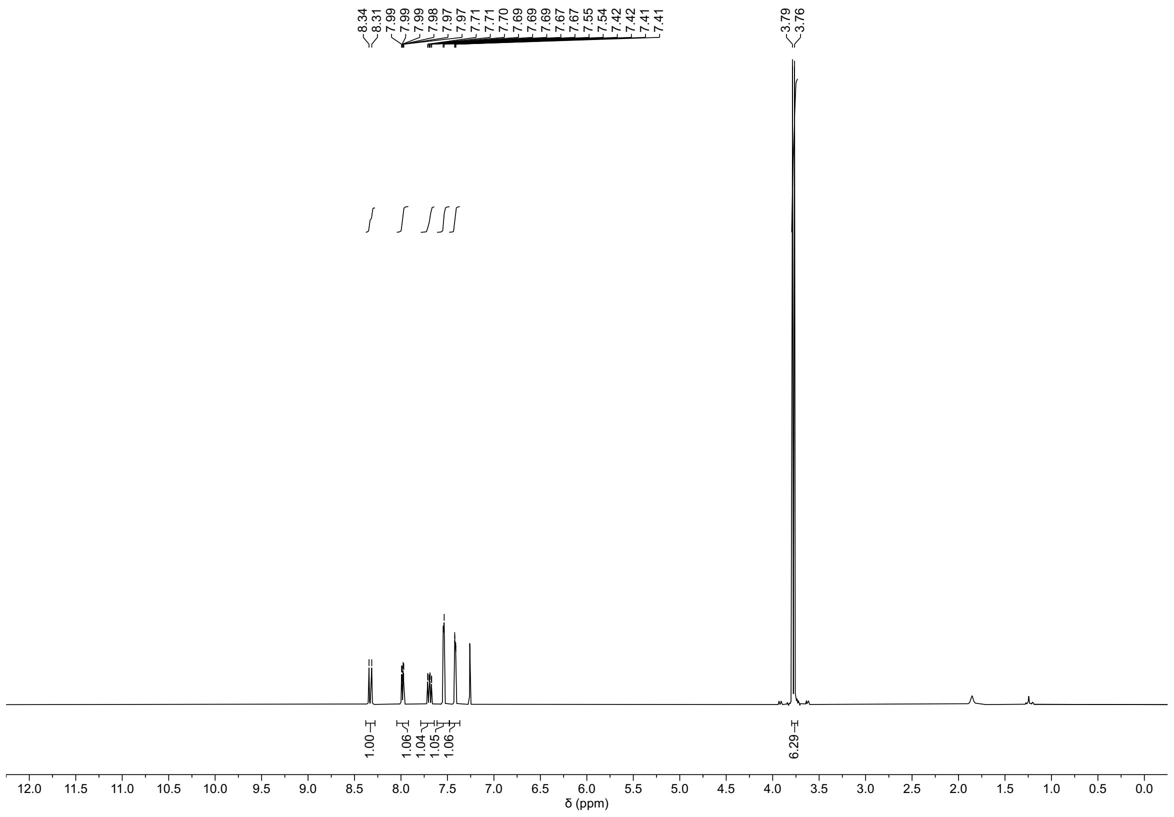

Figure 103. ^{1}H NMR – Dimethyl benzo[*b*]thiophen-5-ylphosphonate (**7u**), 500 MHz, $CDCl_3$.

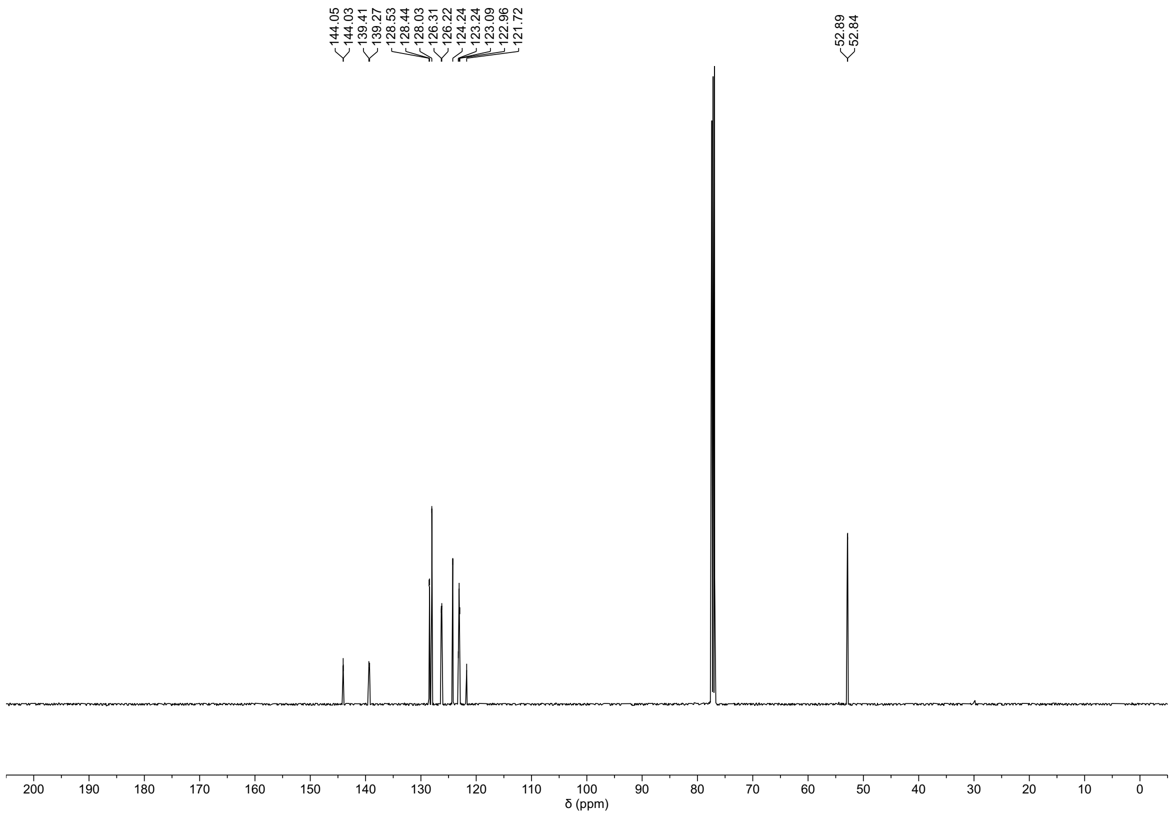

Figure 104. ^{13}C NMR – Dimethyl benzo[*b*]thiophen-5-ylphosphonate (**7u**), 126 MHz, $CDCl_3$.

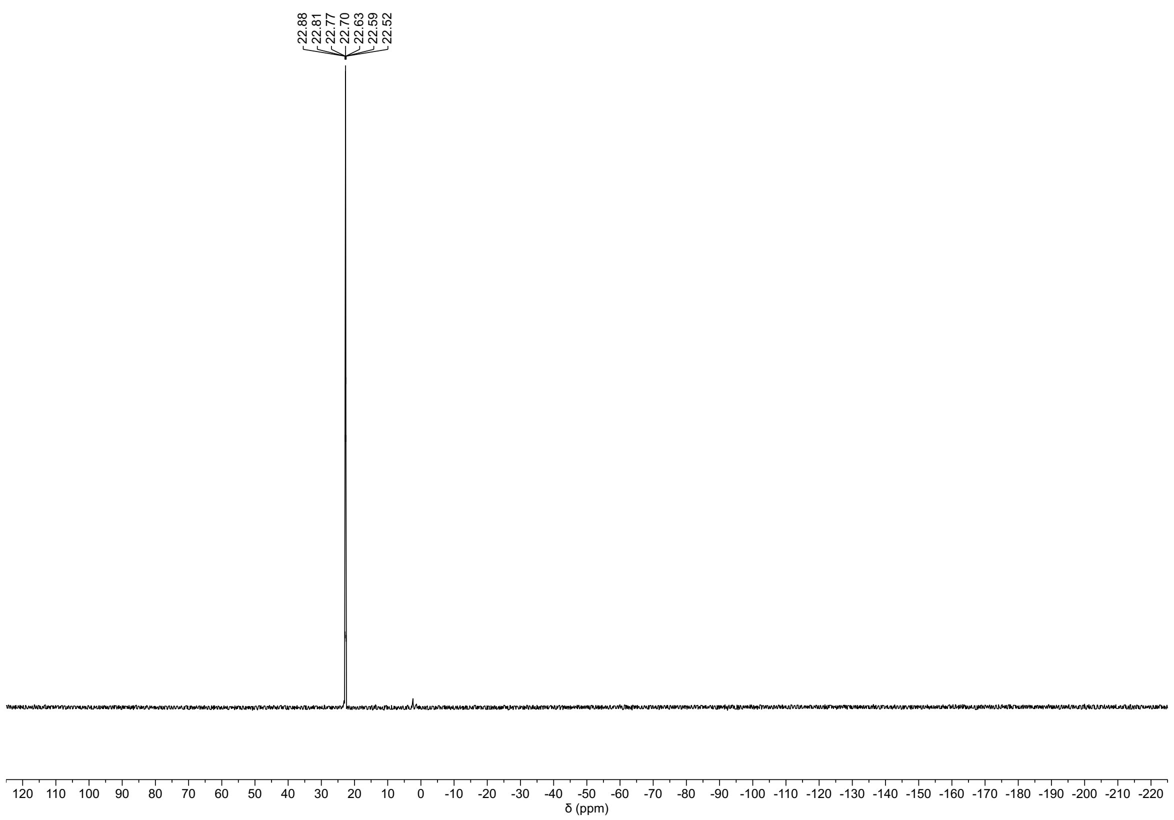

Figure 105. ^{31}P NMR – Dimethyl benzo[*b*]thiophen-5-ylphosphonate (**7u**), 202 MHz, $CDCl_3$.

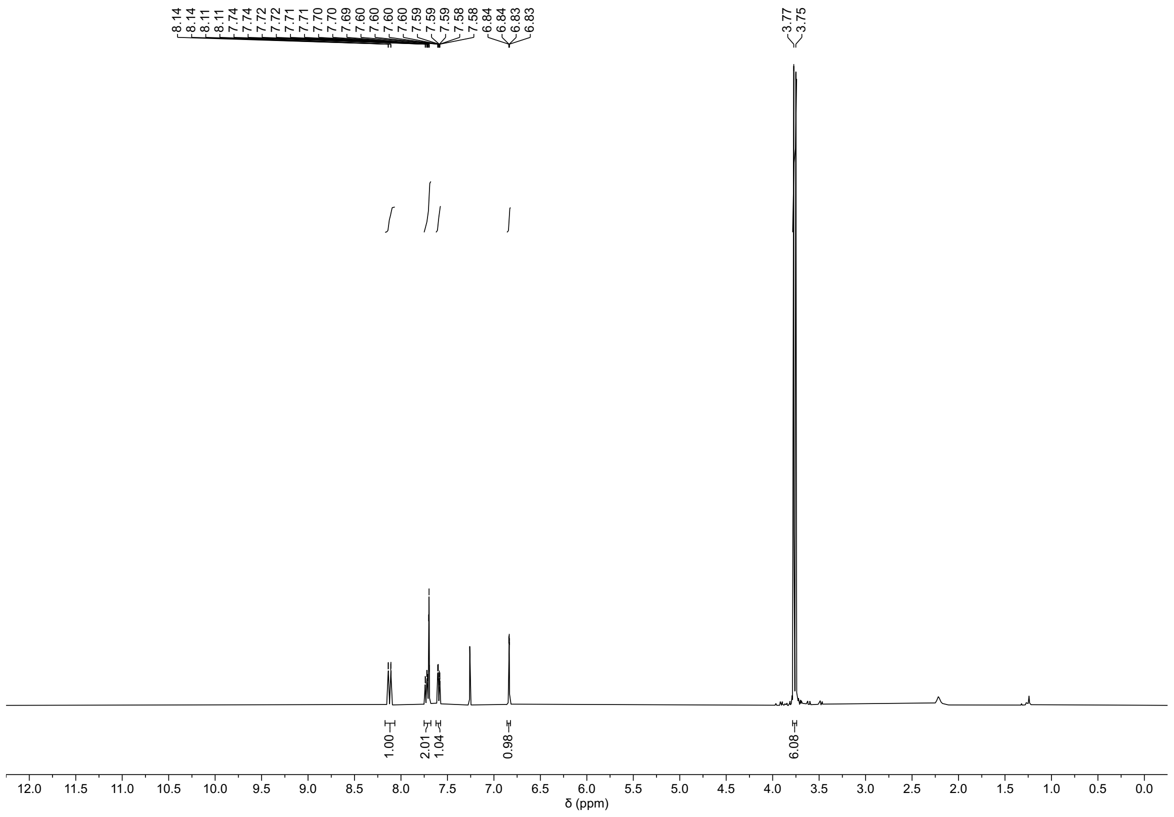

Figure 106. ^{1}H NMR – Dimethyl benzofuran-5-ylphosphonate (**7v**), 500 MHz, $CDCl_3$.

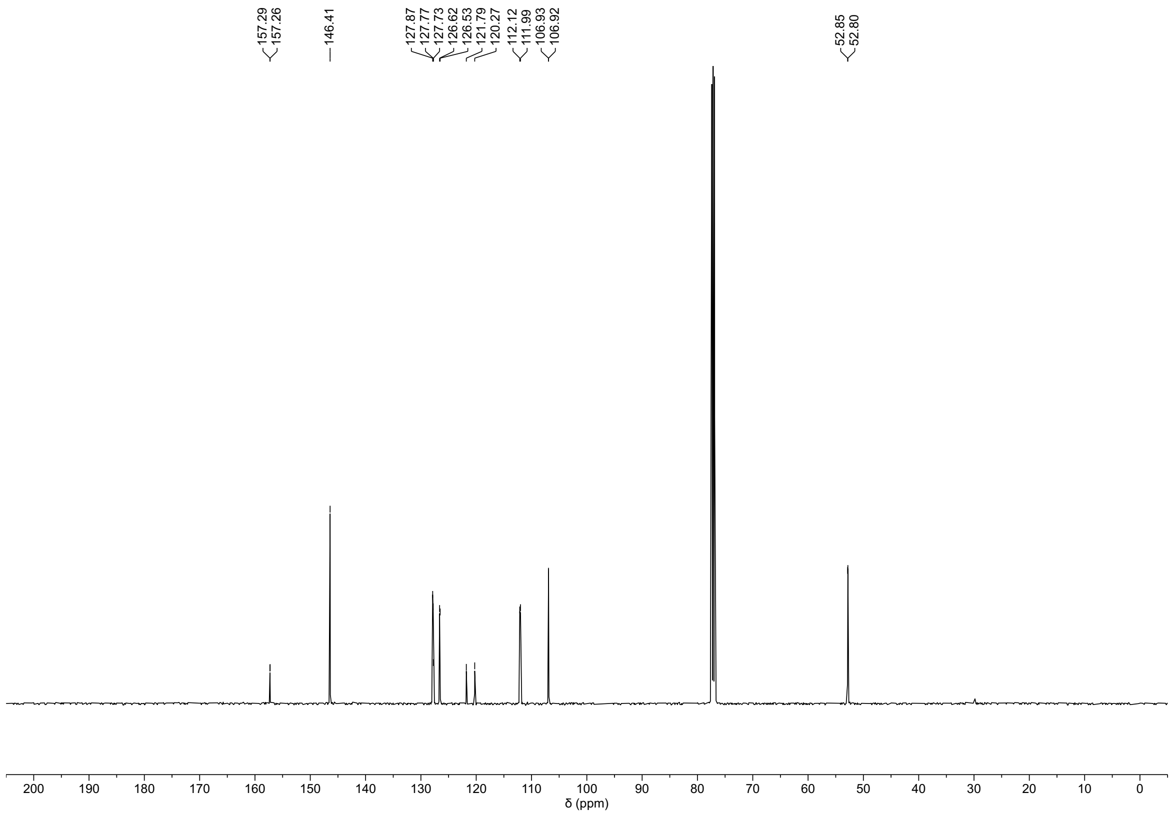

Figure 107. ^{13}C NMR – Dimethyl benzofuran-5-ylphosphonate (**7v**), 126 MHz, $CDCl_3$.

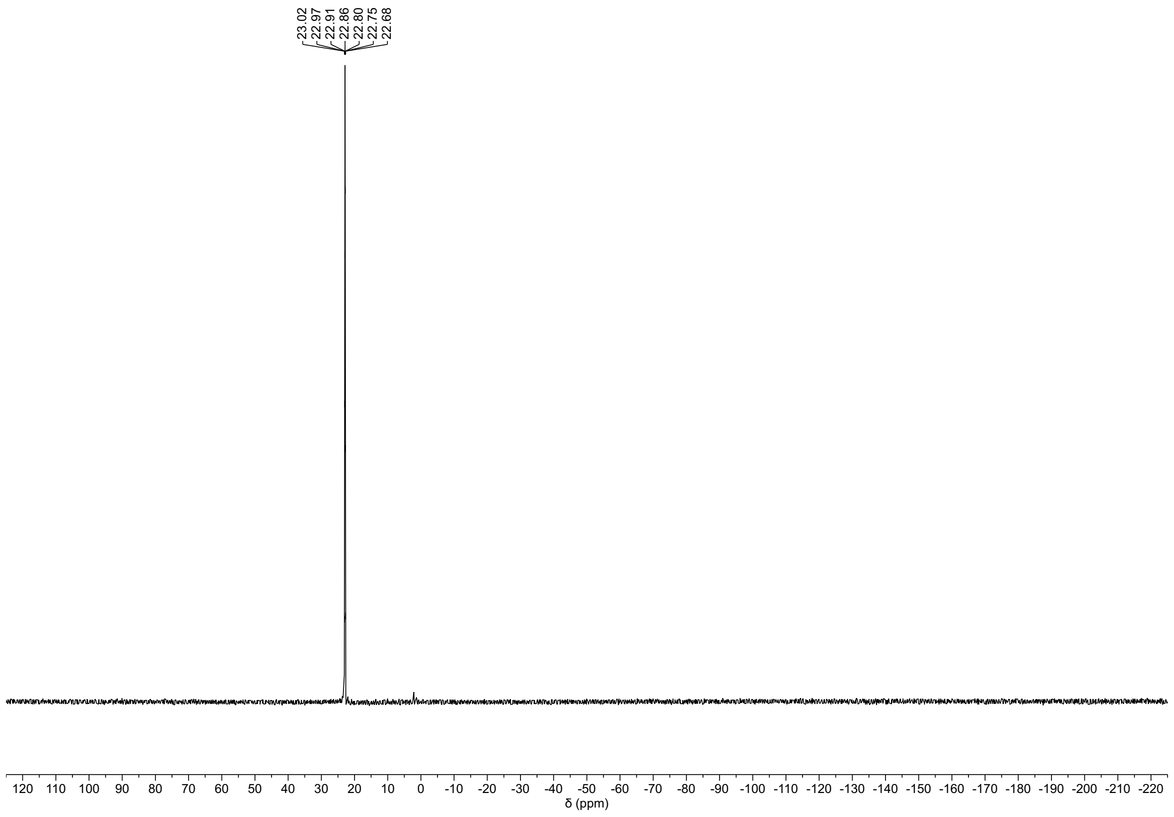

Figure 108. ^{31}P NMR – Dimethyl benzofuran-5-ylphosphonate (**7v**), 202 MHz, $CDCl_3$.

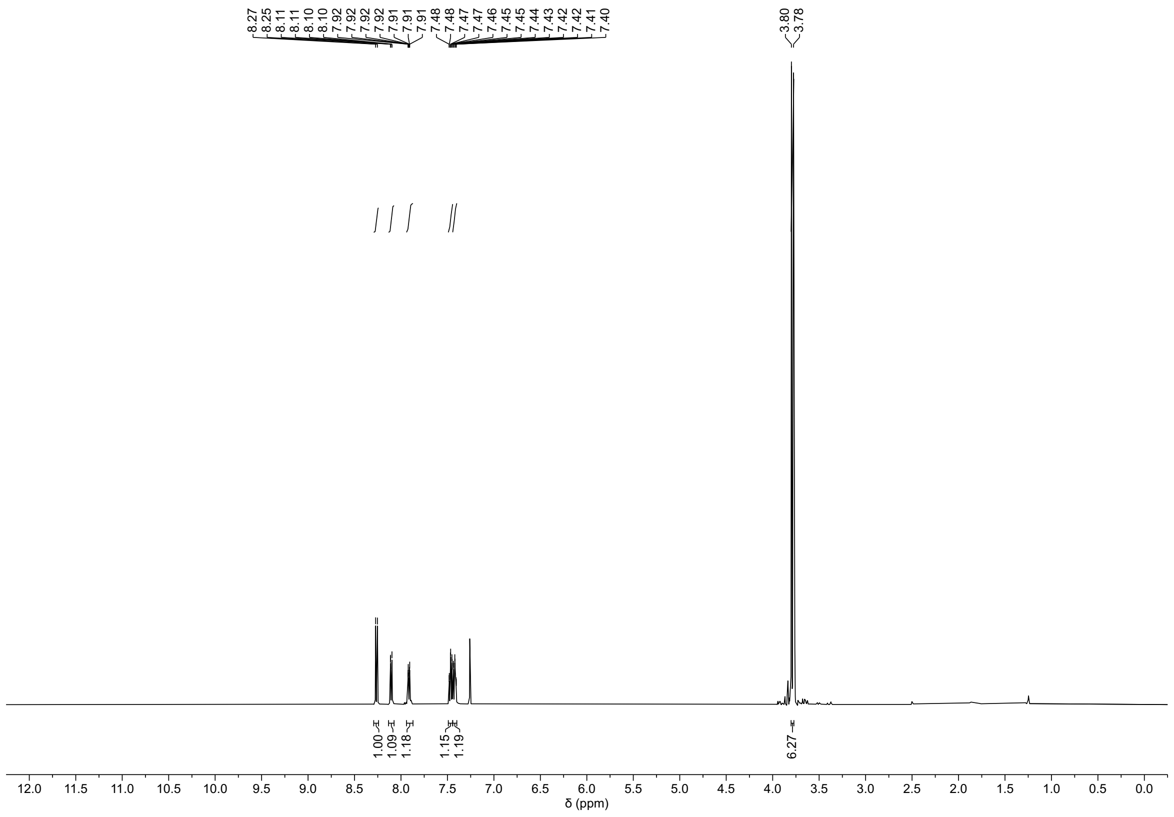

Figure 109. ^{1}H NMR – Dimethyl benzofuran-3-ylphosphonate (**7w**), 500 MHz, $CDCl_3$.

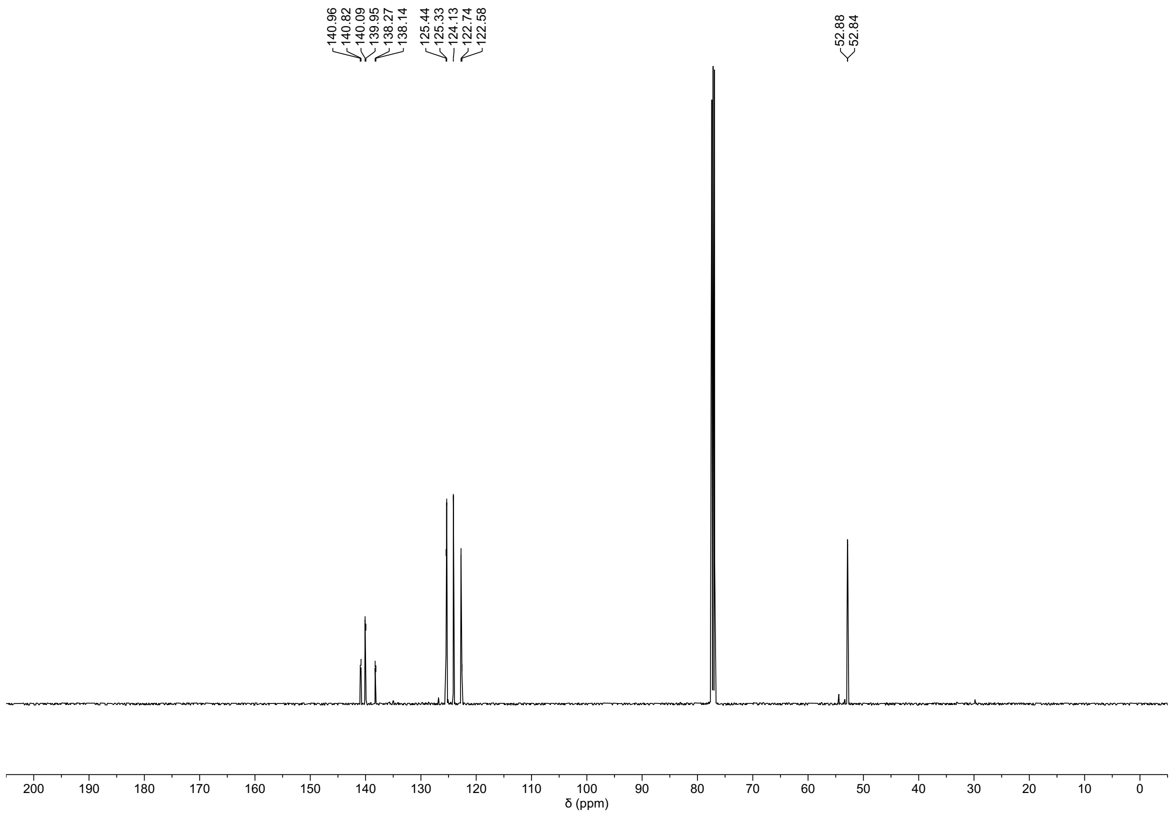

Figure 110. ^{13}C NMR – Dimethyl benzofuran-3-ylphosphonate (**7w**), 126 MHz, $CDCl_3$.

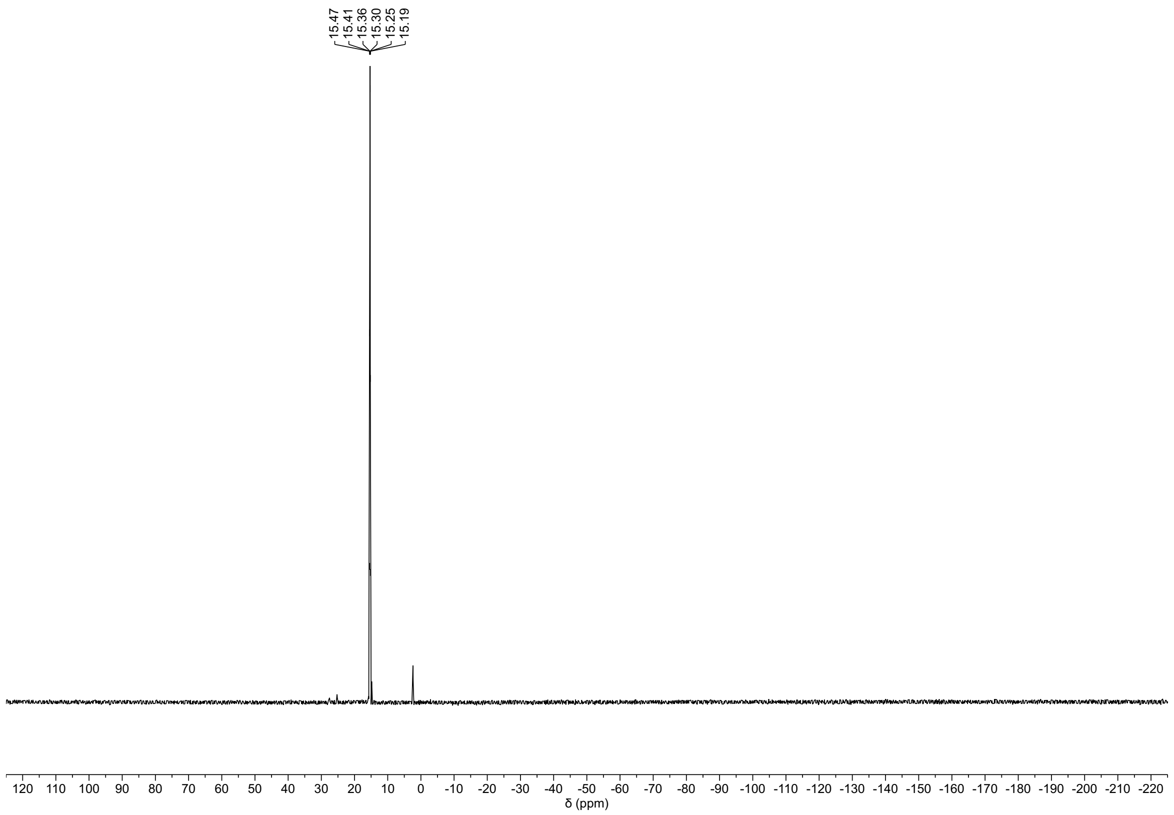

Figure 111. ^{31}P NMR – Dimethyl benzofuran-3-ylphosphonate (**7w**), 202 MHz, $CDCl_3$.

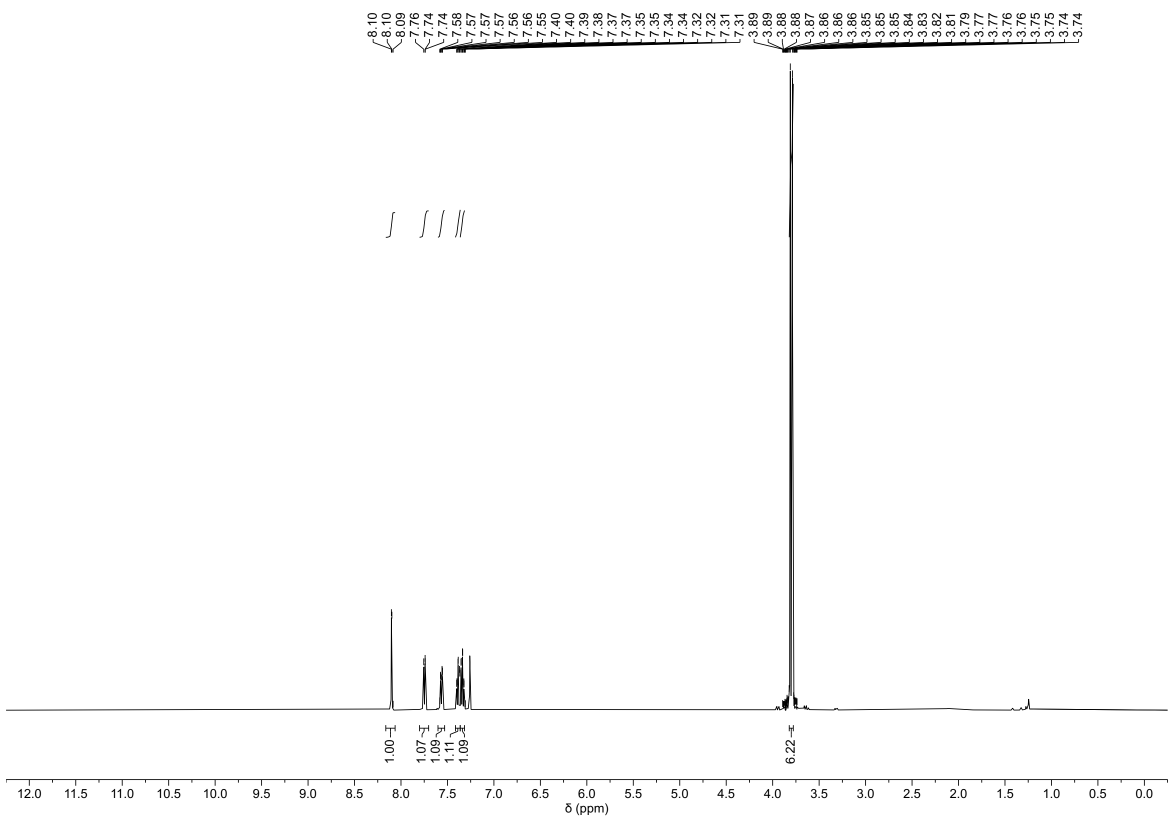

Figure 112. ^{1}H NMR – Dimethyl benzofuran-3-ylphosphonate (**7x**), 500 MHz, $CDCl_3$.

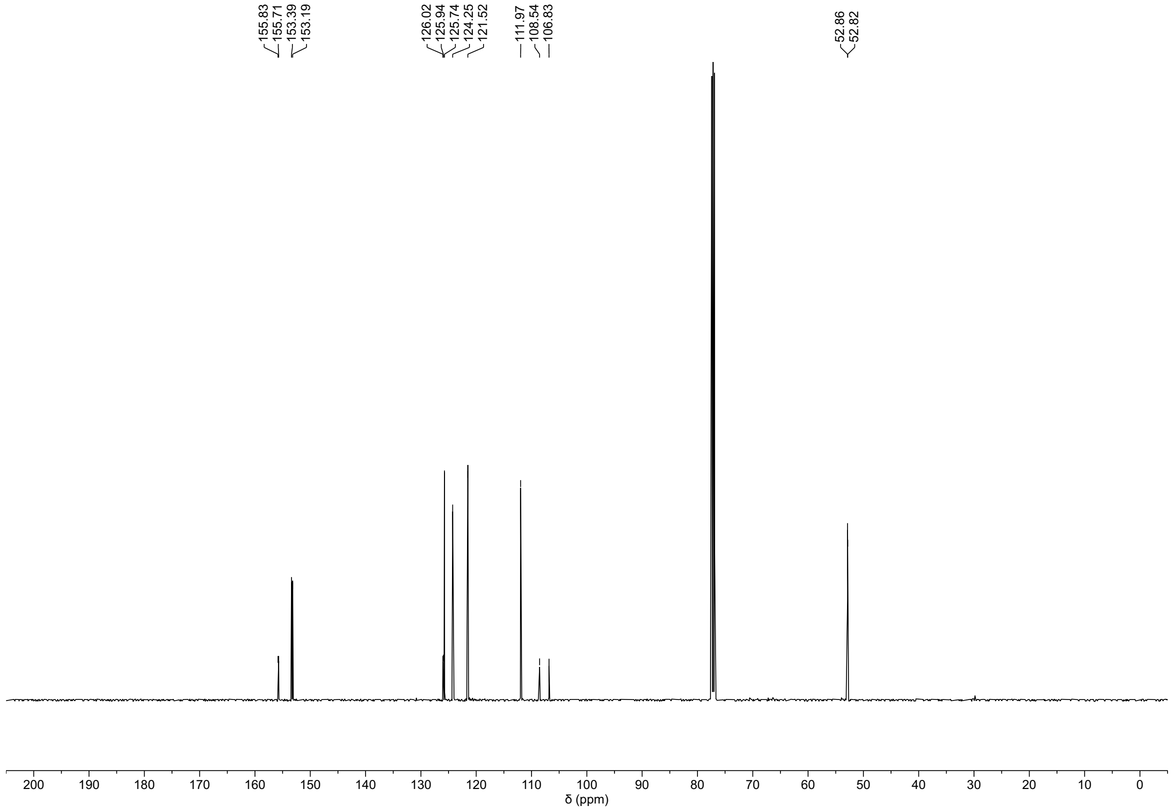

Figure 113. ^{13}C NMR – Dimethyl benzofuran-3-ylphosphonate (**7x**), 126 MHz, $CDCl_3$.

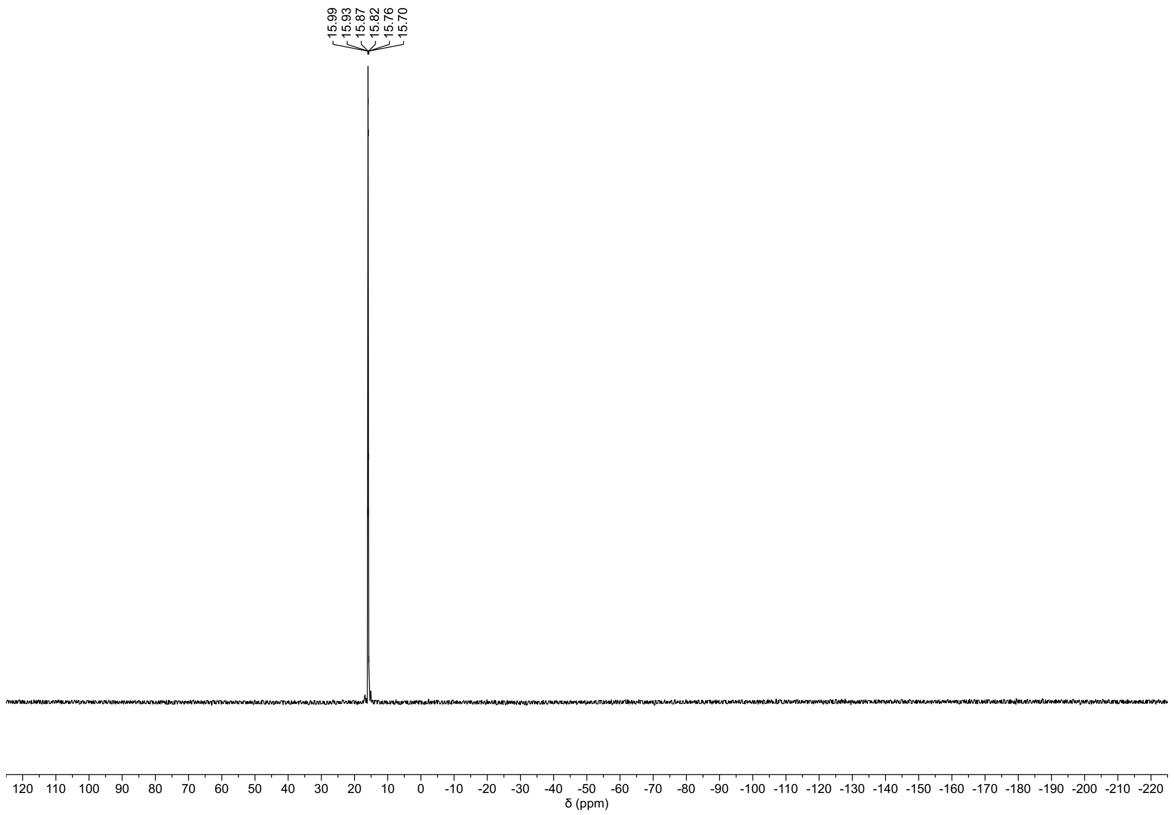

Figure 114. ^{31}P NMR – Dimethyl benzofuran-3-ylphosphonate (**7x**), 202 MHz, $CDCl_3$.

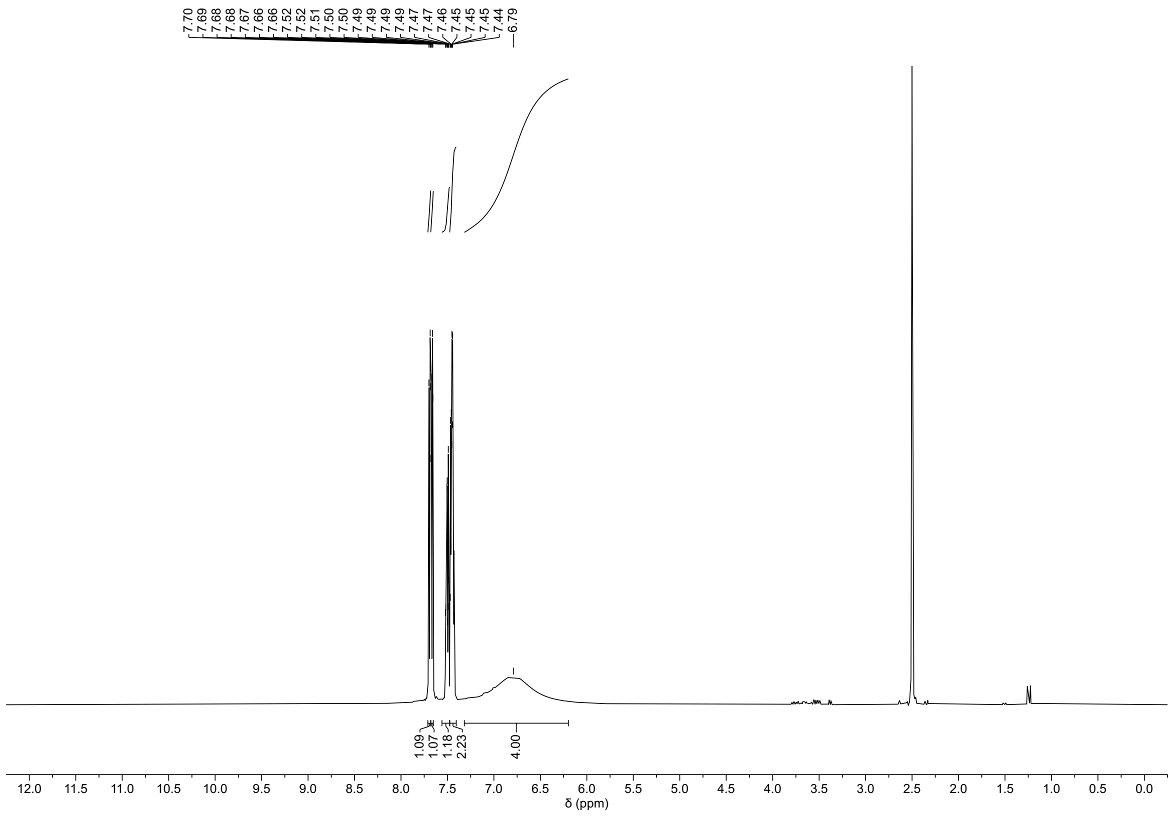

Figure 115. ^{1}H NMR – Phenylphosphonic acid (**8a**), 500 MHz, $CDCl_3$.

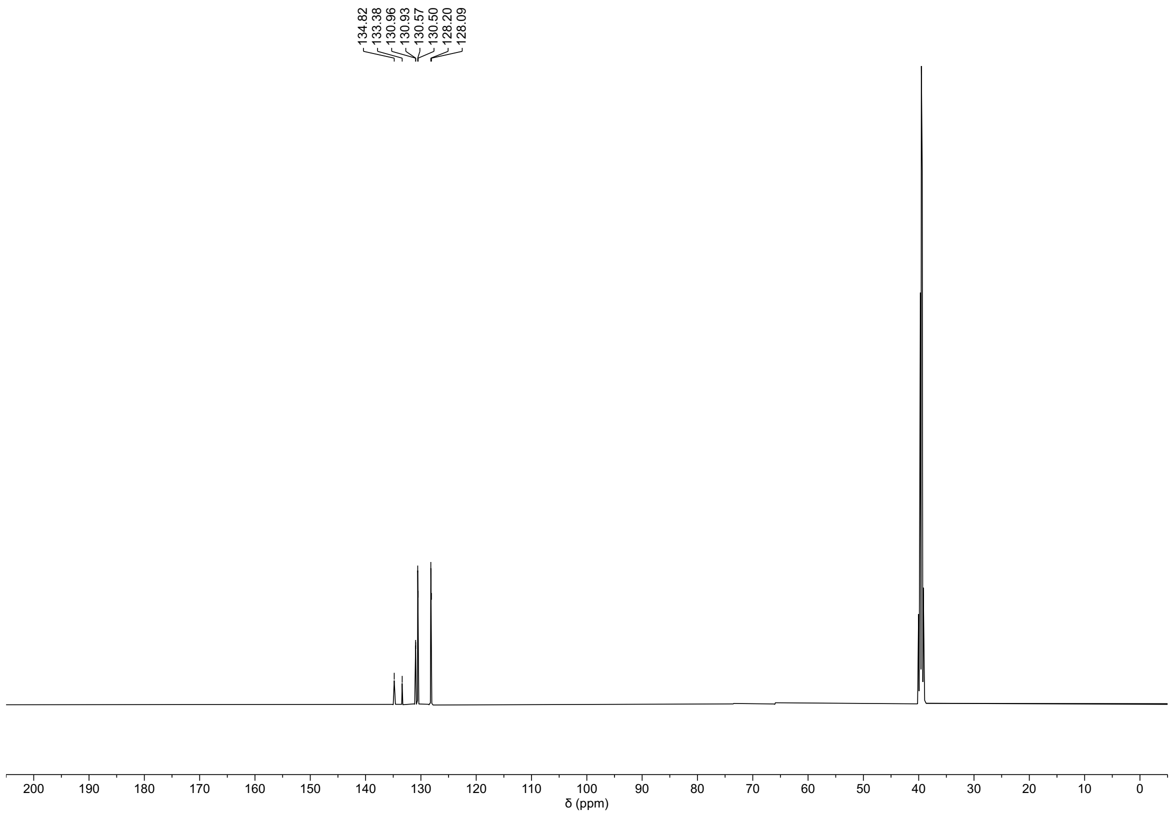

Figure 116. ^{13}C NMR – Phenylphosphonic acid (**8a**), 126 MHz, $CDCl_3$.

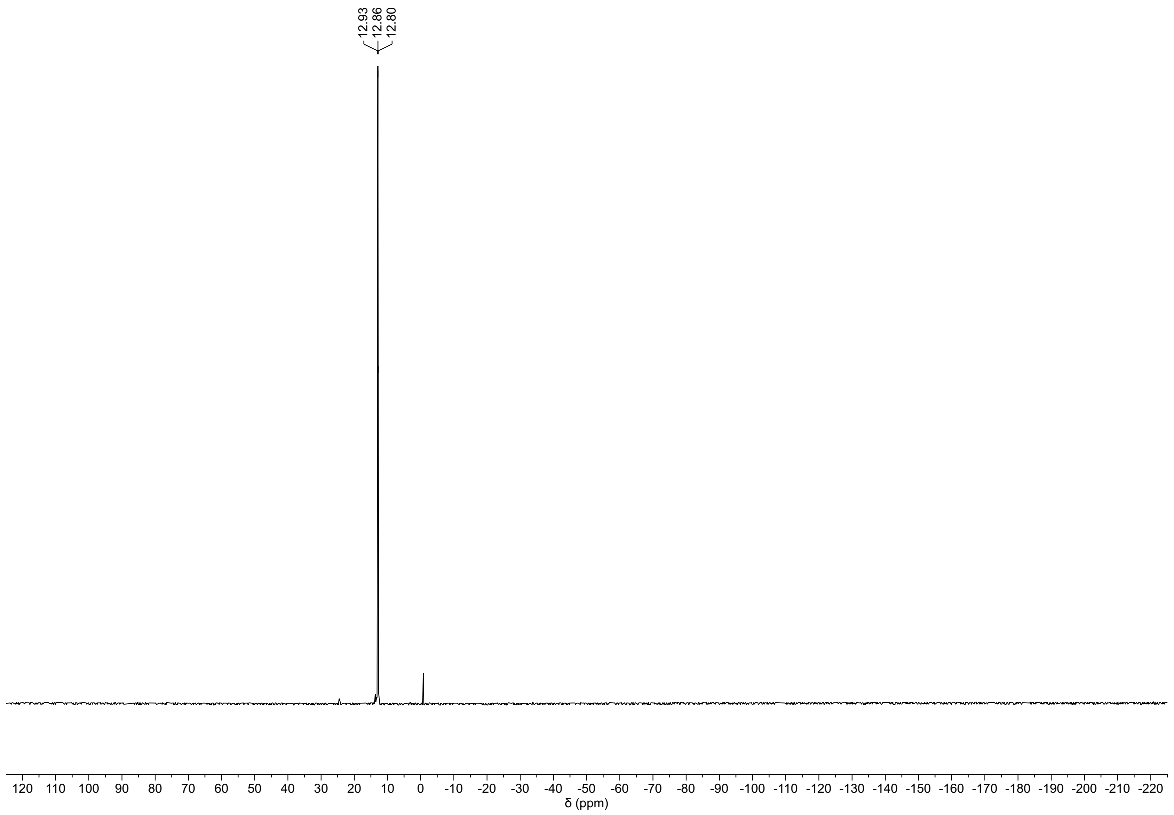

Figure 117. ^{31}P NMR – Phenylphosphonic acid (**8a**), 202 MHz, $CDCl_3$.

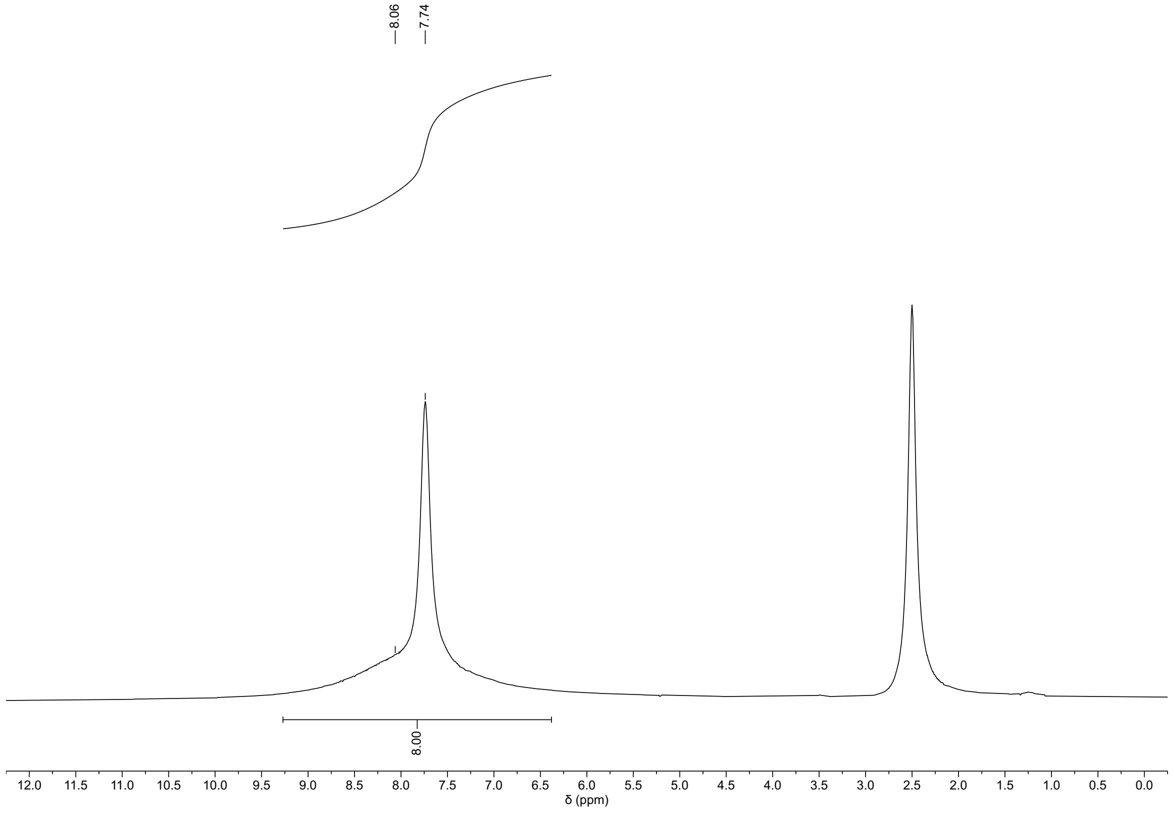

Figure 118. ^{1}H NMR – (4-Phosphonophenyl)phosphonic acid (**8b**), 400 MHz, $CDCl_3$.

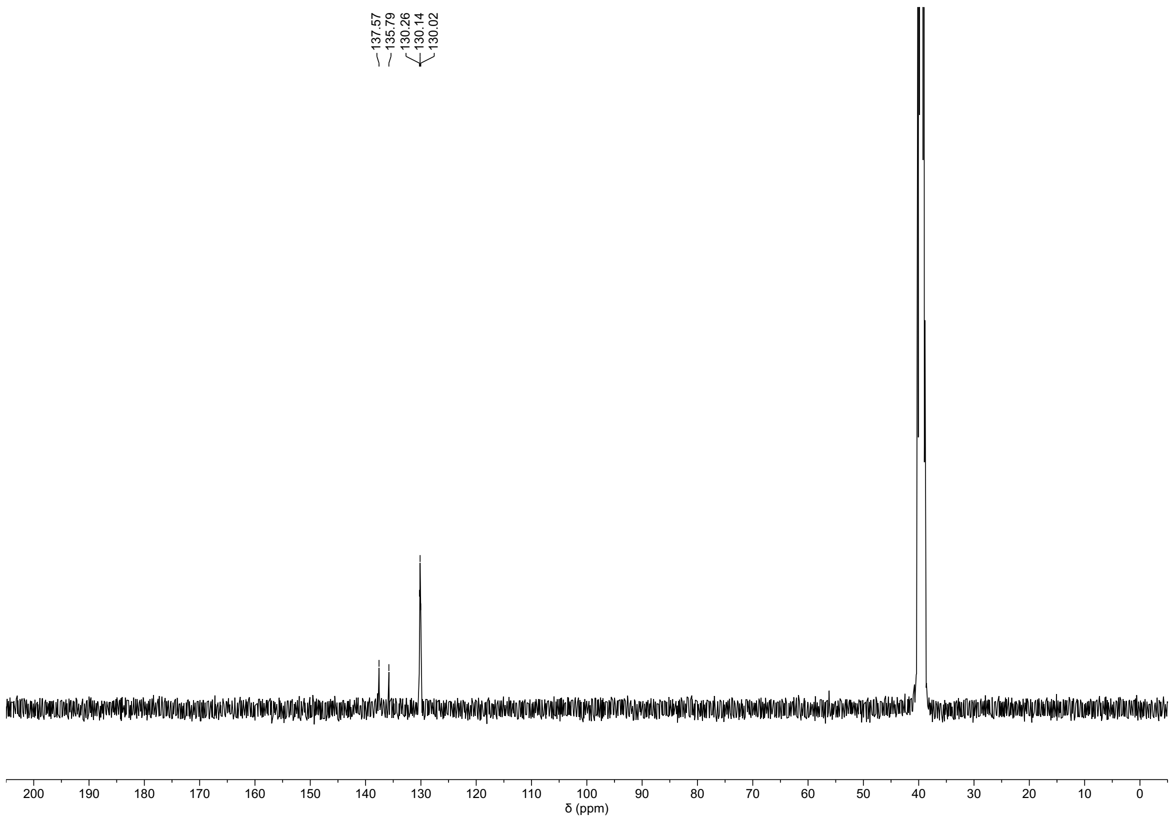

Figure 119. ^{13}C NMR – (4-Phosphonophenyl)phosphonic acid (**8b**), 101 MHz, $CDCl_3$.

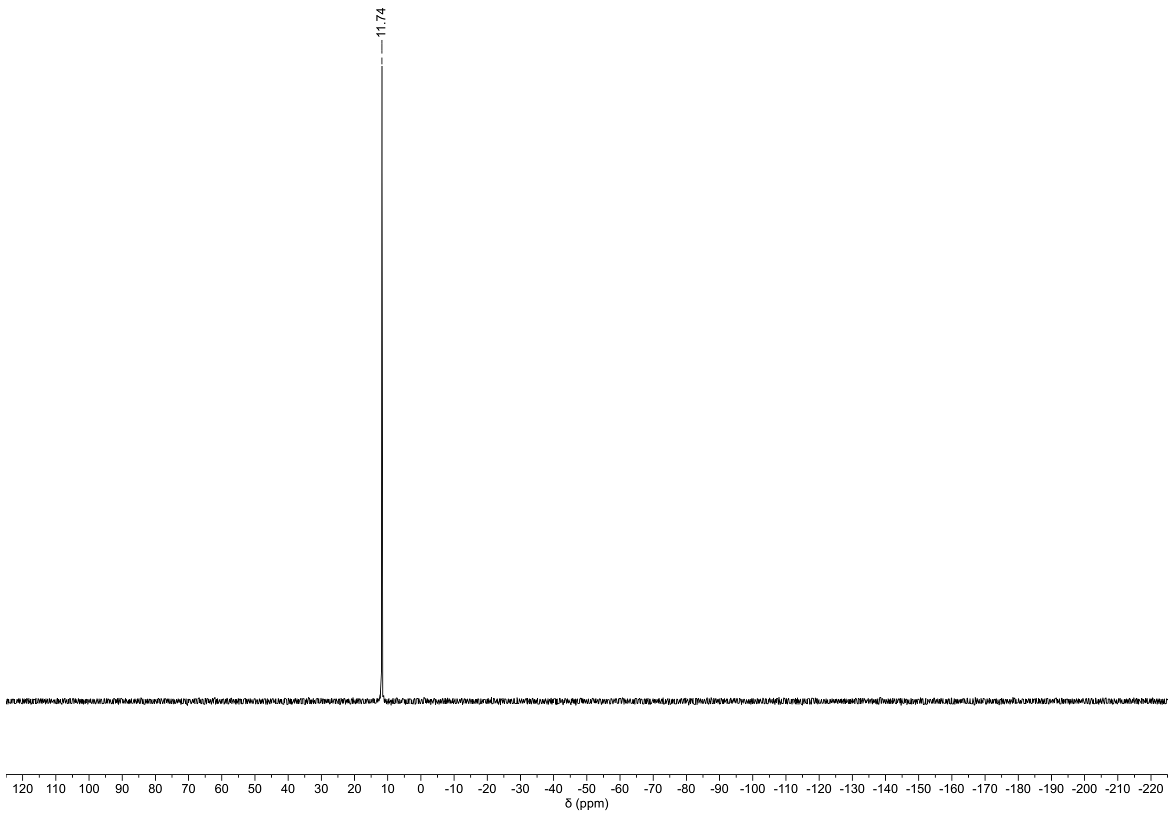

Figure 120. ^{31}P NMR – (4-Phosphonophenyl)phosphonic acid (**8b**), 162 MHz, $CDCl_3$.

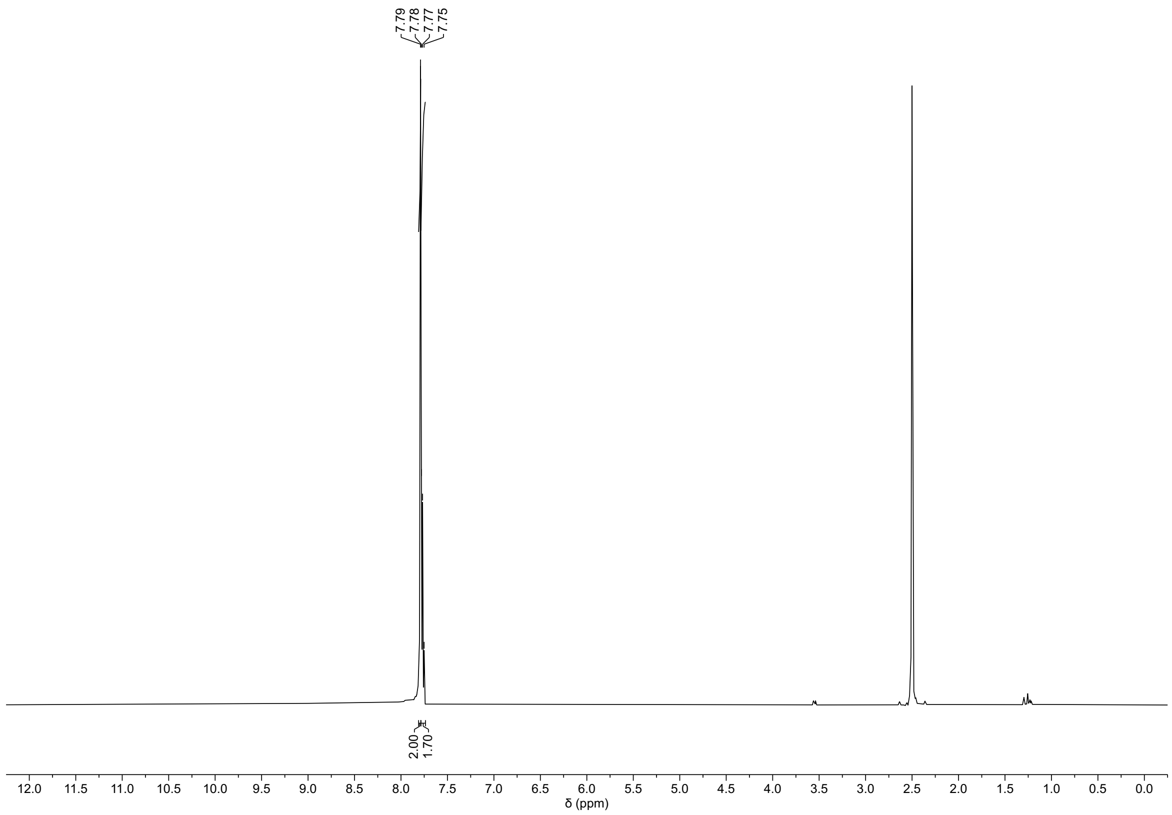

Figure 121. ^{1}H NMR – [4-(4-Phosphonophenyl)phenyl]phosphonic acid (**8c**), 500 MHz, $CDCl_3$.

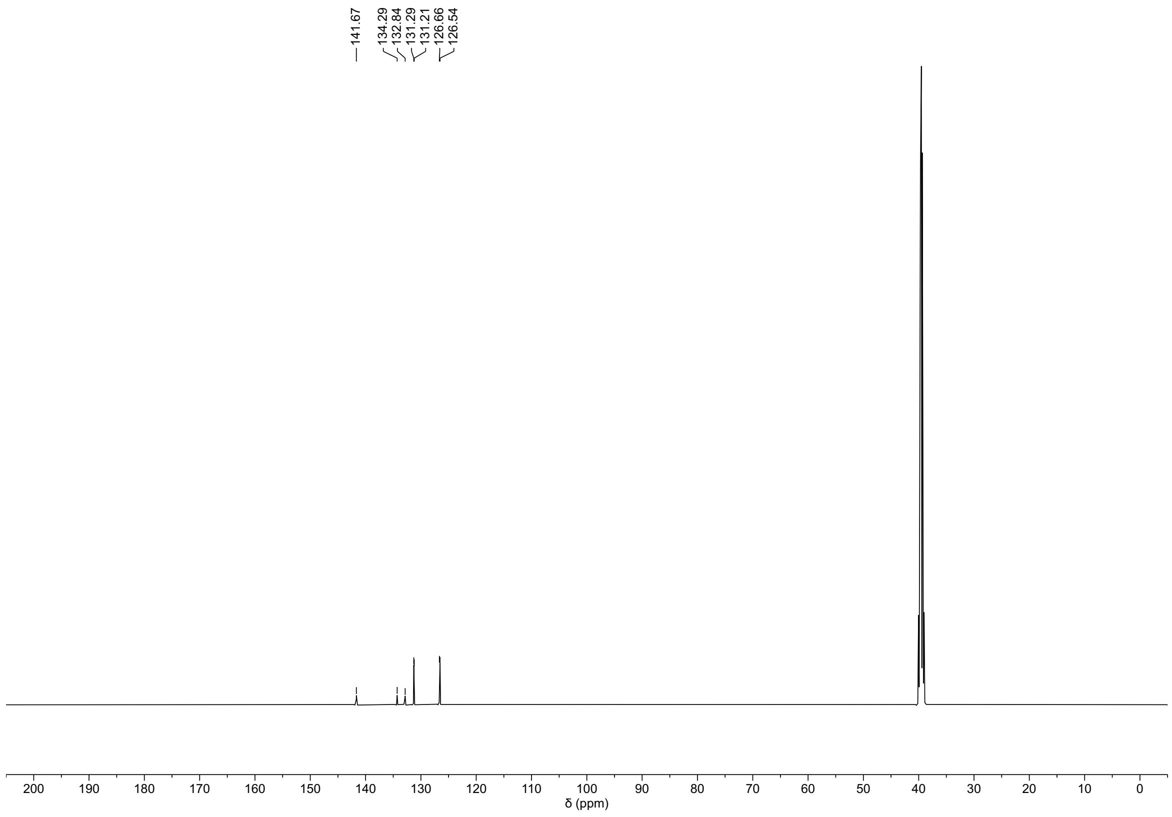

Figure 122. ^{13}C NMR – [4-(4-Phosphonophenyl)phenyl]phosphonic acid (**8c**), 126 MHz, $CDCl_3$.

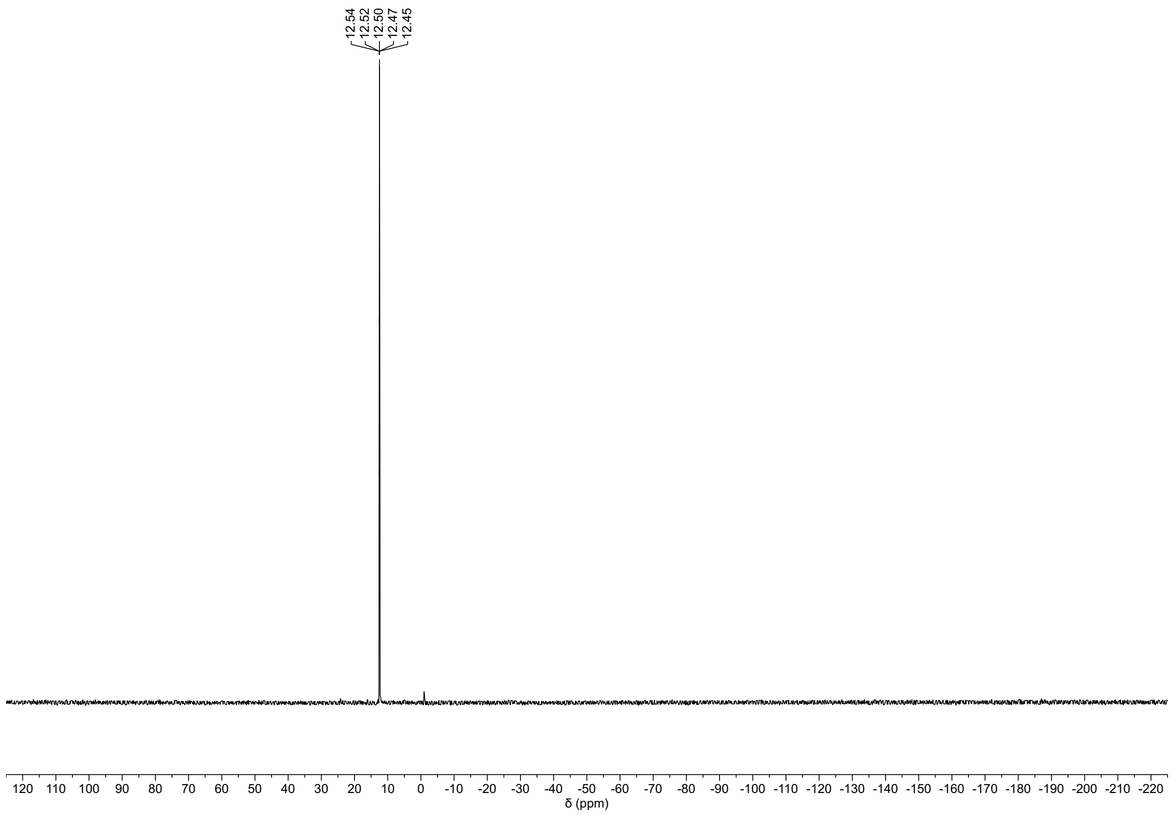

Figure 123. ^{31}P NMR – [4-(4-Phosphonophenyl)phenyl]phosphonic acid (**8c**), 202 MHz, $CDCl_3$.

4. Reaction Optimization

Table 1. Optimization of the photo-Arbuzov reaction of bromobenzene with trimethyl phosphite.

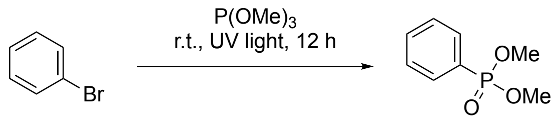

entry	$P(OMe)_3$ (equiv.)	solvent	M of 6a-Br	yield (%)[a]
1	200	–	–	61
2	10.0	1,4-dioxane	0.2	77
3[b]	10.0	1,4-dioxane	0.2	–
4	5.00	1,4-dioxane	0.2	97
5	2.50	1,4-dioxane	0.2	32
6	1.10	1,4-dioxane	0.2	traces
7	5.00	MeOH	0.2	39
8	5.00	MeCN	0.2	46
**9**	**5.00**	**1,4-dioxane**	**1.0**	**96**
10	5.00	1,4-dioxane	2.0	95

Bromobenzene (0.42 mmol), UV light (245 nm, 224 W), r.t, 18 h. [a] determined *via* inverse-gated $^{31}P\{^1H\}$ NMR with trimethylphosphate as internal standard . [b] blue LED (471 nm, 30 W), blacklight (368 nm, 160 W), or LED UV-lamps (385 nm, 64 W) in combination with borosilicate flasks were used.

5. UV-Reactor Setup

For the reaction, a Rayonet® RPR-100 photoreactor by The Southern New England Ultraviolet Co. was used (**Figure 124**). The reactor is equipped with 16 x 14 W PRP-2537A, low-pressure mercury lamps (>80% of the emission occurs at 254 nm). The reaction was set up in a 50 mL quartz glass round-bottom flask and clamped so that is was aligned to the center of the reactor.

To ensure eye protection, when reactor was running, orange toned safety googles (e.g., 3M™ SecureFit™ 400 SF406SGAF-BLA) were used. During the reaction, the outside was shielded from the diffuse UV light by a shield made out of cardboard.

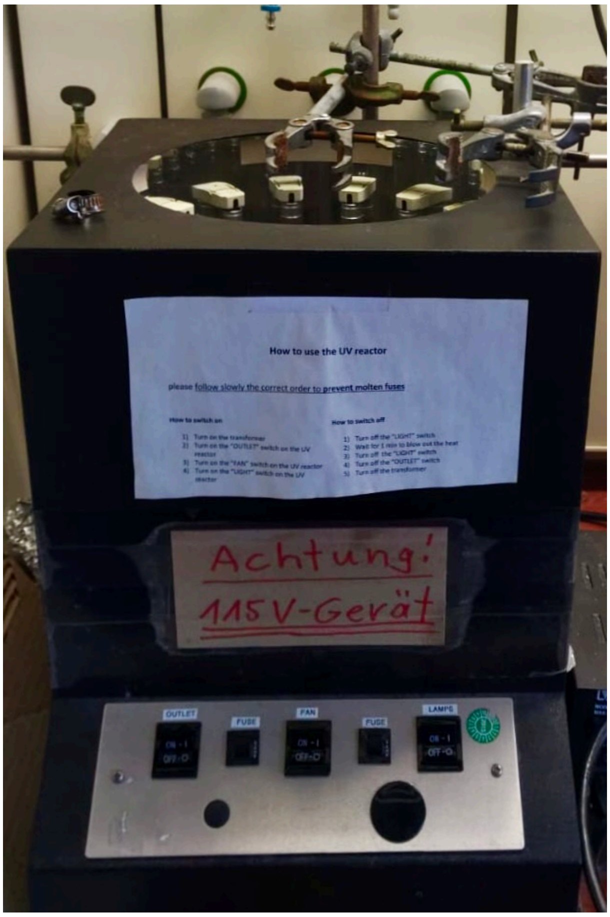

Figure 124. Rayonet® RPR-100 equipped with 16 x 14 W RPR-2537A lamps.

6. Tentative Mechanism

The domino-type reactivity which was observed for some *para*-substituted derivatives supports the suggested mechanism, which involves a radical-nucleophilic aromatic substitution ($S_{RN}1$) reaction in the first step (**Scheme 1**). This is either followed by de-alkylation (path A) or by S_NAr reaction with a second trialkyl phosphite molecule if an adequate leaving group is present in *para*-position (path B).

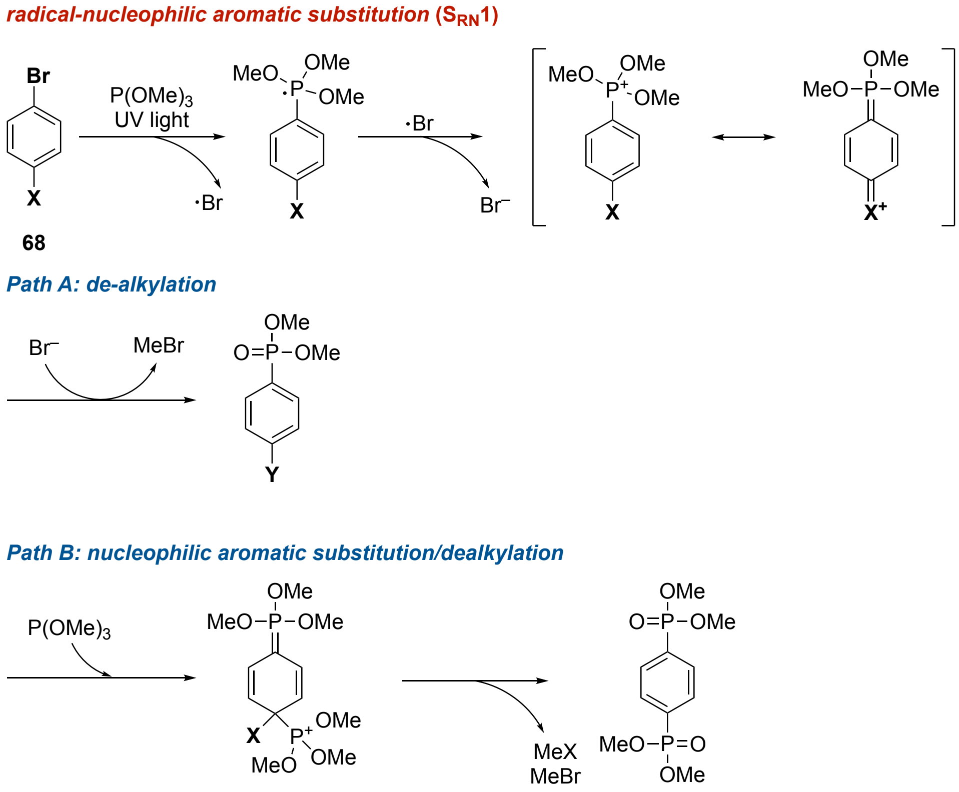

Scheme 1. Suggested mechanism for the photo-Arbuzov reaction of *para*-substituted aryl halides.
